# Design, Synthesis and Anticancer Evaluation of Novel SO_2_F-Containing Medicinally Privileged Structures *via* Ketone-Directed C–H Functionalization

**DOI:** 10.3390/molecules31173094

**Published:** 2026-09-03

**Authors:** Seungwan Kim, Lingayya Rajaka, Saegun Kim, In Jae Bang, Hyun Woo Lee, Minseo Choi, Jihyo Lee, Myunghwa Jung, Hyosik Woo, Sangil Han, Ki Yong Lee, Hyun Jin Kim, Ha Ryong Kim, Sang Hoon Han

**Affiliations:** 1 College of Pharmacy, Korea University, Sejong 30019, Republic of Korea; kjyk0813@naver.com (S.K.); lingamaiahchem@gmail.com (L.R.);; 2 Institute of Pharmaceutical Science and Translational Research, Korea University, Sejong 30019, Republic of Korea; 3 College of Pharmacy, Research Institute of Pharmaceutical Sciences, Sookmyung Women’s University, Seoul 04310, Republic of Korea; saegunkim@sookmyung.ac.kr; 4 Data Convergence Drug Research Center, Therapeutics and Biotechnology Division, Korea Research Institute of Chemical Technology (KRICT), Daejeon 34114, Republic of Korea; 5 Department of Innovative Pharmaceutical Sciences, Kyungpook National University, Daegu 41566, Republic of Korea; 6 Interdisciplinary Major Program in Innovative Pharmaceutical Sciences, Korea University, Sejong 30019, Republic of Korea; 7 Medicinal Chemistry and Pharmacology, Korea National University of Science and Technology, Daejeon 34113, Republic of Korea

**Keywords:** ketone-directed C–H functionalization, chromone derivatives, xanthone derivatives, ethenesulfonyl fluoride, cytotoxic activity

## Abstract

The weakly coordinating ketone group-directed C–H functionalizations of chromones, flavones, 1,4-naphthoquinones, (thio)xanthones, and acridones with ethenesulfonyl fluoride under rhodium(III) or ruthenium(II) catalysis are described. These protocols efficiently provide a range of chromone-based scaffolds and xanthone analogues bearing an SO_2_F group with excellent site-selectivity and functional group compatibility. The practicality of this approach was demonstrated by simple SuFEx conjugations with representative biologically relevant molecules, DNA conjugation, and Michael addition with a secondary amine. All the synthesized derivatives were initially screened for their *in vitro* cytotoxic activity against human lung adenocarcinoma A549 cells. In particular, compound **5aa** with a xanthone scaffold was found to be highly cytotoxic.

## 1. Introduction

**Figure 1:**
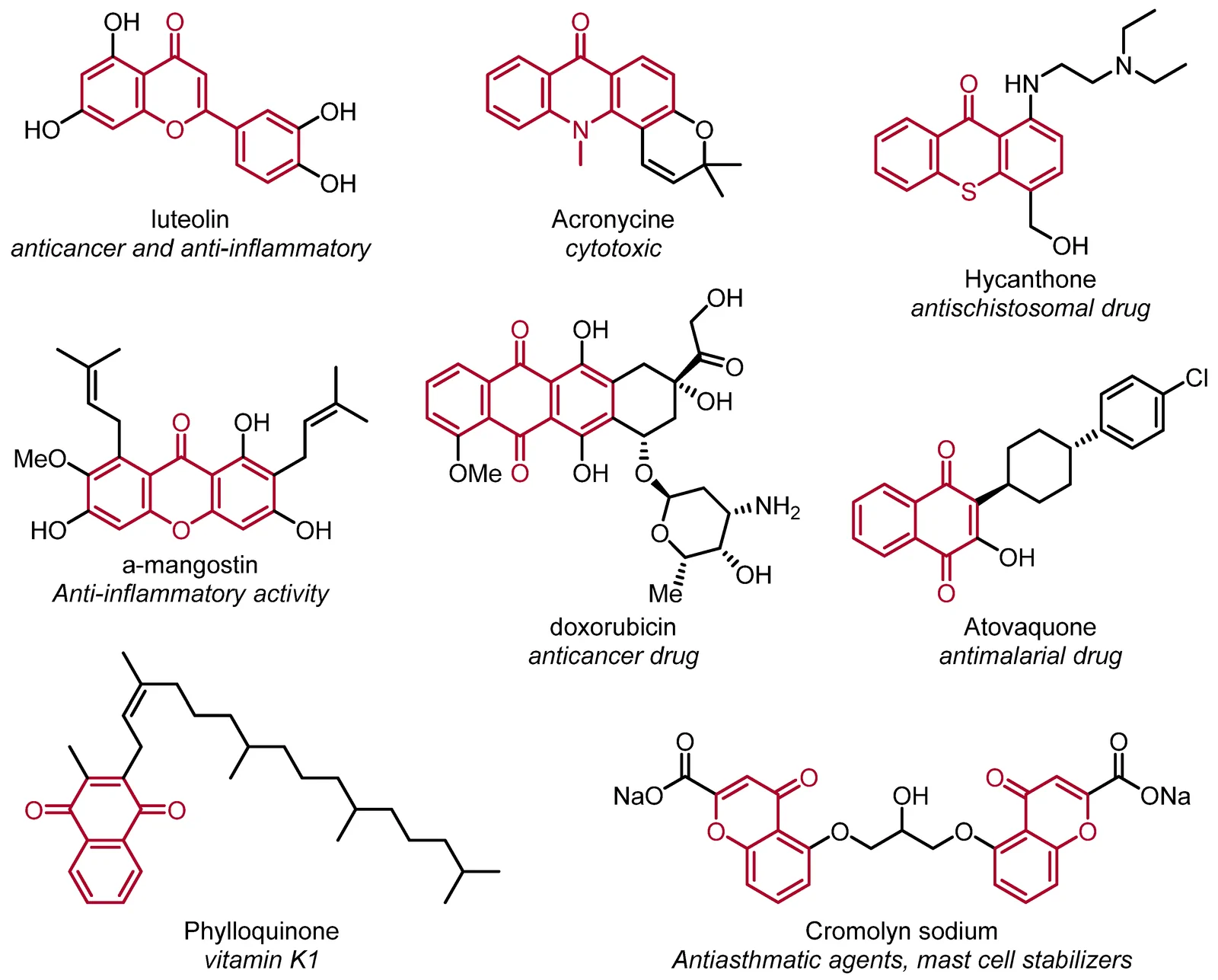
Representative and biologically relevant chromones, flavones, xanthones, thioxanthones, acridones, and 1,4-naphthoquinones.

Natural product-inspired scaffolds such as chromones, flavones, xanthones, thioxanthones, acridones, and 1,4-naphthoquinones constitute an important class of privileged structures that are widely distributed in natural products, pharmaceuticals, and biologically active small molecules [1,2,3,4,5]. These scaffolds exhibit diverse biological activities, including anticancer, antibacterial, antiviral, antioxidant, and anti-inflammatory effects (Figure 1) [1,2,3,4,5,6,7,8,9,10,11]. Consequently, considerable efforts have been devoted to the synthesis of structurally diversified analogues for structure-activity relationship (SAR) studies and the discovery of new therapeutic agents [12,13]. Despite their biological significance, efficient methods for the direct diversification of these medicinally relevant heterocyclic frameworks remain highly desirable.

Transition-metal-catalyzed C–H functionalization has emerged as one of the most powerful and atom-economical strategies for the direct modification of complex molecules [14]. In particular, Rh(III)- and Ru(II)-catalyzed ketone-directed C–H activation has enabled the site-selective functionalization of chromones, flavones, xanthones, quinones, and related heterocyclic scaffolds, providing rapid access to structurally diverse analogues without requiring prefunctionalized substrates [15,16,17,18]. Numerous transformations, such as alkylation, amidation, and amination, have been successfully developed over the past decade [19]. Nevertheless, these methods have primarily focused on structural diversification, while comparatively little attention has been paid to the installation of synthetically versatile functional groups that enable further downstream derivatization.

Among recently developed coupling partners, ethenesulfonyl fluoride (ESF) has emerged as a particularly attractive bifunctional synthetic building block [20]. In addition to serving as an electron-deficient alkene for transition-metal-catalyzed C–C bond formation, ESF introduces a sulfonyl fluoride (SO_2_F) functionality that remains intact throughout the coupling process [21,22]. The retained SO_2_F group serves as a versatile handle for Sulfur(VI) Fluoride Exchange (SuFEx) chemistry, a highly efficient click reaction that has attracted increasing attention in synthetic chemistry, medicinal chemistry, and chemical biology [23,24]. Owing to its unique combination of chemical stability and controllable reactivity, the sulfonyl fluoride functionality enables rapid and selective post-synthetic diversification with a variety of biologically relevant nucleophiles [25]. Consequently, incorporation of an SO_2_F handle into biologically privileged scaffolds provides a unique opportunity to combine molecular diversification with subsequent functionalization through SuFEx chemistry. This modular derivatization substantially expands the synthetic utility of the resulting molecules and offers opportunities for future bioconjugation applications, including peptide conjugation, protein modification, and the development of antibody-drug conjugates (ADCs) [26]. Despite these attractive features, the integration of ESF-mediated C–H functionalization with medicinally important heterocyclic scaffolds remains largely unexplored [27].

Herein, we report Rh(III)- or Ru(II)- catalyzed ketone-directed C–H olefination of chromones, flavones, (thio)xanthone derivatives, 1,4-naphthoquinones, and acridones using ethenesulfonyl fluoride as a bifunctional coupling partner. This protocol provides efficient access to diverse alkenyl sulfonyl fluoride derivatives with good functional-group compatibility. Notably, the sulfonyl fluoride functionality is retained throughout the transformation, enabling efficient post-synthetic diversification through SuFEx conjugation with a representative amino acid derivative and analgesic acetaminophen. Furthermore, all synthetic compounds have been screened for cytotoxic activity against human lung adenocarcinoma A549 cells. Moreover, compounds selected from the initial activity screening were further tested against various cancer cell lines, such as human breast adenocarcinoma cells (MCF-7), human liver carcinoma cells (HepG2), and human ovarian carcinoma cells (SKOV3). Taken together, the observed cytotoxic activity and conjugation capability highlight the potential utility of these compounds as versatile platforms for future bioconjugation and targeted therapeutic applications.

## 2. Results and Discussion

To identify the optimal reaction conditions, the coupling reaction of 4*H*-chromen-4-one (**1a**) with ethenesulfonyl fluoride (**2a**) was selected as a model transformation (Table 1). A broad range of reaction conditions was examined, and representative results from these studies are summarized in Table 1. We initially employed PivOH as the additive under conditions adapted from a previously reported Rh(III)-catalyzed C–H functionalization protocol [15]. Under these conditions, the reaction of **1a** with **2a** in the presence of [RhCp*Cl_2_]_2_ and AgSbF_6_ afforded the desired product **3a** in 17% yield (Table 1, entry 1). A similar result was obtained with AcOH, providing **3a** in 21% yield (Table 1, entry 2). Subsequent screening of additives identified AgOAc as the most effective additive (Table 1, entries 3–9). Interestingly, replacement of the Rh(III) catalyst with [Ru(*p*-cymene)Cl_2_]_2_ in the presence of Cu(OAc)_2_ afforded **3a** in 83% yield, indicating that the transformation could also be efficiently promoted by a Ru(II) catalyst (Table 1, entry 10). Further optimization of the Ru(II)-catalyzed conditions (Table 1, entries 11 and 12) revealed that the combination of [Ru(*p*-cymene)Cl_2_]_2_ (2.5 mol%), AgSbF_6_ (10 mol%), and Cu(OAc)_2_ (50 mol%) in DCE at 90 °C for 12 h under air afforded **3a** in 91% yield. Meanwhile, the reaction temperature was investigated for the Rh(III)-catalyzed system, and lowering the temperature from 120 to 60 °C did not diminish the reaction efficiency, affording **3a** in 98% yield (Table 1, entry 13). In contrast, only a trace amount of product was detected at room temperature (Table 1, entry 14). Finally, reducing the loading of AgOAc from 200 to 150 mol% maintained the excellent reaction efficiency, affording **3a** in 98% yield, whereas a further reduction to 100 mol% led to a decreased yield of 82% (Table 1, entries 15 and 16). Thus, the conditions shown in entry 15 were selected as the optimized Rh(III)-catalyzed conditions. Control experiments confirmed that both the Rh(III) catalyst and AgSbF_6_ were essential, as no reaction occurred in the absence of either component (Table 1, entries 17 and 18).

**Figure 2:**
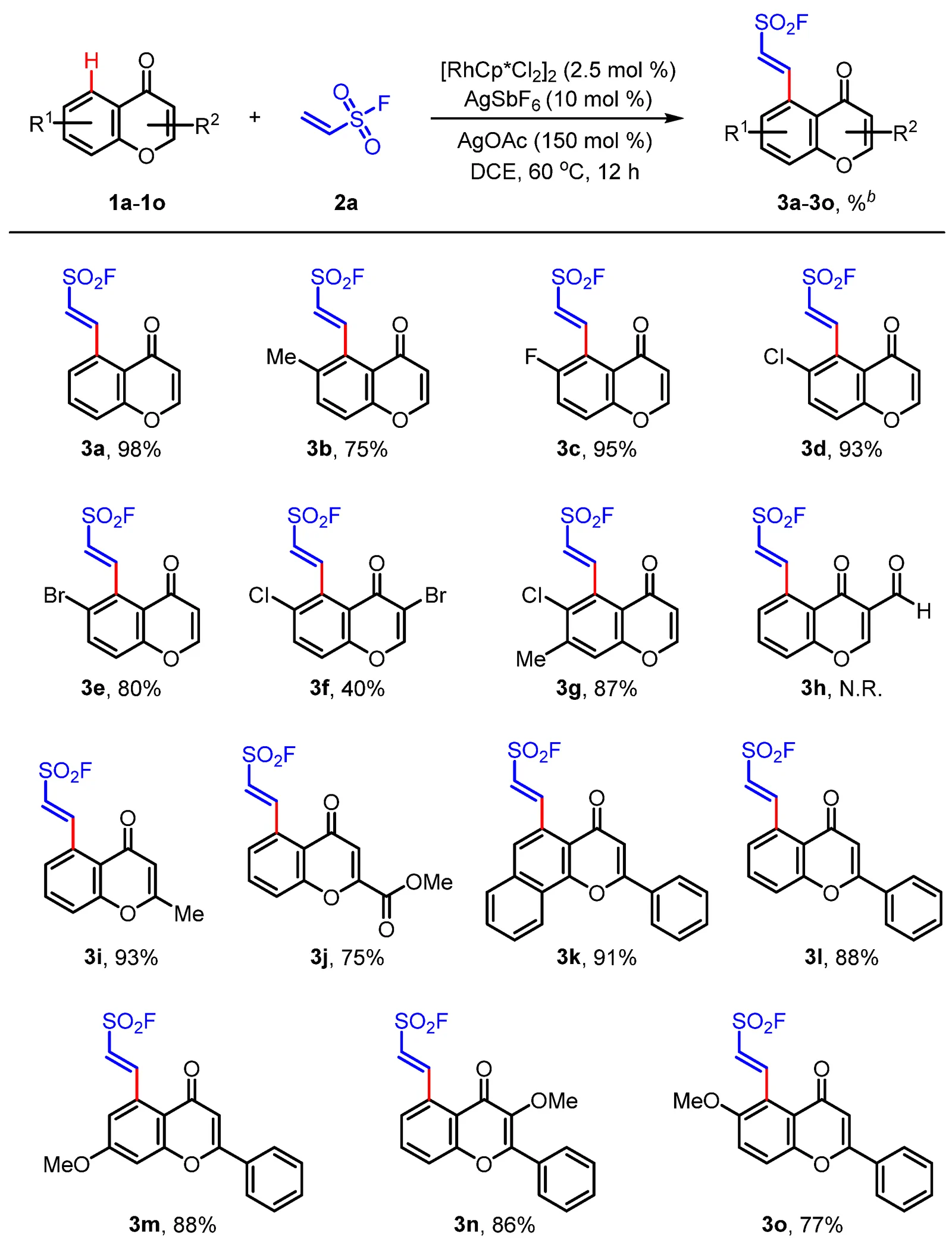
Scope of Chromone-based scaffolds with Ethenesulfonyl Fluoride *^a^*. *^a^* Reaction conditions: **1a**–**1o** (0.2 mmol), **2a** (0.24 mmol, 1.2 equiv), [RhCp*Cl_2_]_2_ (2.5 mol %), AgSbF_6_ (10 mol %), AgOAc (150 mol %), DCE (1 mL) under air at 60 °C for 12 h in pressure tubes. *^b^* Yield by flash column chromatography.

With the optimized reaction conditions in hand, the substrate scope of chromone-based scaffolds with ethenesulfonyl fluoride (ESF) was investigated, as summarized in Figure 2. Both electron-donating and electron-withdrawing substituents at the C6-position of the chromone ring were generally well tolerated, providing the desired products **3b**–**3e** in good to excellent yields (75–95%). Among the disubstituted chromones, 3-bromo-6-chlorochromone **1f** remained reactive, although the corresponding product **3f** was obtained in a diminished yield (40%). In contrast, 6-chloro-7-methylchromone **1g** afforded **3g** in 87% yield. Notably, however, the aldehyde-substituted substrate **1h** failed to undergo the transformation under the optimized reaction conditions. This lack of reactivity may be attributed to bidentate coordination of the aldehyde and ketone carbonyl groups to the metal center, potentially interfering with the catalytic cycle. Furthermore, substitution at the C2-position was also compatible with the present catalytic system. C2-substituted chromones bearing an electron-donating methyl group or an electron-withdrawing ester group afforded the corresponding products **3i** and **3j** in 93% and 75% yields, respectively. Encouraged by these results, the substrate scope was further extended to 2-aryl-substituted chromones, commonly known as flavones. Flavone derivatives were also suitable substrates, delivering the desired products **3k**–**3o** in consistently high yields (77–91%), thereby demonstrating the broad applicability of the protocol to structurally diverse chromone-based scaffolds.

**Figure 3:**
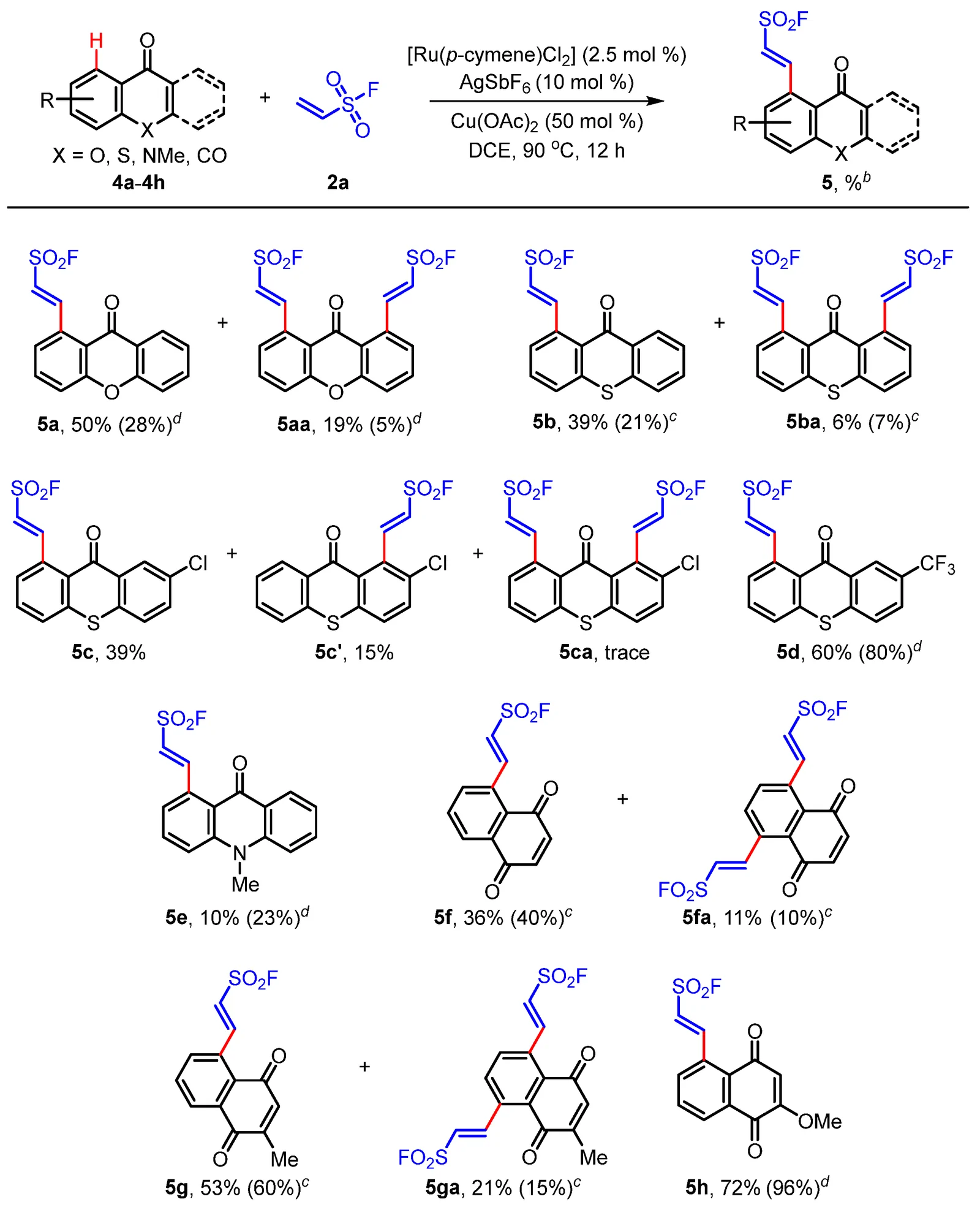
Scope of xanthone analogues with Ethenesulfonyl Fluoride *^a^*. *^a^* Reaction conditions: **4a**–**4h** (0.2 mmol), **2a** (0.6 mmol, 3 equiv), [Ru(*p*-cymene)Cl_2_]_2_ (2.5 mol %), AgSbF_6_ (10 mol %), Cu(OAc)_2_ (50 mol %) DCE (1 mL) under air at 90 °C for 12 h in pressure tubes. *^b^* Yield by flash column chromatography. *^c^* **2a** (1.2 mmol, 6 equiv) was used. *^d^* **2a** (2 mmol, 10 equiv) was used.

Next, we extended the substrate scope to xanthone analogues under the optimized Ru(II)-catalyzed conditions using ethenesulfonyl fluoride (ESF), as summarized in Figure 3. Xanthone (**4a**) underwent smooth olefination to afford the corresponding mono-olefinated product **5a** in 50% yield together with the bis-olefinated product **5aa** in 19% yield. In contrast, thioxanthone (**4b**) exhibited lower reactivity, affording the corresponding mono- and bis-olefinated products **5b** and **5ba** in 39% and 6% yields, respectively. Attempts to improve the formation of the bis-olefinated products by increasing the loading of ESF (10 equiv. for **4a** and 6 equiv. for **4b**) were unsuccessful, resulting in diminished overall reactivity. Chloro-substituted thioxanthone **4c** afforded a separable mixture of regioisomeric mono-olefinated products **5c** and **5c’** in 39% and 15% yields, respectively, while only a trace amount of the corresponding bis-olefinated product **5ca** was detected. Interestingly, the trifluoromethyl-substituted thioxanthone **4d** proved to be a suitable substrate, delivering **5d** in 60% yield, which was further improved to 80% by increasing the amount of ESF. Moreover, *N*-methylacridone (**4e**) was also compatible with the present catalytic system, albeit giving **5e** in a modest yield (10%), which was slightly improved to 23% by increasing the amount of ESF to 10 equivalents. Finally, the substrate scope was further expanded to 1,4-naphthoquinone derivatives. 1,4-Naphthoquinone (**4f**) and 2-methyl-1,4-naphthoquinone (**4g**) afforded the corresponding mono-olefinated products **5f** and **5g** in 36% and 53% yields, respectively, together with minor amounts of the bis-olefinated products **5fa** and **5ga**. In addition, 2-methoxy-1,4-naphthoquinone (**4h**) smoothly underwent the transformation to furnish **5h** in 72% yield, which was further improved to 96% by increasing the amount of ESF.

**Scheme 1:**
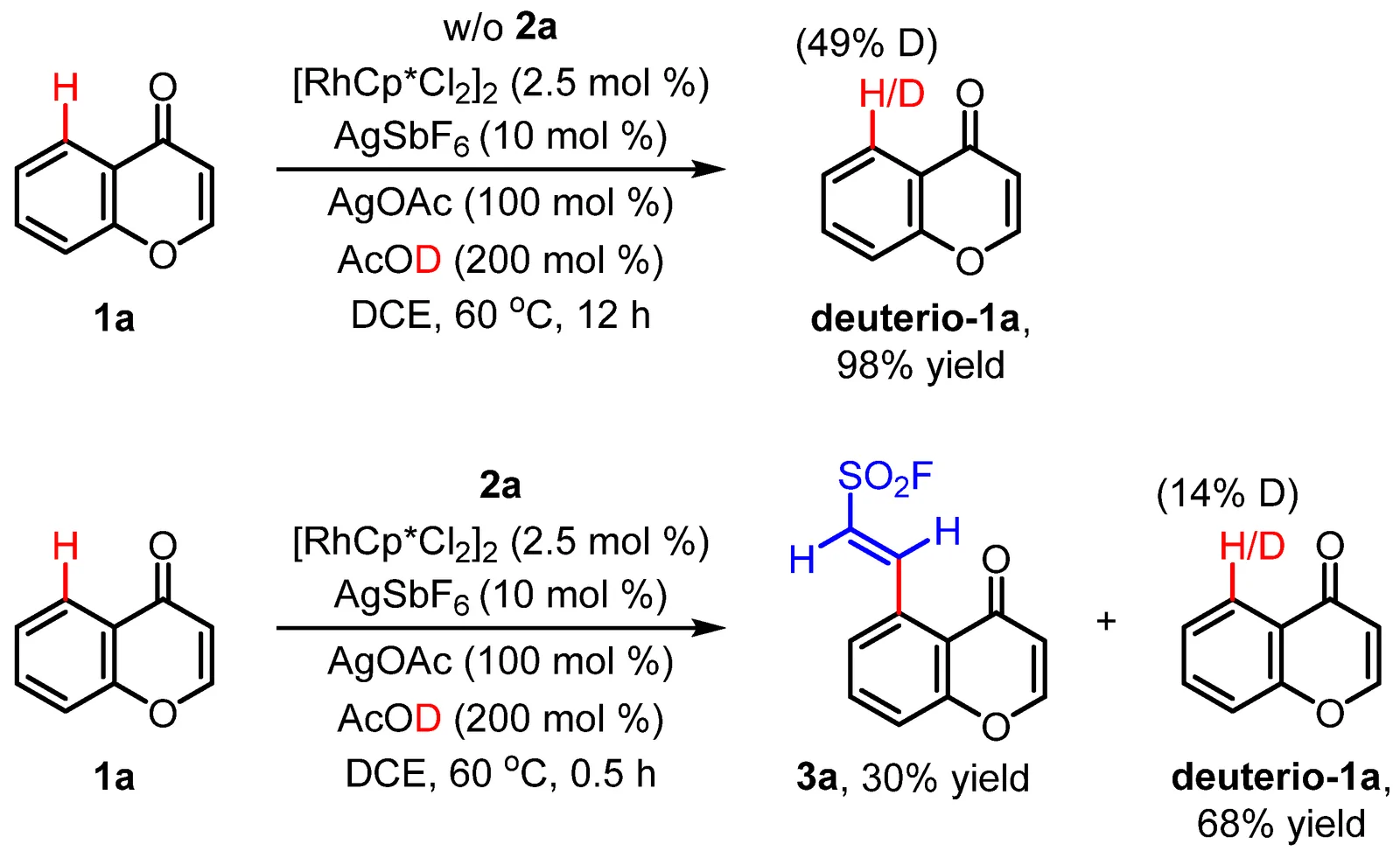
Deuterium-labeling experiment.

To gain mechanistic insight, the deuterium-labeling experiments were carried out using AcOD under the conditions shown in Scheme 1. In the absence of ESF, treatment of **1a** with AcOD resulted in 49% deuterium incorporation at the C5-position of the recovered chromone. When the reaction was performed in the presence of ESF, deuterium incorporation at the C5-position of the recovered starting material (14% D) was again observed, whereas no detectable deuterium incorporation was found in the olefinated product **3a**. These results suggest that the initial C–H activation step might be reversible. The absence of deuterium incorporation in the olefinated product is also consistent with the proposed catalytic pathway involving *β*-hydride elimination, as suggested by previous studies [27,28,29].

**Scheme 2:**
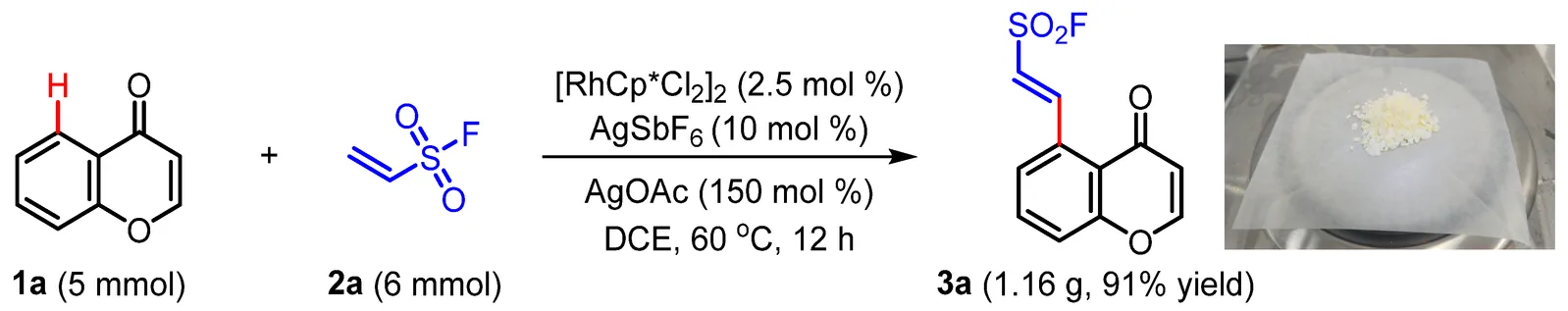
Scale-up experiment.

To further highlight the robustness and practicality of the developed protocol, the reaction was successfully performed on a gram scale under the optimized reaction conditions (Scheme 2), affording **3a** in 91% yield (1.16 g).

**Scheme 3:**
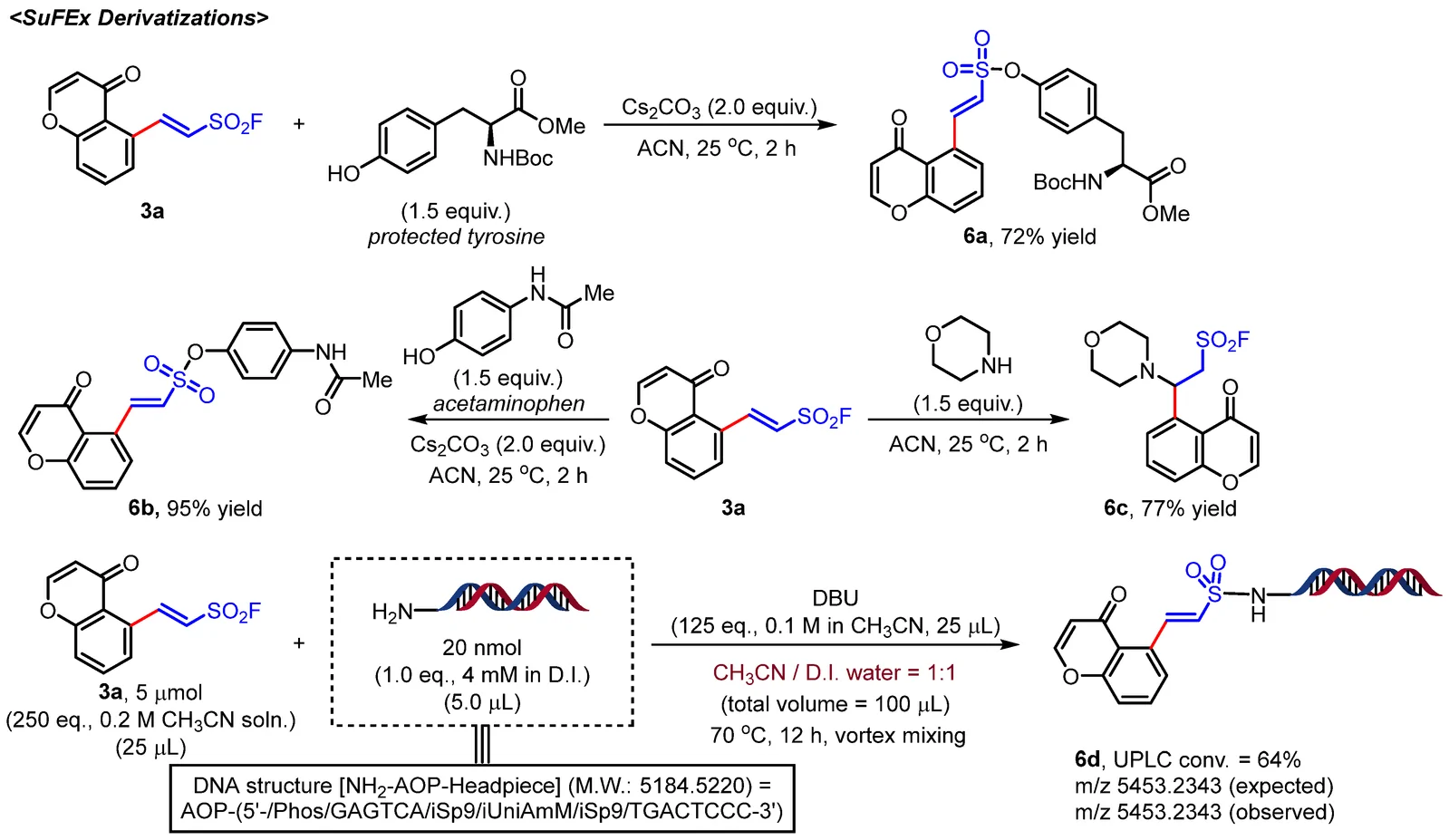
Synthetic Applications of the SuFEx-Active Product.

To further demonstrate the synthetic utility of the vinyl sulfonyl fluoride products, representative downstream transformations were investigated (Scheme 3). Initially, intermolecular SuFEx reactions of **3a** with the protected amino acid derivative of tyrosine and commercially available drug acetaminophen proceeded smoothly to afford the corresponding sulfonate derivatives **6a** and **6b** in 72% and 95% yields, respectively. Surprisingly, treatment of **3a** with morpholine did not undergo SuFEx substitution but instead furnished the corresponding Michael addition product **6c** in 77% yield, while retaining the sulfonyl fluoride functionality for further SuFEx derivatization. Finally, to explore the potential utility of these compounds as bioconjugation handles, preliminary DNA conjugation experiments were carried out using an amino-modified DNA headpiece (NH_2_-AOP-headpiece) under aqueous conditions (DMSO/D.I. water = 1:1, *v*/*v*) in the presence of DBU. To our delight, successful formation of the desired DNA-conjugated product **6d** was confirmed by UPLC-HRMS analysis, demonstrating the compatibility of the sulfonyl fluoride scaffold with DNA under the reaction conditions employed.

All the synthesized SO_2_F-containing chromones, xanthones, naphthoquinones, and acridones were initially screened for cytotoxic activity against human lung adenocarcinoma A549 cells, and the results are summarized in Table 2. The A549 cancer cells were exposed for 24 h to increasing concentrations of all compounds, and cell viability was determined using the WST-8 assay. In particular, compounds **3a**, **3b**, **3c**, **3d**, **3m**, and **5aa** exhibited EC_50_ values below 15 μM and were selected for further evaluation. Thus, those compounds were further tested against various cancer cell lines, such as human breast adenocarcinoma cells (MCF-7), human liver carcinoma cells (HepG2), and human ovarian carcinoma cells (SKOV3), as shown in Table 3. Compound **3c** displayed cytotoxicity in A549 and HepG2 cells, with EC_50_ values of 9.13 ± 0.36 and 7.26 ± 0.44 μM, respectively. In addition, compound **3d** also exhibited cytotoxic activity, with EC_50_ values of 11.79 ± 0.52, 11.55 ± 0.16, 7.0 ± 1.0, and 14.28 ± 0.39 μM in A549, MCF-7, HepG2, and SKOV3 cells, respectively. Notably, compound **5aa** showed the overall most favorable EC_50_ values among the six selected compounds across the additionally tested cell lines. These results reveal that SO_2_F-containing chromone and xanthone derivatives might have potential as anti-cancer agents.

## 3. Materials and Methods

### 3.1. General Information and Methods

All the experiments were carried out in a well-ventilated fume hood under ambient reaction conditions. Commercially available reagents were used without additional purification, unless otherwise stated. Ethenesulfonyl fluoride (**2a**) was purchased from TCI (Tokyo, Japan). Chromone, flavone, xanthone, thioxanthone, napthoquinone, and acridone were purchased from TCI. Sealed tubes (13 × 100 mm) were purchased from Fisher Scientific (Waltham, MA, USA), dried in an oven overnight, and cooled at room temperature before use. Thin-layer chromatography was carried out using plates coated with Kieselgel 60 F_254_ (Merck, Darmstadt, Germany). Nuclear magnetic resonance spectra (^1^H, ^13^C and ^19^F NMR) were recorded on a JEOL 400 (Akishima, Japan), Bruker 400 and 600 MHz NMR instrument (Billerica, MA, USA) in CDCl_3_ solution, and chemical shifts are reported as parts per million (ppm). Resonance patterns are reported with the notations s (singlet), d (doublet), t (triplet), q (quartet), m (multiplet) and br (broad). Coupling constants (*J*) are reported in hertz (Hz). IR spectra were recorded on a JASCO FT/IR4600 spectrophotometer (Hachioji, Japan) and are reported as cm^−1^. High-resolution mass spectra (HRMS) were recorded on a JEOL JMS-700 spectrometer (Akishima, Japan).

### 3.2. Experimental Procedures

#### 3.2.1. Typical Procedure for the Synthesis of SO_2_F-Containing Chromone-Based Scaffolds

To an oven-dried sealed tube charged with 4*H*-chromen-4-one (**1a**) (29.2 mg, 0.2 mmol, 100 mol %), [RhCp*Cl_2_]_2_ (3.1 mg, 0.005 mmol, 2.5 mol %), AgSbF_6_ (6.8 mg, 0.02 mmol, 10 mol %), AgOAc (50.0 mg, 0.3 mmol, 150 mol %), 1,2-dichloroethane (1 mL), was added ethenesulfonyl fluoride (**2a**) (26.4 mg, 0.24 mmol, 120 mol %). The reaction mixture was stirred for 12 h at 60 °C. After completion of the reaction, the reaction mass was cooled to room temperature. The crude reaction mass was purified by flash column chromatography (*n*-hexanes/EtOAc = 2:1) to afford **3a** (50.0 mg) in 98% yield.

#### 3.2.2. Typical Procedure for the Synthesis of SO_2_F-Containing Xanthone Analogues

To an oven-dried sealed tube charged with 9*H*-xanthen-9-one (**4a**) (39.2 mg, 0.2 mmol, 100 mol %), [Ru(*p*-cymene)Cl_2_]_2_ (3.1 mg, 0.005 mmol, 2.5 mol %), AgSbF_6_ (6.8 mg, 0.02 mmol, 10 mol %), Cu(OAc)_2_ (18.2 mg, 0.1 mmol, 50 mol %), 1,2-dichloroethane (1 mL), was added ethenesulfonyl fluoride (**2a**) (66.1 mg, 0.6 mmol, 300 mol %). The reaction mixture was stirred for 12 h at 90 °C. After completion of the reaction, the reaction mass was cooled to room temperature. The crude reaction mass was purified by flash column chromatography (*n*-hexanes/EtOAc = 5:1) to afford **5a** (30.5 mg) in 50% yield.

#### 3.2.3. Deuterium-Labeling Experiments

To an oven-dried sealed tube charged with 4*H*-chromen-4-one (**1a**) (29.2 mg, 0.2 mmol, 100 mol %), [RhCp*Cl_2_]_2_ (3.1 mg, 0.005 mmol, 2.5 mol %), AgSbF_6_ (6.8 mg, 0.02 mmol, 10 mol %), AgOAc (33.4 mg, 0.2 mmol, 100 mol %), 1,2-dichloroethane (1 mL), was added AcOD (24.4 mg, 0.4 mmol, 200 mol %). The reaction mixture was stirred for 12 h at 60 °C. After completion of the reaction, the reaction mass was cooled down to room temperature. The crude reaction mass was purified by flash column chromatography (*n*-hexanes/EtOAc = 2:1) to afford deuterio-**1a** (28.9 mg) in 98% yield. In case of the presence of ethenesulfonyl fluoride (**2a**) (66.1 mg, 0.6 mmol, 300 mol %). The reaction mixture was stirred for 0.5 h at 60 °C. After completion of the reaction, the reaction mass was cooled down to room temperature. The crude reaction mass was purified by flash column chromatography (*n*-hexanes/EtOAc = 2:1) to afford **3a** (15.3 mg) in 30% yield and deuterio-**1a** (20.0 mg) in 68% yield.

#### 3.2.4. Scale-Up Experiment

To an oven-dried sealed tube charged with 4*H*-chromen-4-one (**1a**) (730.7 mg, 5.0 mmol, 100 mol %), [RhCp*Cl_2_]_2_ (77.2 mg, 0.125 mmol, 2.5 mol %), AgSbF_6_ (171.8 mg, 0.5 mmol, 10 mol %), AgOAc (1251.8 mg, 7.5 mmol, 150 mol %), 1,2-dichloroethane (10.0 mL), was added ethenesulfonyl fluoride (**2a**) (660.6 mg, 6.0 mmol, 120 mol %). The reaction mixture was stirred for 12 h at 60 °C. After completion of the reaction, the reaction mass was cooled to room temperature. The crude reaction mass was purified by flash column chromatography (*n*-hexanes/EtOAc = 2:1) to afford **3a** (1.16 g) in 91% yield.

#### 3.2.5. Experimental Procedure for **6a**

To an oven-dried sealed tube was added (*E*)-2-(4-oxo-4*H*-chromen-5-yl)ethene-1-sulfonyl fluoride (**3a**) (25.4 mg, 0.1 mmol, 100 mol %), protected tyrosine (44.3 mg, 0.15 mmol, 150 mol %), and Cs_2_CO_3_ (65.1 mg, 0.2 mmol, 200 mol %), and acetonitrile (1 mL). The reaction mixture was stirred for 2 h at 25 °C. After completion of the reaction as indicated by TLC analysis, the crude reaction mass was purified by flash column chromatography (*n*-hexanes/EtOAc = 1:1) to afford **6a** (38.2 mg) in 72% yield.

#### 3.2.6. Experimental Procedure for **6b**

(*E*)-2-(4-Oxo-4*H*-chromen-5-yl)ethene-1-sulfonyl fluoride (**3a**) (25.4 mg, 0.1 mmol, 100 mol %), acetaminophen (22.6 mg, 0.15 mmol, 150 mol %), and Cs_2_CO_3_ (65.1 mg, 0.2 mmol, 200 mol %), acetonitrile (1 mL) were added to an oven-dried sealed tube. The reaction mixture was stirred for 2 h at 25 °C. After completion of the reaction as indicated by TLC analysis, the crude reaction mass was purified by flash column chromatography (*n*-hexanes/EtOAc = 1:4) to afford **6b** (36.7 mg) in 95% yield.

#### 3.2.7. Experimental Procedure for **6c**

To an oven-dried sealed tube charged with (*E*)-2-(4-oxo-4*H*-chromen-5-yl)ethene-1-sulfonyl fluoride (**3a**) (25.4 mg, 0.1 mmol, 100 mol %), morpholine (13.0 mg, 0.15 mmol, 150 mol %), acetonitrile (1 mL). The reaction mixture was allowed to stir for 2 h at 25 °C. After completion of the reaction as indicated by TLC analysis, the crude reaction mass was purified by flash column chromatography (*n*-hexanes/EtOAc = 1:1) to afford **6c** (26.3 mg) in 77% yield.

#### 3.2.8. Experimental Procedure for DNA SuFEx Conjugation

A solution of DNA-NH_2_ (20 nmol, 4 mM in deionized water, 5 μL) was added to an EP tube (Eppendorf Tube^®^, Eppendorf, Hamburg, Germany) containing water (45 μL). Then a solution of **3a** (0.2 M in CH_3_CN, 25 μL, 250 eq) and a solution of DBU (0.1 M in CH_3_CN, 25 μL, 125 eq) were added. The reaction tube was mixed by vortex at 70 °C. After 12 h, 10% by volume of 5 M NaCl solution was added, followed by cold ethanol (2.5 times by volume, ethanol stored at −20 °C). The reaction tube was allowed to stand for 10 min at −80 °C, then centrifuged at 15,000 RPM for 30 min. After discarding the supernatant, the precipitate was freeze-dried and analyzed with UPLC to check the desired DNA-conjugated product **6d**.

#### 3.2.9. Cell Lines and Culture Conditions

Human lung adenocarcinoma A549 cells, human hepatocellular carcinoma HepG2 cells, human breast adenocarcinoma MCF-7 cells, and human ovarian adenocarcinoma SK-OV-3 cells were obtained from the Korean Cell Line Bank (Seoul, Republic of Korea). A549 cells were maintained in RPMI 1640 medium supplemented with 25 mM HEPES and NaHCO_3_, HepG2 cells were maintained in minimum essential medium (MEM), and MCF-7 and SK-OV-3 cells were maintained in Dulbecco’s modified Eagle’s medium (DMEM). All culture media were supplemented with 10% fetal bovine serum and 1% penicillin–streptomycin. All cells were maintained at 37 °C in a humidified atmosphere containing 5% CO_2_.

#### 3.2.10. Cancer Cell Viability Assay (WST-8 Assay)

Cells were seeded in 96-well plates (SPL Life Sciences, Pocheon, Republic of Korea) at a density of 1.0 × 10^4^ cells/well in 100 μL of culture medium and incubated for 24 h. The culture medium was then removed, and the cells were exposed for 24 h to nine concentrations of each compound prepared by 2-fold serial dilution, with a maximum concentration of 50 μM. The corresponding concentration of DMSO was used for the vehicle-control group. Following compound exposure, the treatment medium was removed, and 100 μL of fresh culture medium containing 10 μL of Quanti-MAX WST-8 Cell Viability Assay Kit reagent (#QM5000, Biomax, Guri, Republic of Korea) and 90 μL of culture medium was added to each well. The cells were incubated at 37 °C for 1 h in a 5% CO_2_ incubator. Absorbance was measured at 440 nm with a reference wavelength of 690 nm using an Epoch microplate spectrophotometer (BioTek, Winooski, VT, USA). The WST-8 assay was performed in three independent experiments, and cell viability was calculated relative to the vehicle-control group in each experiment. EC_50_ values were calculated separately for each experiment by four-parameter logistic sigmoidal regression using SigmaPlot 12.0 and are presented as the mean ± standard deviation (SD). The EC_50_ was defined as the compound concentration required to reduce cell viability by 50%.

### 3.3. Characterization Data for All Synthesized Products

(*E*)-2-(4-Oxo-4*H*-chromen-5-yl)ethene-1-sulfonyl fluoride (**3a**) [22]: 50.0 mg (98%); Pale yellow solid; m.p. 146–148 °C; ^1^H NMR (600 MHz, CDCl_3_) δ 9.20 (d, *J* = 15.2 Hz, 1H), 7.85 (d, *J* = 5.8 Hz, 1H), 7.73–7.71 (m, 1H), 7.62 (dd, *J* = 8.5, 1.2 Hz, 1H), 7.44 (d, *J* = 7.5 Hz, 1H), 6.69 (dd, *J* = 15.4, 2.2 Hz, 1H), 6.36 (d, *J* = 5.9 Hz, 1H) ppm; ^13^C NMR (150 MHz, CDCl_3_) δ 178.25, 157.54, 154.86, 149.50 (d, *J* = 3.3 Hz), 133.52, 133.18, 125.41, 122.65, 121.96, 120.84 (d, *J* = 28.0 Hz), 114.35 ppm; ^19^F NMR (376 MHz, CDCl_3_) δ 61.68 ppm; FT-IR (CHCl_3_, film) υ 3075, 1643, 1614, 1472, 1391, 1345, 1226, 1186, 1107, 981, 868, 831, 747 cm^−1^; HRMS (FAB+) calcd for C_11_H_8_FO_4_S [M+H]^+^ 255.0122, found 255.0123.

(*E*)-2-(6-Methyl-4-oxo-4*H*-chromen-5-yl)ethene-1-sulfonyl fluoride (**3b**): 40.3 mg (75%); White solid; m.p. 174–176 °C; ^1^H NMR (600 MHz, CDCl_3_) δ 8.62 (d, *J* = 15.8 Hz, 1H), 7.82 (d, *J* = 5.9 Hz, 1H), 7.59 (d, *J* = 8.7 Hz, 1H), 7.46 (d, *J* = 8.7 Hz, 1H), 6.38 (dd, *J* = 15.8, 2.3 Hz, 1H), 6.32 (d, *J* = 5.9 Hz, 1H), 2.44 (s, 3H) ppm; ^13^C NMR (150 MHz, CDCl_3_) δ 178.02, 155.70, 154.81, 150.35 (d, *J* = 3.3 Hz), 136.46, 133.11, 131.21, 123.09, 121.89 (d, *J* = 26.9 Hz), 119.79, 113.94, 20.21 ppm; FT-IR (CHCl_3_, film) υ 3072, 2923, 1644, 1396, 1342, 1228, 1188, 895, 828 cm^−1^; HRMS (FAB+) calcd for C_12_H_10_FO_4_S [M+H]^+^ 269.0278 found 269.0280.

(*E*)-2-(6-Fluoro-4-oxo-4*H*-chromen-5-yl)ethene-1-sulfonyl fluoride (**3c**): 51.8 mg (95%); White solid; m.p. 166–168 °C; ^1^H NMR (600 MHz, CDCl_3_) δ 9.06 (d, *J* = 15.8 Hz, 1H), 7.86 (d, *J* = 5.8 Hz, 1H), 7.63 (dd, *J* = 9.3, 4.3 Hz, 1H), 7.53 (t, *J* = 9.5 Hz, 1H), 6.97 (dd, *J* = 16.0, 1.0 Hz, 1H), 6.35 (d, *J* = 5.9 Hz, 1H) ppm; ^13^C NMR (150 MHz, CDCl_3_) δ 178.10 (d, *J* = 3.3 Hz), 157.64 (d, *J* = 255.3 Hz), 154.81, 153.95, 140.83 (d, *J* = 3.3 Hz), 124.78 (dd, *J* = 28.0, 14.3 Hz), 123.45 (d, *J* = 9.3 Hz), 123.18 (d, *J* = 2.7 Hz), 122.45 (d, *J* = 27.4 Hz), 118.34 (d, *J* = 13.2 Hz), 114.04 ppm; FT-IR (CHCl_3_, film) υ 3086, 1637, 1461, 1402, 1360, 1294, 1189, 918, 839 cm^−1^; HRMS (FAB+) calcd for C_11_H_7_F_2_O_4_S [M+H]^+^ 273.0028, found 273.0031.

(*E*)-2-(6-Chloro-4-oxo-4*H*-chromen-5-yl)ethene-1-sulfonyl fluoride (**3d**): 53.7 mg (93%); White solid; m.p. 173–175 °C; ^1^H NMR (600 MHz, CDCl_3_) δ 8.46 (d, *J* = 15.8 Hz, 1H), 7.85 (d, *J* = 6.0 Hz, 1H), 7.77 (d, *J* = 8.9 Hz, 1H), 7.53 (d, *J* = 9.1 Hz, 1H), 6.68 (dd, *J* = 15.7, 2.2 Hz, 1H), 6.35 (d, *J* = 5.9 Hz, 1H) ppm; ^13^C NMR (150 MHz, CDCl_3_) δ 176.98, 155.79, 155.01, 146.14 (d, *J* = 3.3 Hz), 135.34, 130.42, 129.98, 124.28 (d, *J* = 7.7 Hz), 124.12, 121.57, 114.16 ppm; FT-IR (CHCl_3_, film) υ 3073, 2920, 2850, 1640, 1619, 1581, 1447, 1402, 1338, 1282, 1228, 1191, 994, 876, 802, 754 cm^−1^; HRMS (FAB+) calcd for C_11_H_7_ClFO_4_S [M+H]^+^ 288.9732, found 288.9735.

(*E*)-2-(6-Bromo-4-oxo-4*H*-chromen-5-yl)ethene-1-sulfonyl fluoride (**3e**): 53.4 mg (80%); White solid; m.p. 163–165 °C; ^1^H NMR (600 MHz, CDCl_3_) δ 8.39 (dd, *J* = 15.8, 6.0 Hz, 1H), 7.95 (d, *J* = 9.1 Hz, 1H), 7.85 (dd, *J* = 6.0, 2.5 Hz, 1H), 7.46 (d, *J* = 8.9 Hz, 1H), 6.60 (dt, *J* = 15.8, 2.5 Hz, 1H), 6.36 (dd, *J* = 6.0, 3.7 Hz, 1H) ppm; ^13^C NMR (150 MHz, CDCl_3_) δ 176.69, 156.26, 155.03, 148.32 (d, *J* = 3.3 Hz), 138.31, 132.63, 124.62, 124.05 (d, *J* = 27.5 Hz), 121.60, 119.27, 114.11 ppm; FT-IR (CHCl_3_, film) υ 3069, 3033, 2921, 2850, 1639, 1617, 1578, 1445, 1400, 1338, 1200, 991, 950, 871, 803, 744, 703 cm^−1^; HRMS (FAB+) calcd for C_11_H_7_BrFO_4_S [M+H]^+^ 332.9227, found 332.9230.

(*E*)-2-(3-Bromo-6-chloro-4-oxo-4*H*-chromen-5-yl)ethene-1-sulfonyl fluoride (**3f**): 29.5 mg (40%); White solid; m.p. 224–226 °C; ^1^H NMR (600 MHz, CDCl_3_) δ 8.43 (d, *J* = 15.8 Hz, 1H), 8.24 (s, 1H), 7.82 (d, *J* = 8.9 Hz, 1H), 7.56 (d, *J* = 9.1 Hz, 1H), 6.70 (dd, *J* = 15.7, 2.2 Hz, 1H) ppm; ^13^C NMR (150 MHz, CDCl_3_) δ 171.90, 155.44, 153.61, 145.44 (d, *J* = 3.3 Hz), 135.86, 130.80 (d, *J* = 4.4 Hz), 125.06, 124.88, 122.32, 121.45, 111.78 ppm; FT-IR (CHCl_3_, film) υ 3073, 2920, 2850, 1640, 1619, 1447, 1402, 1338, 1282, 1228, 1191, 1110, 994, 943, 876, 802, 754, 707 cm^−1^; HRMS (FAB+) calcd for C_11_H_6_BrClFO_4_S [M+H]^+^ 366.8837, found 368.8995.

(*E*)-2-(6-Chloro-7-methyl-4-oxo-4*H*-chromen-5-yl)ethene-1-sulfonyl fluoride (**3g**): 52.8 mg (87%); White solid; m.p. 161–163 °C; ^1^H NMR (600 MHz, CDCl_3_) δ 8.45 (d, *J* = 15.8 Hz, 1H), 7.80 (d, *J* = 5.9 Hz, 1H), 7.46 (s, 1H), 6.62 (dd, *J* = 15.7, 2.2 Hz, 1H), 6.31 (dd, *J* = 6.0, 2.1 Hz, 1H), 2.56 (s, 3H) ppm; ^13^C NMR (150 MHz, CDCl_3_) δ 176.84, 155.26, 154.77, 146.96 (d, *J* = 3.3 Hz), 144.22, 130.63 (d, *J* = 4.4 Hz), 124.17, 123.99, 122.29, 121.68, 114.00, 21.94 ppm; FT-IR (CHCl_3_, film) υ 3062, 2921, 1747, 1649, 1595, 1456, 1444, 1396, 1348, 1283, 1221, 1187, 1052, 944, 876, 818, 762, 699 cm^−1^; HRMS (FAB+) calcd for C_12_H_9_ClFO_4_S [M+H]^+^ 302.9889, found 302.9893.

(*E*)-2-(2-Methyl-4-oxo-4*H*-chromen-5-yl)ethene-1-sulfonyl fluoride (**3i**): 49.9 mg (93%); White solid; m.p. 144–146 °C; ^1^H NMR (600 MHz, CDCl_3_) δ 9.24 (d, *J* = 15.4 Hz, 1H), 7.69–7.66 (m, 1H), 7.57 (d, *J* = 8.5 Hz, 1H), 7.41 (d, *J* = 7.4 Hz, 1H), 6.68 (dd, *J* = 15.4, 2.2 Hz, 1H), 6.17–6.16 (m, 1H), 2.39 (s, 3H) ppm; ^13^C NMR (150 MHz, CDCl_3_) δ 178.81, 165.91, 157.48, 149.65 (d, *J* = 3.3 Hz), 133.15, 132.91, 125.04, 121.63, 121.38, 120.53 (d, *J* = 28.0 Hz), 111.93, 20.35 ppm; FT-IR (CHCl_3_, film) υ 3070, 2924, 2852, 1646, 1617, 1587, 1567, 1475, 1438, 1400, 1368, 1188, 1117, 1029, 958, 865, 840, 793, 752 cm^−1^; HRMS (FAB+) calcd for C_12_H_10_FO_4_S [M+H]^+^ 269.0278, found 269.0282.

Methyl (*E*)-5-(2-(fluorosulfonyl)vinyl)-4-oxo-4*H*-chromene-2-carboxylate (**3j**): 46.9 mg (75%); White solid; m.p. 183–185 °C; ^1^H NMR (600 MHz, CDCl_3_) δ 9.14 (d, *J* = 15.4 Hz, 1H), 7.80–7.76 (m, 2H), 7.49 (d, *J* = 5.7 Hz, 1H), 7.10 (s, 1H), 6.72 (dd, *J* = 15.4, 2.2 Hz, 1H), 4.03 (s, 3H) ppm; ^13^C NMR (150 MHz, CDCl_3_) δ 179.05, 160.62, 157.02, 151.59, 148.72 (d, *J* = 3.3 Hz), 134.47, 133.10, 125.99, 122.41, 122.13, 121.52 (d, *J* = 28.6 Hz), 116.05, 53.84 ppm; FT-IR (CHCl_3_, film) υ 3083, 2956, 2922, 2853, 1746, 1644, 1587, 1471, 1396, 1307, 1263, 1171, 1102, 969, 920, 888, 797, 747 cm^−1^; HRMS (FAB+) calcd for C_13_H_10_FO_6_S [M+H]^+^ 313.0177, found 313.0181.

(*E*)-2-(4-Oxo-2-phenyl-4*H*-benzo[*h*]chromen-5-yl)ethene-1-sulfonyl fluoride (**3k**): 69.3 mg (91%); Pale yellow solid; m.p. 255–257 °C; ^1^H NMR (600 MHz, CDCl_3_) δ 9.36 (d, *J* = 15.2 Hz, 1H), 8.67–8.65 (m, 1H), 8.05–8.03 (m, 2H), 8.02–7.99 (m, 1H), 7.86 (s, 1H), 7.85–7.81 (m, 2H), 7.63–7.61 (m, 3H), 6.96 (s, 1H), 6.80 (dd, *J* = 15.3, 2.2 Hz, 1H) ppm; ^13^C NMR (150 MHz, CDCl_3_) δ 178.88, 162.43, 150.41, 134.58, 132.11, 131.41, 130.65, 129.50, 129.20, 128.96, 128.69, 126.47, 126.45, 125.98, 125.59, 122.93, 119.63, 119.45, 118.15, 109.98 ppm; FT-IR (CHCl_3_, film) υ 3074, 2914, 2845, 1637, 1618, 1452, 1399, 1364, 1186, 1105, 954, 867, 772, 749 cm^−1^; HRMS (FAB+) calcd for C_21_H_14_FO_4_S [M+H]^+^ 381.0591, found 381.0593.

(*E*)-2-(4-Oxo-2-phenyl-4*H*-chromen-5-yl)ethene-1-sulfonyl fluoride (**3l**): 58.2 mg (88%); White solid; m.p. 213–215 °C; ^1^H NMR (600 MHz, CDCl_3_) δ 9.30 (d, *J* = 15.4 Hz, 1H), 7.92 (d, *J* = 8.3 Hz, 2H), 7.74 (d, *J* = 3.2 Hz, 2H), 7.59–7.53 (m, 3H), 7.46 (dd, *J* = 5.5, 3.0 Hz, 1H), 6.82 (s, 1H), 6.72 (d, *J* = 15.4 Hz, 1H) ppm; ^13^C NMR (150 MHz, CDCl_3_) δ 179.02, 163.06, 157.28, 149.46 (d, *J* = 2.7 Hz), 133.44, 133.05, 132.19, 131.06, 129.32, 126.47, 125.28, 121.82 (d, *J* = 6.6 Hz), 120.90, 120.71, 108.78 ppm; FT-IR (CHCl_3_, film) υ 3076, 3004, 2921, 2851, 1626, 1591, 1567, 1476, 1404, 1377, 1194, 1119, 961, 902, 866, 853, 747 cm^−1^; HRMS (FAB+) calcd for C_17_H_12_FO_4_S [M+H]^+^ 331.0435, found 331.0443.

(*E*)-2-(7-Methoxy-4-oxo-2-phenyl-4*H*-chromen-5-yl)ethene-1-sulfonyl fluoride (**3m**): 63.5 mg (88%); White solid; m.p. 213–216 °C; ^1^H NMR (400 MHz, CDCl_3_) δ 9.25 (d, *J* = 15.4 Hz, 1H), 7.89 (dd, *J* = 8.1, 1.7 Hz, 2H), 7.58–7.50 (m, 3H), 7.10 (d, *J* = 2.4 Hz, 1H), 6.98 (dd, *J* = 2.5, 0.8 Hz, 1H), 6.72 (s, 1H), 6.68 (dd, *J* = 15.5, 2.2 Hz, 1H), 3.97 (s, 3H) ppm; ^13^C NMR (100 MHz, CDCl_3_) δ 178.43, 163.14, 162.58, 159.10, 149.41 (d, *J* = 2.9 Hz), 134.30, 131.97, 131.16, 129.27, 126.32, 120.88 (d, *J* = 27.8 Hz), 115.72, 114.64, 108.58, 103.40, 56.33 ppm; FT-IR (CHCl_3_, film) υ 3072, 1637, 1593, 1398, 1353, 1220, 1187, 1160, 1106, 959, 837, 774 cm^−1^; HRMS (FAB+) calcd for C_18_H_14_FO_5_S [M+H]^+^ 361.0540, found 361.0546.

(*E*)-2-(3-Methoxy-4-oxo-2-phenyl-4*H*-chromen-5-yl)ethene-1-sulfonyl fluoride (**3n**): 62.0 mg (86%); White solid; m.p. 213–215 °C; ^1^H NMR (600 MHz, CDCl_3_) δ 9.32 (d, *J* = 15.4 Hz, 1H), 8.13–8.10 (m, 2H), 7.74–7.69 (m, 2H), 7.55–7.53 (m, 3H), 7.45 (dd, *J* = 6.8, 2.3 Hz, 1H), 6.74 (dd, *J* = 15.4, 2.2 Hz, 1H), 3.90 (s, 3H) ppm; ^13^C NMR (150 MHz, CDCl_3_) δ 175.91, 156.26, 155.28, 149.43 (d, *J* = 3.3 Hz), 142.41, 133.26, 133.13, 131.30, 130.35, 128.86, 128.61, 124.88, 121.90, 121.81, 120.84 (d, *J* = 28.0 Hz), 60.37 ppm; FT-IR (CHCl_3_, film) υ 3081, 3060, 2953, 2921, 2852, 1736, 1630, 1612, 1591, 1446, 1402, 1379, 1232, 1187, 1080, 929, 863 cm^−1^; HRMS (FAB+) calcd for C_18_H_14_FO_5_S [M+H]^+^ 361.0540, found 361.0542.

(*E*)-2-(6-Methoxy-4-oxo-2-phenyl-4*H*-chromen-5-yl)ethene-1-sulfonyl fluoride (**3o**): 55.5 mg (77%); White solid; m.p. 250–252 °C; ^1^H NMR (600 MHz, CDCl_3_) δ 9.51 (d, *J* = 15.7 Hz, 1H), 7.91 (d, *J* = 7.2 Hz, 2H), 7.72 (d, *J* = 9.2 Hz, 1H), 7.57–7.52 (m, 3H), 7.42 (d, *J* = 9.4 Hz, 1H), 7.30 (d, *J* = 15.8 Hz, 1H), 6.79 (s, 1H), 4.03 (s, 3H) ppm; ^13^C NMR (150 MHz, CDCl_3_) δ 179.58, 162.28, 156.42, 151.82, 142.52, 132.01, 131.15, 129.28, 126.39, 123.56 (d, *J* = 26.4 Hz), 122.78, 122.53, 118.11, 117.63, 108.67, 56.79 ppm; FT-IR (CHCl_3_, film) υ 3098, 3055, 2921, 2852, 1632, 1570, 1468, 1451, 1386, 1370, 1290, 1202, 1183, 1135, 1059, 997, 912, 872 cm^−1^; HRMS (FAB+) calcd for C_18_H_14_FO_5_S [M+H]^+^ 361.0540, found 361.0542.

(*E*)-2-(9-Oxo-9*H*-xanthen-1-yl)ethene-1-sulfonyl fluoride (**5a**): 30.5 mg (50%); White solid; m.p. 205–207 °C; ^1^H NMR (600 MHz, CDCl_3_) δ 9.25 (d, *J* = 15.3 Hz, 1H), 8.31 (dd, *J* = 7.9, 1.7 Hz, 1H), 7.77 (t, *J* = 8.1 Hz, 2H), 7.67 (dd, *J* = 8.5, 1.3 Hz, 1H), 7.51 (d, *J* = 8.5 Hz, 1H), 7.44–7.39 (m, 2H), 6.73 (dd, *J* = 15.4, 2.3 Hz, 1H) ppm; ^13^C NMR (150 MHz, CDCl_3_) δ 177.92, 157.19, 155.54, 150.12 (d, *J* = 3.3 Hz), 135.56, 134.45, 134.00, 126.96, 124.76, 124.29, 122.33, 121.79, 120.76 (d, *J* = 28.0 Hz), 119.60, 117.88 ppm; FT-IR (CHCl_3_, film) υ 3078, 2920, 1644, 1614, 1585, 1565, 1468, 1394, 1352, 1236, 1210, 1186, 1143, 869, 855, 787, 742 cm^−1^; HRMS (FAB+) calcd for C_15_H_10_FO_4_S [M+H]^+^ 305.0278, found 305.0281.

(1*E*,1′*E*)-2,2′-(9-Oxo-9*H*-xanthene-1,8-diyl)bis(ethene-1-sulfonyl fluoride) (**5aa**): 15.7 mg (19%); White solid; m.p. 265–267 °C; ^1^H NMR (600 MHz, CDCl_3_) δ 9.09 (d, *J* = 15.3 Hz, 2H), 7.82–7.79 (m, 2H), 7.68 (d, *J* = 8.5 Hz, 2H), 7.44 (d, *J* = 7.5 Hz, 2H), 6.73 (dd, *J* = 15.4, 2.2 Hz, 2H) ppm; ^13^C NMR (100 MHz, CDCl_3_) δ 178.49, 156.48, 149.46, 135.00, 134.25, 125.14, 121.65, 121.44, 120.09 ppm; FT-IR (CHCl_3_, film) υ 3075, 2922, 2850, 1637, 1614, 1584, 1562, 1474, 1397, 1356, 1211, 1186, 1155, 1003, 956, 917, 863, 793, 758 cm^−1^; HRMS (FAB+) calcd for C_17_H_11_F_2_O_6_S_2_ [M+H]^+^ 412.9960, found 412.9967.

(*E*)-2-(9-Oxo-9*H*-thioxanthen-1-yl)ethene-1-sulfonyl fluoride (**5b**): 25.0 mg (39%); Orange solid; m.p. 165–167 °C; ^1^H NMR (600 MHz, CDCl_3_) δ 8.88 (d, *J* = 15.2 Hz, 1H), 8.55 (dd, *J* = 8.1, 1.6 Hz, 1H), 7.73 (d, *J* = 8.2 Hz, 1H), 7.68–7.65 (m, 2H), 7.59 (d, *J* = 8.0 Hz, 1H), 7.54–7.51 (m, 1H), 7.40 (d, *J* = 7.4 Hz, 1H), 6.59 (dd, *J* = 15.3, 2.3 Hz, 1H) ppm; ^13^C NMR (150 MHz, CDCl_3_) δ 181.06, 152.82 (d, *J* = 2.8 Hz), 139.64, 136.74, 136.20, 132.94, 131.91, 130.13, 130.05, 129.12, 127.75, 127.63, 127.09, 125.73, 118.53 (d, *J* = 27.5 Hz) ppm; FT-IR (CHCl_3_, film) υ 3076, 3058, 2955, 2919, 2851, 1686, 1623, 1445, 1393, 1317, 1214, 1193, 1159, 1079, 954, 937, 858, 752 cm^−1^; HRMS (FAB−) calcd for C_15_H_9_FO_3_S_2_ [M]^+^ 319.9977, found 319.9980.

(1*E*,1′*E*)-2,2′-(9-Oxo-9*H*-thioxanthene-1,8-diyl)bis(ethene-1-sulfonyl fluoride) (**5ba**): 5.2 mg (6%); Orange solid; m.p. 184–186 °C; ^1^H NMR (600 MHz, CDCl_3_) δ 8.72 (d, *J* = 14.8 Hz, 2H), 7.73 (d, *J* = 8.1 Hz, 2H), 7.70 (t, *J* = 7.6 Hz, 2H), 7.46 (d, *J* = 7.2 Hz, 2H), 6.63 (dd, *J* = 15.2, 2.1 Hz, 2H) ppm; ^13^C NMR (150 MHz, CDCl_3_) δ 182.26, 151.29 (d, *J* = 3.3 Hz), 138.44, 136.53, 132.46, 128.67, 128.64, 128.15, 119.78 (d, *J* = 28.0 Hz) ppm; FT-IR (CHCl_3_, film) υ 3070, 2954, 2920, 2851, 1695, 1609, 1574, 1448, 1397, 1189, 961, 861, 776 cm^−1^; HRMS (FAB+) calcd for C_17_H_11_F_2_O_5_S_3_ [M+H]^+^ 428.9731, found 428.9736.

(*E*)-2-(7-Chloro-9-oxo-9*H*-thioxanthen-1-yl)ethene-1-sulfonyl fluoride (**5c**): 27.7 mg (39%); Pale yellow solid; m.p. 197–200 °C; ^1^H NMR (600 MHz, CDCl_3_) δ 8.86 (d, *J* = 15.2 Hz, 1H), 8.55 (d, *J* = 2.3 Hz, 1H), 7.73 (d, *J* = 8.2 Hz, 1H), 7.70–7.67 (m, 1H), 7.63 (dd, *J* = 8.6, 2.4 Hz, 1H), 7.55 (d, *J* = 8.5 Hz, 1H), 7.43 (d, *J* = 7.3 Hz, 1H), 6.60 (dd, *J* = 15.2, 2.3 Hz, 1H) ppm; ^13^C NMR (150 MHz, CDCl_3_) δ 180.07, 148.84 (d, *J* = 3.3 Hz), 137.86, 135.94, 134.19, 133.63, 133.56, 133.16, 131.61, 130.39, 129.75, 128.69, 127.31, 125.76, 121.43 (d, *J* = 27.5 Hz) ppm; FT-IR (CHCl_3_, film) υ 3076, 2957, 2920, 2851, 1714, 1619, 1578, 1446, 1396, 1315, 1274, 1189, 1096, 950, 860, 760 cm^−1^; HRMS (FAB+) calcd for C_15_H_9_ClFO_3_S_2_ [M+H]^+^ 354.9660, found 354.9661.

(*E*)-2-(2-Chloro-9-oxo-9*H*-thioxanthen-1-yl)ethene-1-sulfonyl fluoride (**5c’**): 10.7 mg (15%); Pale yellow solid; m.p. 161–163 °C; ^1^H NMR (600 MHz, CDCl_3_) δ 8.53 (dd, *J* = 8.1, 1.7 Hz, 1H), 8.43 (d, *J* = 15.7 Hz, 1H), 7.72 (d, *J* = 8.6 Hz, 1H), 7.68–7.65 (m, 1H), 7.61 (d, *J* = 8.8 Hz, 1H), 7.58 (d, *J* = 8.1 Hz, 1H), 7.53–7.51 (m, 1H), 6.50 (dd, *J* = 15.7, 2.3 Hz, 1H) ppm; ^13^C NMR (150 MHz, CDCl_3_) δ 180.05, 152.42 (d, *J* = 3.3 Hz), 139.35, 136.93, 134.40, 133.58, 133.29, 132.26, 131.08, 129.72, 129.17, 127.90, 127.28, 127.22, 118.99 (d, *J* = 27.5 Hz) ppm; FT-IR (CHCl_3_, film) υ 2953, 2920, 2852, 1708, 1629, 1590, 1460, 1376, 1235, 1164, 1081, 816, 754 cm^−1^; HRMS (FAB+) calcd for C_15_H_9_ClFO_3_S_2_ [M+H]^+^ 354.9660, found 354.9669.

(*E*)-2-(9-Oxo-7-(trifluoromethyl)-9*H*-thioxanthen-1-yl)ethene-1-sulfonyl fluoride (**5d**): 46.7 mg (60%); Red solid; m.p. 159–161 °C; ^1^H NMR (600 MHz, CDCl_3_) δ 8.86 (d, *J* = 17.9 Hz, 1H), 7.86 (dd, *J* = 8.4, 2.1 Hz, 1H), 7.76 (dd, *J* = 8.2, 1.5 Hz, 1H), 7.73–7.71 (m, 3H), 7.46 (dt, *J* = 7.2, 1.2 Hz, 1H), 6.62 (dd, *J* = 15.2, 2.3 Hz, 1H) ppm; ^13^C NMR (150 MHz, CDCl_3_) δ 180.09, 151.98 (d, *J* = 3.3 Hz), 139.90, 138.83, 136.84, 132.48, 132.16, 129.86, 129.28, 129.06, 128.74 (q, *J* = 3.3 Hz), 128.14, 127.47 (q, *J* = 4.4 Hz), 127.24, 126.57, 119.19 (d, *J* = 27.5 Hz) ppm; FT-IR (CHCl_3_, film) υ 3084, 2919, 1729, 1706, 1672, 1612, 1526, 1494, 1458, 1422, 1348, 1232, 1189, 916, 790, 744 cm^−1^; HRMS (FAB+) calcd for C_16_H_9_F_4_O_3_S_2_ [M+H]^+^ 388.9924, found 388.9924.

(*E*)-2-(10-Methyl-9-oxo-9,10-dihydroacridin-1-yl)ethene-1-sulfonyl fluoride (**5e**): 6.4 mg (10%); Yellow solid; m.p. 240–242 °C; ^1^H NMR (600 MHz, CDCl_3_) δ 9.20 (d, *J* = 15.3 Hz, 1H), 8.51 (dd, *J* = 8.0, 1.7 Hz, 1H), 7.79–7.74 (m, 2H), 7.70 (d, *J* = 8.8 Hz, 1H), 7.56 (d, *J* = 8.5 Hz, 1H), 7.36–7.33 (m, 1H), 7.22 (d, *J* = 7.0 Hz, 1H), 6.60 (dd, *J* = 15.3, 2.2 Hz, 1H), 3.95 (s, 3H) ppm; ^13^C NMR (150 MHz, CDCl_3_) δ 178.83, 153.14 (d, *J* = 3.0 Hz), 143.82, 142.31, 135.22, 134.48, 133.29, 127.97, 123.50, 122.33, 122.10, 120.36, 118.45 (d, *J* = 26.9 Hz), 118.13, 114.99, 34.70 ppm; FT-IR (CHCl_3_, film) υ 3079, 2955, 2919, 2851, 1737, 1595, 1497, 1462, 1396, 1373, 1278, 1188, 1171, 1071, 868, 781, 747 cm^−1^; HRMS (FAB+) calcd for C_16_H_13_FNO_3_S [M+H]^+^ 318.0595, found 318.0600.

(*E*)-2-(5,8-Dioxo-5,8-dihydronaphthalen-1-yl)ethene-1-sulfonyl fluoride (**5f**): 19.2 mg (36%); Brown solid; m.p. 192–194 °C; ^1^H NMR (600 MHz, CDCl_3_) δ 8.81 (d, *J* = 15.4 Hz, 1H), 8.30 (dd, *J* = 7.7, 1.4 Hz, 1H), 7.86 (t, *J* = 7.7 Hz, 1H), 7.76 (d, *J* = 8.5 Hz, 1H), 7.05–7.01 (m, 2H), 6.74 (dd, *J* = 15.4, 2.3 Hz, 1H) ppm; ^13^C NMR (150 MHz, CDCl_3_) δ 185.97, 149.02, 139.69, 137.93, 134.24, 134.10, 133.38, 129.91, 122.36, 122.17 ppm; FT-IR (CHCl_3_, film) υ 3071, 2952, 2917, 2849, 1708, 1654, 1608, 1398, 1328, 1293, 1215, 1185, 1086, 1037, 962, 860, 762 cm^−1^; HRMS (FAB−) calcd for C_12_H_7_FO_4_S [M]^+^ 266.0049, found 266.0042.

(1*E*,1′*E*)-2,2′-(5,8-Dioxo-5,8-dihydronaphthalene-1,4-diyl)bis(ethene-1-sulfonyl fluoride) (**5fa**): 8.3 mg (11%); Brown solid; m.p. 178–180 °C; ^1^H NMR (600 MHz, CDCl_3_) δ 8.69 (d, *J* = 15.4 Hz, 2H), 7.81 (s, 2H), 7.07 (s, 2H), 6.75 (dd, *J* = 15.4, 2.2 Hz, 2H) ppm; ^13^C NMR (100 MHz, CDCl_3_) δ 185.17, 148.33, 138.73, 136.15, 134.33, 131.18, 122.98 (d, *J* = 29.2 Hz) ppm; FT-IR (CHCl_3_, film) υ 3073, 2954, 2920, 2851, 1713, 1661, 1614, 1461, 1390, 1329, 1278, 1219, 1186, 1094, 952, 856, 840, 769, 744 cm^−1^; HRMS (FAB−) calcd for C_14_H_8_F_2_O_6_S_2_ [M]^+^ 373.9730, found 373.9729.

(*E*)-2-(6-Methyl-5,8-dioxo-5,8-dihydronaphthalen-1-yl)ethene-1-sulfonyl fluoride (**5g**) [27]: 29.8 mg (53%); Brown solid; m.p. 193–195 °C; ^1^H NMR (600 MHz, CDCl_3_) δ 8.84 (d, *J* = 15.2 Hz, 1H), 8.31 (dd, *J* = 7.8, 1.5 Hz, 1H), 7.81 (t, *J* = 7.8 Hz, 1H), 7.73 (d, *J* = 7.6 Hz, 1H), 6.87 (q, *J* = 1.6 Hz, 1H), 6.73 (dd, *J* = 15.4, 2.3 Hz, 1H), 2.22 (d, *J* = 1.6 Hz, 3H) ppm; ^13^C NMR (150 MHz, CDCl_3_) δ 185.85, 184.73, 149.23 (d, *J* = 2.7 Hz), 147.73, 136.67, 135.09, 133.86, 133.78, 133.66, 132.89, 130.05, 121.93 (d, *J* = 28.0 Hz), 16.31 ppm; FT-IR (CHCl_3_, film) υ 3073, 2916, 2848, 1651, 1617, 1402, 1321, 1279, 1219, 1193, 1115, 995, 864, 756 cm^−1^; HRMS (FAB+) calcd for C_13_H_10_FO_4_S [M+H]^+^ 281.0278, found 281.0280.

(1*E*,1′*E*)-2,2′-(6-Methyl-5,8-dioxo-5,8-dihydronaphthalene-1,4-diyl)bis(ethene-1-sulfonyl fluoride) (**5ga**) [27]: 16.4 mg (21%); Brown solid; m.p. 169–170 °C; ^1^H NMR (600 MHz, CDCl_3_) δ 8.72 (d, *J* = 14.5 Hz, 1H), 8.67 (d, *J* = 15.2 Hz, 1H), 7.79–7.76 (m, 2H), 6.93 (q, *J* = 1.6 Hz, 1H), 6.74 (dd, *J* = 2.4, 1.3 Hz, 1H), 6.72 (dd, *J* = 2.3, 1.3 Hz, 1H), 2.23 (d, *J* = 1.5 Hz, 3H) ppm; ^13^C NMR (150 MHz, CDCl_3_) δ 185.89, 185.01, 148.90 (d, *J* = 3.3 Hz), 148.67, 148.56 (d, *J* = 3.3 Hz), 136.15, 135.82, 134.07 (d, *J* = 4.4 Hz), 131.56, 131.42, 122.68, 122.51 (d, *J* = 8.8 Hz), 122.35, 16.54 ppm; FT-IR (CHCl_3_, film) υ 3073, 2954, 2920, 2851, 1713, 1654, 1613, 1462, 1400, 1321, 1219, 1186, 1145, 1022, 958, 863, 842, 773, 749 cm^−1^; HRMS (FAB−) calcd for C_15_H_10_F_2_O_6_S_2_ [M]^+^ 387.9887, found 387.9889.

(*E*)-2-(6-Methoxy-5,8-dioxo-5,8-dihydronaphthalen-1-yl)ethene-1-sulfonyl fluoride (**5h**): 42.7 mg (72%); Brown solid; m.p. 221–223 °C; ^1^H NMR (600 MHz, CDCl_3_) δ 8.86 (d, *J* = 15.5 Hz, 1H), 8.34 (dd, *J* = 7.8, 1.5 Hz, 1H), 7.80 (t, *J* = 7.7 Hz, 1H), 7.73 (d, *J* = 7.8 Hz, 1H), 6.70 (dd, *J* = 15.4, 2.2 Hz, 1H), 6.20 (s, 1H), 3.93 (s, 3H) ppm; ^13^C NMR (150 MHz, CDCl_3_) δ 185.68, 179.39, 159.90, 149.61 (d, *J* = 3.3 Hz), 134.59, 133.55, 133.08, 132.53, 130.08, 129.77, 121.70 (d, *J* = 28.0 Hz), 111.03, 56.80 ppm; FT-IR (CHCl_3_, film) υ 3079, 3056, 2952, 2921, 2852, 1730, 1684, 1643, 1614, 1459, 1402, 1374, 1327, 1240, 1191, 1120, 1063, 965, 863, 812, 754 cm^−1^; HRMS (FAB+) calcd for C_13_H_10_FO_5_S [M+H]^+^ 297.0227, found 297.0229.

Methyl (*S*,*E*)-2-((*tert*-butoxycarbonyl)amino)-3-(4-(((2-(4-oxo-4*H*-chromen-5-yl)vinyl)sulfonyl)oxy)phenyl)propanoate (**6a**): 38.2 mg (72%); White solid; m.p. 83–84 °C; ^1^H NMR (600 MHz, CDCl_3_) δ 8.81 (d, *J* = 15.4 Hz, 1H), 7.82 (d, *J* = 5.8 Hz, 1H), 7.68 (t, *J* = 8.0 Hz, 1H), 7.56 (d, *J* = 8.5 Hz, 1H), 7.37 (d, *J* = 7.5 Hz, 1H), 7.27 (d, *J* = 8.6 Hz, 2H), 7.14 (d, *J* = 8.5 Hz, 2H), 6.65 (d, *J* = 15.4 Hz, 1H), 6.35 (d, *J* = 6.0 Hz, 1H), 5.28 (d, *J* = 8.3 Hz, 1H), 4.57 (q, *J* = 6.7, 6.1 Hz, 1H), 3.68 (s, 3H), 3.09 (qd, *J* = 13.1, 5.1 Hz, 2H) 1.39 (s, 9H) ppm; ^13^C NMR (150 MHz, CDCl_3_) δ 178.12, 172.13, 157.45, 155.23, 154.57, 148.76, 147.16, 135.34, 134.01, 133.37, 130.79, 125.32, 123.50, 122.53, 122.40, 121.17, 114.20, 80.02, 54.38, 52.33, 37.67, 28.36 ppm; FT-IR (CHCl_3_, film) υ 3351, 3074, 2974, 2952, 2923, 2852, 1734, 1704, 1643, 1592, 1503, 1474, 1363, 1347, 1283, 1233, 1198, 1143, 1113, 1017, 981, 869, 835, 788, 704 cm^−1^; HRMS (FAB+) calcd for C_26_H_28_NO_9_S [M+H]^+^ 530.1479, found 530.1486. [α]_D_ [20] 41.5 (*c* 1.0, MeOH).

4-Acetamidophenyl (*E*)-2-(4-oxo-4*H*-chromen-5-yl)ethene-1-sulfonate (**6b**): 36.7 mg (95%); White solid; m.p. 208–209 °C; ^1^H NMR (600 MHz, DMSO-*d*_6_) δ 10.07 (s, 1H), 8.79 (d, *J* = 15.4 Hz, 1H), 8.28 (d, *J* = 6.0 Hz, 1H), 7.86–7.83 (m, 1H), 7.77 (dd, *J* = 11.6, 8.0 Hz, 2H), 7.62 (d, *J* = 8.9 Hz, 2H), 7.49 (d, *J* = 15.4 Hz, 1H), 7.30 (d, *J* = 9.1 Hz, 2H), 6.35 (d, *J* = 5.9 Hz, 1H), 2.02 (s, 3H) ppm; ^13^C NMR (150 MHz, DMSO-*d_6_*) δ 178.00, 168.42, 156.97, 156.22, 145.30, 144.17, 138.33, 133.78, 132.48, 125.76, 124.08, 122.78, 121.59, 121.54, 119.98, 113.54, 23.94 ppm; FT-IR (CHCl_3_, film) υ 3303, 3276, 3199, 3139, 3080, 1674, 1636, 1608, 1540, 1504, 1473, 1402, 1365, 1351, 1309, 1262, 1230, 1199, 1165, 1150, 1121, 1036, 1008, 980, 953, 869, 849, 792, 746, 708 cm^−1^; HRMS (FAB+) calcd for C_19_H_16_NO_6_S [M+H]^+^ 386.0693, found 386.0692.

2-Morpholino-2-(4-oxo-4*H*-chromen-5-yl)ethane-1-sulfonyl fluoride (**6c**): 26.3 mg (77%); Orange sticky oil; ^1^H NMR (600 MHz, CDCl_3_) δ 7.79 (d, *J* = 5.9 Hz, 1H), 7.67 (t, *J* = 8.0 Hz, 1H), 7.48 (d, *J* = 8.5 Hz, 1H), 7.46 (d, *J* = 7.6 Hz, 1H), 6.30 (d, *J* = 5.9 Hz, 1H), 4.12 (dd, *J* = 14.8, 7.0 Hz, 1H), 3.92 (ddd, *J* = 14.8, 6.5, 2.9 Hz, 2H), 3.67 (t, *J* = 4.7 Hz, 4H), 2.59–2.56 (m, 2H), 2.53–2.49 (m, 2H) ppm; ^13^C NMR (150 MHz, CDCl_3_) δ 179.64, 158.26, 153.93, 137.28, 132.68, 125.44, 123.04, 119.39, 114.63, 67.15, 56.63, 53.39 (d, *J* = 12.6 Hz), 50.04 ppm; FT-IR (CHCl_3_, film) υ 3072, 2954, 2922, 2852, 1681, 1644, 1590, 1556, 1463, 1393, 1347, 1285, 1228, 1186, 1154, 1105, 1067, 1033, 981, 869, 832, 792, 761, 696, 673 cm^−1^; HRMS (EI+) calcd for C_15_H_16_FNO_5_S [M]^+^ 341.0733, found 341.0730.

## 4. Conclusions

In conclusion, we described the rhodium(III)/ruthenium(II)-catalyzed C–H olefination reactions of chromones, flavones, (thio)xanthone derivatives, 1,4-naphthoquinones, and acridones with ethenesulfonyl fluoride to afford medicinally important SO_2_F-containing compounds. These methodologies have demonstrated good substrate compatibility and functional group tolerance. In addition, the synthetic utility of the protocol was demonstrated through gram-scale synthesis and diverse SuFEx transformations with various nucleophiles. Notably, successful DNA conjugation highlights the potential of these SO_2_F-containing scaffolds for DNA-encoded library (DEL) chemistry. Furthermore, all synthesized compounds were evaluated for cytotoxic activity against human lung adenocarcinoma A549 cell lines. Six compounds exhibiting EC_50_ values below 15 μM were further evaluated against human breast adenocarcinoma MCF-7, human liver carcinoma HepG2, and human ovarian carcinoma SKOV3 cells. Among the selected compounds, compound **5aa,** showing a high cytotoxic effect, was further screened against other cancer cell lines and was found to exhibit cytotoxic effect. These preliminary cytotoxicity results suggest that synthesized compounds, especially compounds such as compound **5aa**, warrant further biological investigation.

## Figures and Tables

**Table 1:** Selected optimization of reaction conditions *^a^*.

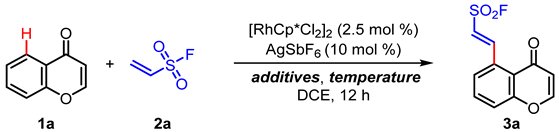			
Entry	Additive (mol%)	Temp.	Yield (%) *^b^*
1	PivOH (200)	120 °C	17
2	AcOH (200)	120 °C	21
3	Ag_2_O (200)	120 °C	40
4	AgTFA (200)	120 °C	30
5	NaOAc (200)	120 °C	9
6	AgOAc (200)	120 °C	89
7	Ag_2_CO_3_ (200)	120 °C	84
8	CuOAc (200)	120 °C	79
9	Cu(OAc)_2_ (200)	120 °C	80
10 *^c^*	Cu(OAc)_2_ (200)	120 °C	83
11 *^c^*	Cu(OAc)_2_ (200)	90 °C	88
**12 *^c^***	**Cu(OAc)_2_ (50)**	**90 °C**	**91**
13	AgOAc (200)	60 °C	98
14	AgOAc (200)	R.T.	trace
**15**	**AgOAc (150)**	**60 °C**	**98**
16	AgOAc (100)	60 °C	82
17 *^d^*	AgOAc (150)	60 °C	N.R.
18 *^e^*	AgOAc (150)	60 °C	N.R.

*^a^* Reaction conditions: **1a** (0.2 mmol), **2a** (0.24 mmol, 1.2 equiv), [RhCp*Cl_2_]_2_ (2.5 mol%), additive (quantity noted), DCE (1 mL) under air at noted temperature for 12 h in pressure tubes. *^b^* Yield by flash column chromatography. *^c^* [Ru(*p*-cymene)Cl_2_]_2_ (2.5 mol%) was used as a catalyst. *^d^* The reaction was carried out in the absence of catalyst. *^e^* The reaction was carried out in the absence of AgSbF_6_.

**Table 2:** Cytotoxicity of synthetic compounds against human A549 cancer cell lines *^a^*.

Compound	EC_50_ (μM)	Compound	EC_50_ (μM)	Compound	EC_50_ (μM)
**3a**	12.03 ± 0.99	**3k**	48.4 ± 2.7	**5c**	>50
**3b**	11.93 ± 0.69	**3l**	16.5 ± 1.1	**5c’**	>50
**3c**	9.13 ± 0.36	**3m**	13.56 ± 0.56	**5d**	21.84 ± 0.18
**3d**	11.79 ± 0.52	**3n**	>50	**5e**	48.6 ± 1.3
**3e**	22.7 ± 1.5	**3o**	>50	**5f**	27.36 ± 0.47
**3f**	49.74 ± 0.78	**5a**	25.04 ± 0.07	**5fa**	>50
**3g**	15.98 ± 0.24	**5aa**	10.44 ± 0.40	**5g**	>50
**3i**	17.4 ± 1.1	**5b**	>50	**5ga**	>50
**3j**	48.3 ± 4.6	**5ba**	>50	**5h**	19.48 ± 0.26

*^a^* EC_50_ values are presented as the mean ± SD from three independent experiments. EC_50_ was defined as the concentration of a compound required to reduce cell viability to 50% of the vehicle-control value.

**Table 3:** Cytotoxicity of selected compounds against various human cancer cell lines *^a^*.

Compound	A549 (EC_50_, μM)	MCF-7 (EC_50_, μM)	SKOV3 (EC_50_, μM)	HepG2 (EC_50_, μM)
**3a**	12.03 ± 0.99	14.86 ± 0.92	24.02 ± 0.92	13.04 ± 0.68
**3b**	11.93 ± 0.69	24.1 ± 1.9	29.6 ± 1.1	27.14 ± 0.90
**3c**	9.13 ± 0.36	15.54 ± 0.45	20.5 ± 1.7	7.26 ± 0.44
**3d**	11.79 ± 0.52	11.55 ± 0.16	14.28 ± 0.39	7.0 ± 1.0
**3m**	13.56 ± 0.56	12.53 ± 0.91	29.6 ± 2.6	26.35 ± 0.64
**5aa**	10.44 ± 0.40	14.08 ± 0.33	6.0 ± 1.1	3.73 ± 0.28

*^a^* EC_50_ values are presented as the mean ± SD from three independent experiments. EC_50_ was defined as the concentration of a compound required to reduce cell viability to 50% of the vehicle-control value.

## Data Availability

The original contributions presented in this study are included in the Article and Supplementary Materials. Supplementary Materials are available for ^1^H and ^13^C NMR copies of all products and DNA conjugation data.

## Supplementary Materials

The following supporting information can be downloaded at: https://www.mdpi.com/article/10.3390/molecules31173094/s1.
