# Supplementary material for: Design, Synthesis and Anticancer Evaluation of Novel SO2F-Containing Medicinally Privileged Structures via Ketone-Directed C–H Functionalization
Source: Molecules. 2026 Sep 3;31(17):3094. doi: 10.3390/molecules31173094 (PMC13567039; doi:10.3390/molecules31173094)

Supplementary Materials

Design, Synthesis and Anticancer Evaluation of Novel SO_2F-Containing Medicinally Privileged Structures *via* Ketone-Directed C−H Functionalization

Seungwan Kim [1,†], Lingayya Rajaka [1,2,†], Saegun Kim [3,†], In Jae Bang [2], Hyun Woo Lee [1], Minseo Choi [4], Jihyo Lee [1], Myunghwa Jung [1], Hyosik Woo [1], Sangil Han [5], Ki Yong Lee [1,6], Hyun Jin Kim [4,7,*], Ha Ryong Kim [1,6,*] and Sang Hoon Han [1,*]

1 College of Pharmacy, Korea University, Sejong 30019, Republic of Korea

2 Institute of Pharmaceutical Science and Translational Research, Korea University, Sejong 30019, Republic of Korea

3 College of Pharmacy, Research Institute of Pharmaceutical Sciences, Sookmyung Women's University, Seoul 04310, Republic of Korea

4 Data Convergence Drug Research Center, Therapeutics and Biotechnology Division, Korea Research Institute of Chemical Technology (KRICT), Daejeon 34114, Republic of Korea

5 Department of Innovative Pharmaceutical Sciences, Kyungpook National University, Daegu 41566, Republic of Korea

6 Interdisciplinary Major Program in Innovative Pharmaceutical Sciences, Korea University, Sejong 30019, Republic of Korea

7 Medicinal Chemistry and Pharmacology, Korea National University of Science and Technology, Daejeon 34113, Republic of Korea

* Corresponding author:

E-mail: hans3@korea.ac.kr (S.H.H.); haryongkim@korea.ac.kr (H.R.K.); hyunjin@krict.re.kr (H.J.K.)

[†] These authors contributed equally to this work.

List of the Contents

1. General information for DNA SuFEx Conjugation -- S3

2. LC and Deconvoluted Mass Spectra of on-DNA Products ----------------------------------- S5

3. NMR spectra of all products -- S7

4. HR-MS analytical data of all products --- S70

1. General Information for DNA SuFEx Conjugation

All commercial reagents were used as received without further purification. The molecular structure of the AOP-headpiece is depicted in Figure S1. Reaction was performed in Eppendorf Safe-Lock microcentrifuge tubes without stirring. Reaction mixtures were vortexed with a Thermomixer and subsequently centrifuged with a Eppendorf™ Refrigerated Centrifuge 5424 R (max. speed: 15,000 rpm). After the final ethanol precipitation, a Eppendorf™ Refrigerated Centrifuge 5424 R (max. speed: 15,000 rpm) was used for centrifugation. UPLC analyses were performed on a condition a tandem ultra-performance liquid chromatography/mass spectrometry (UPLC-MS) analysis of oligonucleotides and DNA-barcoded compounds was performed on a Waters SQ Detector 2 mass spectrometer (running in negative ion mode electrospray ionization) connected to a waters Acquity UPLC (outfitted with a Waters Acquity UPLC Oligonucleotide BEH C18 column [130 Å, 1.7 µm (2.1 × 100 mm)]) and a Waters Acquity TUV Detector. Samples were injected on to the column (heated to 70 °C) and eluted according to the following parameters: 3-90% solvent B over 6.2 minutes at a flow rate of 0.3 mL/min; solvent A: 250 mM HFIP, 8 mM TEA in LCMS-grade water; solvent B: LCMS-grade MeOH. Mass spectra were recorded from 500-2000 m/z and deconvoluted using Bayerspray software to afford the full molecular weight of the parent ions. Yields are reported as a % conversion as determined from UPLC analysis by comparing the integrals of all DNA-derived compounds at 260 nm (low molecular weight impurities, M < 1000 g/mol, which were not DNA-derived, and impurities from previous reactions were not taken into account).

AOP-Headpiece

Sequence: 5’-/Phos/GAGTCA/iSp9-PEG/iAm C7_CO-PEG4-NH2/iSp9-PEG/TGACTCCC-3’

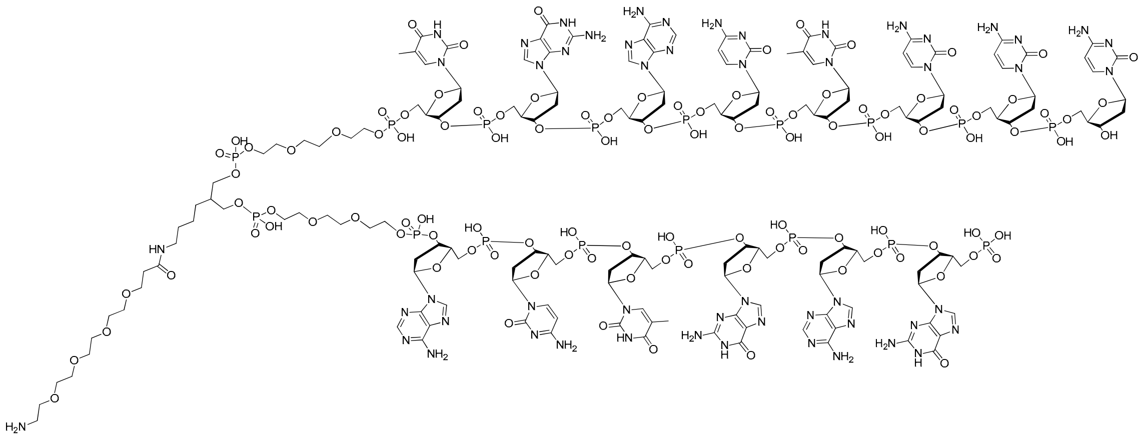

Figure S1. Molecular structure of the AOP-headpiece. MW = 5184.52

2. LC and Deconvoluted Mass Spectra of on-DNA Products

6d

Yield: 63%

Chemical formula: $C_{176}H_{242}N_{53}O_{110}P_{17}S$

Calculated mass: 5416.0232

Observed mass: 5414.9396

Retention time: 3.98 min

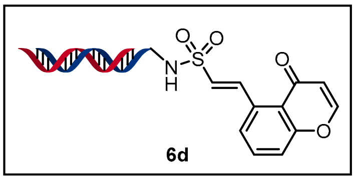

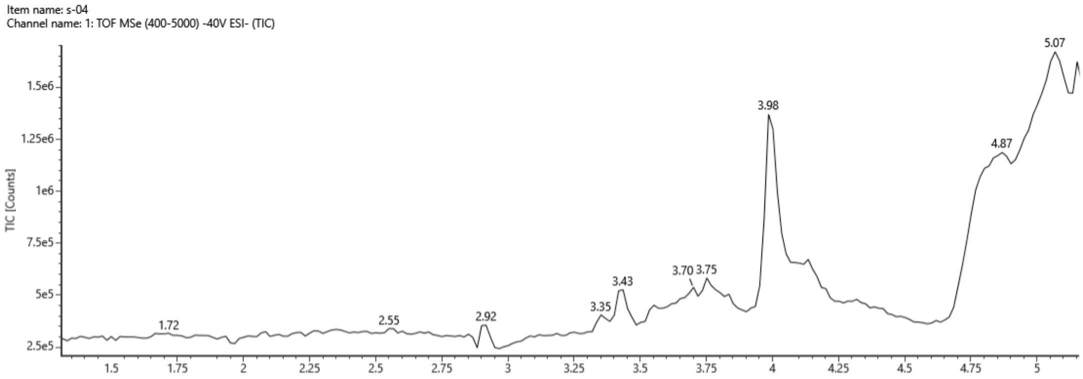

Figure S2. TIC trace of SuFEx derivative **6d**

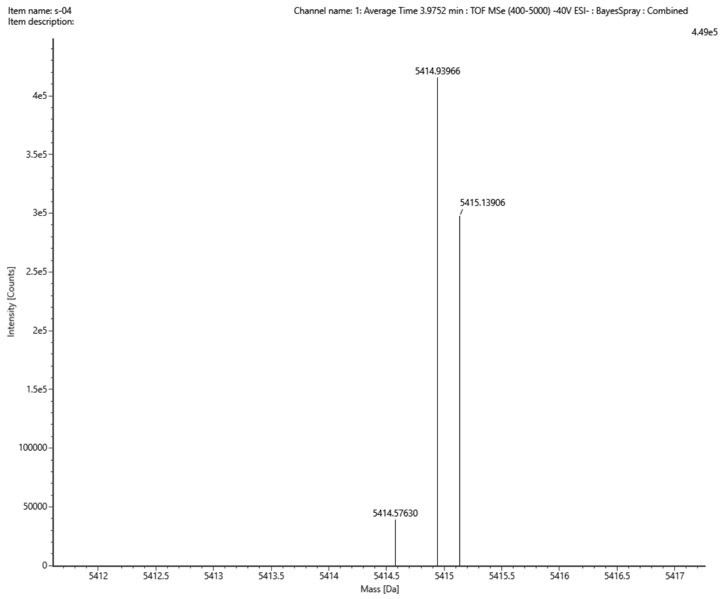

Figure S3. Deconvolution mass spectrum of SuFEx derivative **6d**

3. NMR spectra of all products

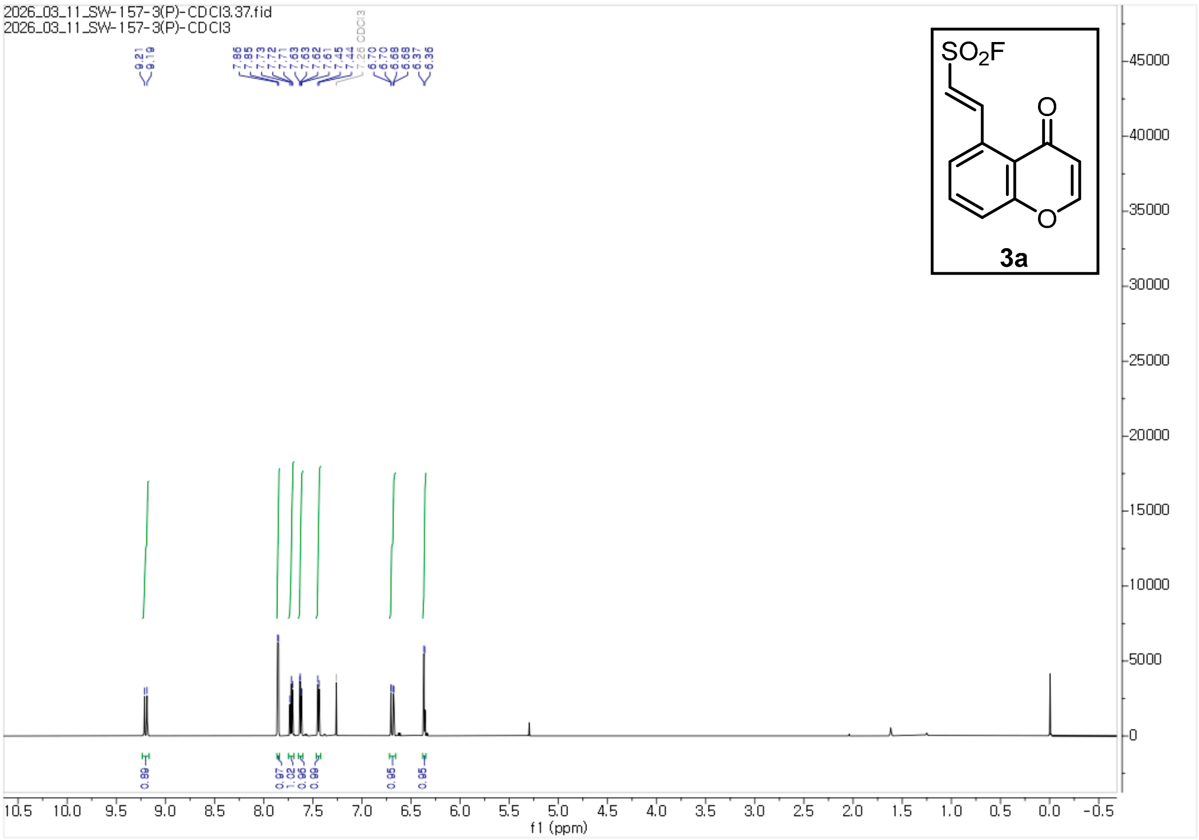

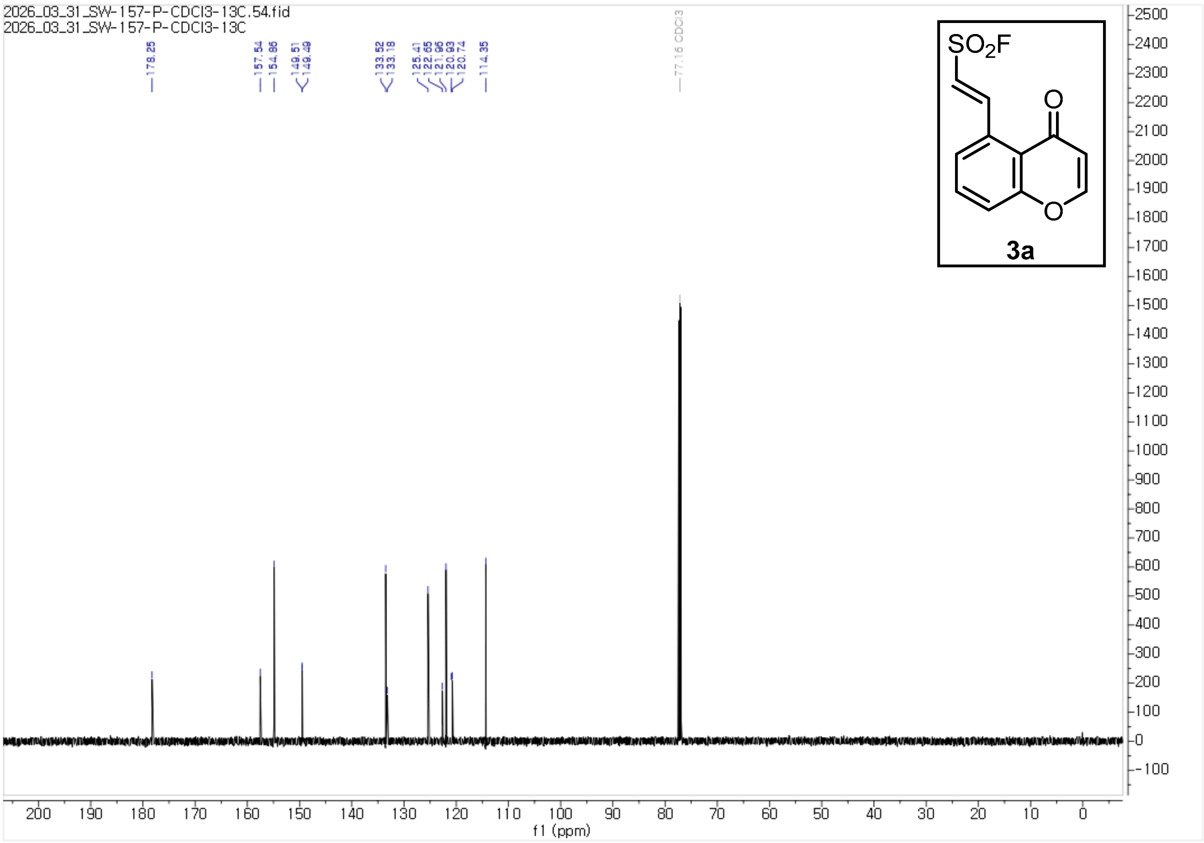

2026_03_31_SW-157-P-CDCl3-13C.54.fid
2026_03_31_SW-157-P-CDCl3-13C
178.25
157.54
154.86
149.51
149.49
133.52
133.18
125.41
122.65
121.96
120.93
120.74
114.35
77.16 CDCl3
SO2F
O
3a
f1 (ppm)

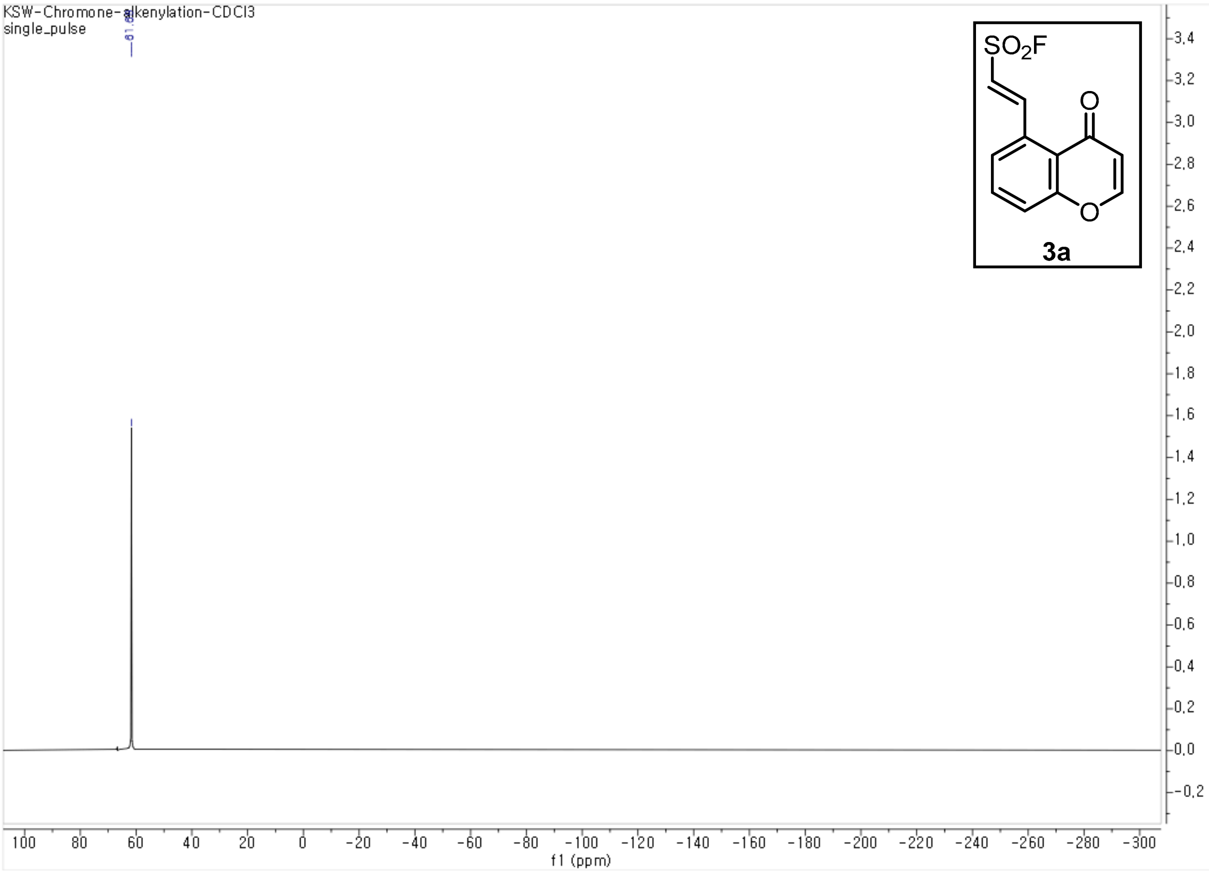
KSW-Chromone-alkenylation-CDCl3
single_pulse
SO_2F
O
O
3a
3,4
3,2
3,0
2,8
2,6
2,4
2,2
2,0
1,8
1,6
1,4
1,2
1,0
0,8
0,6
0,4
0,2
0,0
-0,2
100 80 60 40 20 0 -20 -40 -60 -80 -100 -120 -140 -160 -180 -200 -220 -240 -260 -280 -300
f1 (ppm)

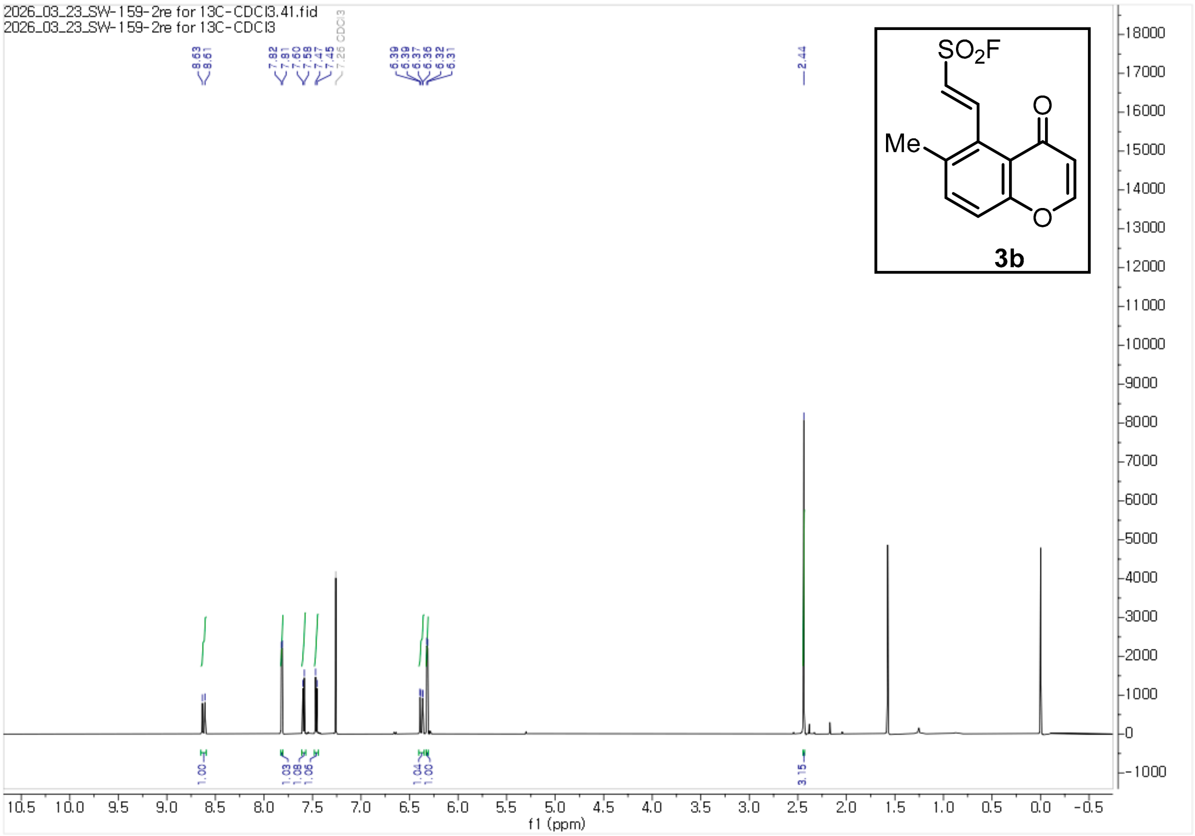
2026_03_23_SW-159-2re for 13C-CDCl3.41.fid
2026_03_23_SW-159-2re for 13C-CDCl3
8.63
8.61
7.82
7.81
7.60
7.58
7.47
7.45
7.26 CDCl3
6.39
6.39
6.37
6.36
6.32
6.31
2.44
SO2F
O
Me
O
3b
1.00
1.03
1.08
1.06
1.04
1.00
3.15
f1 (ppm)

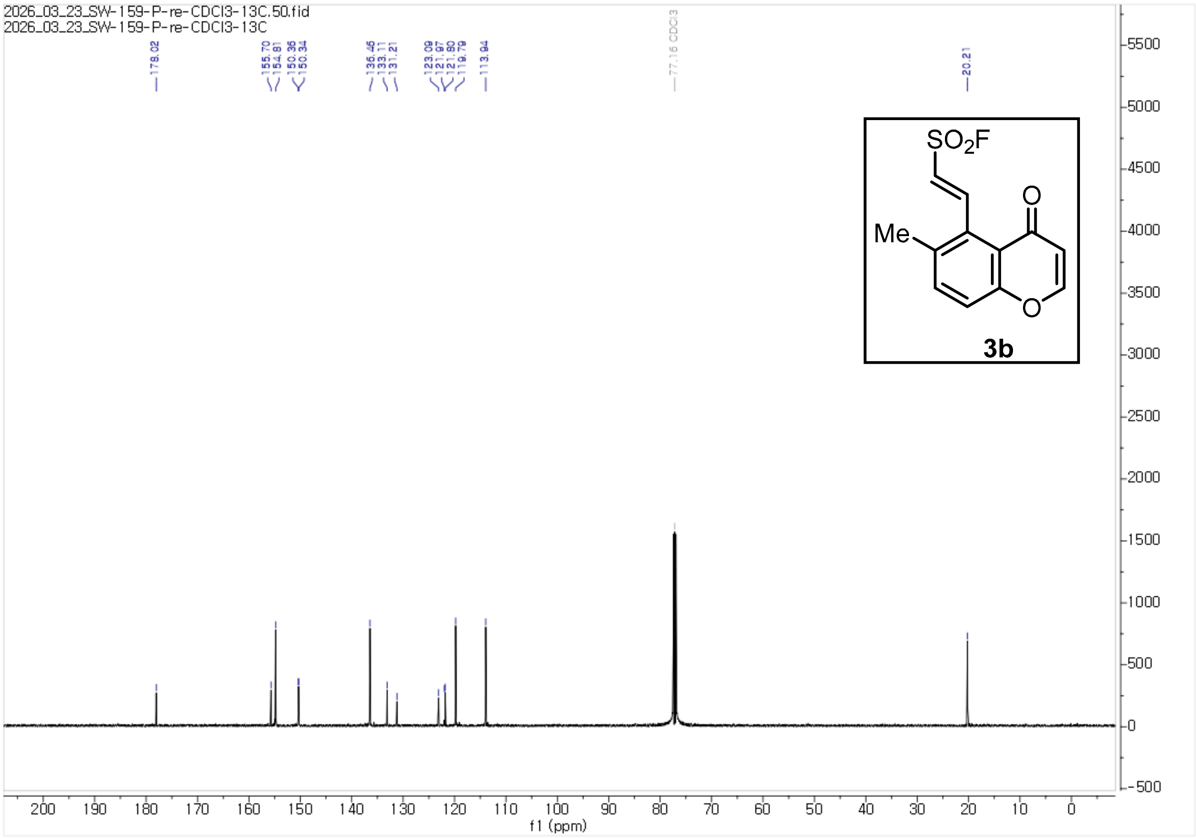
2026_03_23_SW-159-P-re-CDCl3-13C.50.fid
2026_03_23_SW-159-P-re-CDCl3-13C
178.02
155.70
154.81
150.36
150.34
136.46
133.11
131.21
123.09
121.97
121.80
119.79
113.94
77.16 CDCl3
20.21
SO2F
O
Me
O
3b
f1 (ppm)

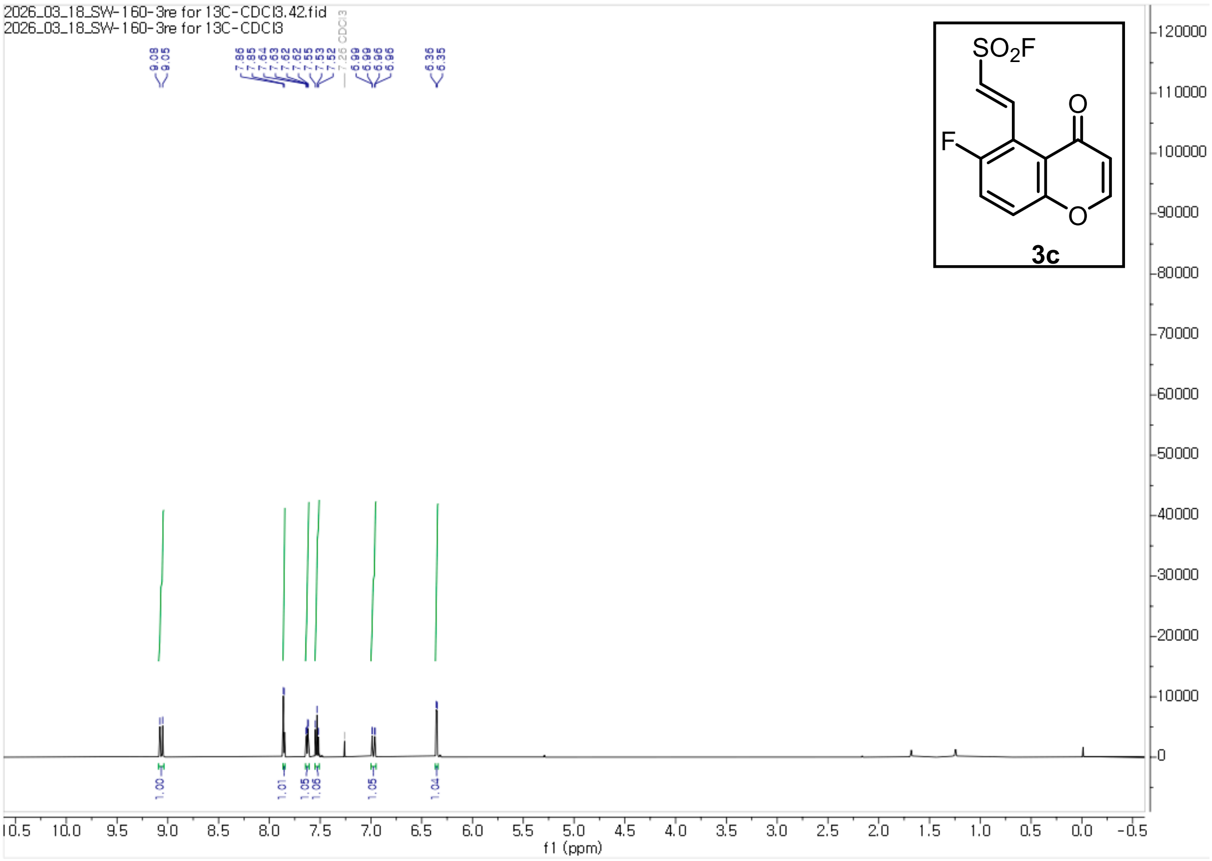
2026_03_18_SW-160-3re for 13C-CDCl3.42.fid
2026_03_18_SW-160-3re for 13C-CDCl3
SO2F
F
O
O
3c
f1 (ppm)

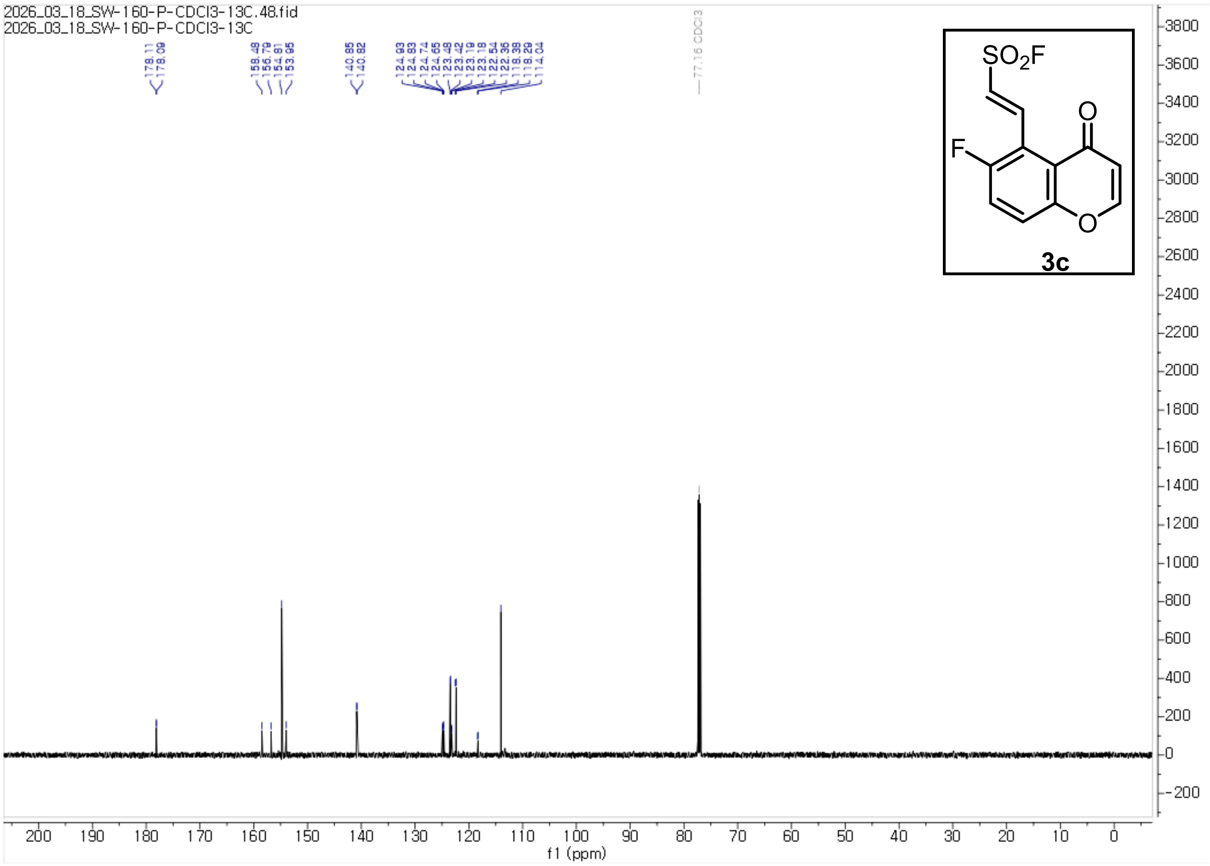
2026_03_18_SW-160-P-CDCl3-13C.48.fid
2026_03_18_SW-160-P-CDCl3-13C
178.11
178.09
158.48
156.79
154.81
153.95
140.85
140.82
124.93
124.83
124.74
124.65
123.48
123.42
123.19
123.18
122.54
122.36
118.38
118.29
114.04
77.16 CDCl3
SO2F
O
F
O
3c
f1 (ppm)

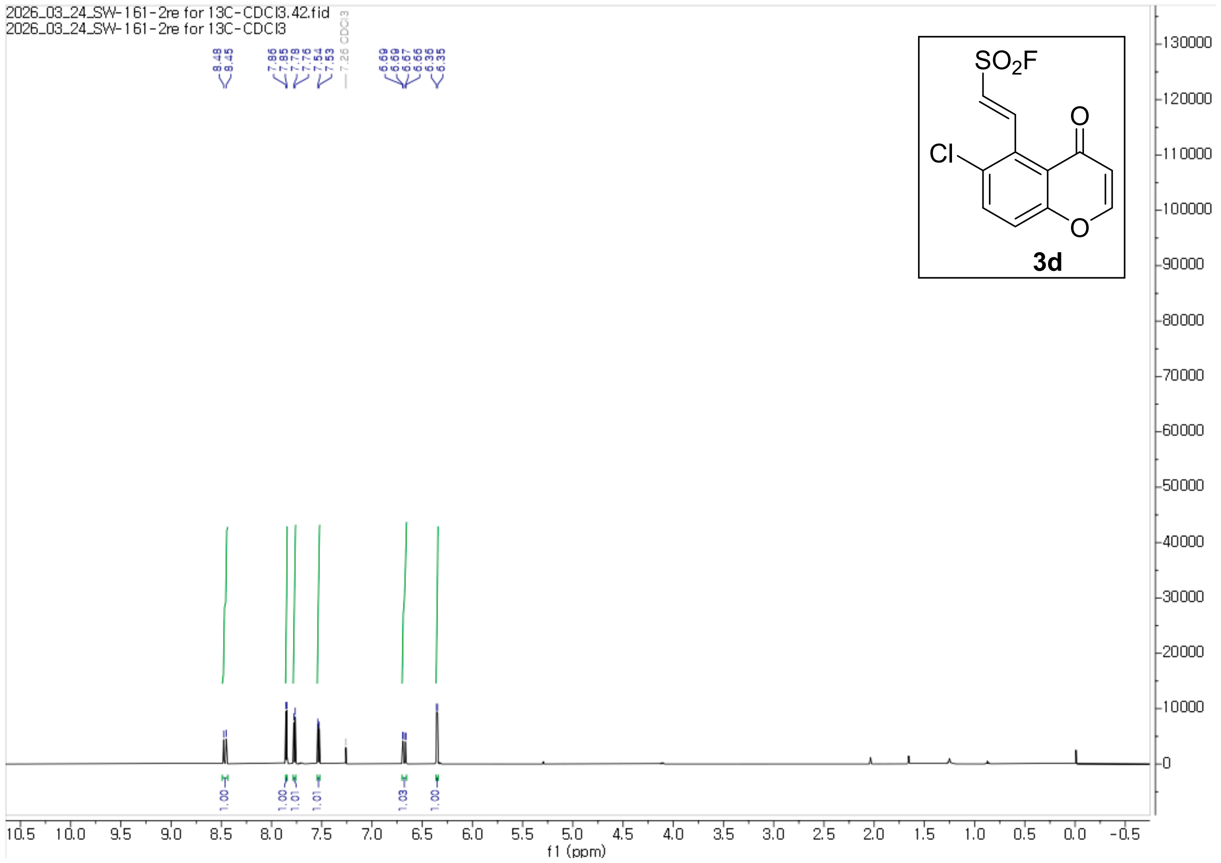
2026_03_24_SW-161-2re for 13C-CDCl3.42.fid
2026_03_24_SW-161-2re for 13C-CDCl3
8.48
8.45
7.86
7.85
7.78
7.76
7.54
7.53
7.26 CDCl3
6.69
6.69
6.67
6.66
6.36
6.35
SO2F
O
Cl
O
3d
1.00
1.00
1.01
1.01
1.03
1.00
f1 (ppm)

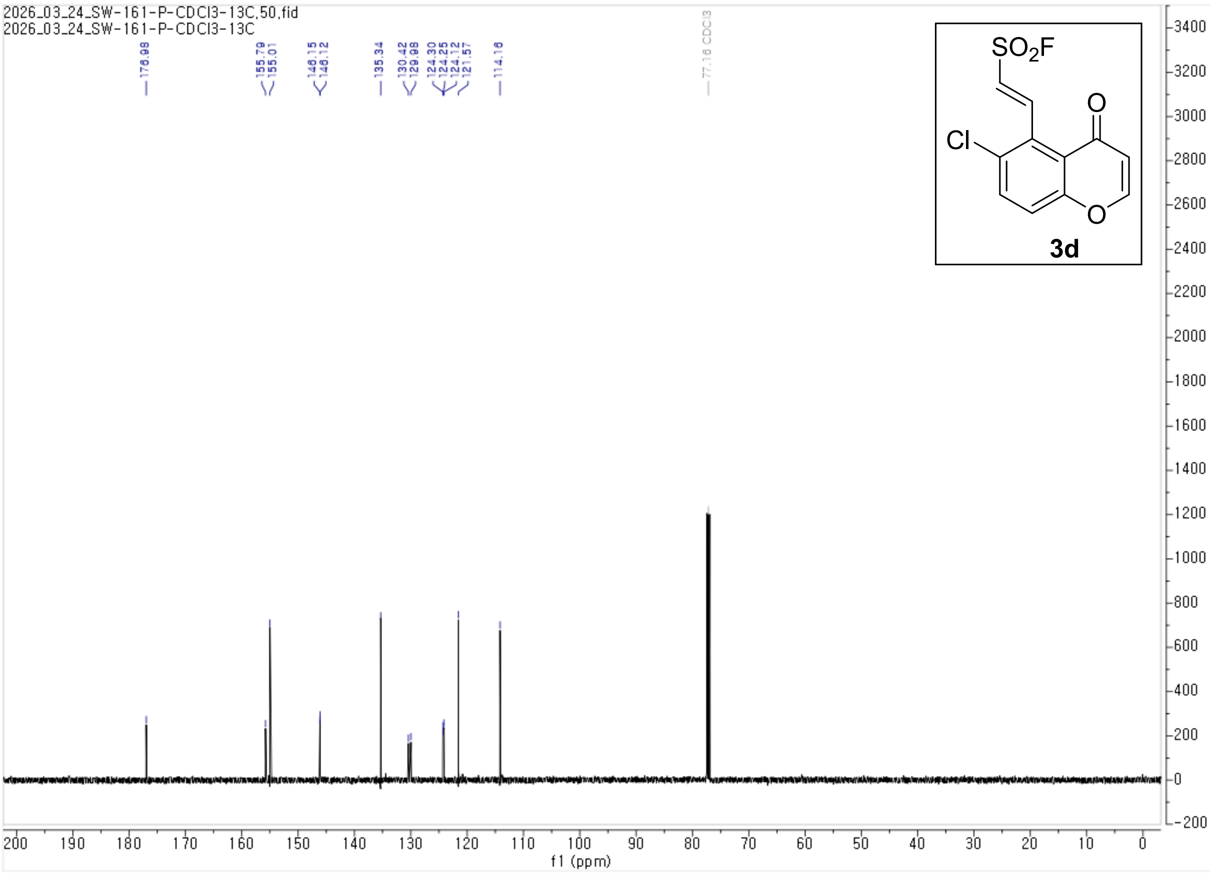
2026_03_24_SW-161-P-CDCl3-13C.50.fid
2026_03_24_SW-161-P-CDCl3-13C
176.98
155.79
155.01
146.15
146.12
135.34
130.42
129.98
124.30
124.25
124.12
121.57
114.16
77.16 CDCl3
SO2F
O
Cl
O
3d
f1 (ppm)

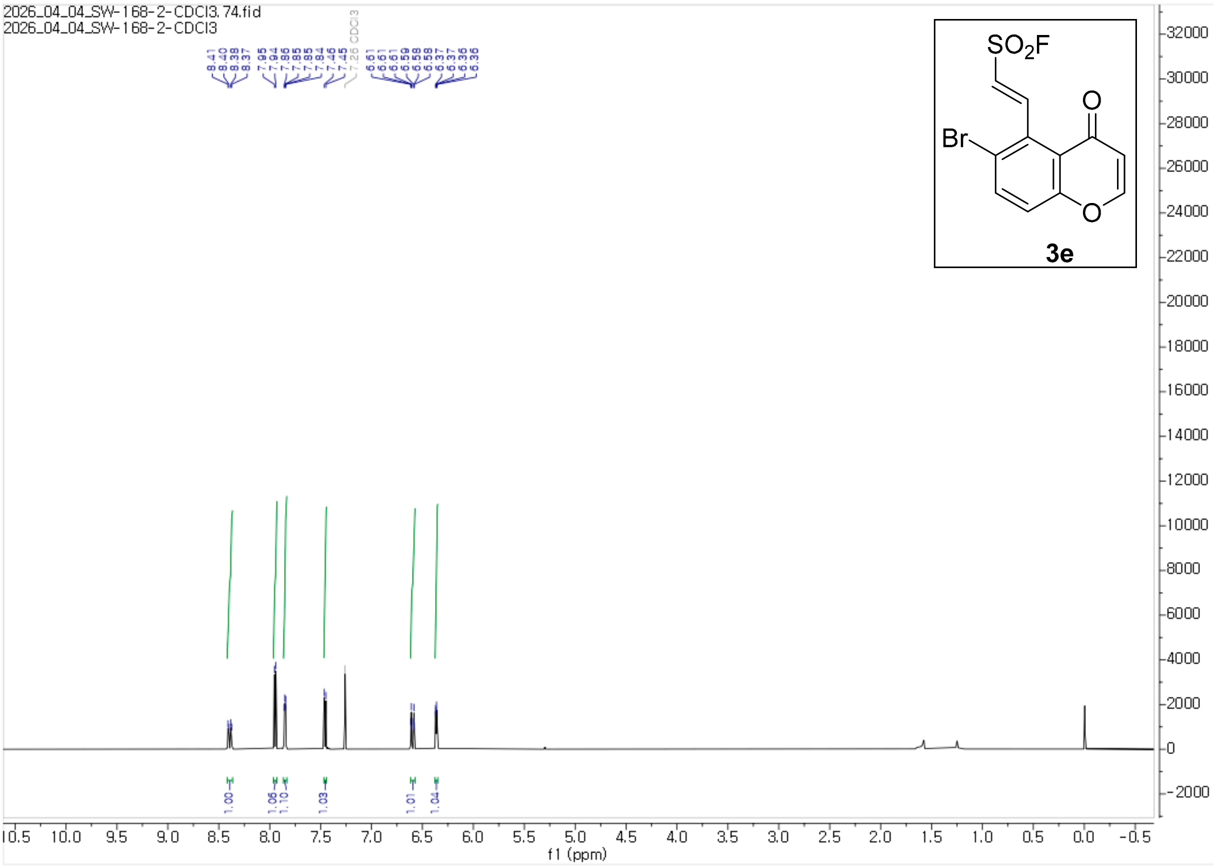
2026_04_04_SW-168-2-CDCl3.74.fid
2026_04_04_SW-168-2-CDCl3
SO2F
O
Br
O
3e
f1 (ppm)

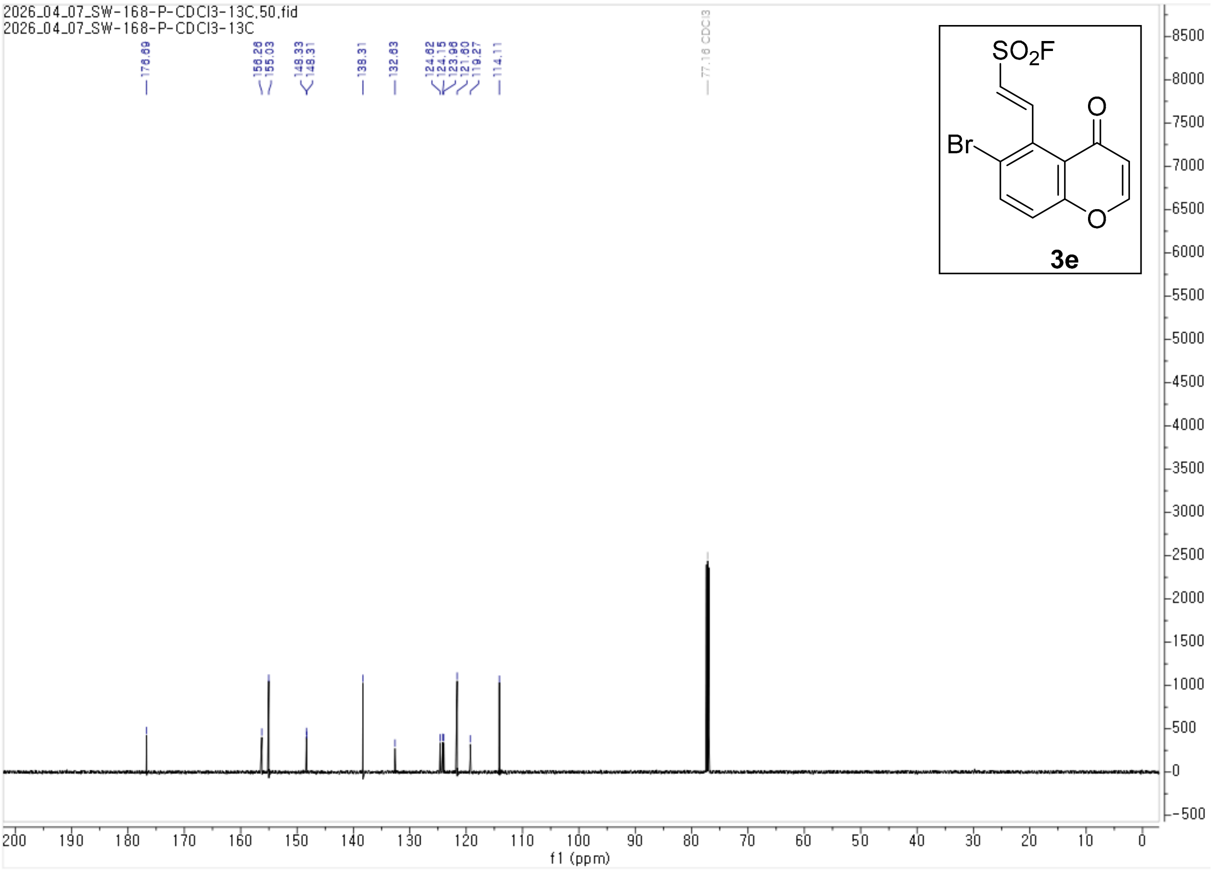
2026_04_07_SW-168-P-CDCl3-13C,50,fid
2026_04_07_SW-168-P-CDCl3-13C
SO2F
Br
O
O
3e
f1 (ppm)

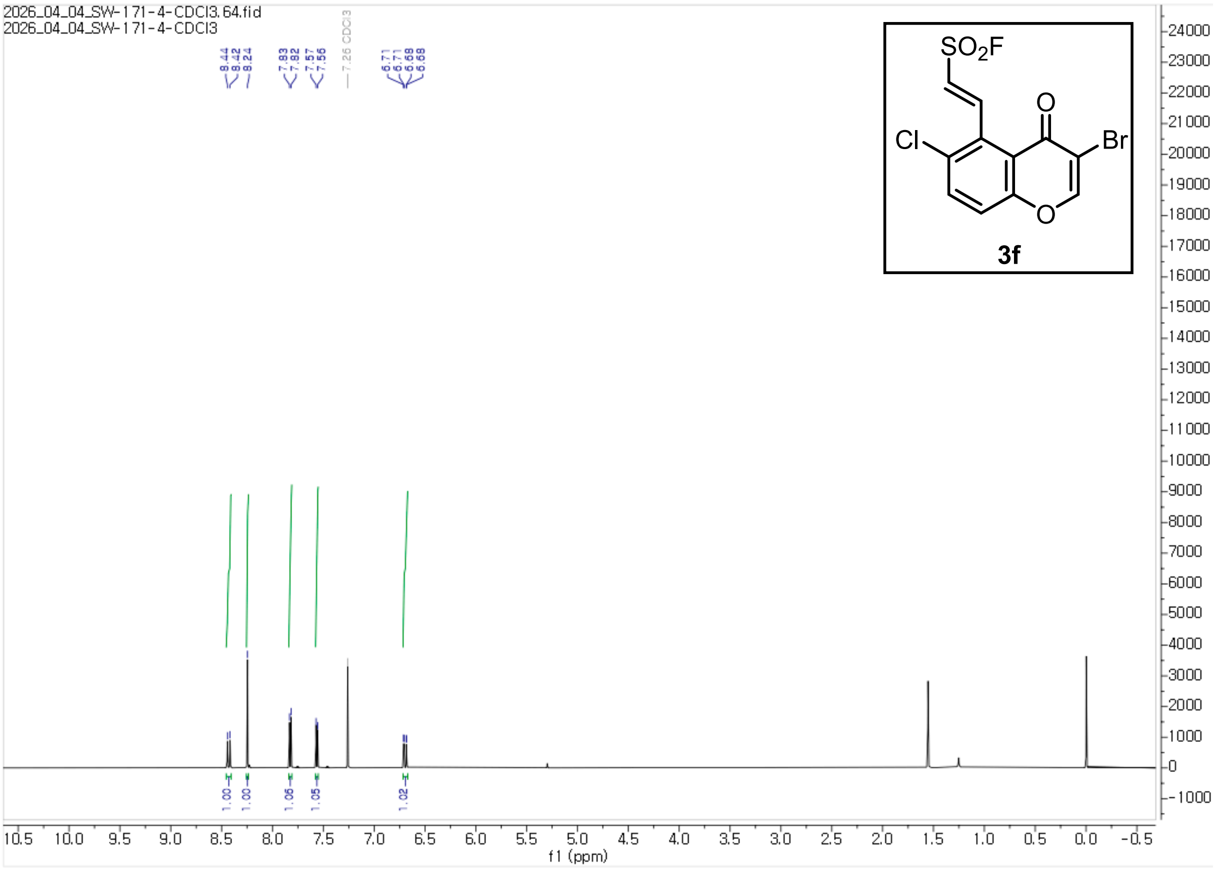
2026_04_04_SW-171-4-CDCl3.64.fid
2026_04_04_SW-171-4-CDCl3
8.44
8.42
8.24
7.83
7.82
7.57
7.56
7.26 CDCl3
6.71
6.71
6.68
6.68
SO2F
O
Cl
Br
O
3f
1.00
1.00
1.06
1.05
1.02
f1 (ppm)

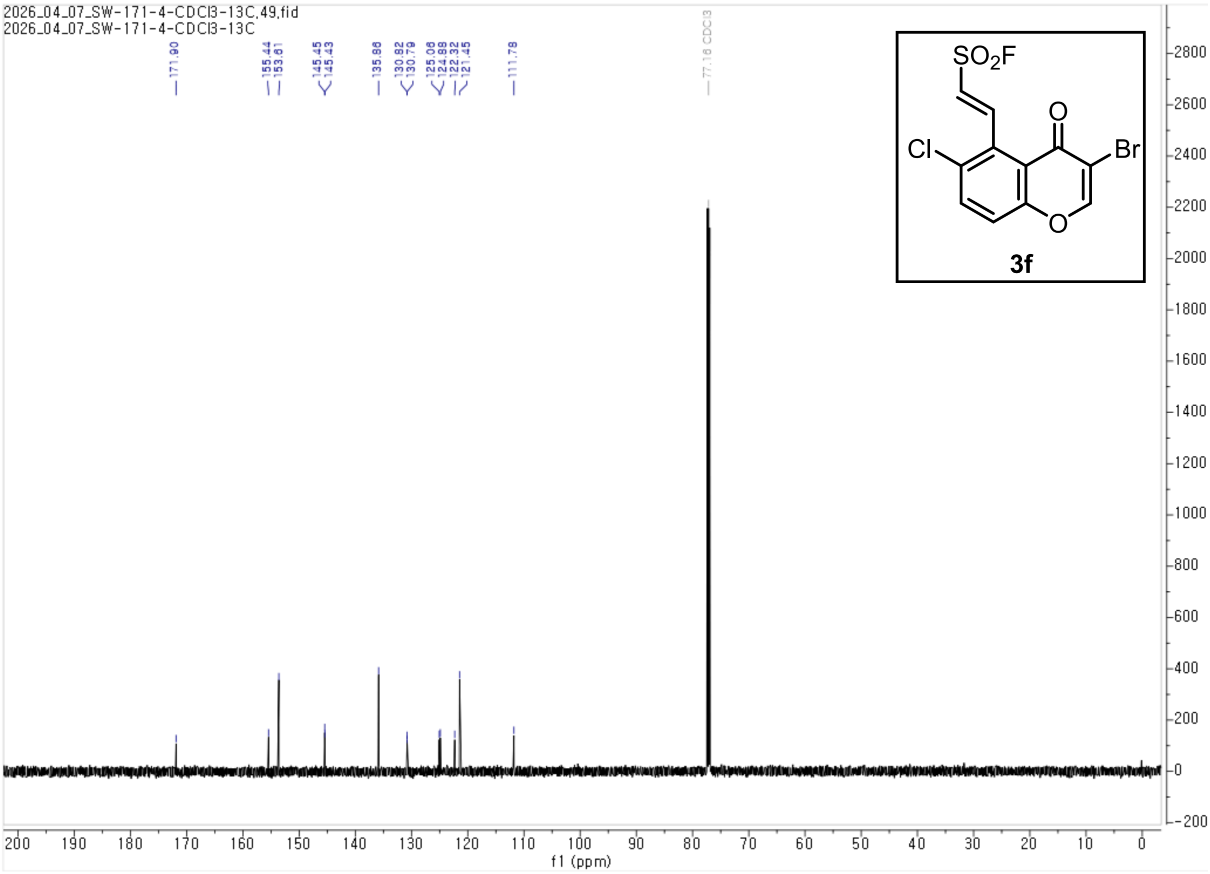

2026_04_07_SW-171-4-CDCl3-13C.49.fid
2026_04_07_SW-171-4-CDCl3-13C
171.90
155.44
153.61
145.45
145.43
135.88
130.92
130.79
125.06
124.88
122.32
121.45
111.78
77.16 CDCl3
SO2F
O
Cl
Br
O
3f
f1 (ppm)

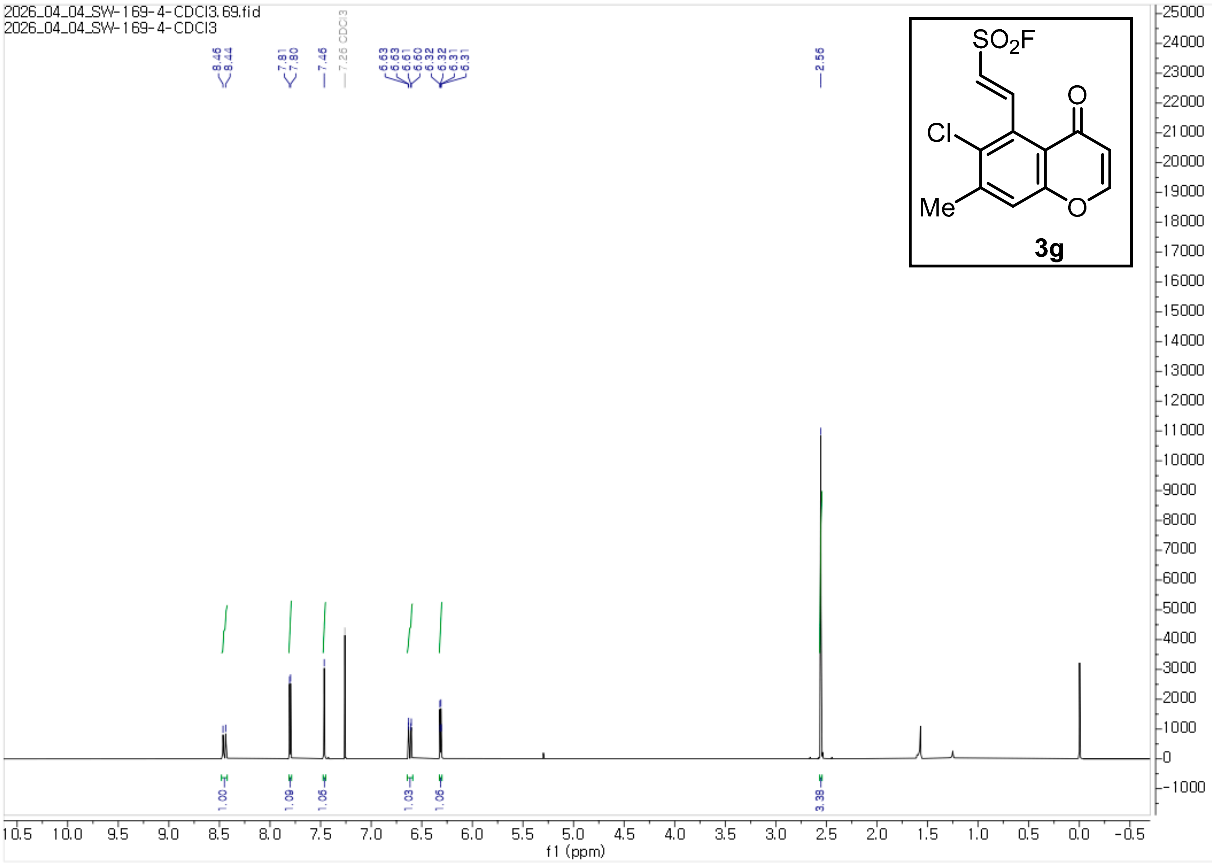
2026_04_04_SW-169-4-CDCl3.69.fid
2026_04_04_SW-169-4-CDCl3
8.45
8.44
7.81
7.80
7.46
7.26 CDCl3
6.63
6.63
6.61
6.60
6.32
6.32
6.31
6.31
2.56
1.00
1.09
1.06
1.03
1.06
3.38
f1 (ppm)
SO2F
O
Cl
Me
O
3g

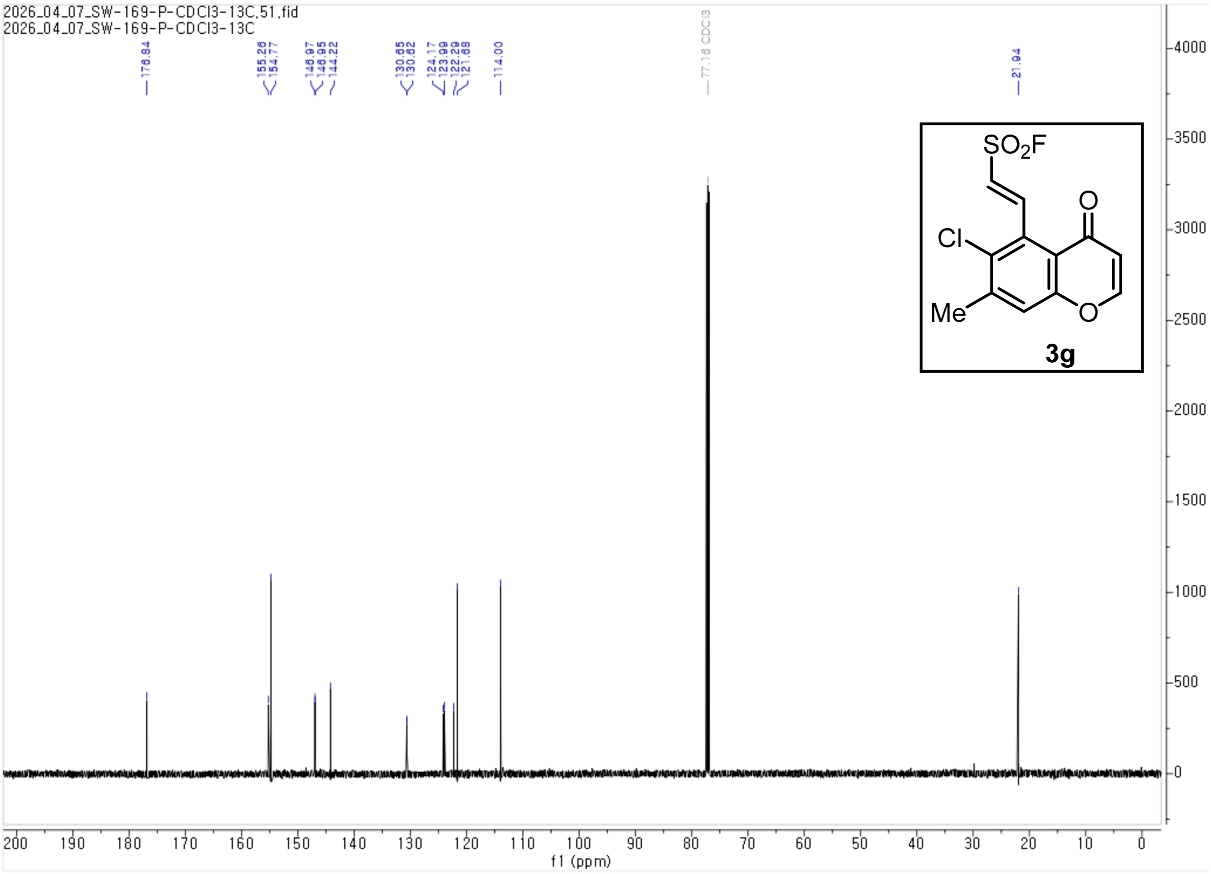
2026_04_07_SW-169-P-CDCl3-13C.51.fid
2026_04_07_SW-169-P-CDCl3-13C
176.84
155.28
154.77
146.97
146.95
144.22
130.65
130.62
124.17
123.99
122.29
121.88
114.00
77.16 CDCl3
21.94
SO2F
O
Cl
Me
O
3g
f1 (ppm)

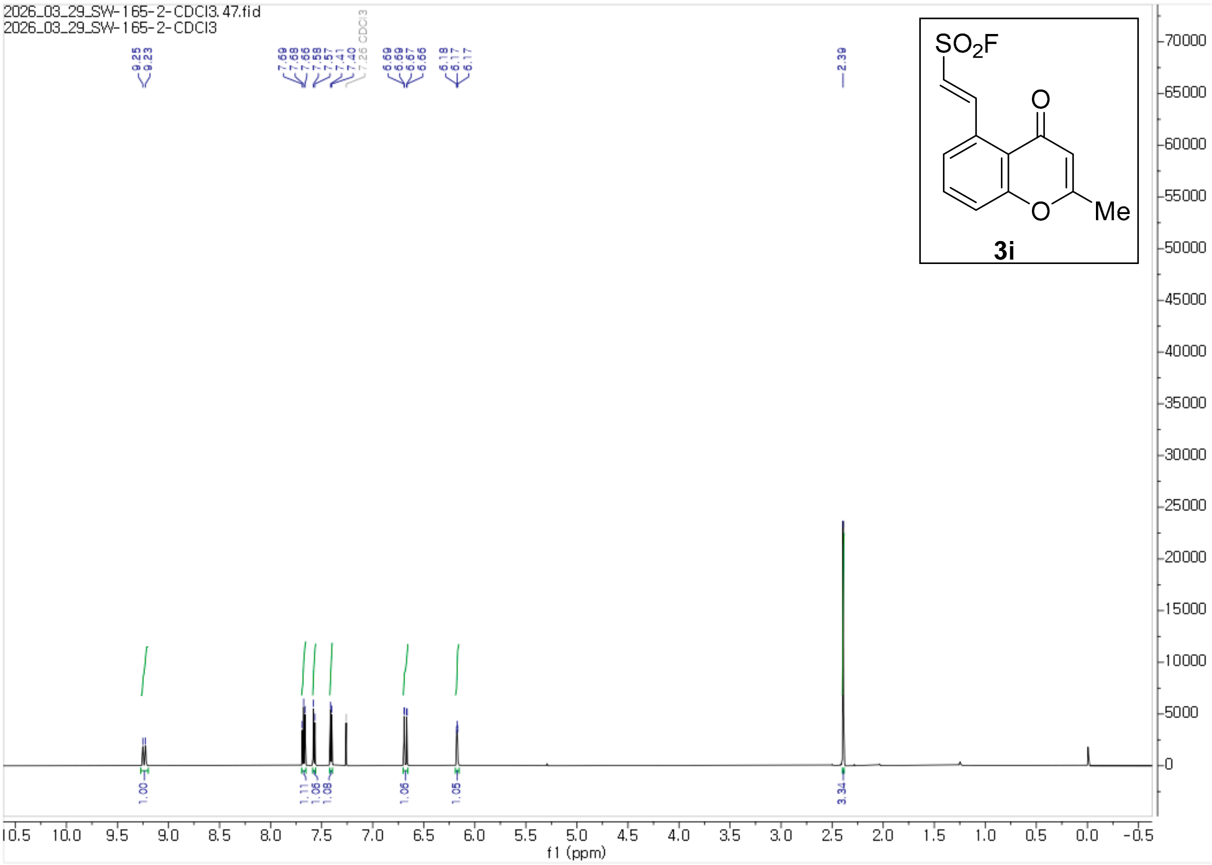

2026_03_29_SW-165-2-CDCl3.47.fid
2026_03_29_SW-165-2-CDCl3
SO2F
O
O
Me
3i
f1 (ppm)

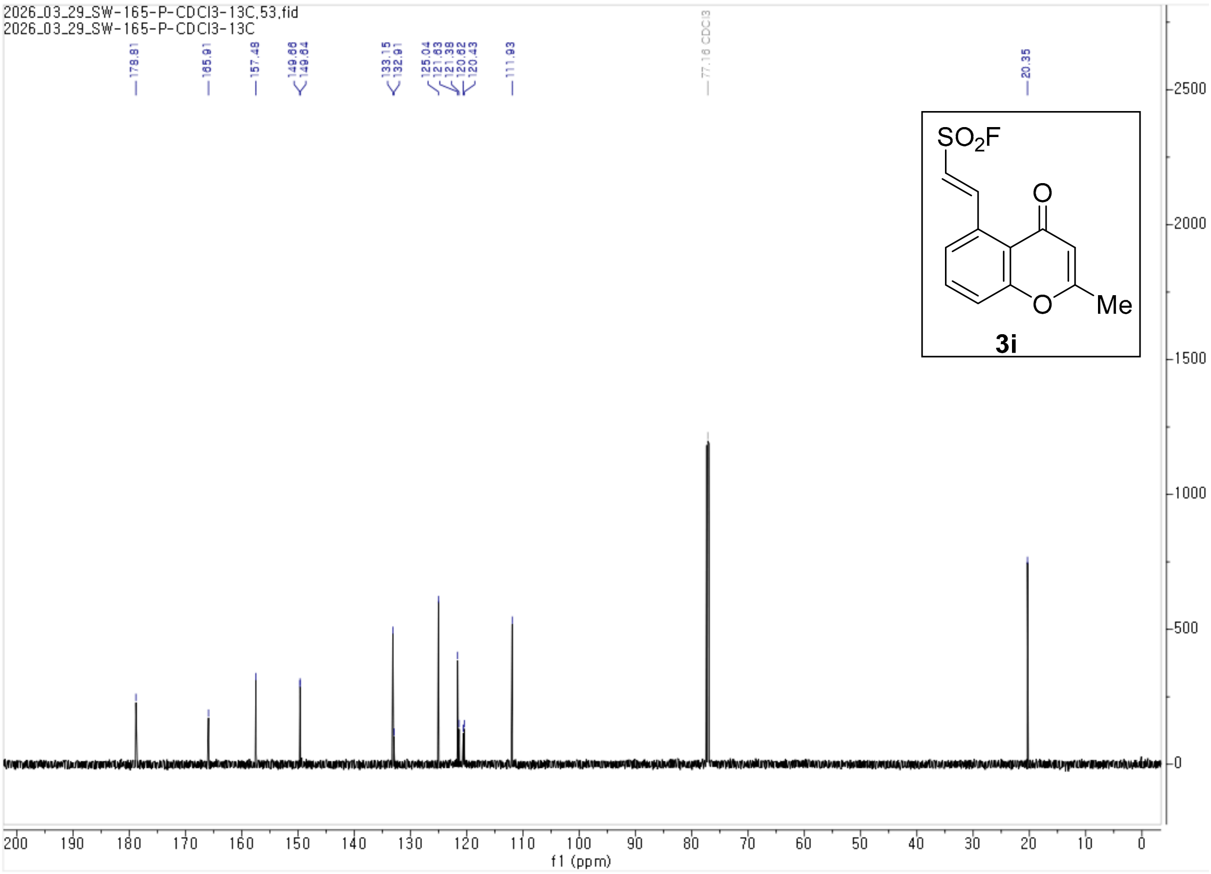
2026_03_29_SW-165-P-CDCl3-13C,53,fid
2026_03_29_SW-165-P-CDCl3-13C
178.81
165.91
157.48
149.86
149.64
133.15
132.91
125.04
121.83
121.38
120.62
120.43
111.93
77.16 CDCl3
20.35
SO2F
O
O
Me
3i
f1 (ppm)

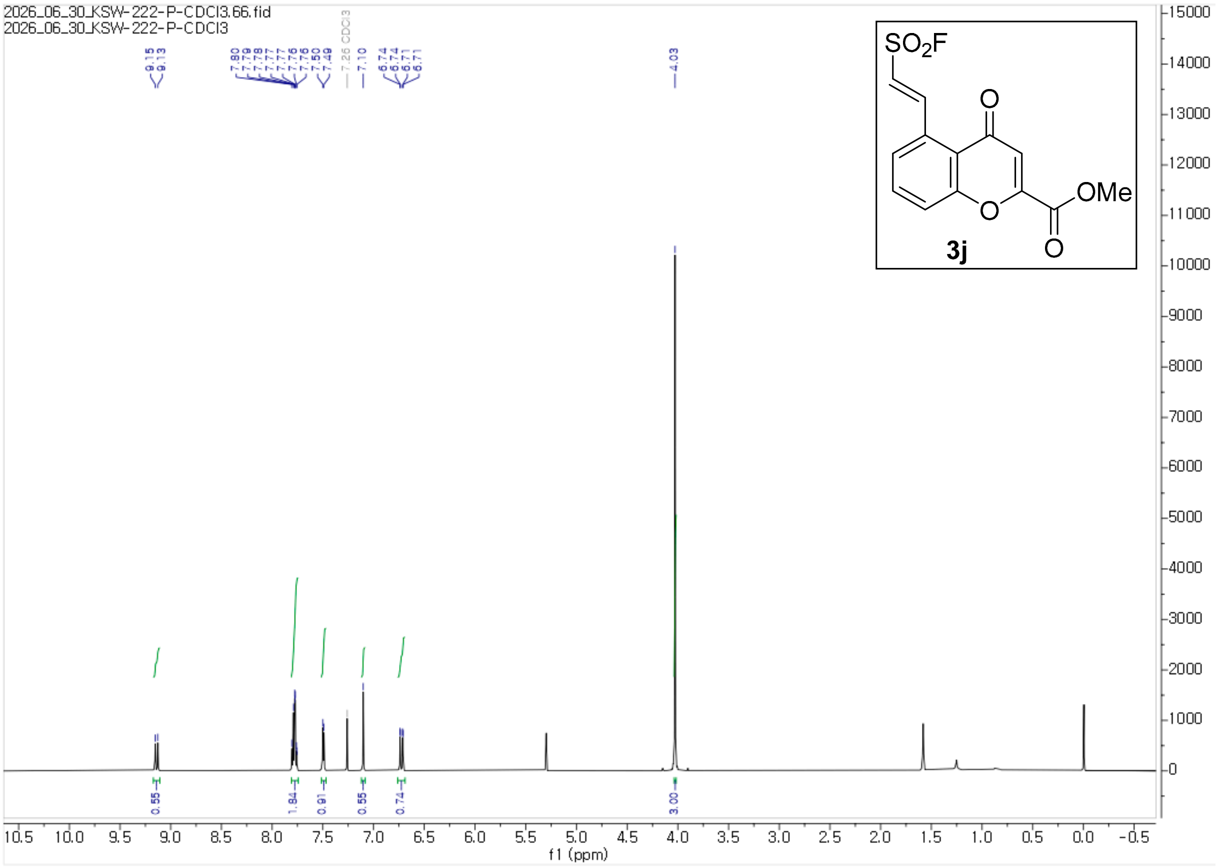
2026_06_30_KSW-222-P-CDCl3.66.fid
2026_06_30_KSW-222-P-CDCl3
9.15
9.13
7.80
7.79
7.78
7.77
7.77
7.76
7.76
7.50
7.49
7.26 CDCl3
7.10
6.74
6.74
6.71
6.71
4.03
SO2F
O
OMe
O
3j
O
0.55
1.84
0.91
0.55
0.74
3.00
f1 (ppm)

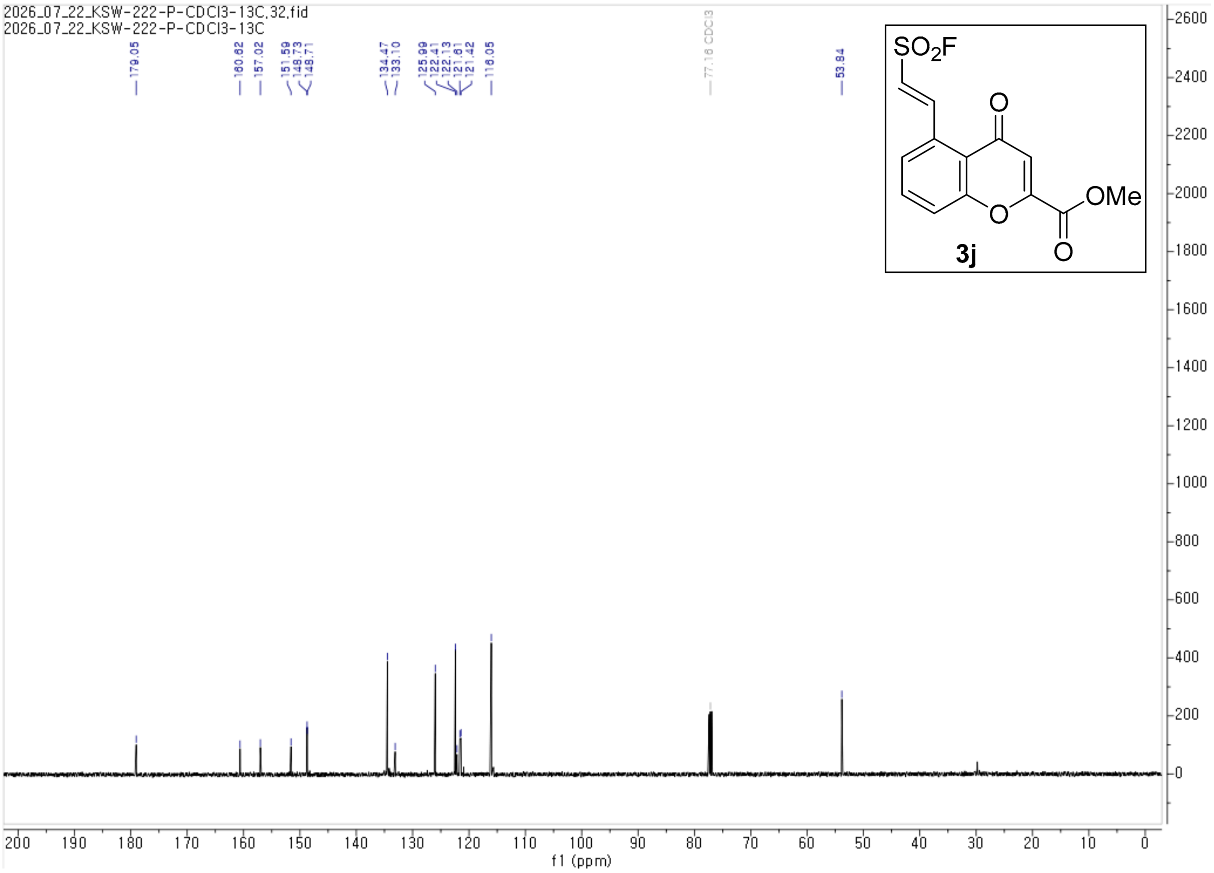
2026_07_22_KSW-222-P-CDCl3-13C.32.fid
2026_07_22_KSW-222-P-CDCl3-13C
179.05
160.82
157.02
151.59
148.73
148.71
134.47
133.10
125.99
122.41
122.13
121.61
121.42
116.05
77.16 CDCl3
53.84
SO2F
O
OMe
O
O
3j
f1 (ppm)

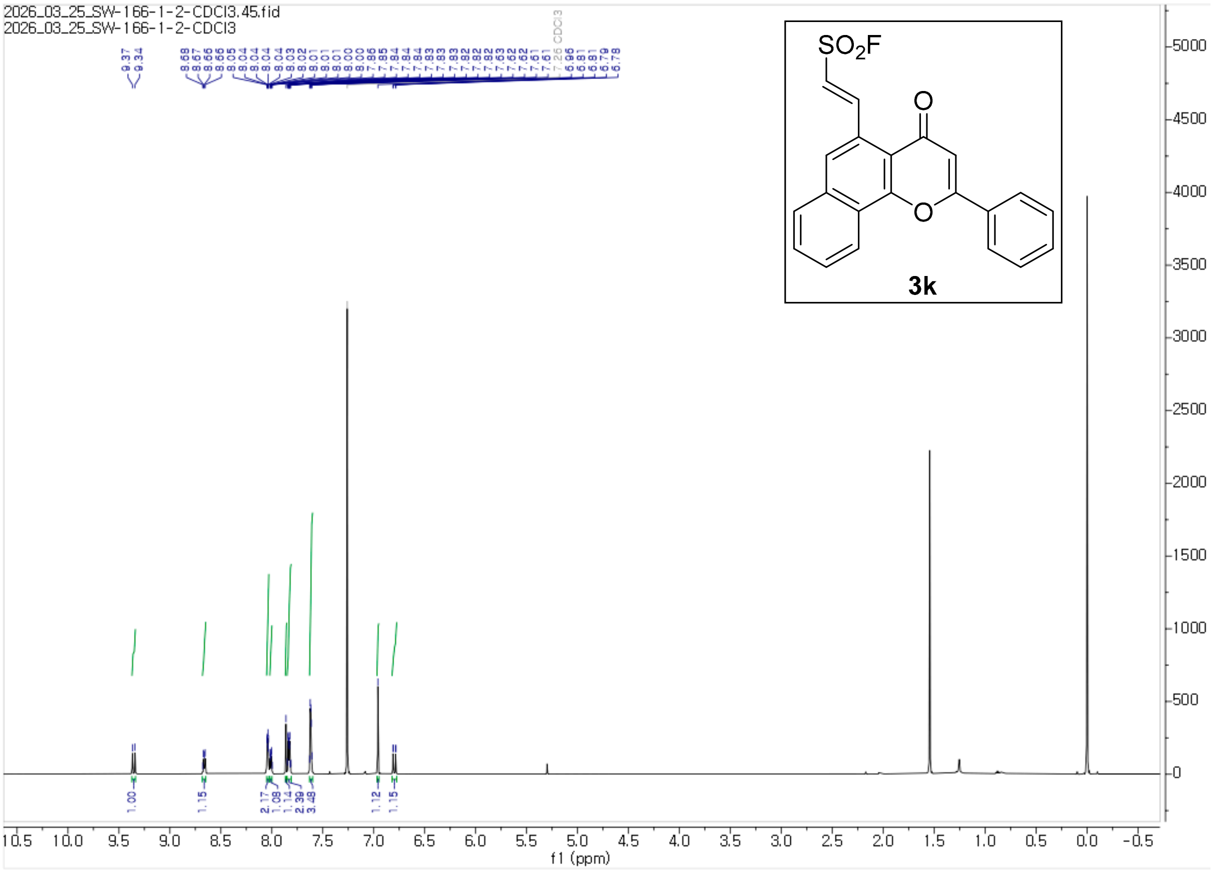
2026_03_25_SW-166-1-2-CDCl3.45.fid
2026_03_25_SW-166-1-2-CDCl3
SO2F
O
O
3k
f1 (ppm)

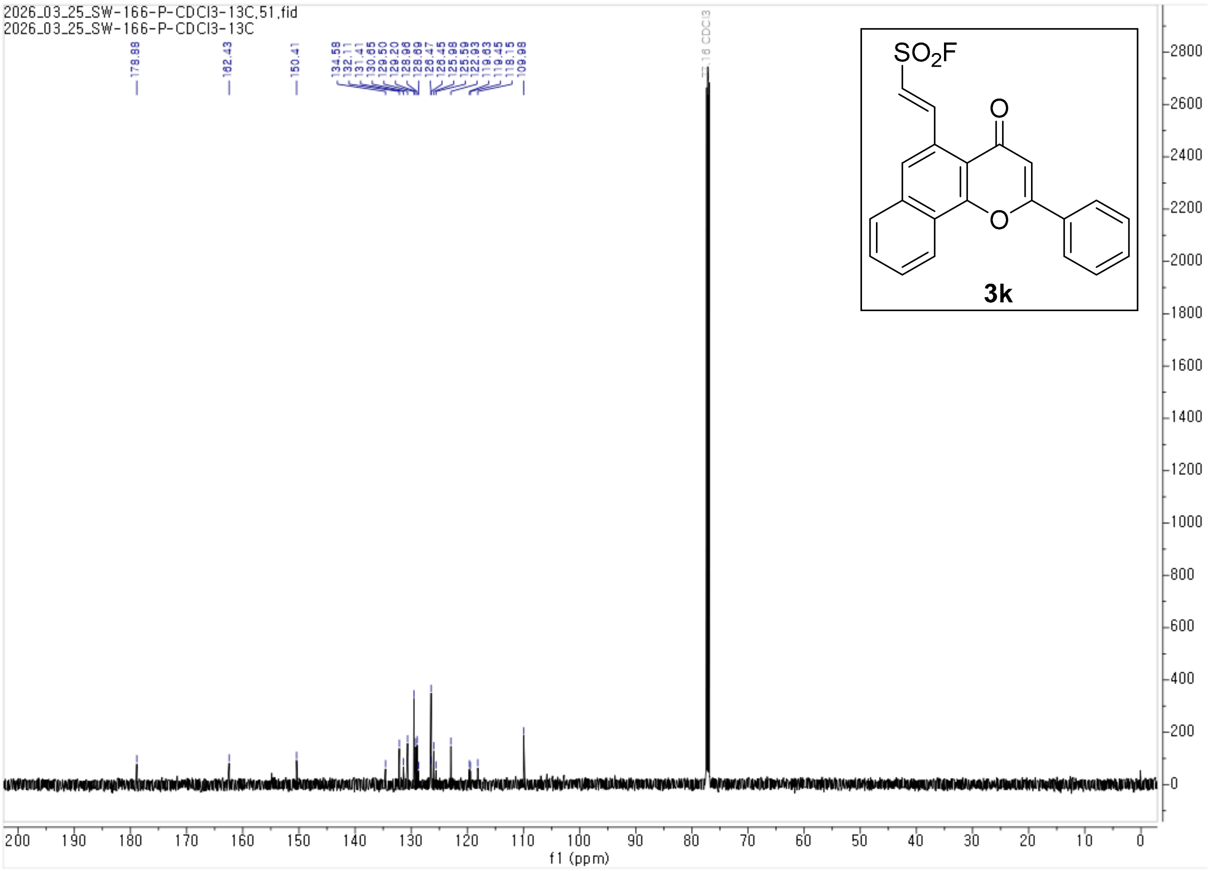

2026_03_25_SW-166-P-CDCl3-13C,51,fid
2026_03_25_SW-166-P-CDCl3-13C
178.88
162.43
150.41
134.58
132.11
131.41
130.65
129.50
129.20
128.98
128.69
126.47
126.45
125.98
125.59
122.93
119.63
119.45
118.15
109.98
77.16 CDCl3
SO2F
O
O
3k
f1 (ppm)

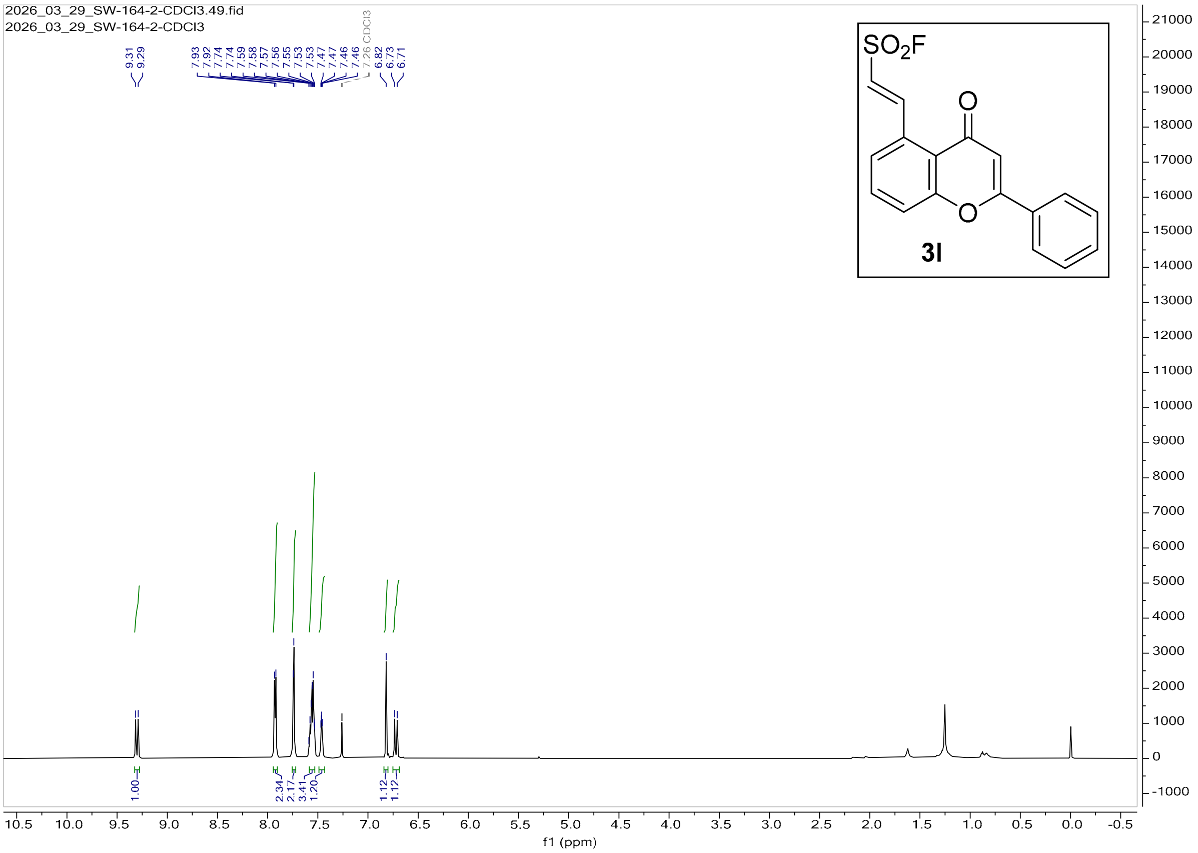

2026_03_29_SW-164-2-CDCl3.49.fid
2026_03_29_SW-164-2-CDCl3
SO2F
O
O
3l
f1 (ppm)

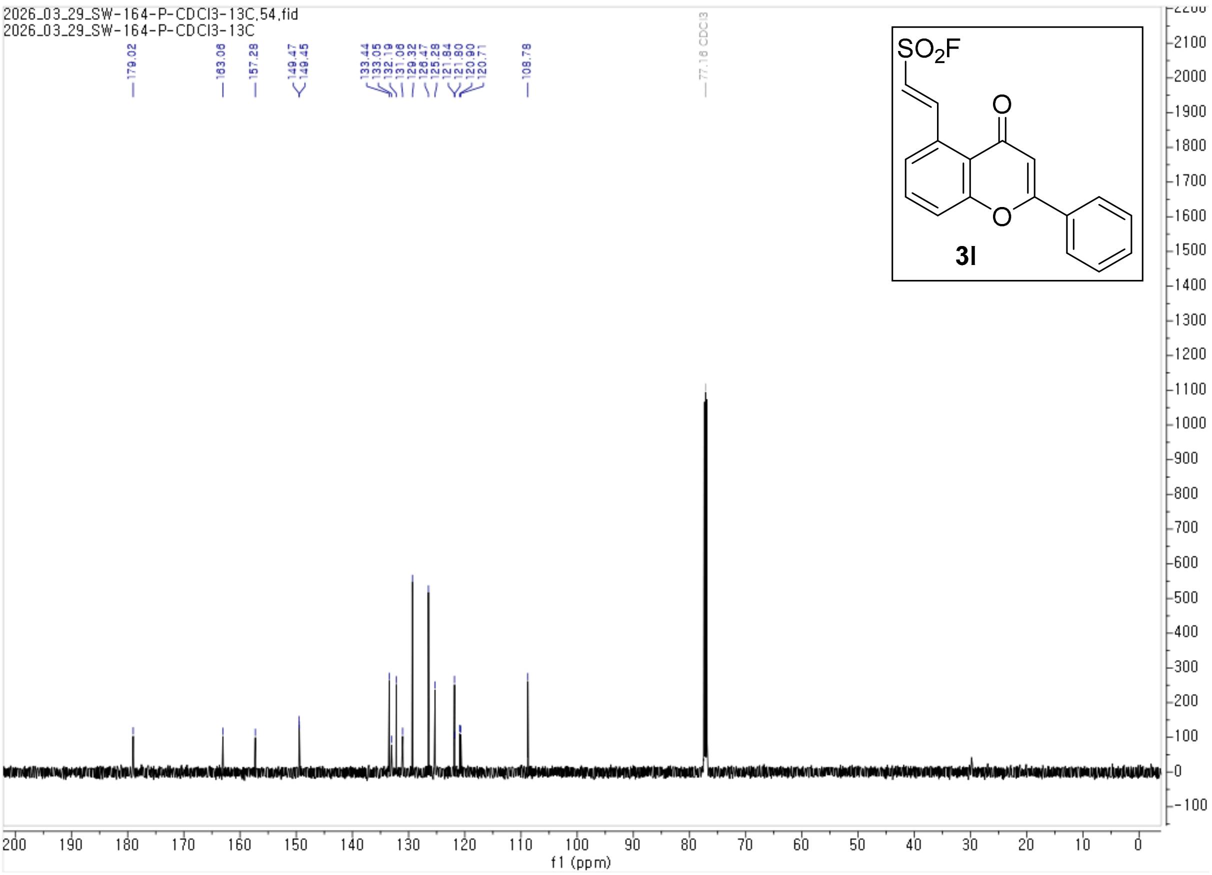
2026_03_29_SW-164-P-CDCl3-13C,54,fid
2026_03_29_SW-164-P-CDCl3-13C
179.02
163.08
157.28
149.47
149.45
133.44
133.05
132.19
131.08
129.32
126.47
125.28
121.84
121.80
120.90
120.71
108.78
77.16 CDCl3
SO2F
O
O
3l
f1 (ppm)

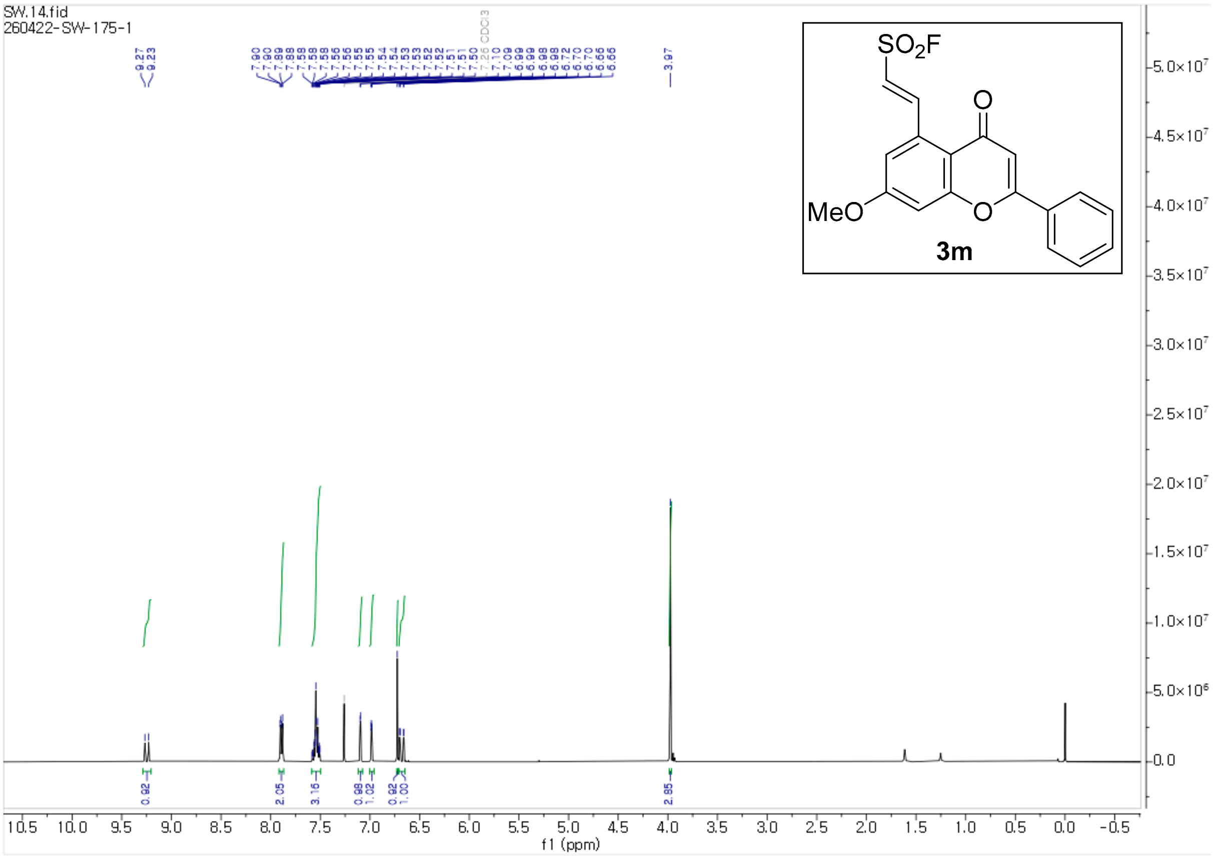

SW.14.fid
260422-SW-175-1
SO2F
O
MeO
O
3m
f1 (ppm)

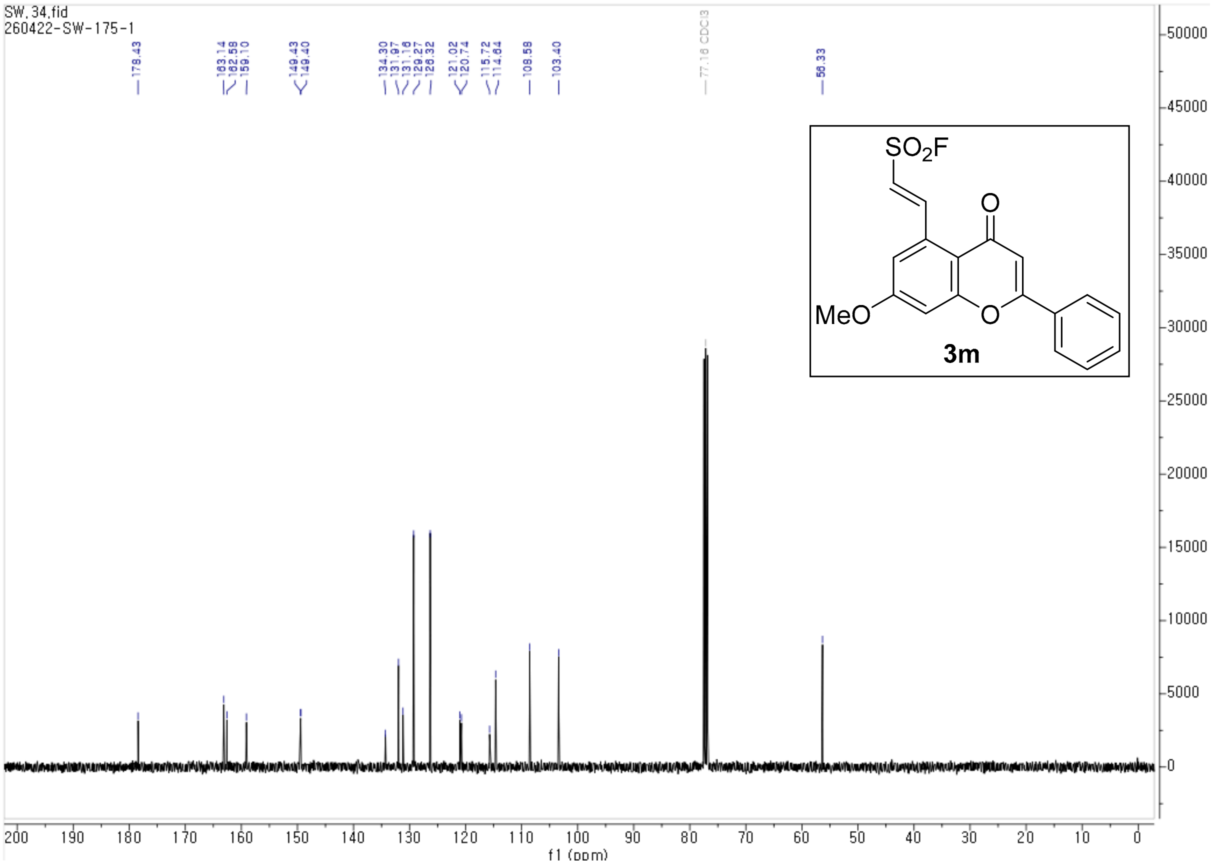
SW,34,fid
260422-SW-175-1
178.43
163.14
162.58
159.10
149.43
149.40
134.30
131.97
131.16
129.27
126.32
121.02
120.74
115.72
114.64
108.58
103.40
77.16 CDCl3
56.33
SO2F
O
MeO
O
3m
f1 (ppm)

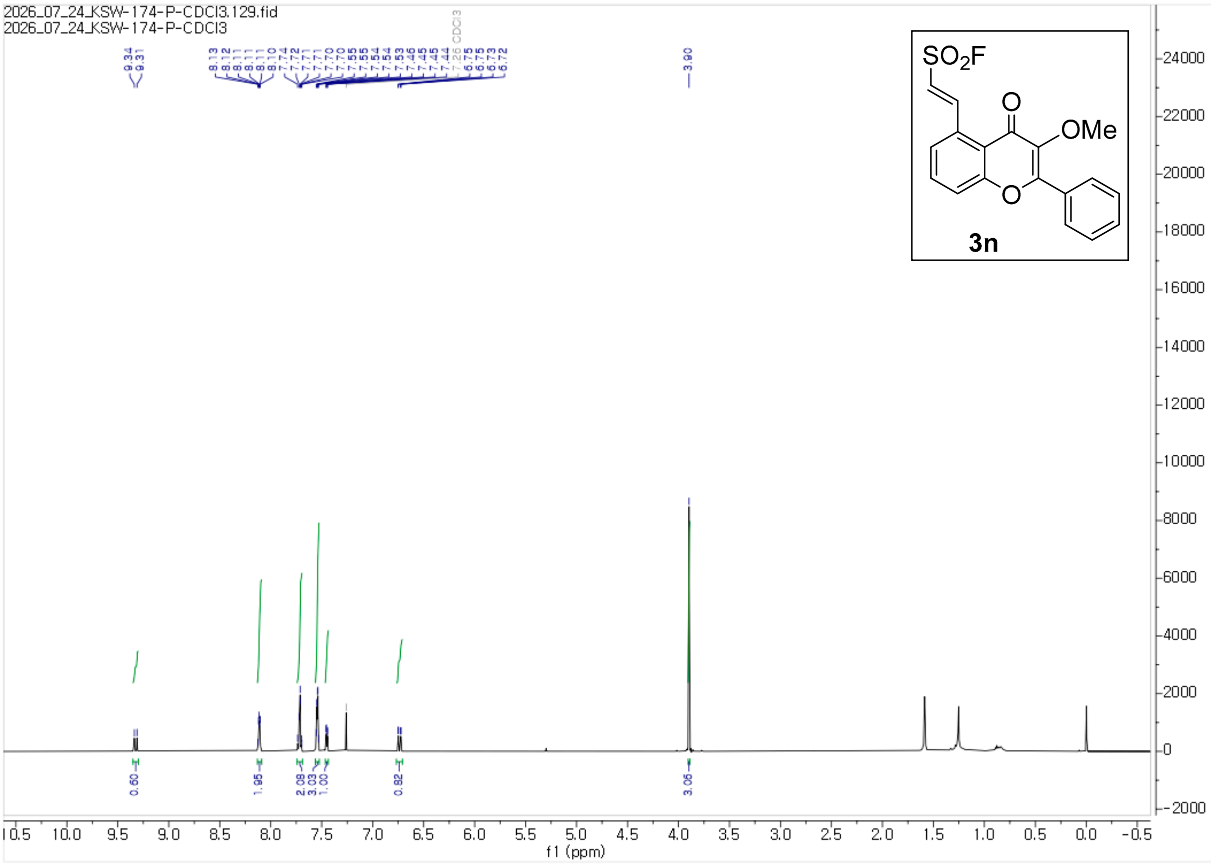
2026_07_24_KSW-174-P-CDCl3.129.fid
2026_07_24_KSW-174-P-CDCl3
SO2F
O
OMe
O
3n
f1 (ppm)

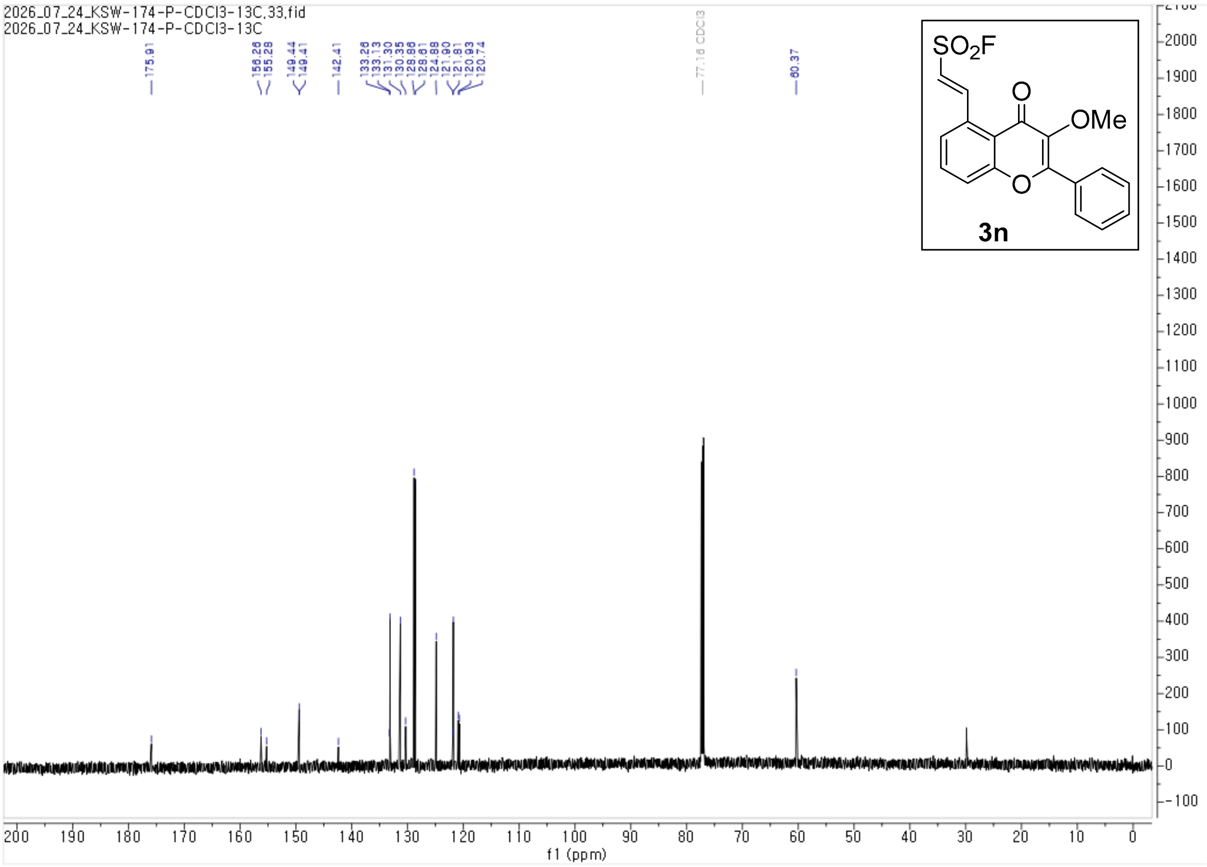

2026_07_24_KSW-174-P-CDCl3-13C.33.fid
2026_07_24_KSW-174-P-CDCl3-13C
175.91
156.26
155.29
149.44
149.41
142.41
133.26
133.13
131.30
130.35
128.86
128.81
124.88
121.90
121.81
120.93
120.74
77.16 CDCl3
60.37
SO2F
O
OMe
O
3n
f1 (ppm)

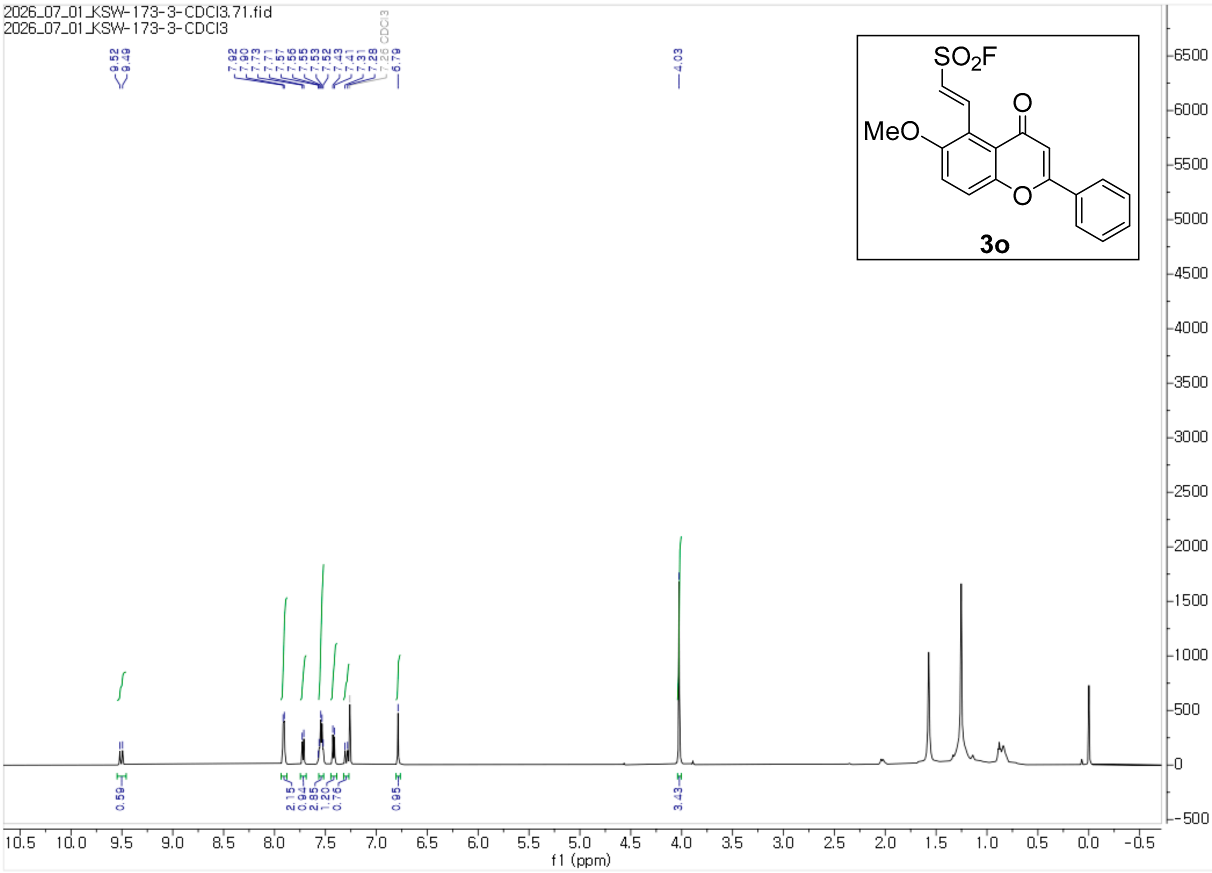
2026_07_01_KSW-173-3-CDCl3.71.fid
2026_07_01_KSW-173-3-CDCl3
9.52
9.49
7.92
7.90
7.73
7.71
7.57
7.56
7.55
7.53
7.52
7.43
7.41
7.31
7.28
7.26 CDCl3
6.79
4.03
0.59
2.15
0.94
2.85
1.20
0.76
0.95
3.43
f1 (ppm)
SO2F
O
MeO
O
3o

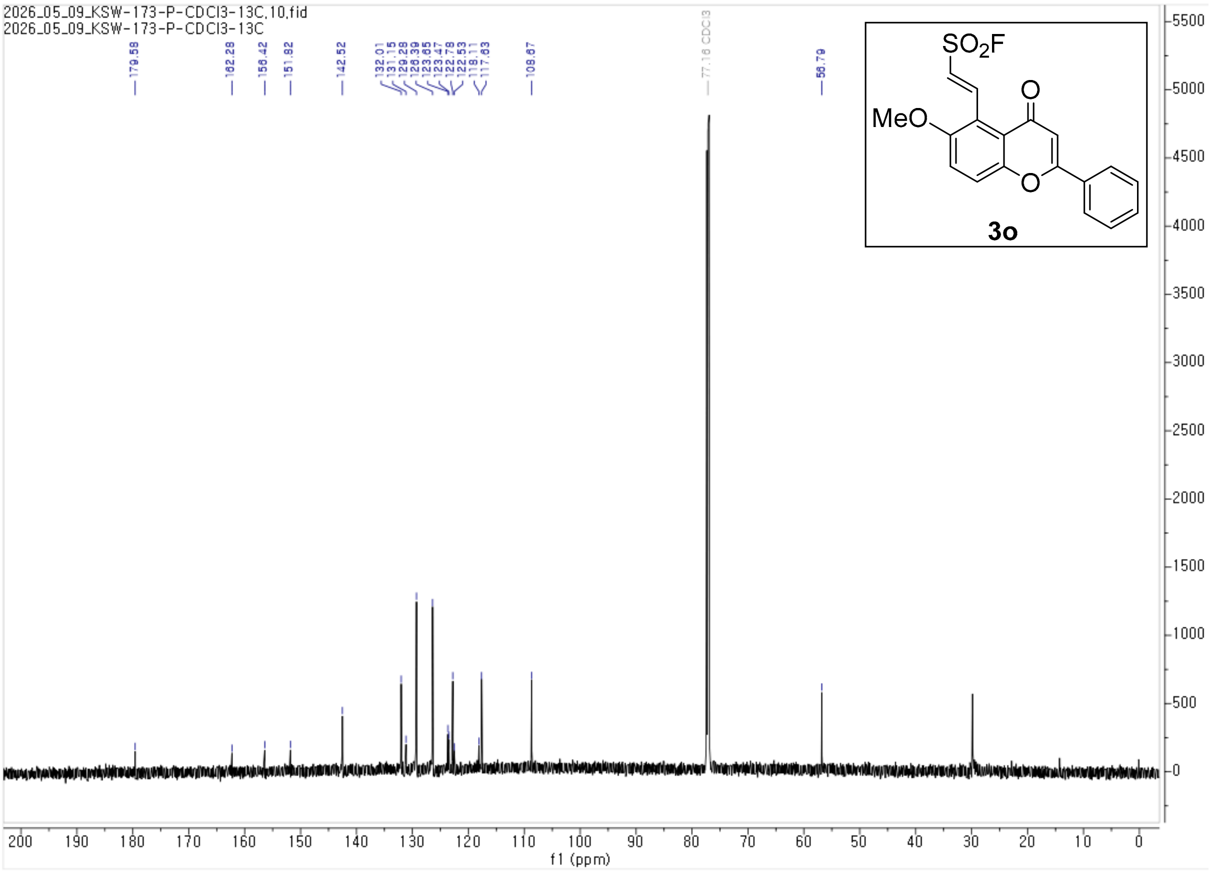
2026_05_09_KSW-173-P-CDCl3-13C,10,fid
2026_05_09_KSW-173-P-CDCl3-13C
179.58
162.28
156.42
151.82
142.52
132.01
131.15
129.28
126.39
123.65
123.47
122.78
122.53
118.11
117.83
108.87
77.16 CDCl3
56.79
SO2F
O
MeO
O
3o
f1 (ppm)
200 190 180 170 160 150 140 130 120 110 100 90 80 70 60 50 40 30 20 10 0
5500 5000 4500 4000 3500 3000 2500 2000 1500 1000 500 0

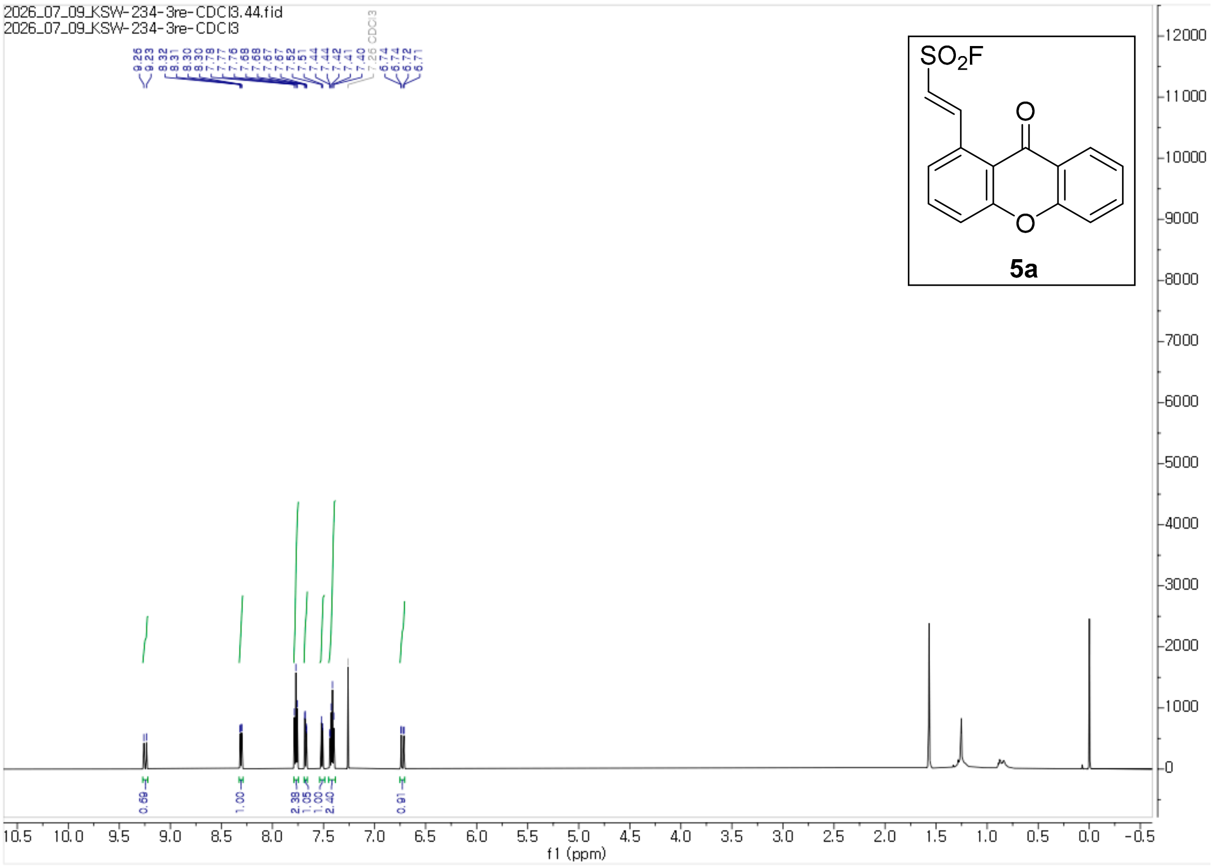

2026_07_09_KSW-234-3re-CDCl3.44.fid
2026_07_09_KSW-234-3re-CDCl3
SO2F
O
O
5a
f1 (ppm)

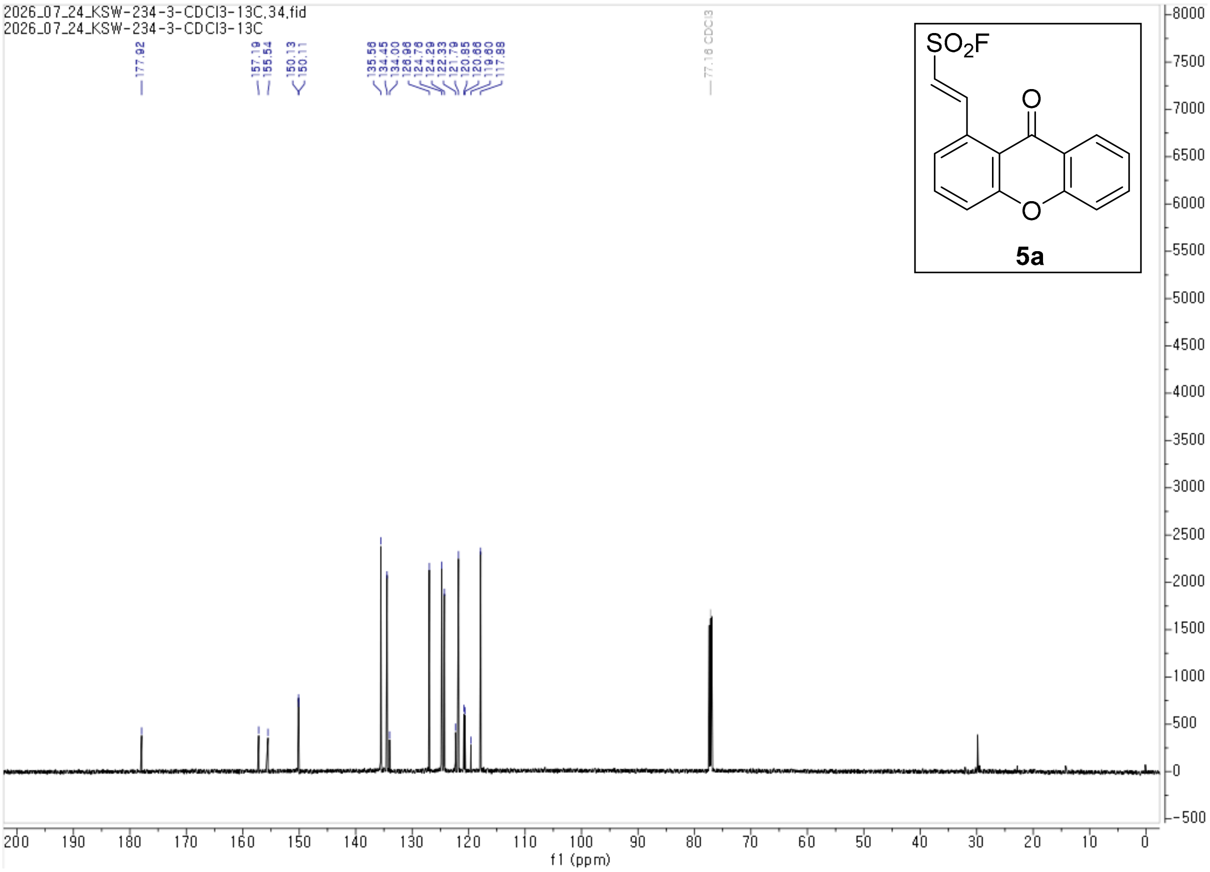
2026_07_24_KSW-234-3-CDCl3-13C,34,fid
2026_07_24_KSW-234-3-CDCl3-13C
177.92
157.19
155.54
150.13
150.11
135.58
134.45
134.00
126.98
124.78
124.29
122.33
121.79
120.85
120.66
119.60
117.88
77.16 CDCl3
SO_2F
O
O
5a
8000
7500
7000
6500
6000
5500
5000
4500
4000
3500
3000
2500
2000
1500
1000
500
0
-500
200 190 180 170 160 150 140 130 120 110 100 90 80 70 60 50 40 30 20 10 0
f1 (ppm)

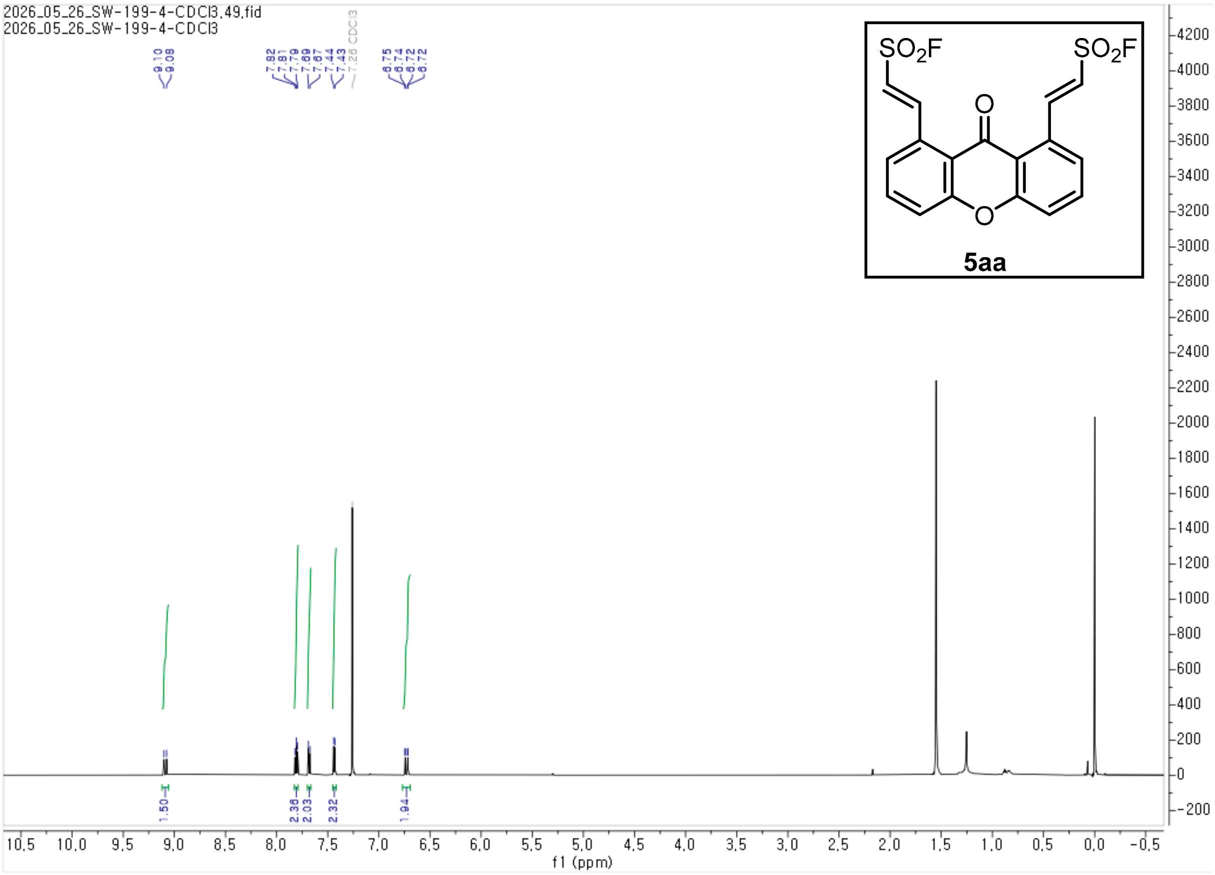
2026_05_26_SW-199-4-CDCl3.49.fid
2026_05_26_SW-199-4-CDCl3
9.10
9.08
7.82
7.81
7.79
7.69
7.67
7.44
7.43
7.26 CDCl3
6.75
6.74
6.72
6.72
1.50
2.38
2.03
2.32
1.94
f1 (ppm)
SO2F
SO2F
O
O
5aa

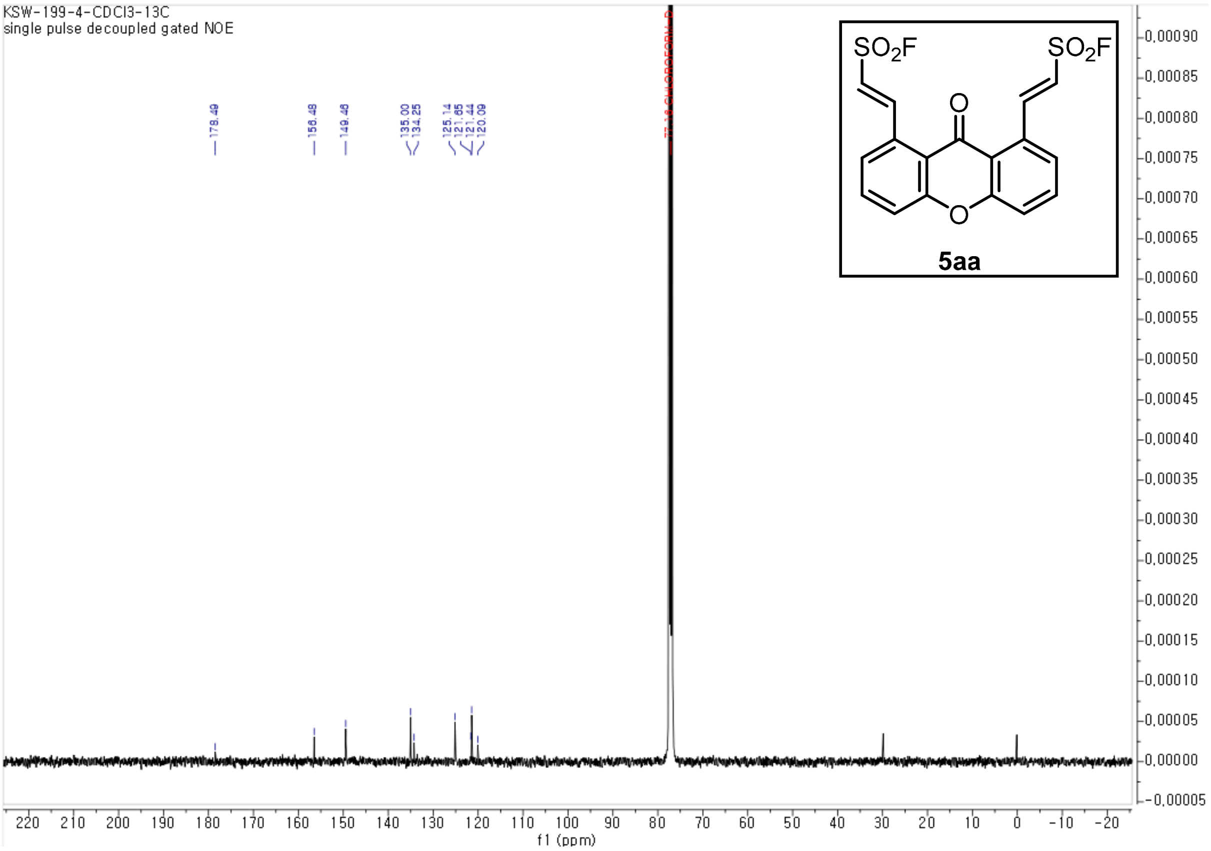
KSW-199-4-CDCl3-13C
single pulse decoupled gated NOE
178.49
156.48
149.48
135.00
134.25
125.14
121.65
121.44
120.09
SO2F
SO2F
O
O
5aa
f1 (ppm)

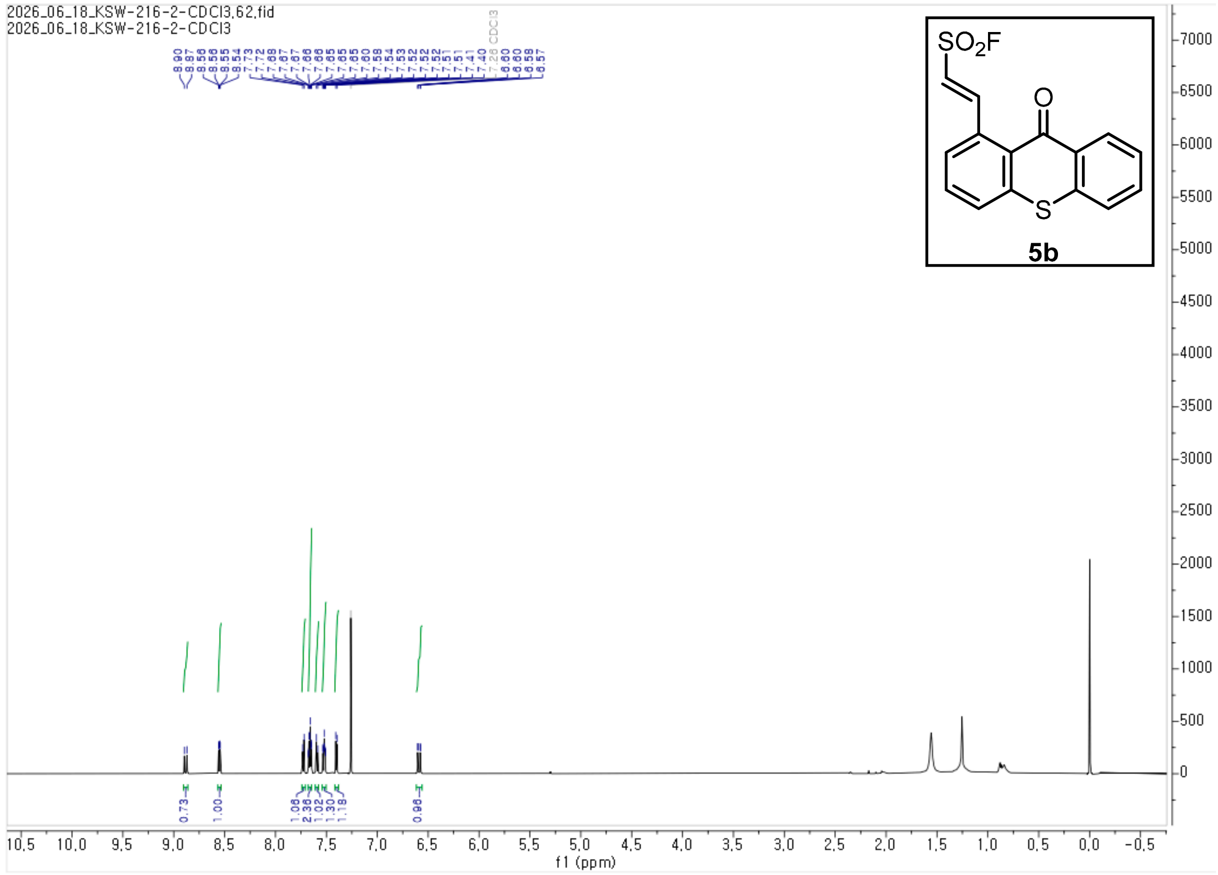
2026_06_18_KSW-216-2-CDCl3.62.fid
2026_06_18_KSW-216-2-CDCl3
SO2F
O
S
5b
f1 (ppm)

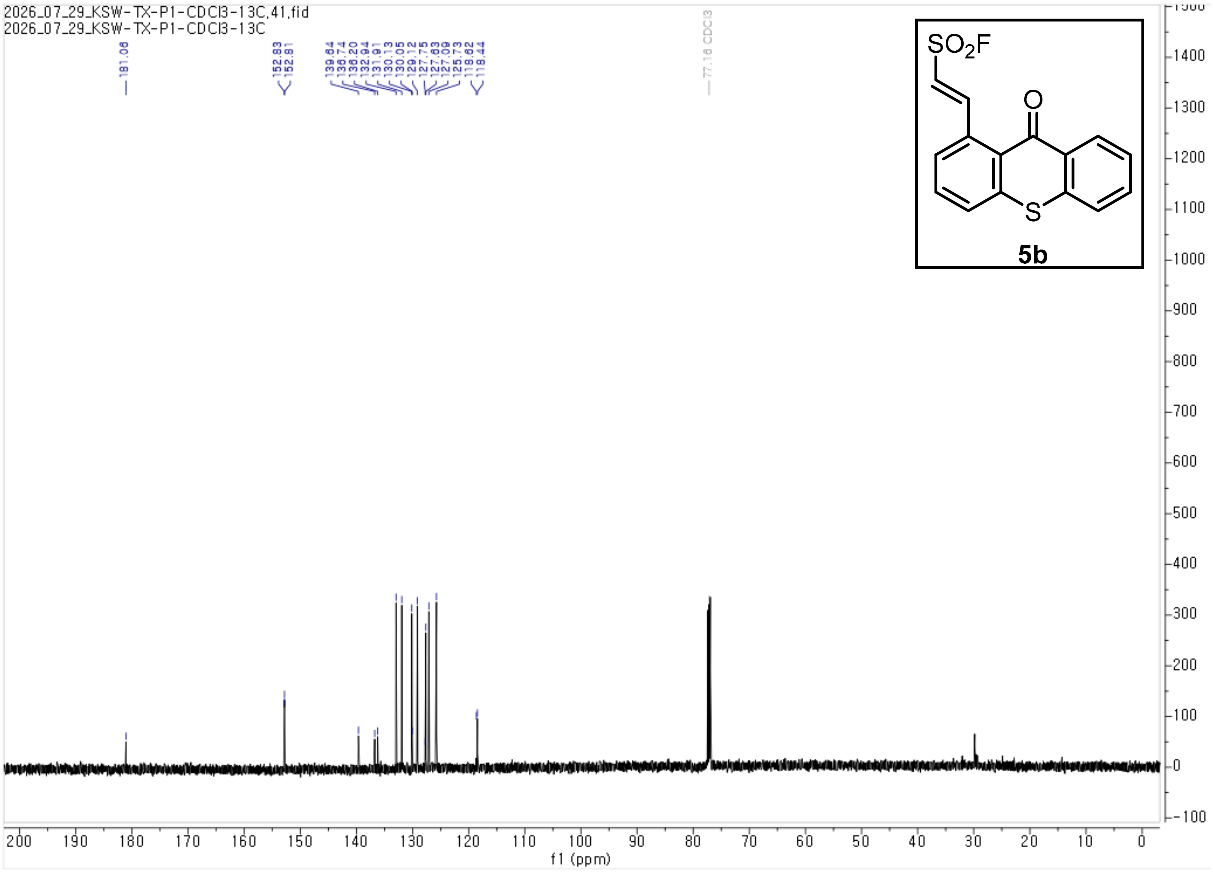
2026_07_29_KSW-TX-P1-CDCl3-13C,41,fid
2026_07_29_KSW-TX-P1-CDCl3-13C
181.08
152.83
152.81
139.64
136.74
136.20
132.94
131.91
130.13
130.05
129.12
127.75
127.63
127.09
125.73
118.62
118.44
77.16 CDCl3
SO2F
O
S
5b
f1 (ppm)

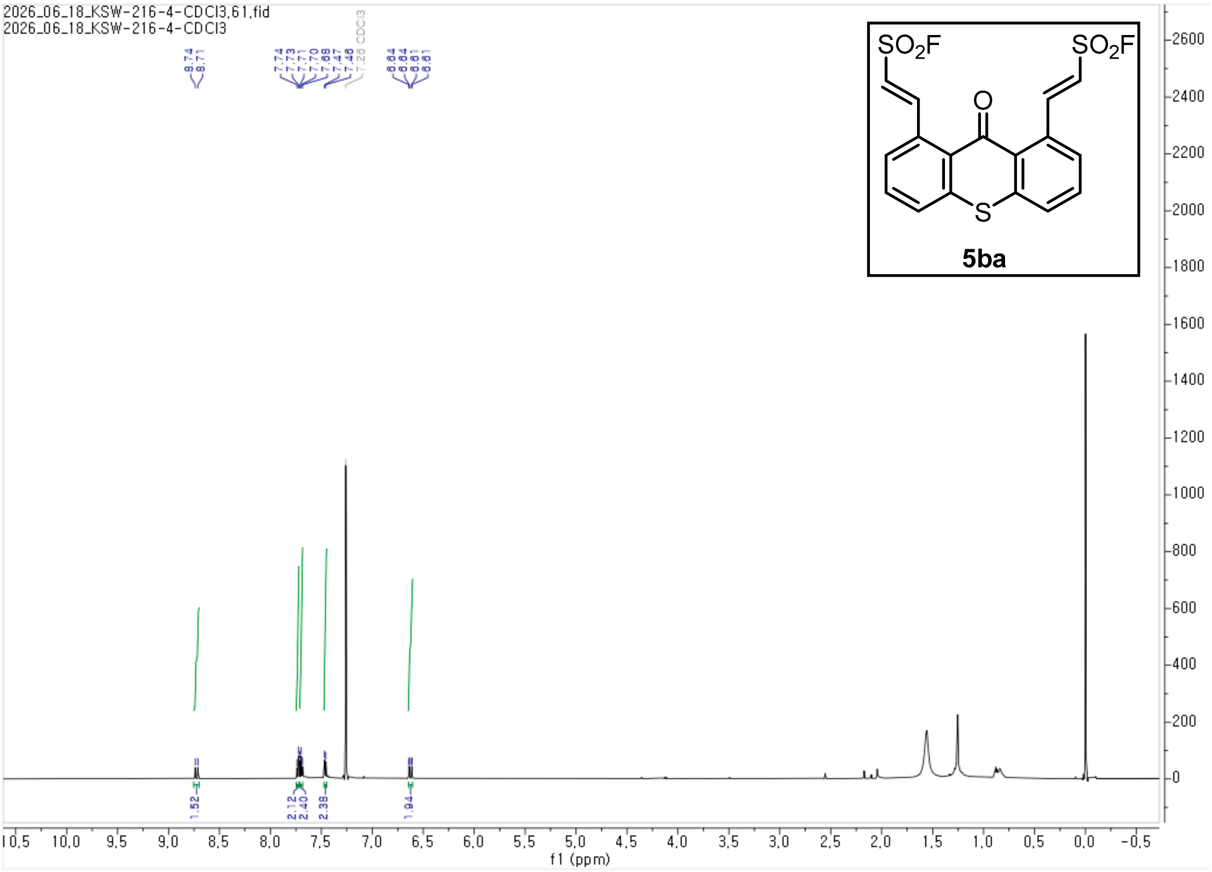
2026_06_18_KSW-216-4-CDCl3,61,fid
2026_06_18_KSW-216-4-CDCl3
8.74
8.71
7.74
7.73
7.71
7.70
7.69
7.47
7.46
7.26 CDCl3
6.64
6.64
6.61
6.61
1.52
2.12
2.40
2.38
1.94
SO2F
SO2F
O
S
5ba
f1 (ppm)

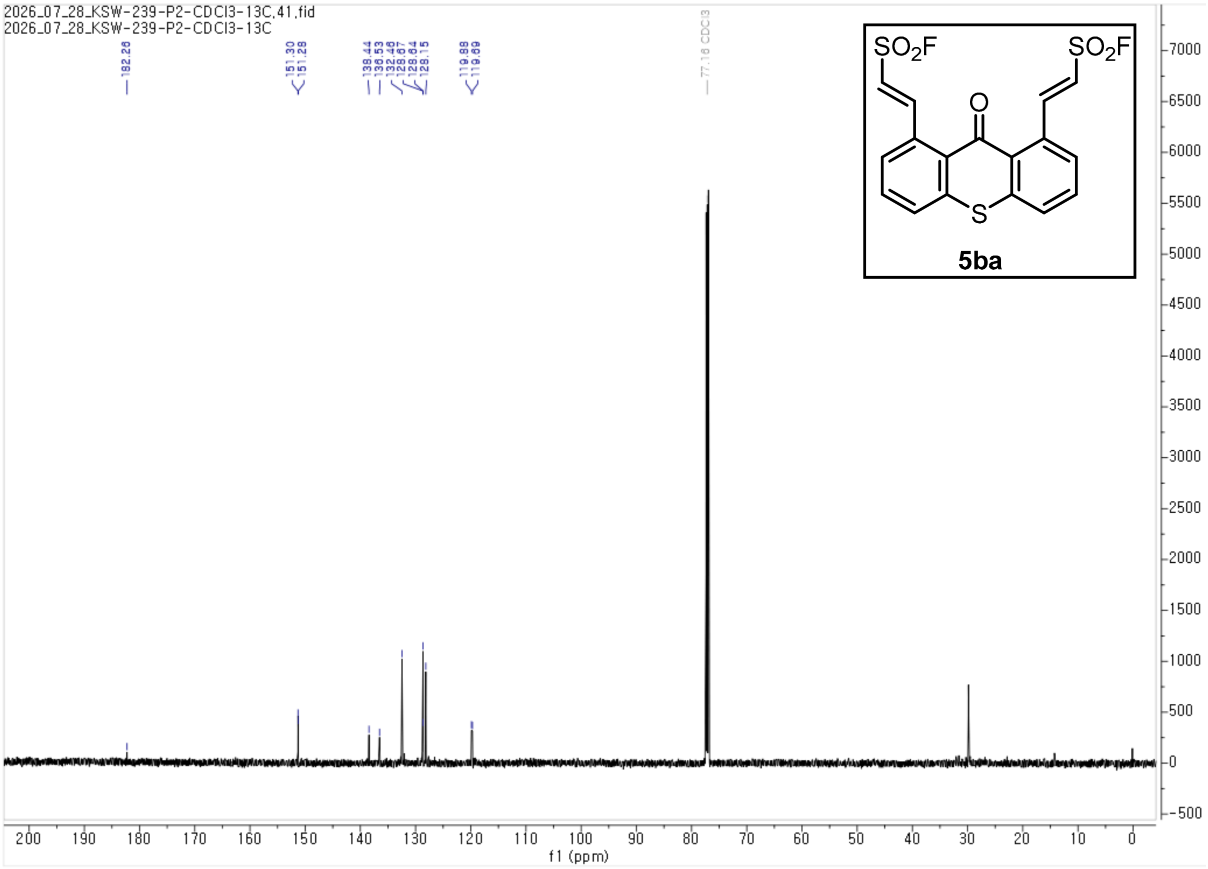
2026_07_28_KSW-239-P2-CDCl3-13C.41.fid
2026_07_28_KSW-239-P2-CDCl3-13C
182.26
151.30
151.28
138.44
136.53
132.46
128.67
128.64
128.15
119.89
119.69
77.16 CDCl3
SO2F
SO2F
O
S
5ba
f1 (ppm)

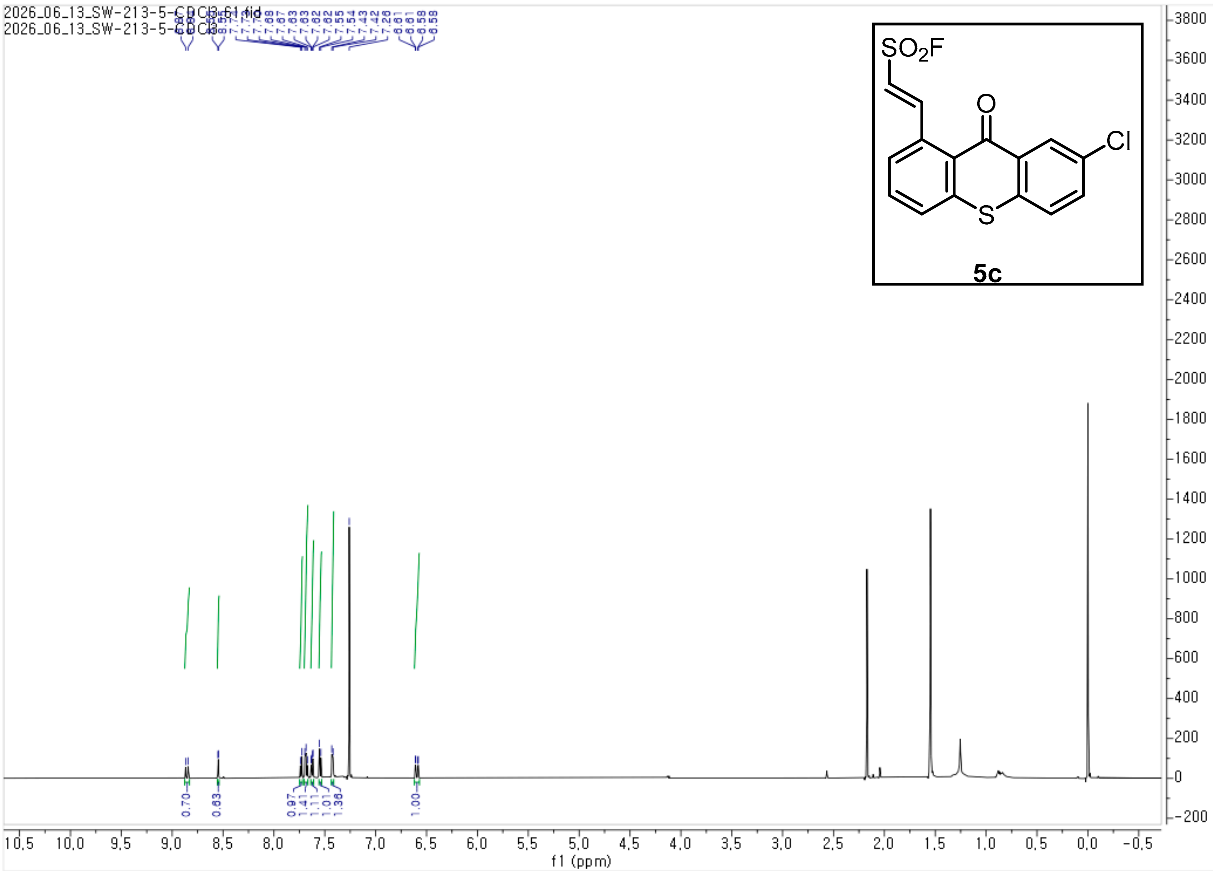
2026_06_13_SW-213-5-CDCl3
SO2F
O
Cl
S
5c
f1 (ppm)

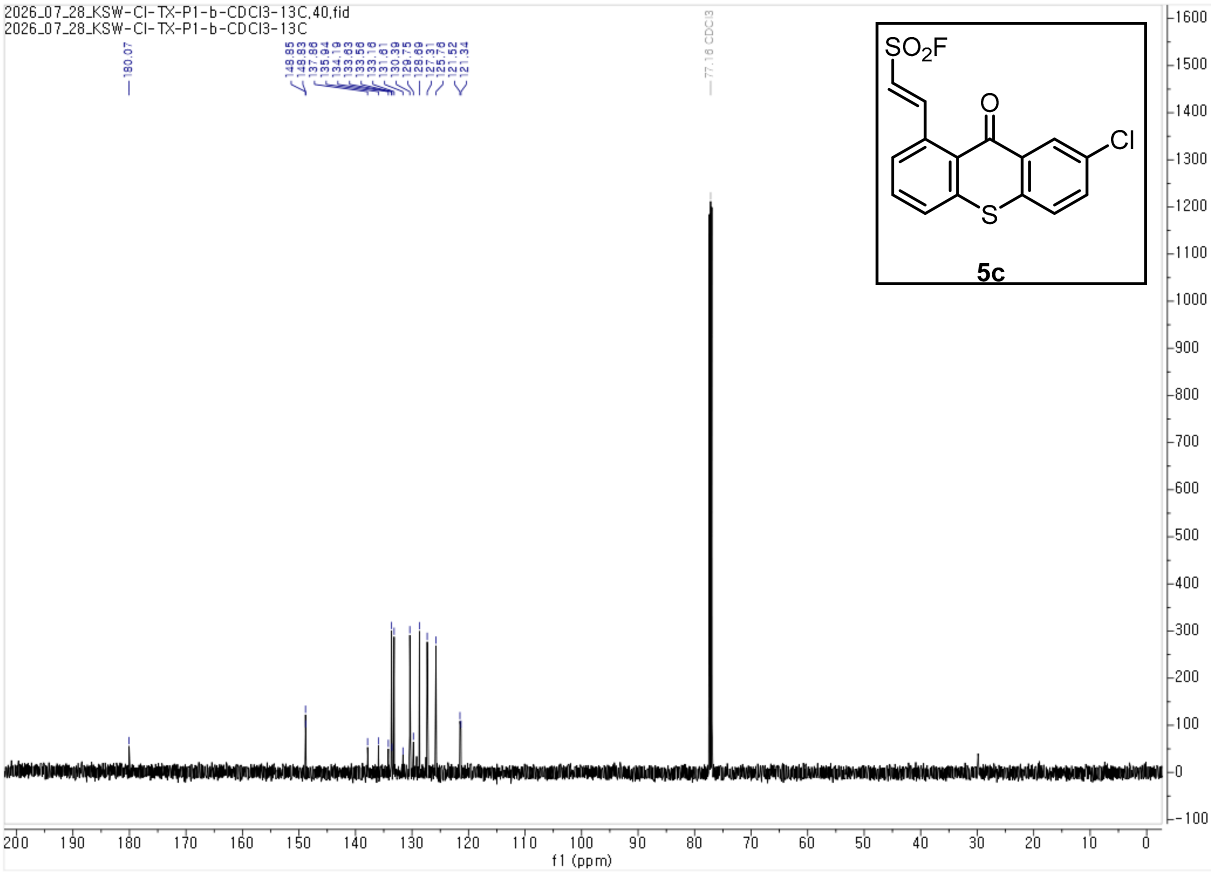
2026_07_28_KSW-Cl-TX-P1-b-CDCl3-13C.40.fid
2026_07_28_KSW-Cl-TX-P1-b-CDCl3-13C
180.07
148.85
148.83
137.88
135.94
134.19
133.63
133.56
133.18
131.81
130.39
129.75
128.89
127.31
125.78
121.52
121.34
77.16 CDCl3
SO2F
O
Cl
S
5c
f1 (ppm)

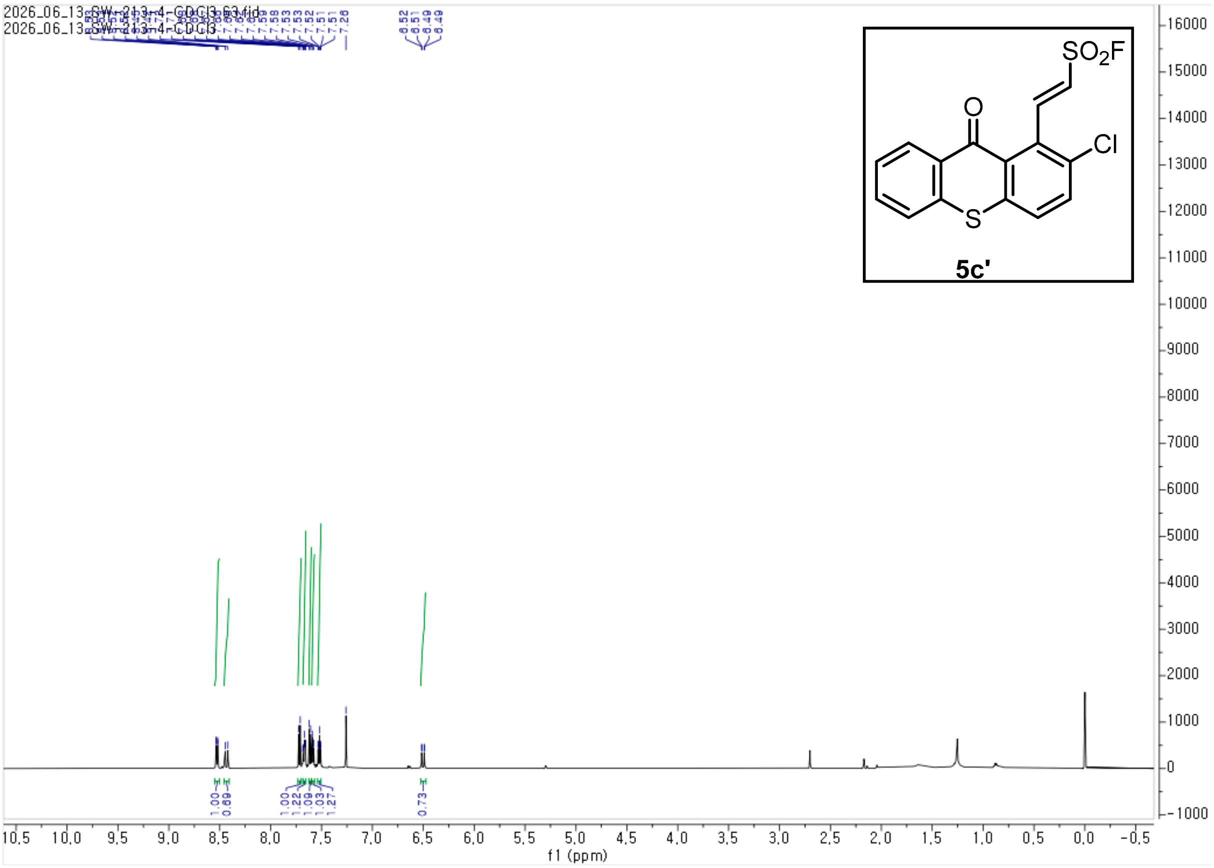
2026_06_13_SW-213-4-CDCl3
SO2F
O
Cl
S
5c'
f1 (ppm)

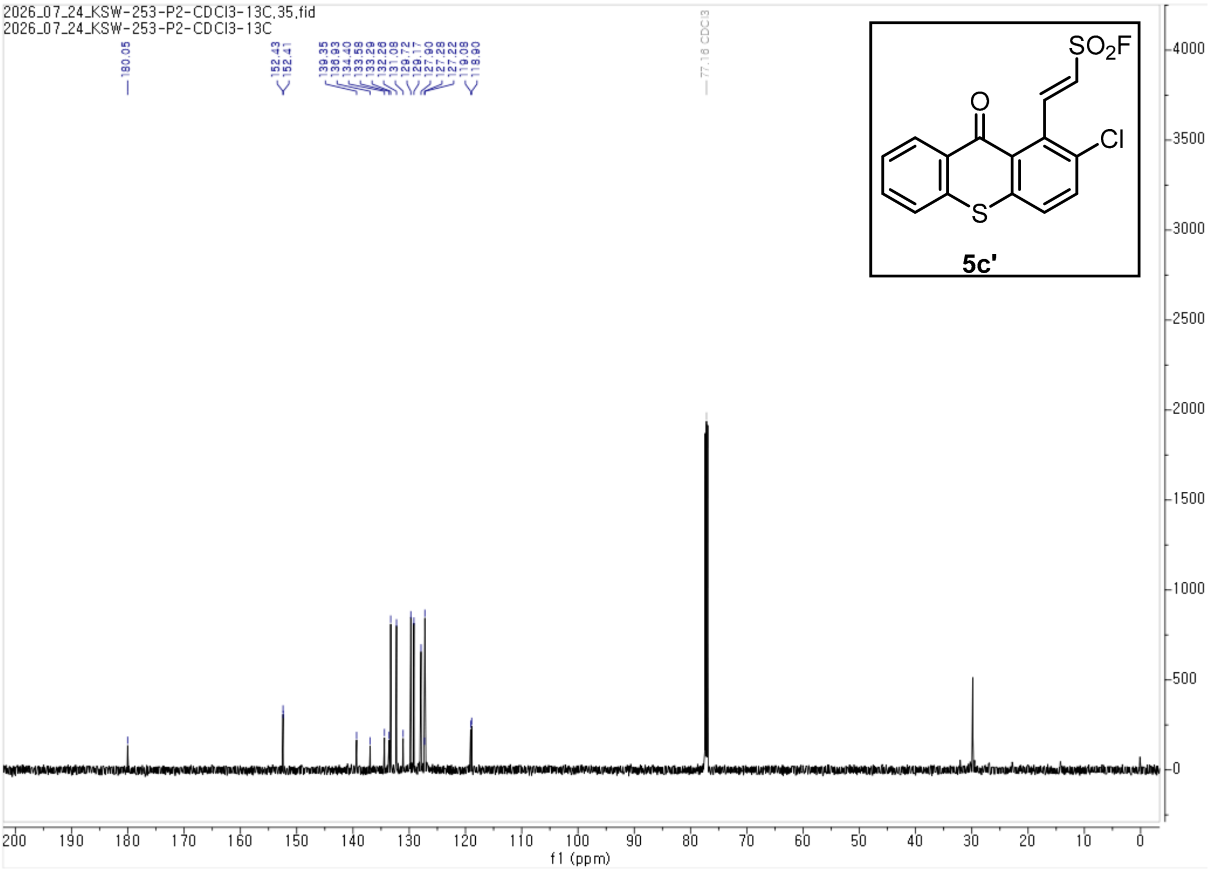
2026_07_24_KSW-253-P2-CDCl3-13C.35.fid
2026_07_24_KSW-253-P2-CDCl3-13C
180.05
152.43
152.41
139.35
136.93
134.40
133.58
133.29
132.28
131.08
129.72
129.17
127.90
127.28
127.22
119.08
118.90
77.16 CDCl3
SO2F
O
Cl
S
5c'
4000
3500
3000
2500
2000
1500
1000
500
0
200 190 180 170 160 150 140 130 120 110 100 90 80 70 60 50 40 30 20 10 0
f1 (ppm)

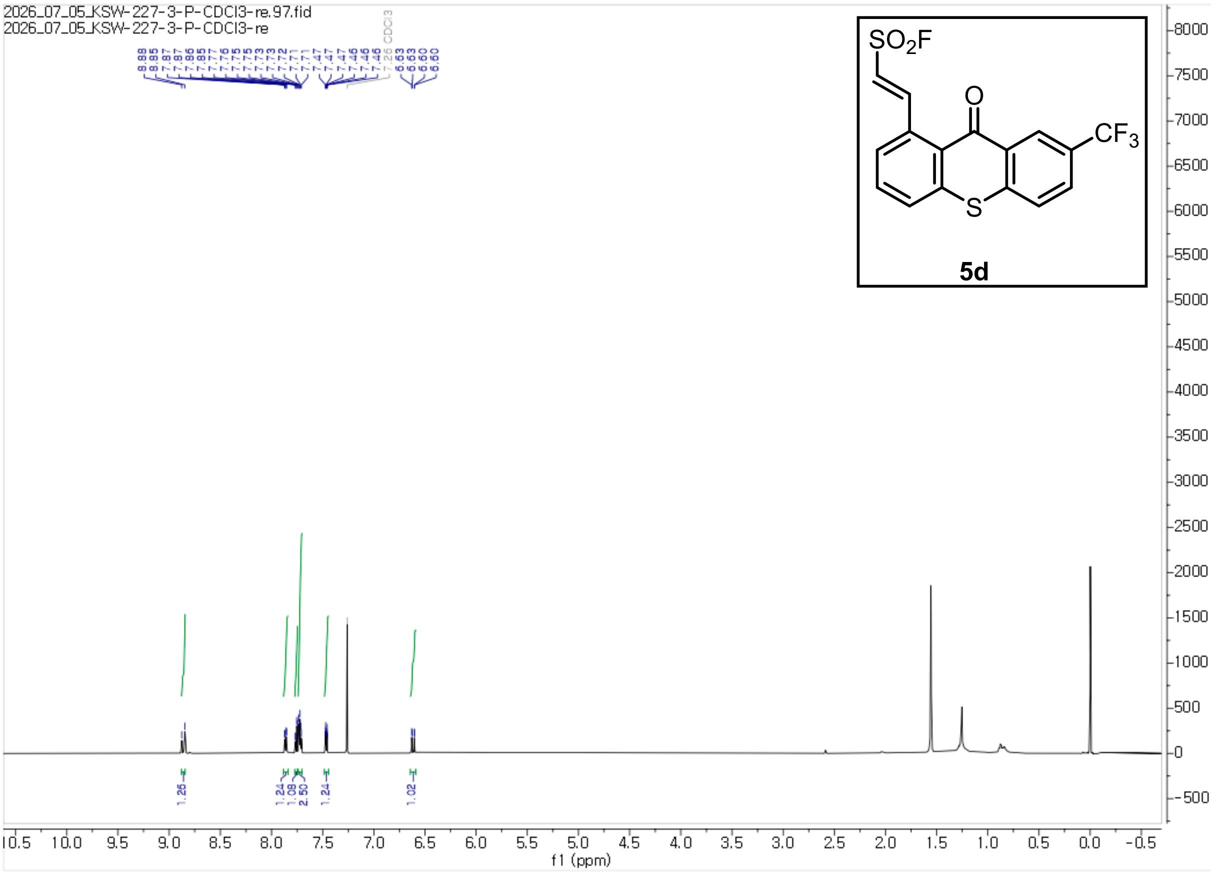
2026_07_05_KSW-227-3-P-CDCl3-re.97.fid
2026_07_05_KSW-227-3-P-CDCl3-re
SO2F
O
CF3
S
5d
f1 (ppm)

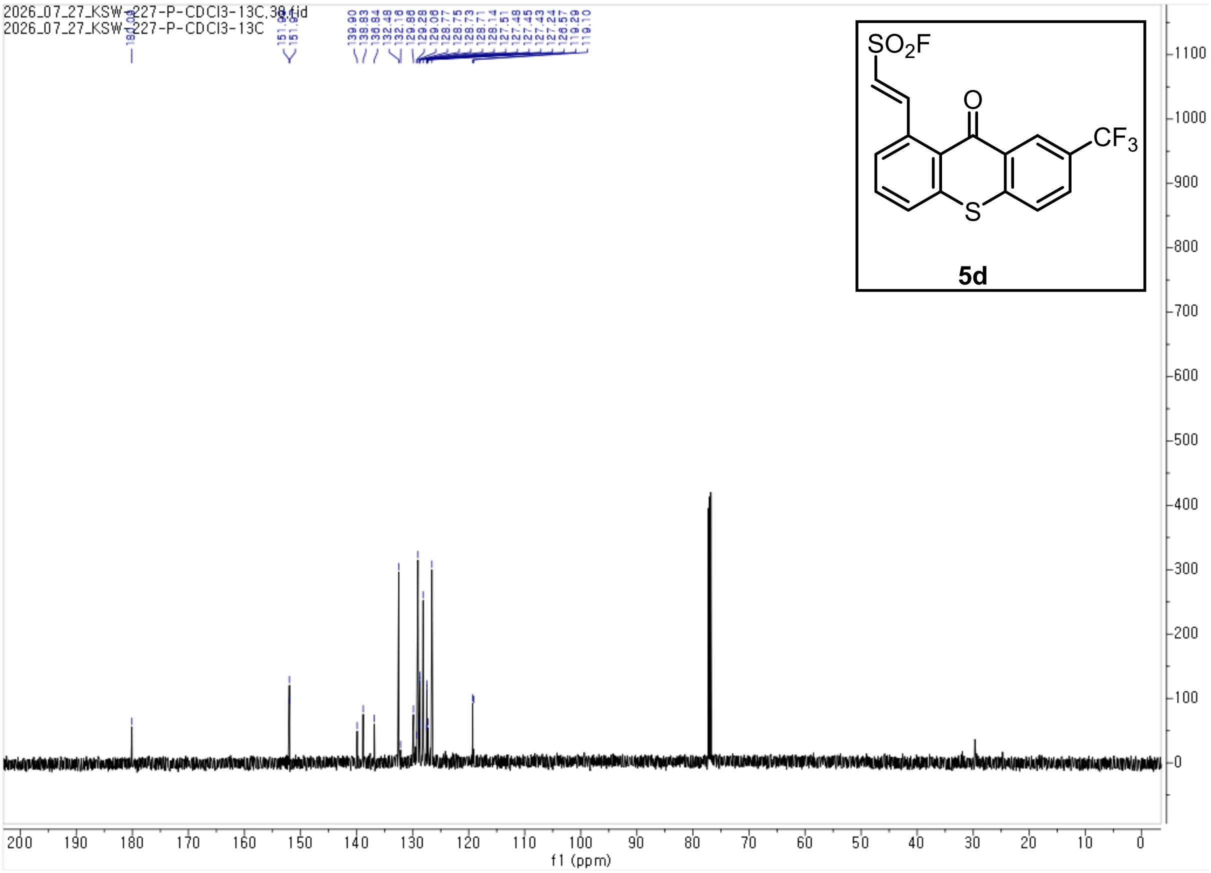

SO2F
O
CF3
S
5d
f1 (ppm)

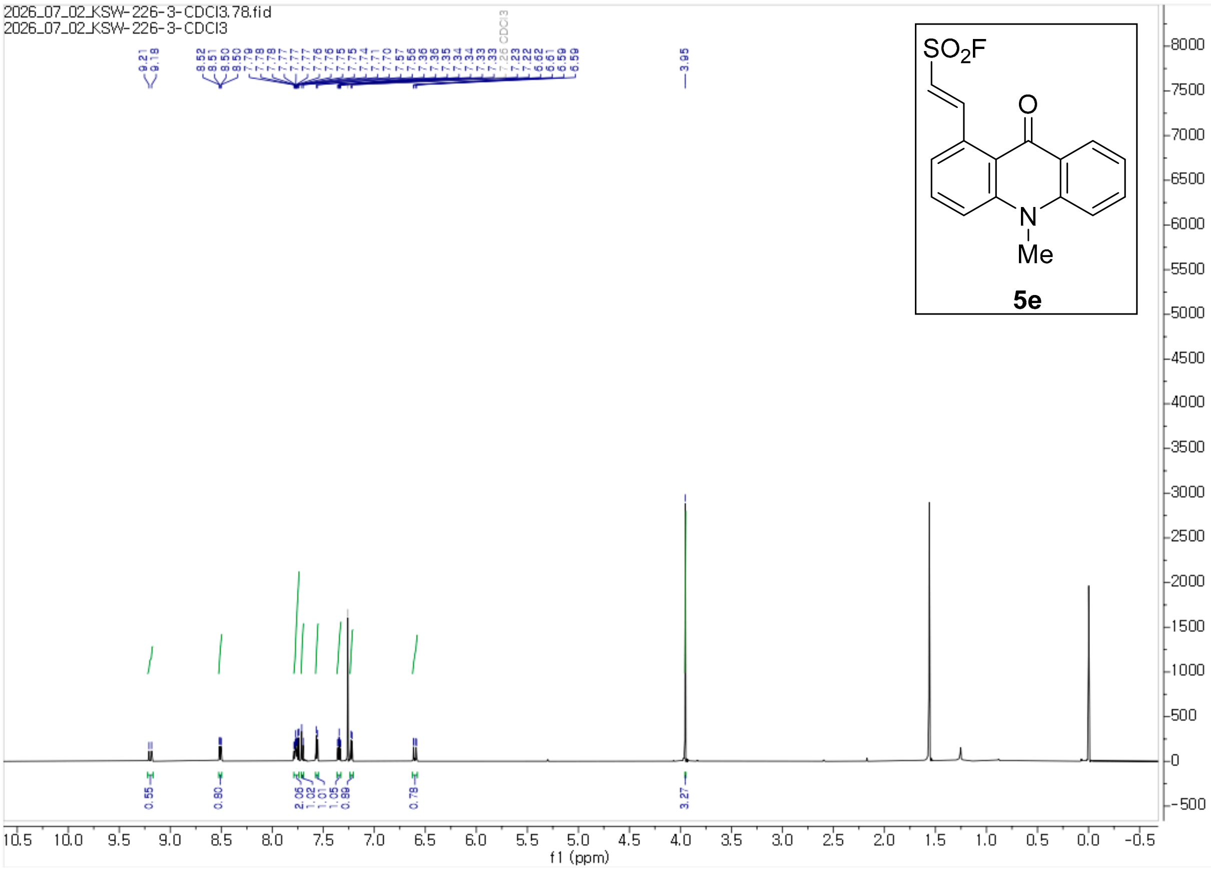

2026_07_02_KSW-226-3-CDCl3.78.fid
2026_07_02_KSW-226-3-CDCl3
SO2F
O
N
Me
5e
f1 (ppm)

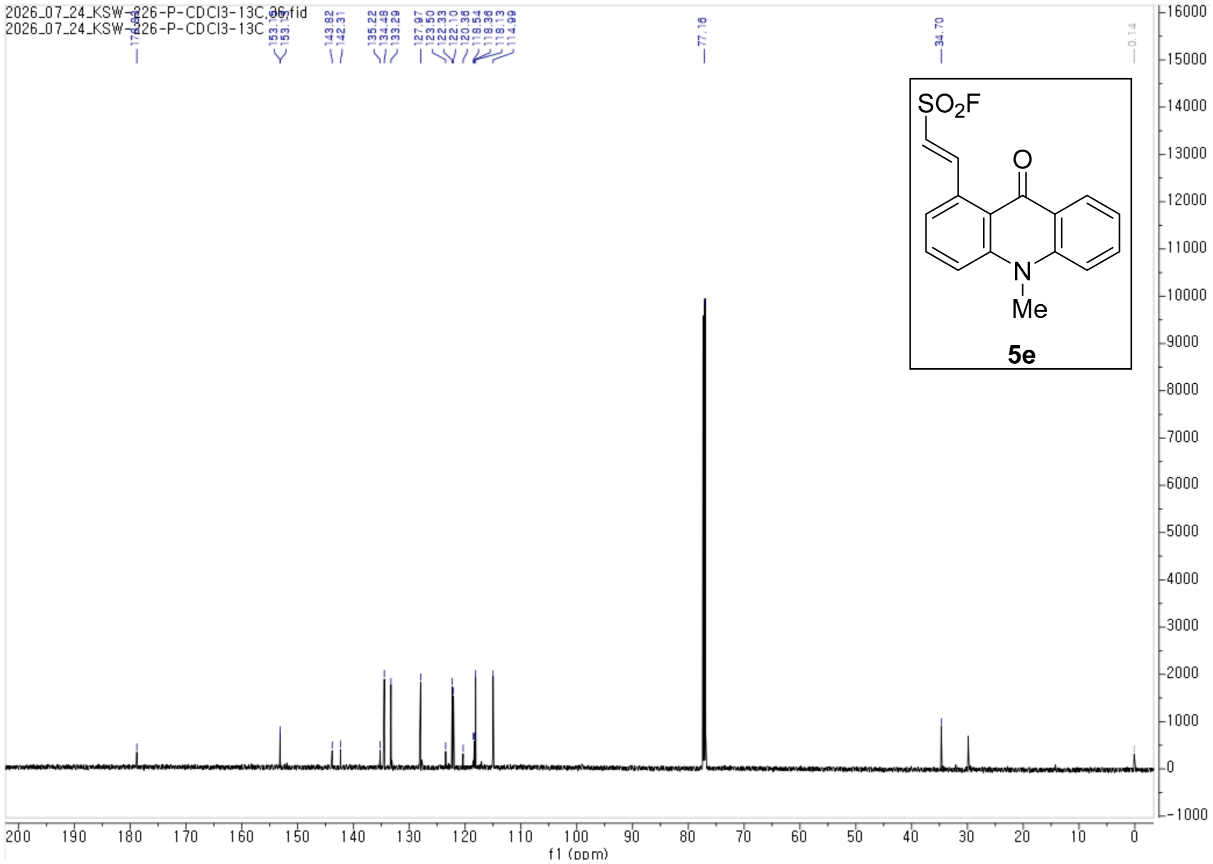
2026_07_24_KSW-226-P-CDCl3-13C.36.fid
2026_07_24_KSW-226-P-CDCl3-13C
143.82
142.31
135.22
134.48
133.29
127.97
123.50
122.33
122.10
120.38
118.54
118.36
118.13
114.99
77.16
34.70
0.14
SO2F
O
N
Me
5e
f1 (ppm)

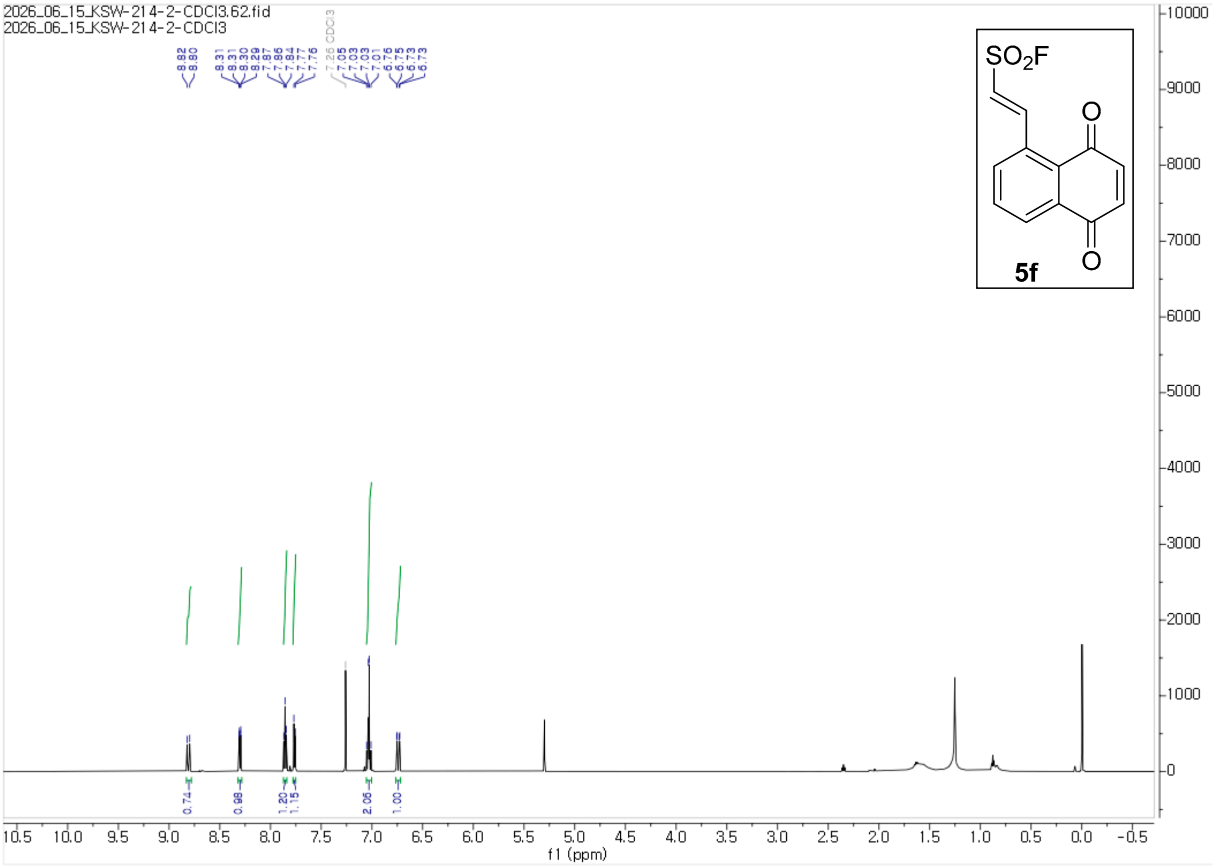
2026_06_15_KSW-214-2-CDCl3.62.fid
2026_06_15_KSW-214-2-CDCl3
SO2F
O
O
5f
f1 (ppm)

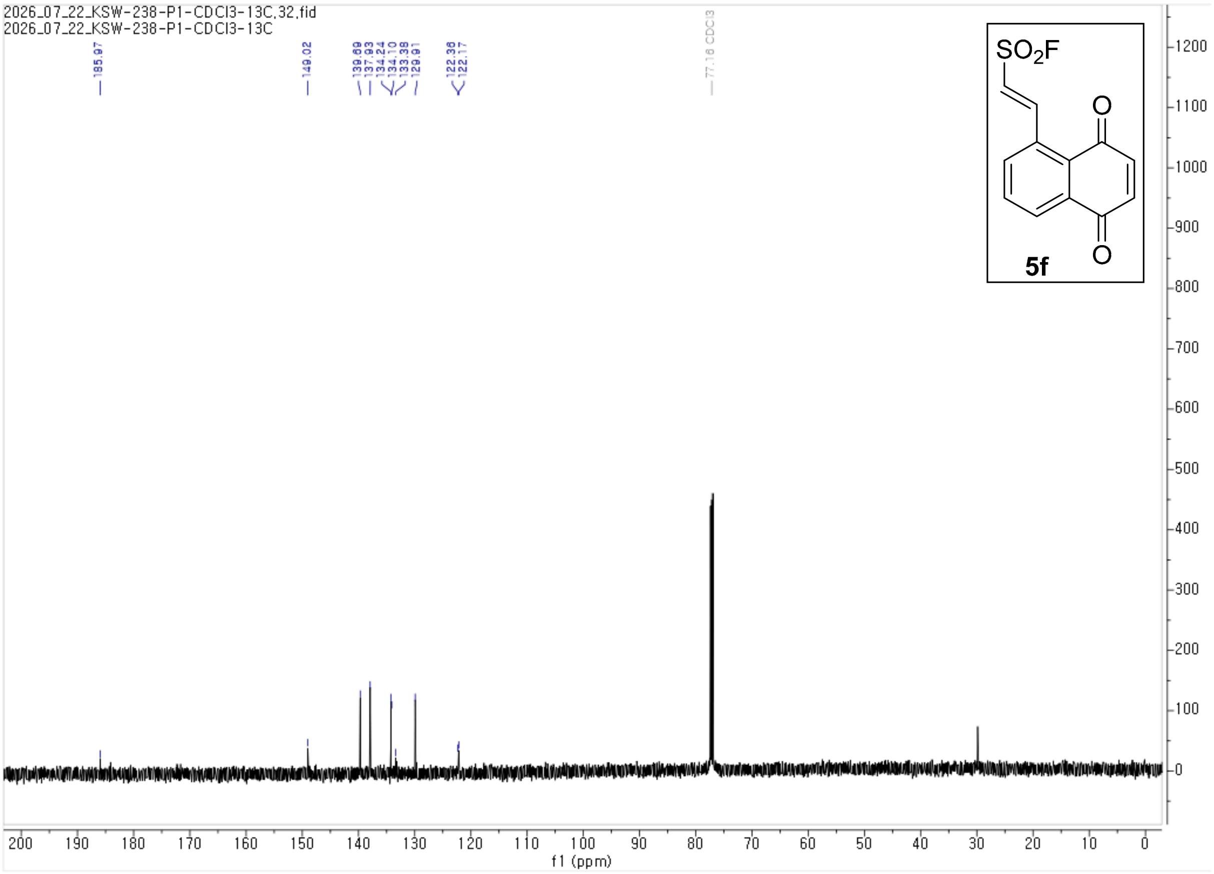
2026_07_22_KSW-238-P1-CDCl3-13C.32.fid
2026_07_22_KSW-238-P1-CDCl3-13C
185.97
149.02
139.89
137.93
134.24
134.10
133.38
129.91
122.38
122.17
77.16 CDCl3
SO2F
O
O
5f
f1 (ppm)

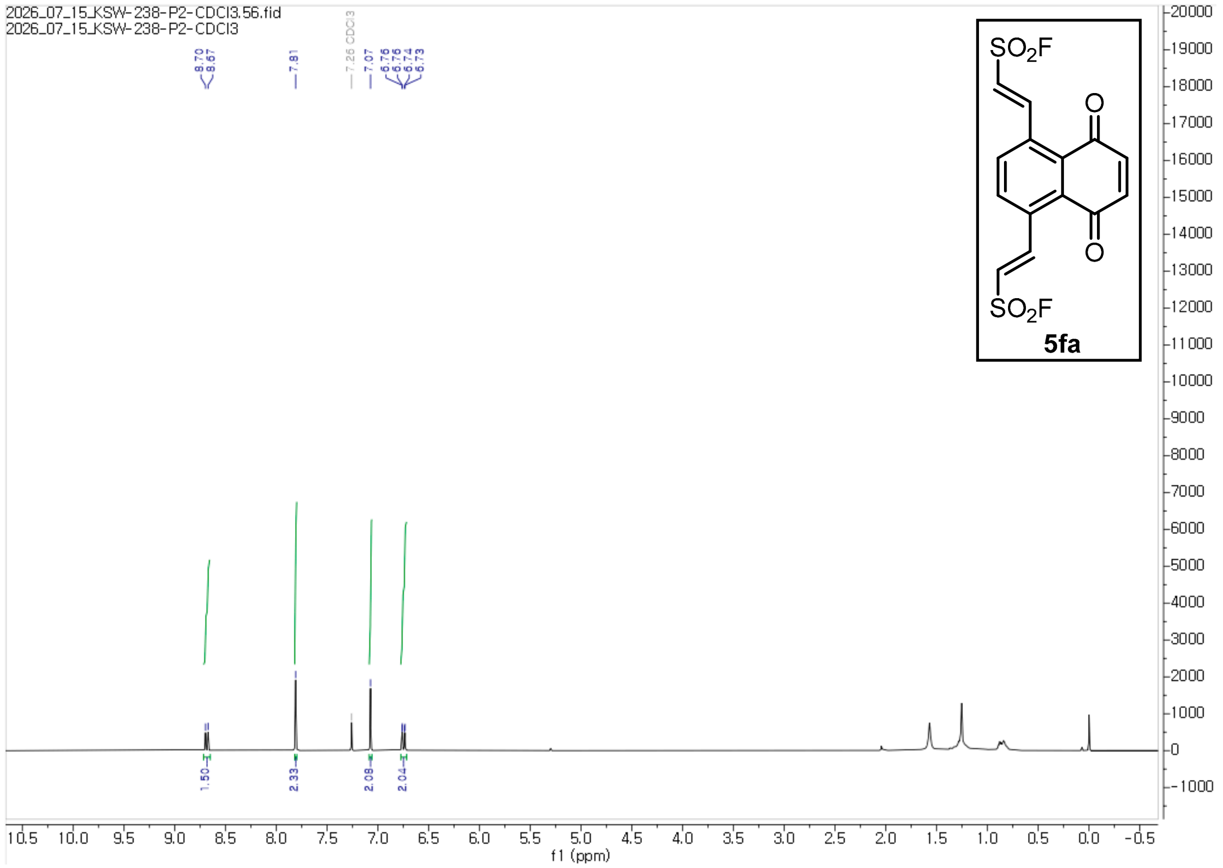
2026_07_15_KSW-238-P2-CDCl3.56.fid
2026_07_15_KSW-238-P2-CDCl3
8.70
8.67
7.81
7.26 CDCl3
7.07
6.76
6.76
6.74
6.73
1.50
2.33
2.08
2.04
f1 (ppm)
SO2F
O
O
SO2F
5fa

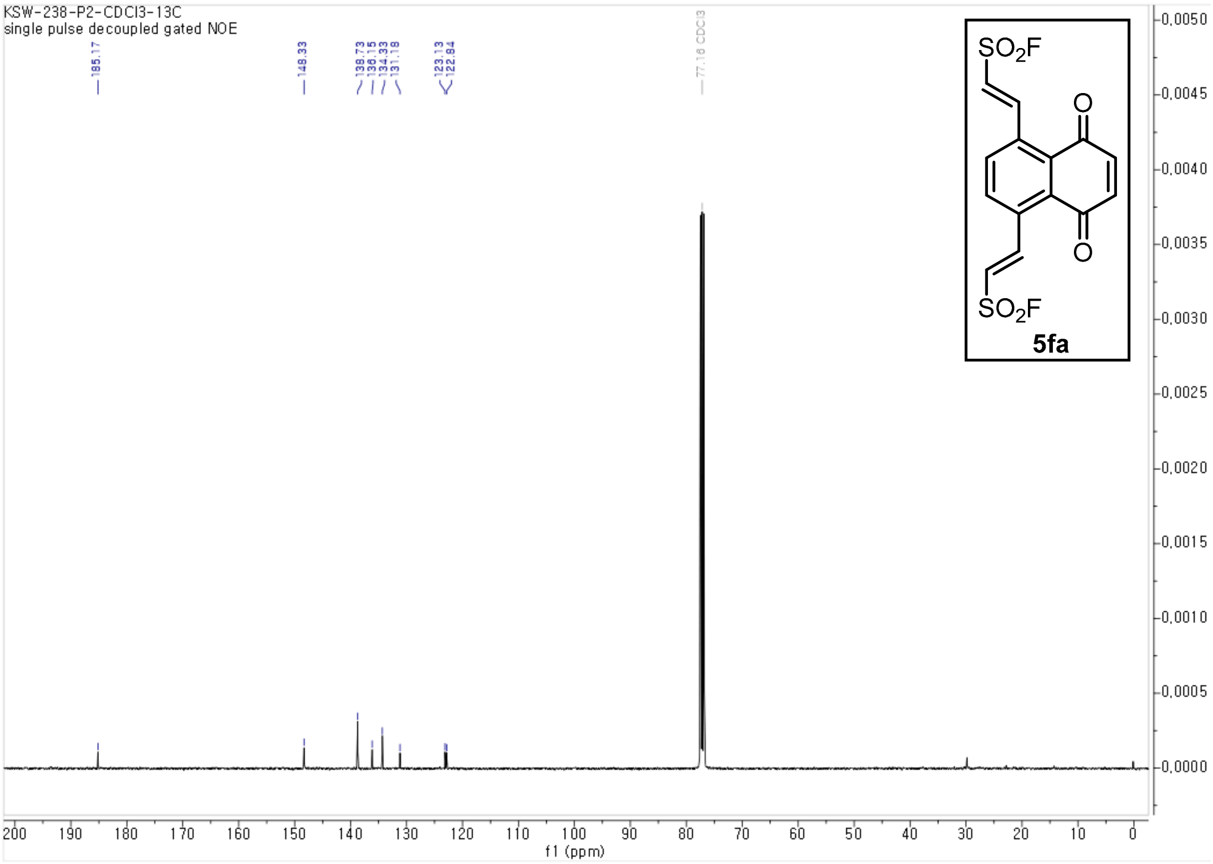
KSW-238-P2-CDCl3-13C
single pulse decoupled gated NOE
185.17
148.33
138.73
136.15
134.33
131.18
123.13
122.84
77.16 CDCl3
SO2F
SO2F
O
O
5fa
f1 (ppm)

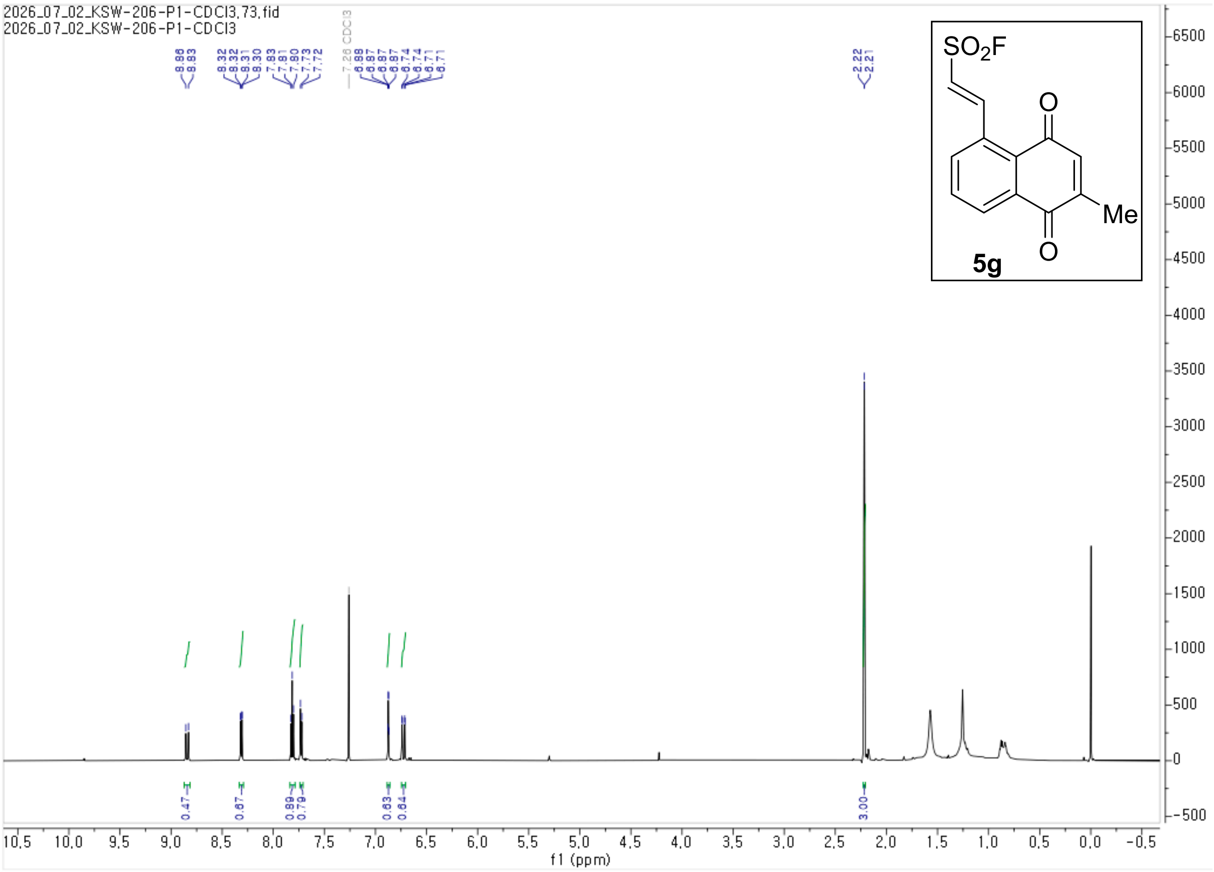
2026_07_02_KSW-206-P1-CDCl3,73,fid
2026_07_02_KSW-206-P1-CDCl3
SO2F
O
O
Me
5g
f1 (ppm)

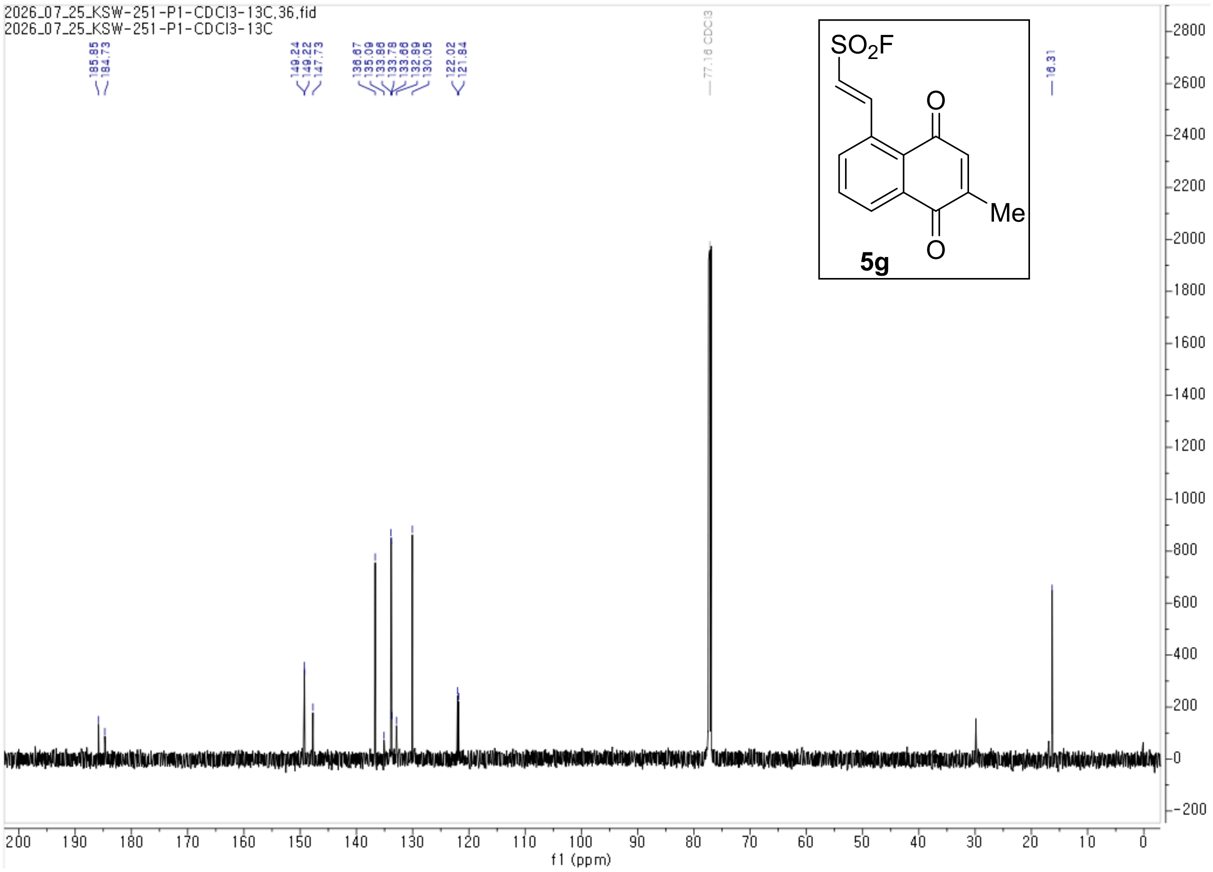
2026_07_25_KSW-251-P1-CDCl3-13C.36.fid
2026_07_25_KSW-251-P1-CDCl3-13C
185.85
184.73
149.24
149.22
147.73
136.67
135.09
133.88
133.78
133.66
132.89
130.05
122.02
121.84
77.16 CDCl3
16.31
SO2F
O
Me
O
5g
f1 (ppm)

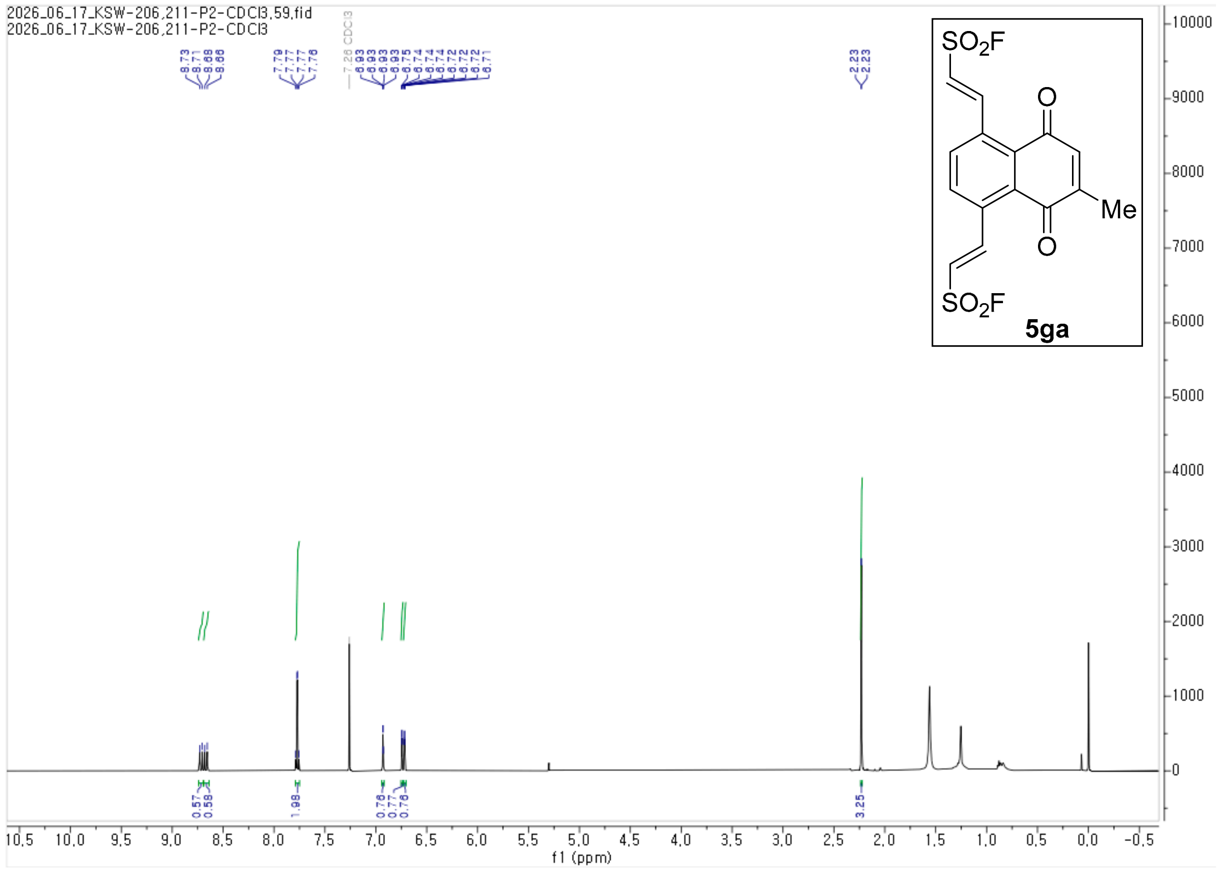
2026_06_17_KSW-206,211-P2-CDCl3,59.fid
2026_06_17_KSW-206,211-P2-CDCl3
SO2F
O
Me
O
SO2F
5ga
f1 (ppm)

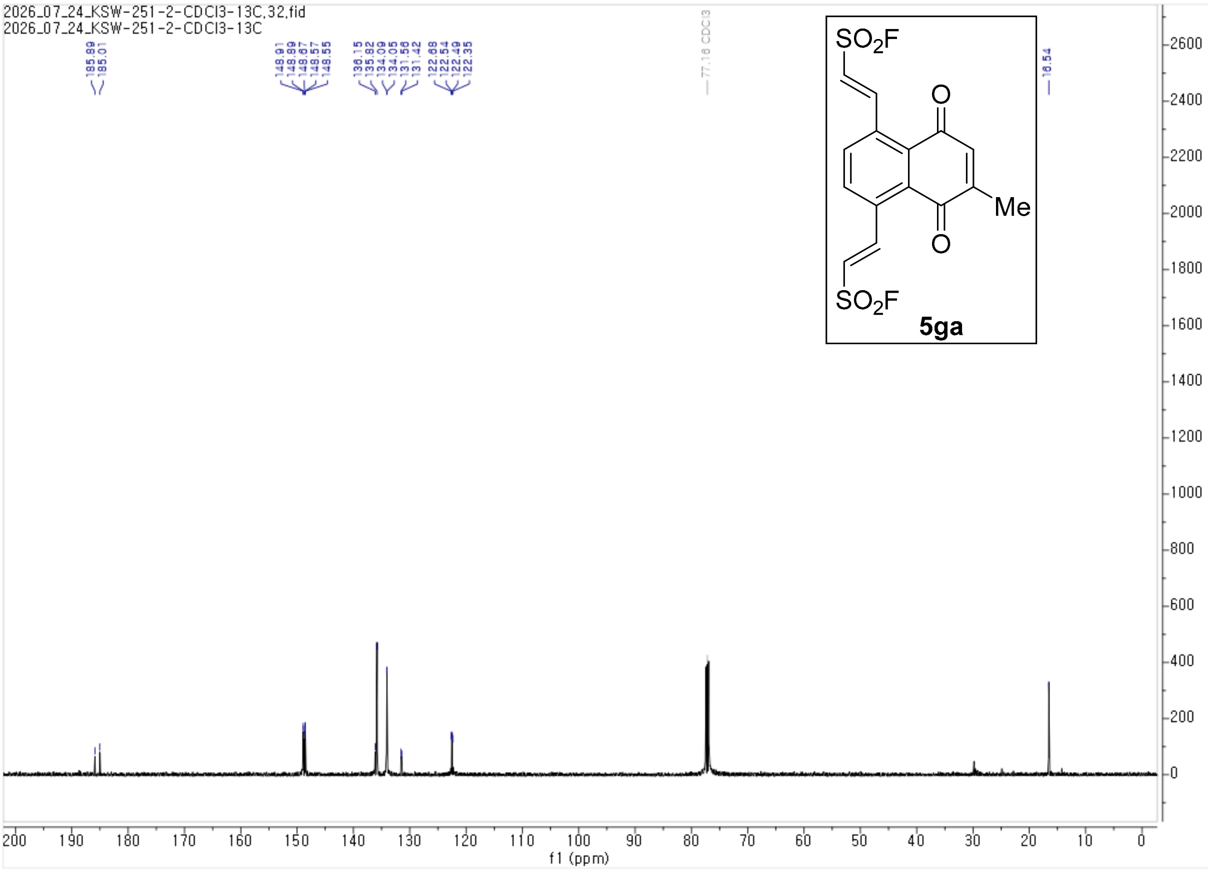

2026_07_24_KSW-251-2-CDCl3-13C.32.fid
2026_07_24_KSW-251-2-CDCl3-13C
185.89
185.01
148.91
148.89
148.67
148.57
148.55
136.15
135.82
134.09
134.05
131.56
131.42
122.68
122.54
122.49
122.35
77.16 CDCl3
16.54
SO2F
O
Me
O
SO2F
5ga
f1 (ppm)

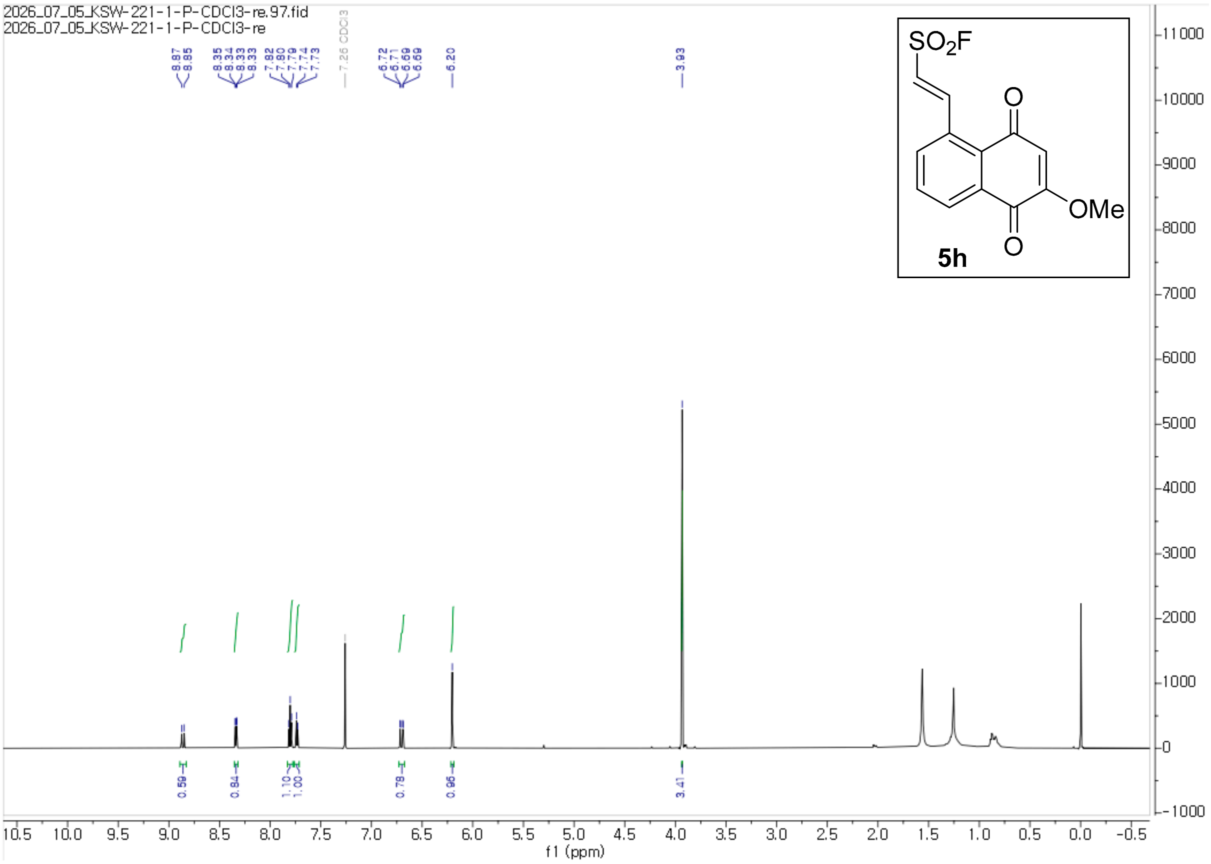

2026_07_05_KSW-221-1-P-CDCl3-re.97.fid
2026_07_05_KSW-221-1-P-CDCl3-re
8.87
8.85
8.35
8.34
8.33
8.33
7.82
7.80
7.79
7.74
7.73
7.26 CDCl3
6.72
6.71
6.69
6.69
6.20
3.93
SO2F
O
O
OMe
5h
0.59
0.84
1.10
1.00
0.78
0.96
3.41
f1 (ppm)

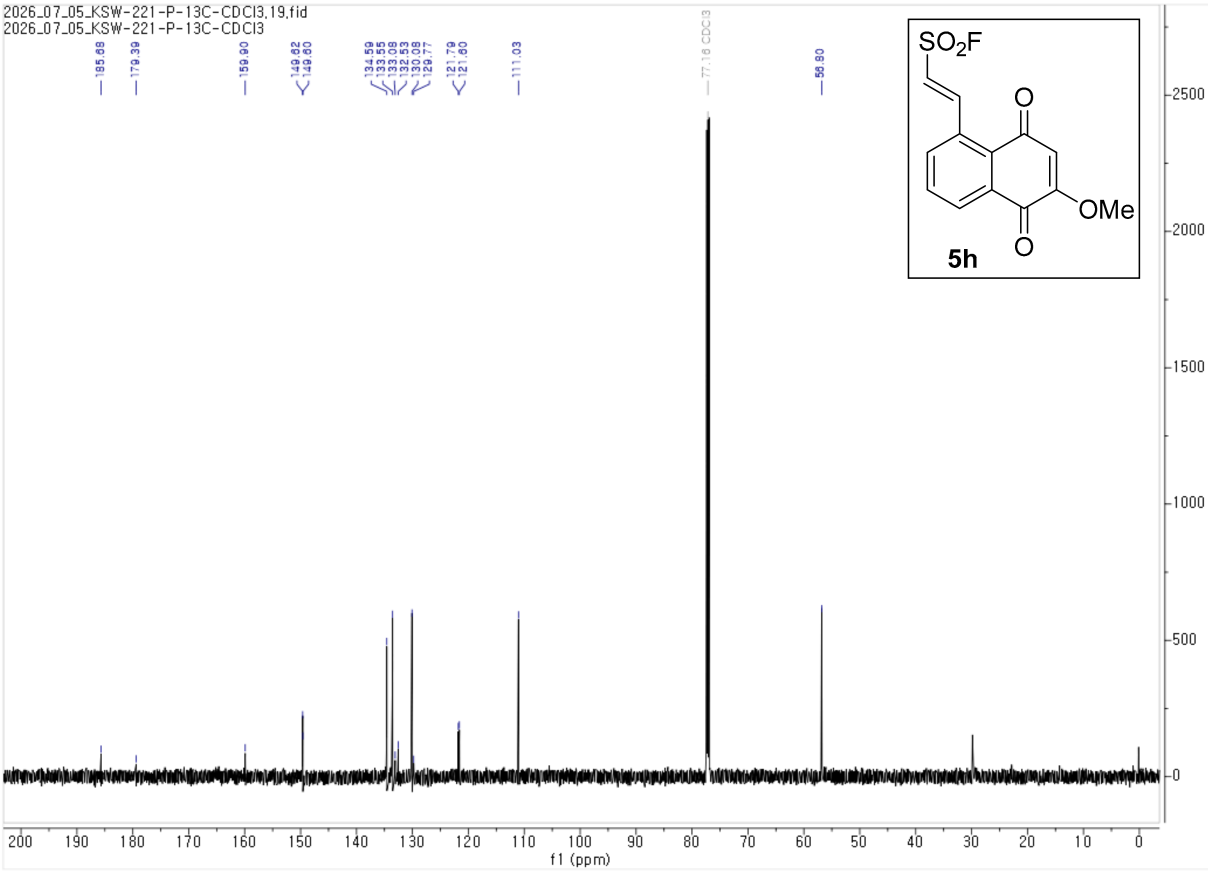
2026_07_05_KSW-221-P-13C-CDCl3.19.fid
2026_07_05_KSW-221-P-13C-CDCl3
185.68
179.39
159.90
149.62
149.60
134.59
133.55
133.08
132.53
130.08
129.77
121.79
121.60
111.03
77.16 CDCl3
56.80
SO2F
O
OMe
O
5h
f1 (ppm)

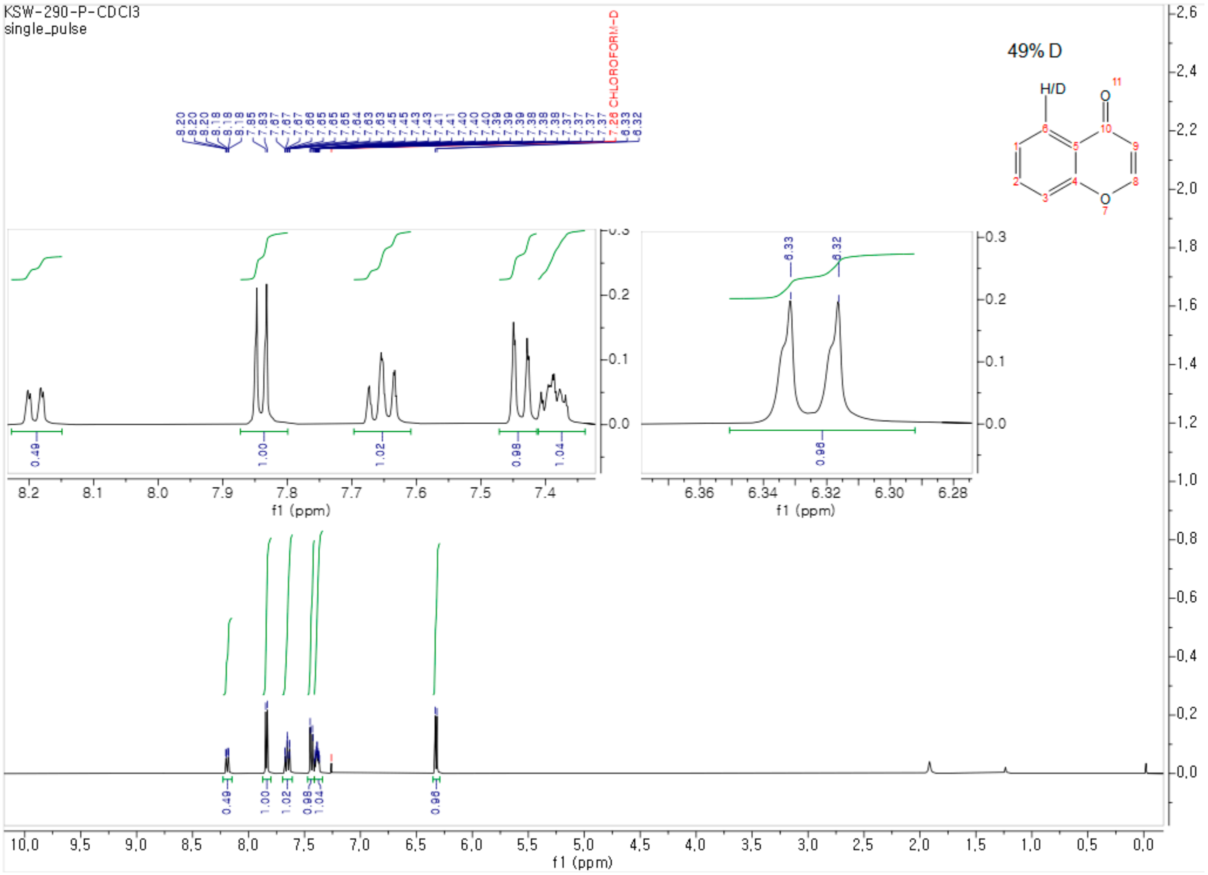
KSW-290-P-CDCl3
single_pulse
CHLOROFORM-D
49% D
H/D
f1 (ppm)

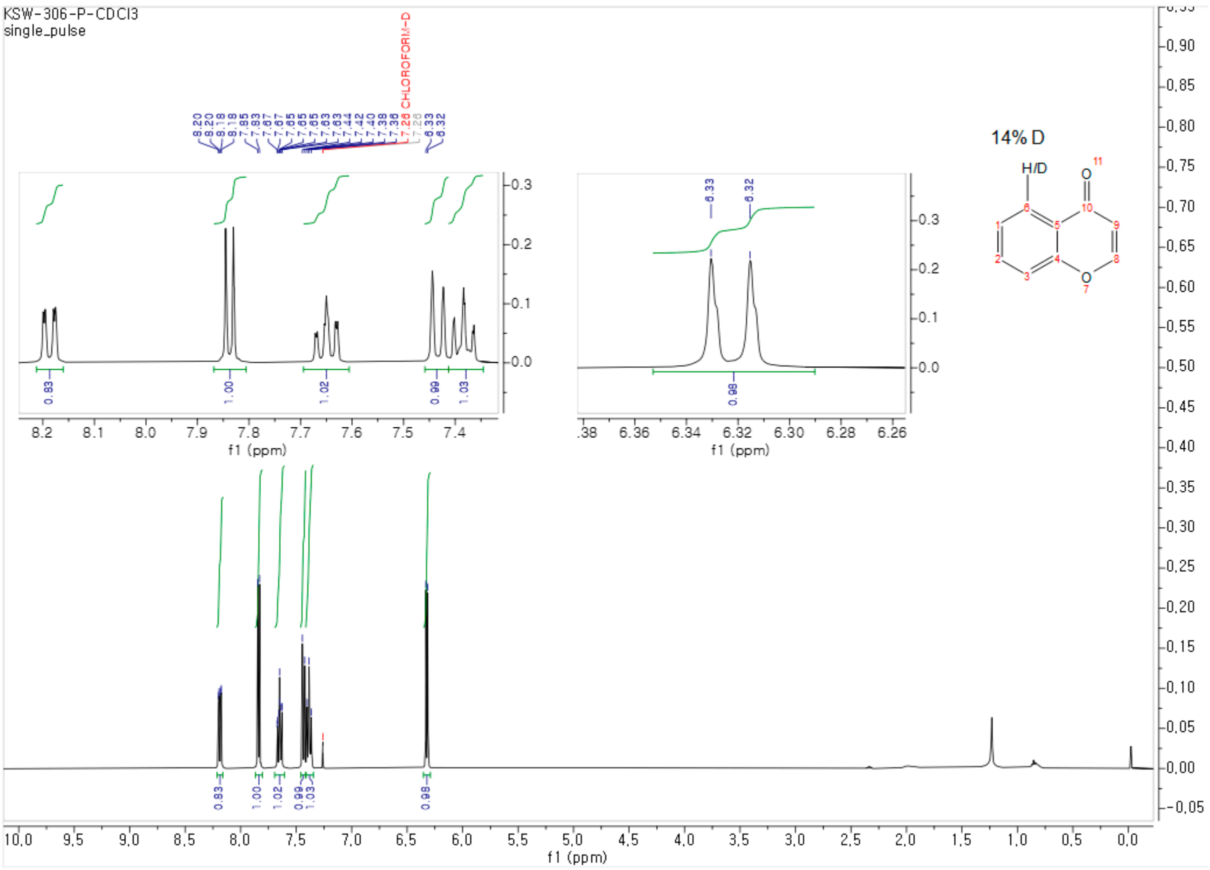
KSW-306-P-CDCl3
single_pulse
CHLOROFORM-D
14% D
H/D
f1 (ppm)

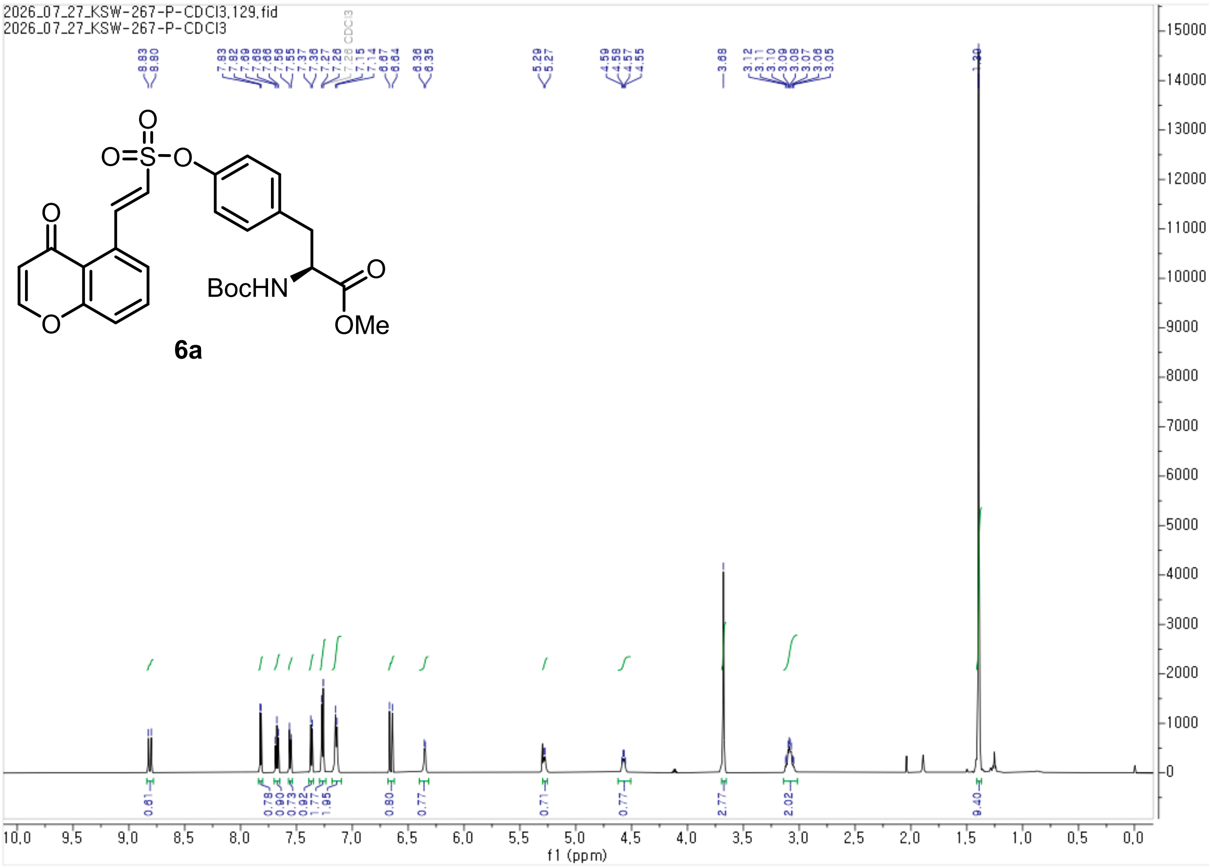
2026_07_27_KSW-267-P-CDCl3.129.fid
2026_07_27_KSW-267-P-CDCl3
BocHN
OMe
6a
f1 (ppm)

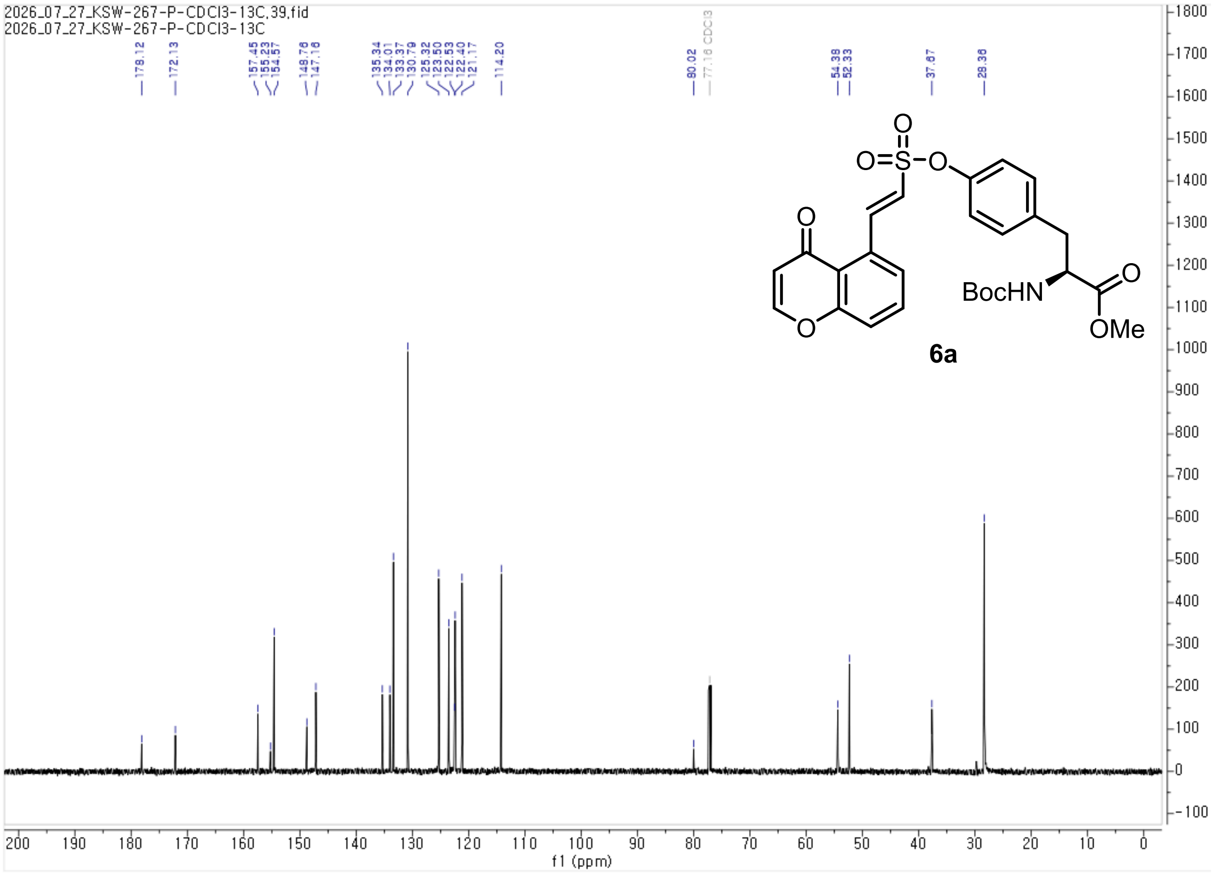
2026_07_27_KSW-267-P-CDCl3-13C.39.fid
2026_07_27_KSW-267-P-CDCl3-13C
178.12
172.13
157.45
155.23
154.57
148.78
147.18
135.34
134.01
133.37
130.79
125.32
123.50
122.53
122.40
121.17
114.20
80.02
77.16 CDCl3
54.38
52.33
37.67
28.36
BocHN
OMe
6a
f1 (ppm)

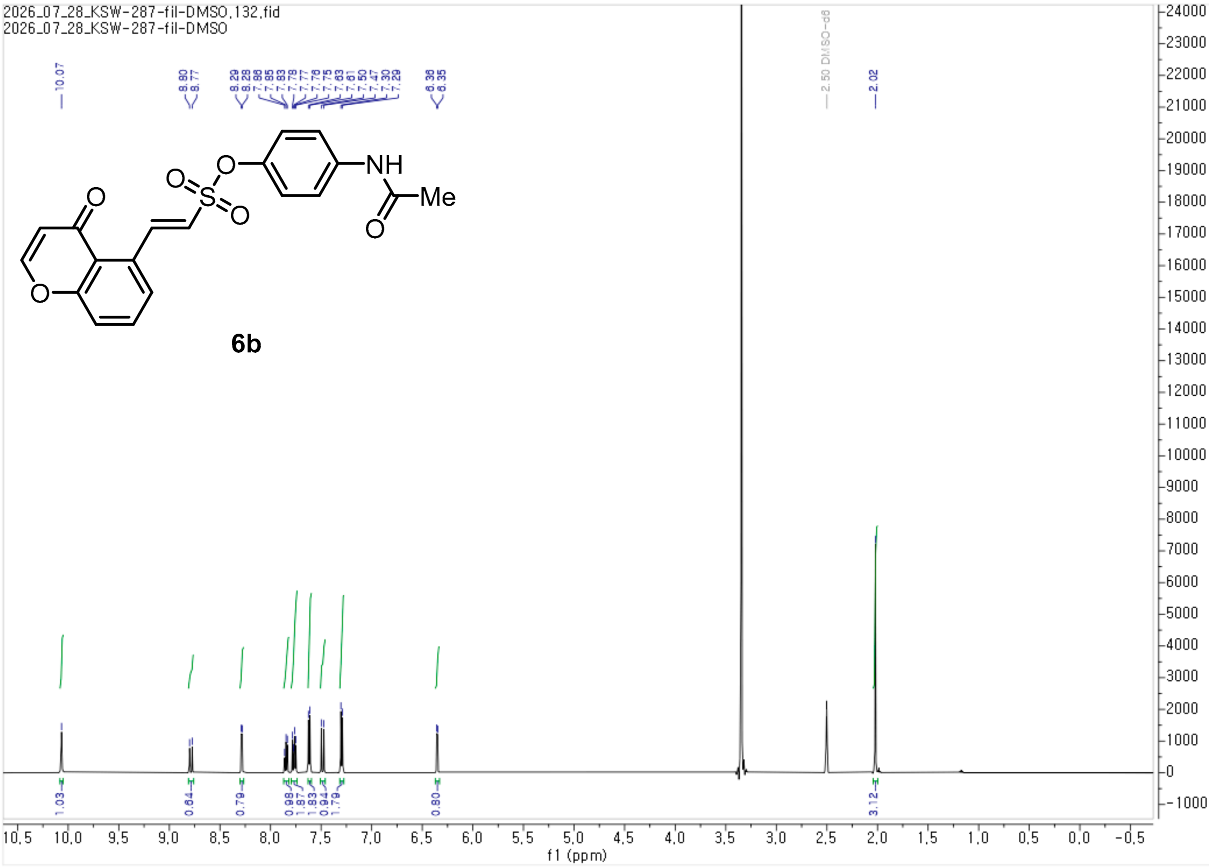
2026_07_28_KSW-287-fil-DMSO,132,fid
2026_07_28_KSW-287-fil-DMSO
6b
f1 (ppm)

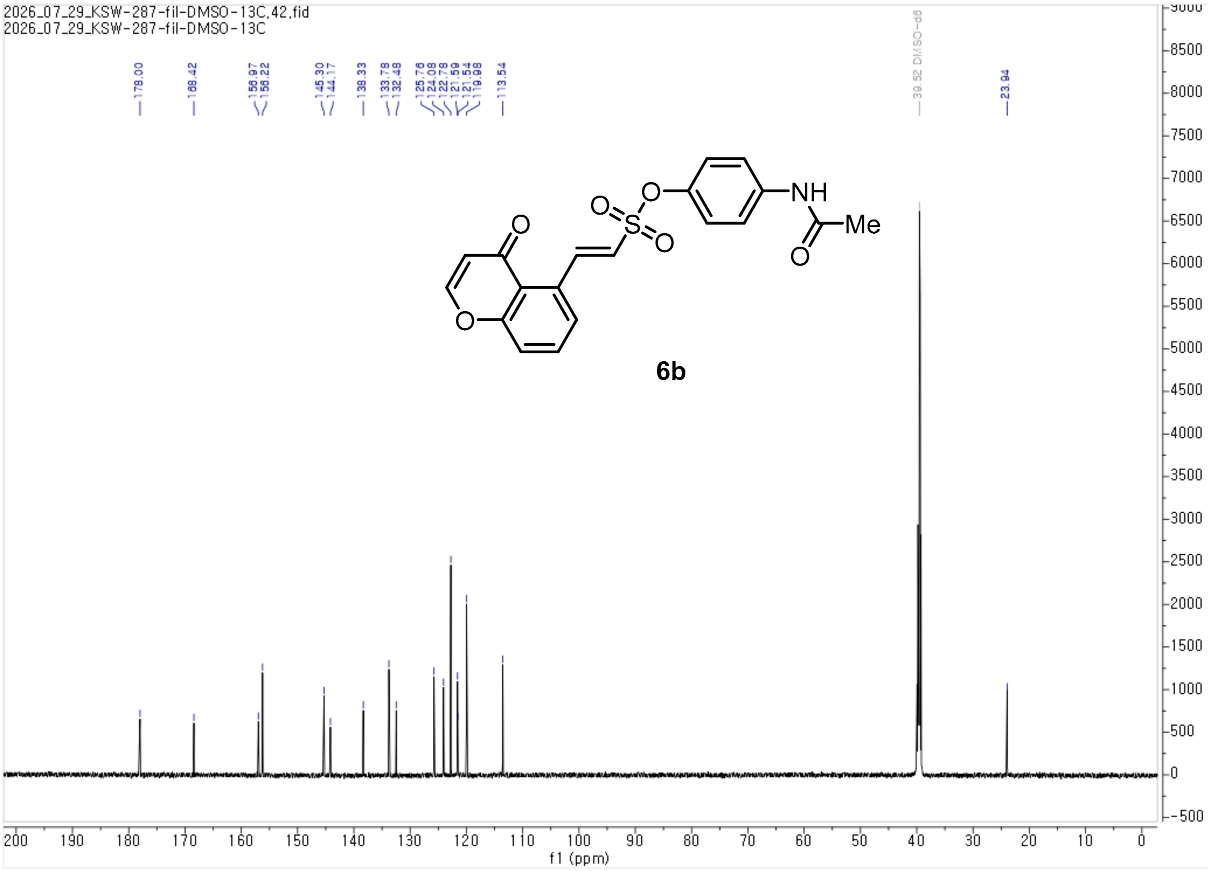
2026_07_29_KSW-287-fil-DMSO-13C.42.fid
2026_07_29_KSW-287-fil-DMSO-13C
178.00
168.42
156.97
156.22
145.30
144.17
138.33
133.78
132.48
125.76
124.08
122.78
121.59
121.54
119.98
113.54
39.52 DMSO-d6
23.94
NH
Me
6b
f1 (ppm)

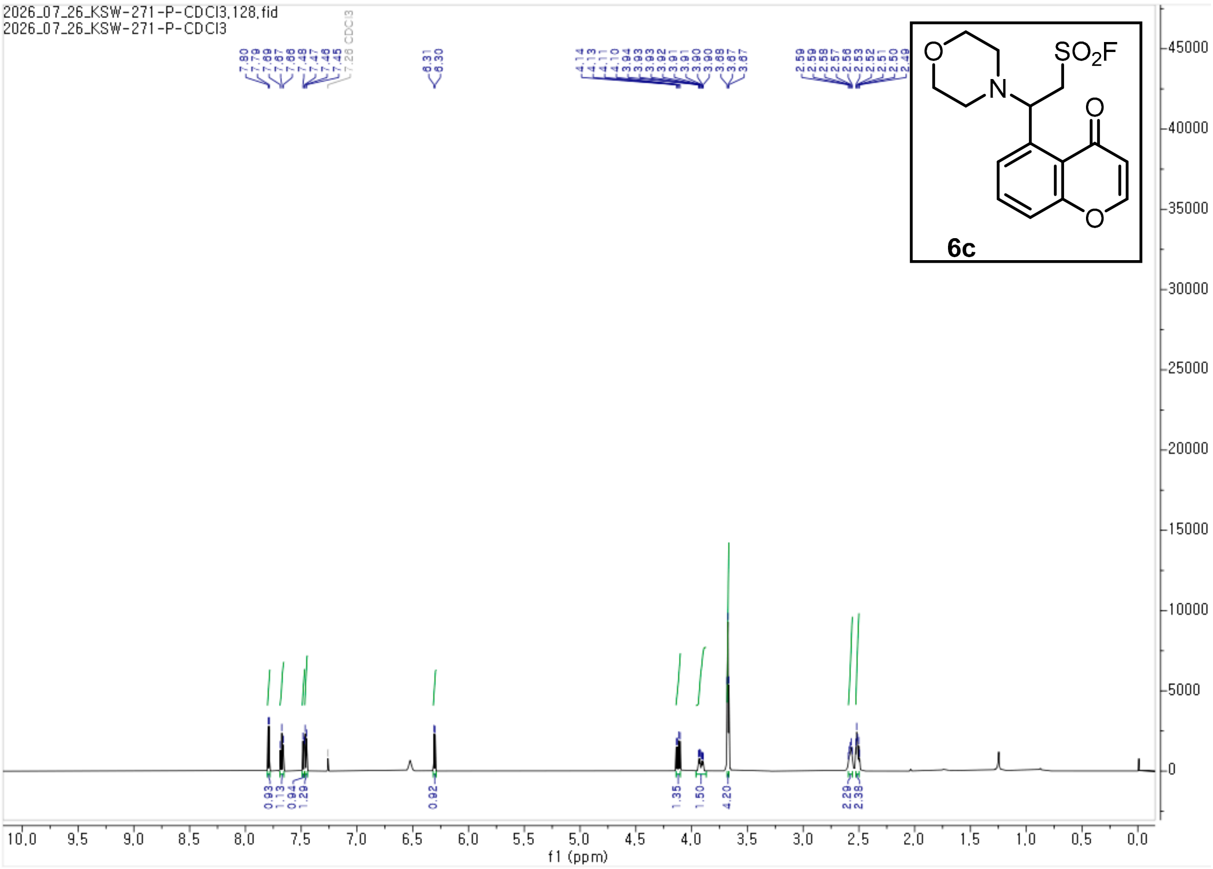
2026_07_26_KSW-271-P-CDCl3.128.fid
2026_07_26_KSW-271-P-CDCl3
6c
SO2F
f1 (ppm)

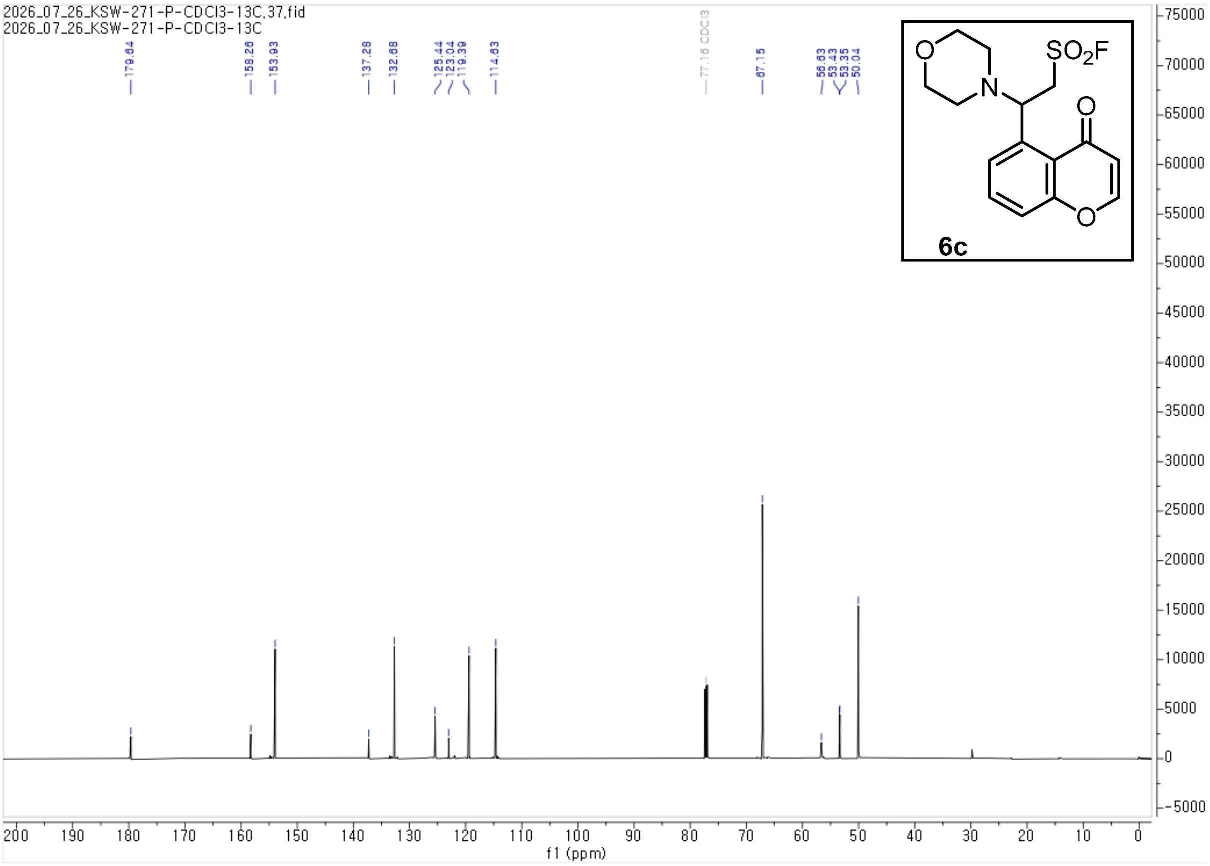

2026_07_26_KSW-271-P-CDCl3-13C,37,fid
2026_07_26_KSW-271-P-CDCl3-13C
179.84
158.26
153.93
137.28
132.68
125.44
123.04
119.39
114.63
77.16 CDCl3
67.15
56.63
53.43
53.35
50.04
6c
f1 (ppm)

4. HR-MS analytical data of all products

Compound 3a

```
[ Elemental Composition ]                                          Page: 1
Data   : FAB-1-SW157-3-002              Date : 27-Apr-2026 11:11
Sample: High resolution GC Mass(JMS-700)
Note : SMlab-Mass Analysis & Research Center
Inlet : Direct                          Ion Mode : FAB+
RT : 0.74 min                           Scan#: 23+25+23
Elements : C 12/0, H 10/0, F 1/0, O 4/0, S 1/0
Mass Tolerance        : 1000ppm, 3mmu if m/z < 3, 5mmu if m/z > 5
Unsaturation (U.S.) : -0.5 - 80.0

Observed m/z Int%   Err[ppm / mmu]    U.S.  Composition
  255.0123   100.0      -1.6 /  -0.4    8.5  C 11 H 8 F O 4 S
```

```
[ Mass Spectrum ]
Data   : FAB-1-SW157-3-002              Date : 27-Apr-2026 11:11
Sample: High resolution GC Mass(JMS-700)
Note : SMlab-Mass Analysis & Research Center
Inlet : Direct                          Ion Mode : FAB+
Spectrum Type : Normal Ion [MF-Linear]
RT : 0.74 min           Scan# : 23+25+23
BP : m/z 255.0123       Int.  : 99.94
Output m/z range : 254.7061 to 255.3015      Cut Level : 20.82 %
```

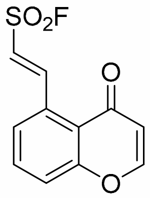

3a

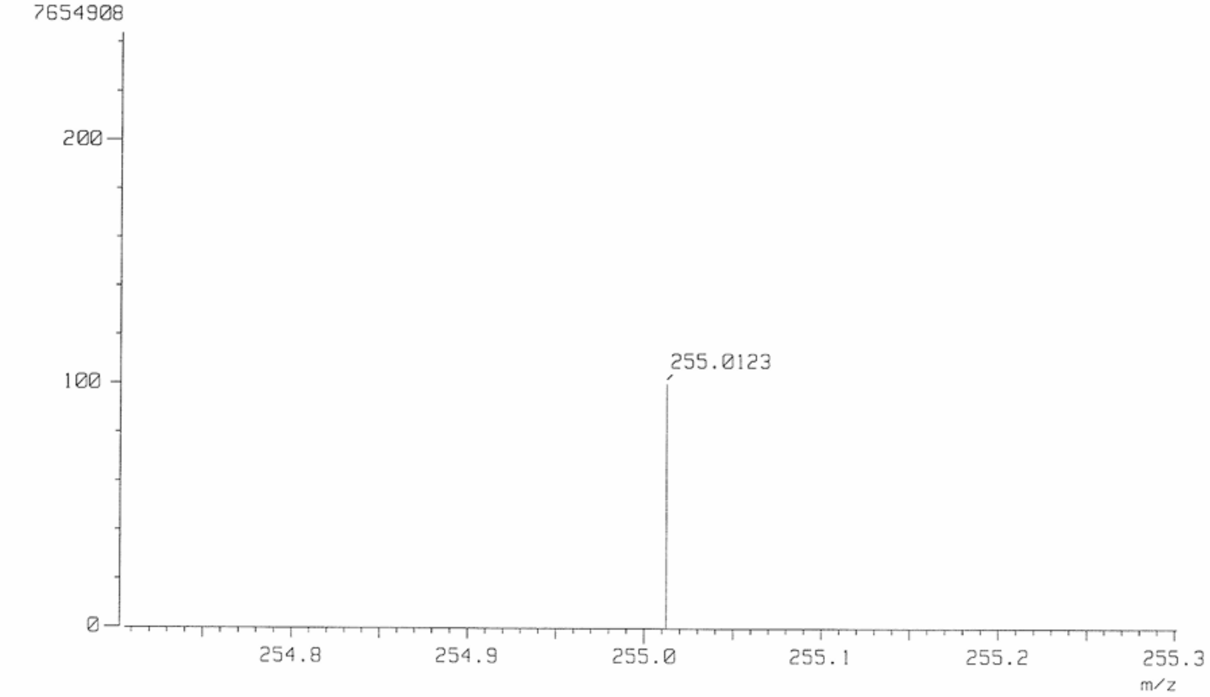

Compound 3b

[Elemental Composition] Page: 1
Data : FAB-3-SW159-2-006 Date : 27-Apr-2026 11:32
Sample: High resolution GC Mass(JMS-700)
Note : SMlab-Mass Analysis & Research Center
Inlet : Direct Ion Mode : FAB+
RT : 0.74 min Scan#: 23+18
Elements : C 13/0, H 10/0, F 1/0, O 4/0, S 1/0
Mass Tolerance : 1000ppm, 3mmu if m/z < 3, 5mmu if m/z > 5
Unsaturation (U.S.) : -0.5 - 80.0

Observed m/z	Int%	Err[ppm / mmu]	U.S.	Composition
269.0280	100.0	-1.6 / -0.4	8.5	C 12 H 10 F O 4 S

[Mass Spectrum]
Data : FAB-3-SW159-2-006 Date : 27-Apr-2026 11:32
Sample: High resolution GC Mass(JMS-700)
Note : SMlab-Mass Analysis & Research Center
Inlet : Direct Ion Mode : FAB+
Spectrum Type : Normal Ion [MF-Linear]
RT : 0.74 min Scan# : 23+18
BP : m/z 269.0280 Int. : 99.96
Output m/z range : 268.7010 to 269.3448 Cut Level : 64.71 %

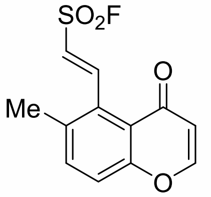

3b

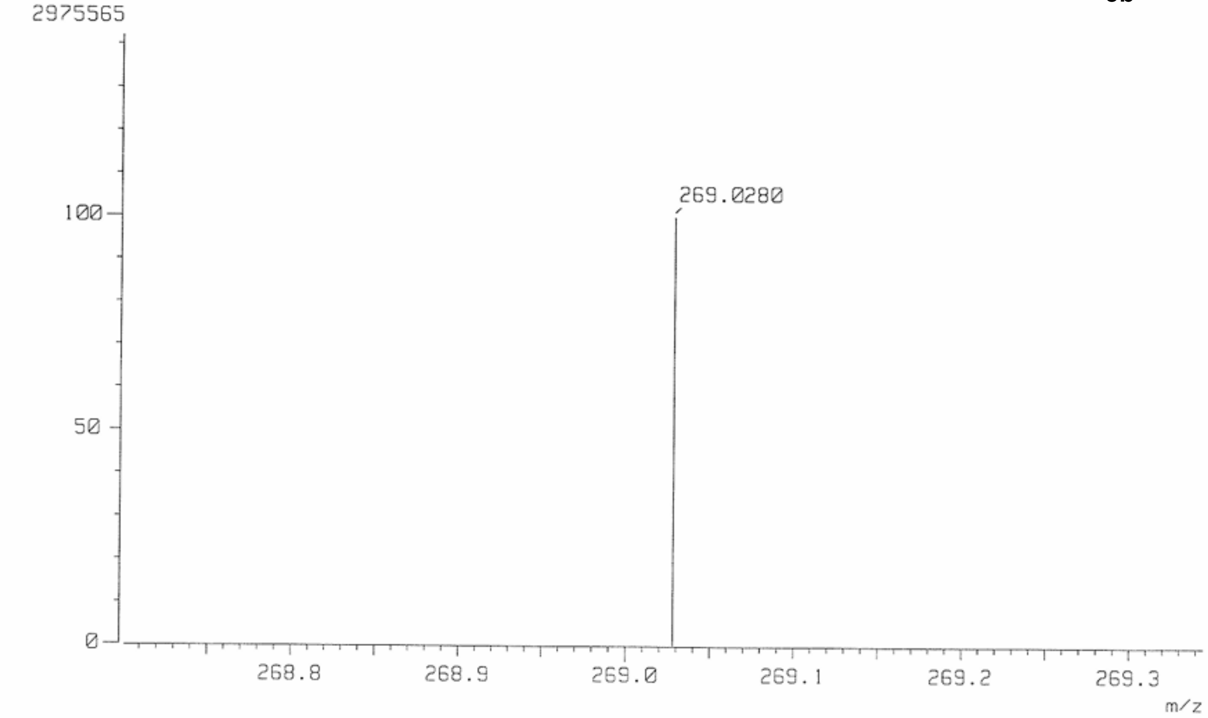

Compound 3c

```
[ Elemental Composition ]                                              Page: 1
Data   : FAB-4-SW160-3-010              Date : 27-Apr-2026 11:43
Sample: High resolution GC Mass(JMS-700)
Note : SMlab-Mass Analysis & Research Center
Inlet : Direct                          Ion Mode : FAB+
RT : 0.20 min                           Scan#: 7+27+7
Elements : C 12/0, H 7/0, F 2/0, O 4/0, S 1/0
Mass Tolerance       : 1000ppm, 3mmu if m/z < 3, 5mmu if m/z > 5
Unsaturation (U.S.)  : -0.5 - 80.0
```

Observed m/z	Int%	Err[ppm / mmu]	U.S.	Composition
273.0031	94.4	-0.7 / -0.2	8.5	C 11 H 7 F 2 O 4 S

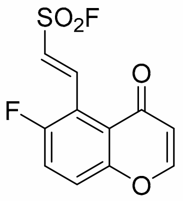

3c

```
[ Mass Spectrum ]
Data   : FAB-4-SW160-3-010              Date : 27-Apr-2026 11:43
Sample: High resolution GC Mass(JMS-700)
Note : SMlab-Mass Analysis & Research Center
Inlet : Direct                          Ion Mode : FAB+
Spectrum Type : Normal Ion [MF-Linear]
RT : 0.20 min            Scan# : 7+27+7
BP : m/z 189.0330        Int.  : 74.11
Output m/z range : 272.7146 to 273.3516      Cut Level : 57.35 %
```

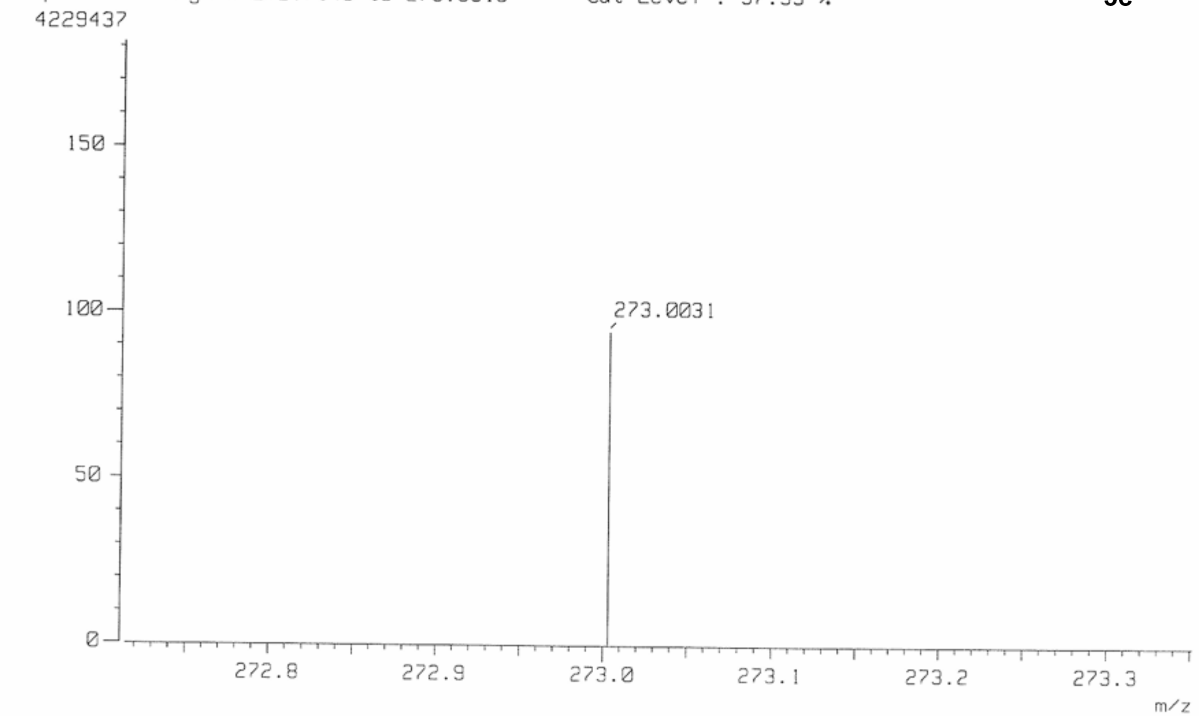

Compound 3d

[Elemental Composition] Page: 1
Data : FAB-5-SW161-2-012 Date : 27-Apr-2026 11:49
Sample: High resolution GC Mass(JMS-700)
Note : SMlab-Mass Analysis & Research Center
Inlet : Direct Ion Mode : FAB+
RT : 2.27 min Scan#: 69
Elements : C 12/0, H 20/0, Cl 1/0, F 1/0, O 4/0, S 1/0
Mass Tolerance : 1000ppm, 3mmu if m/z < 3, 5mmu if m/z > 5
Unsaturation (U.S.) : -0.5 - 80.0

Observed m/z	Int%	Err[ppm / mmu]	U.S.	Composition
288.9735	100.0	-0.9 / -0.3	8.5	C 11 H 7 Cl F O 4 S

[Mass Spectrum]
Data : FAB-5-SW161-2-012 Date : 27-Apr-2026 11:49
Sample: High resolution GC Mass(JMS-700)
Note : SMlab-Mass Analysis & Research Center
Inlet : Direct Ion Mode : FAB+
Spectrum Type : Normal Ion [MF-Linear]
RT : 2.27 min Scan# : 69
BP : m/z 288.9735 Int. : 46.68
Output m/z range : 288.6913 to 289.3489 Cut Level : 72.06 %

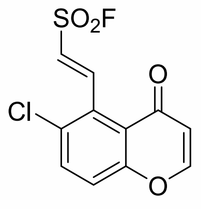

3d

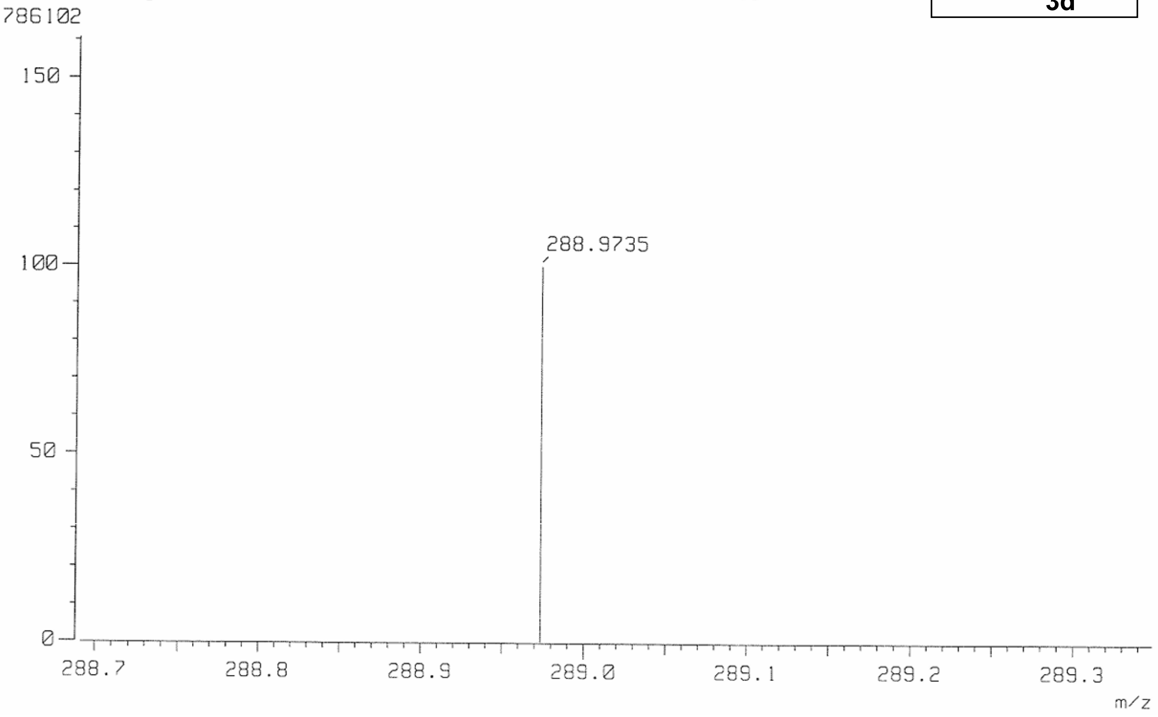

Compound 3e

[Elemental Composition] Page: 1
Data : FAB-10-SW168-2-020 Date : 27-Apr-2026 12:17
Sample: High resolution GC Mass(JMS-700)
Note : SMlab-Mass Analysis & Research Center
Inlet : Direct Ion Mode : FAB+
RT : 2.54 min Scan#: (76,78)+78
Elements : C 13/0, H 10/0, Br 1/0, F 1/0, O 4/0, S 1/0
Mass Tolerance : 1000ppm, 3mmu if m/z < 3, 5mmu if m/z > 5
Unsaturation (U.S.) : -0.5 - 80.0

Observed m/z	Int%	Err[ppm / mmu]	U.S.	Composition
332.9230	35.4	-0.6 / -0.2	8.5	C 11 H 7 Br F O 4 S

[Mass Spectrum]
Data : FAB-10-SW168-2-020 Date : 27-Apr-2026 12:17
Sample: High resolution GC Mass(JMS-700)
Note : SMlab-Mass Analysis & Research Center
Inlet : Direct Ion Mode : FAB+
Spectrum Type : Normal Ion [MF-Linear]
RT : 2.54 min Scan# : (76,78)+78
BP : m/z 307.0967 Int. : 57.49
Output m/z range : 332.6250 to 333.2842 Cut Level : 11.76 %
1865560

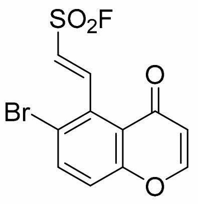

3e

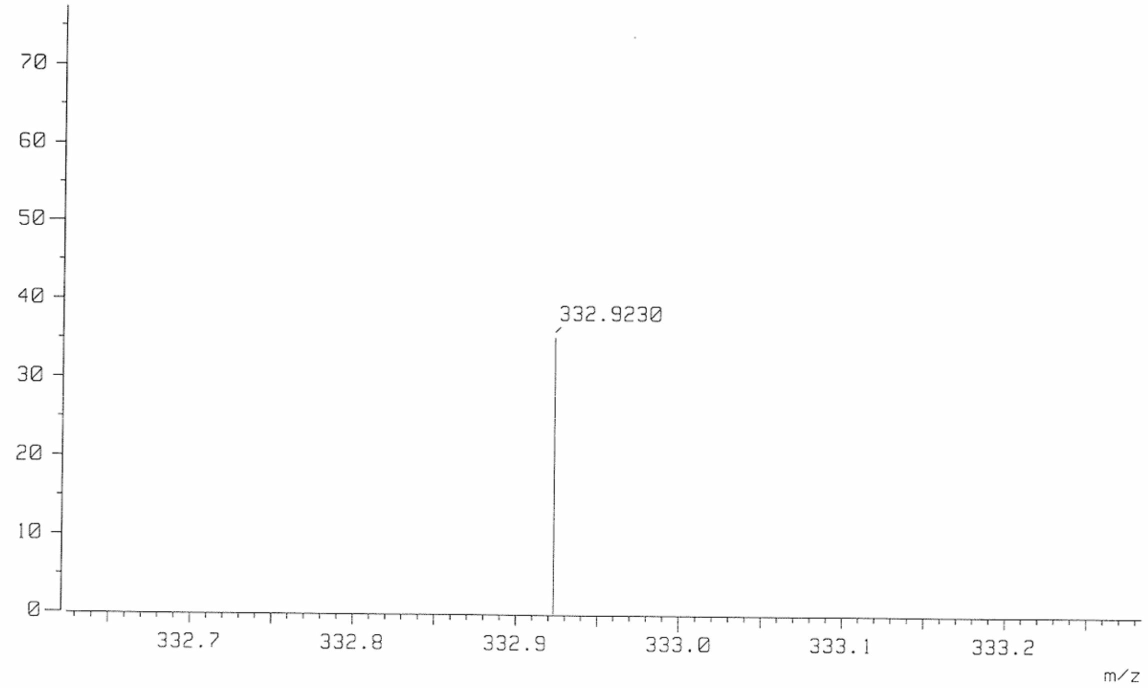

Compound 3f

```
[ Elemental Composition ]                                    Page: 1
Data   : FAB-12-SW171-4-027          Date : 27-Apr-2026 12:36
Sample: High resolution GC Mass(JMS-700)
Note : SMlab-Mass Analysis & Research Center
Inlet : Direct                       Ion Mode : FAB+
RT : 1.97 min                        Scan#: 60+59+60+60
Elements : C 12/0, H 10/0, Br 1/0, Cl 1/0, F 1/0, O 4/0, S 1/0
Mass Tolerance        : 1000ppm, 3mmu if m/z < 3, 5mmu if m/z > 5
Unsaturation (U.S.) : -0.5 - 80.0

Observed m/z Int%   Err[ppm / mmu]    U.S.  Composition
  368.8995    74.4     -1.2 /  -0.4    7.5  C 11 H 8 Br Cl F O 4 S
```

```
[ Mass Spectrum ]
Data   : FAB-12-SW171-4-027          Date : 27-Apr-2026 12:36
Sample: High resolution GC Mass(JMS-700)
Note : SMlab-Mass Analysis & Research Center
Inlet : Direct                       Ion Mode : FAB+
Spectrum Type : Normal Ion [MF-Linear]
RT : 1.97 min          Scan# : 60+59+60+60
BP : m/z 307.1975      Int.  : 45.87
Output m/z range : 368.4946 to 369.2798      Cut Level : 59.56 %
```

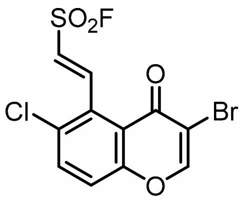

3f

3507730

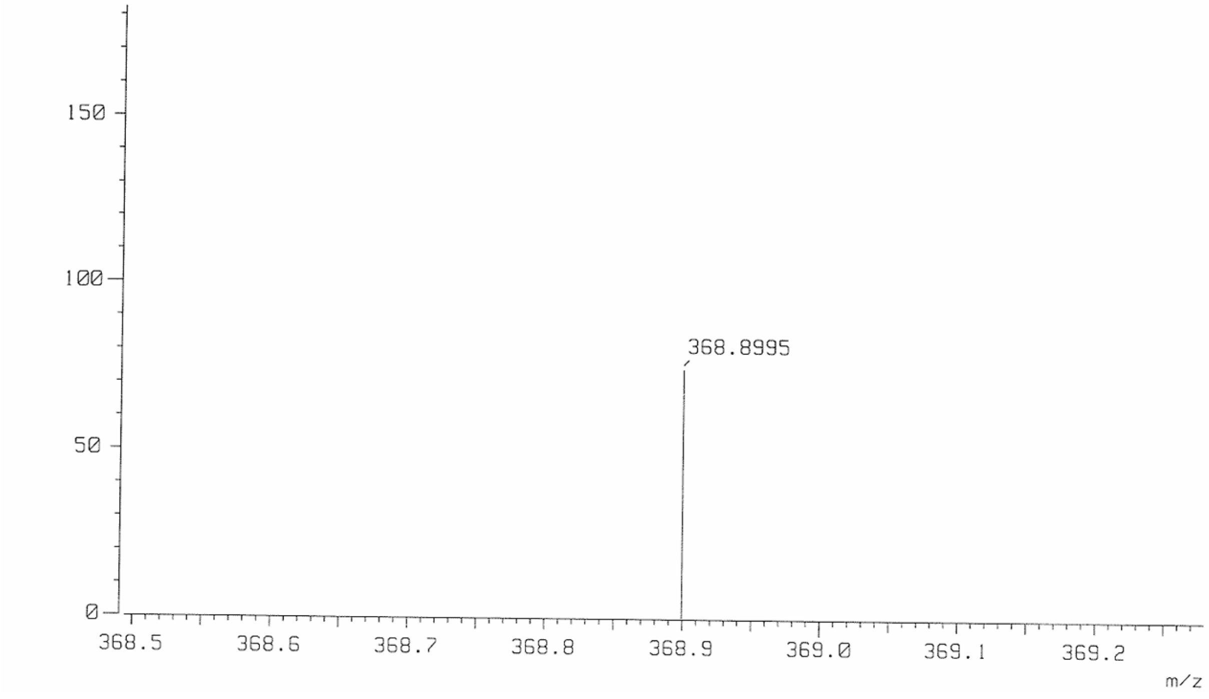

Compound 3g

```
[ Elemental Composition ]                                          Page: 1
Data   : FAB-11-SW169-4-017         Date : 27-Apr-2026 12:08
Sample: High resolution GC Mass(JMS-700)
Note : SMlab-Mass Analysis & Research Center
Inlet : Direct                      Ion Mode : FAB+
RT : 1.10 min                       Scan#: 34+33+31+31
Elements : C 14/0, H 10/0, Cl 1/0, F 1/0, O 4/0, S 1/0
Mass Tolerance      : 1000ppm, 3mmu if m/z < 3, 5mmu if m/z > 5
Unsaturation (U.S.) : -0.5 - 80.0
```

Observed m/z	Int%	Err[ppm / mmu]	U.S.	Composition
302.9893	100.0	-0.3 / -0.1	8.5	C 12 H 9 Cl F O 4 S

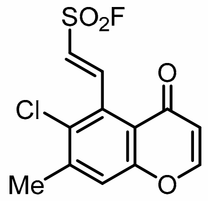

3g

```
[ Mass Spectrum ]
Data   : FAB-11-SW169-4-017         Date : 27-Apr-2026 12:08
Sample: High resolution GC Mass(JMS-700)
Note : SMlab-Mass Analysis & Research Center
Inlet : Direct                      Ion Mode : FAB+
Spectrum Type : Normal Ion [MF-Linear]
RT : 1.10 min             Scan# : 34+33+31+31
BP : m/z 302.9893         Int.  : 52.98
Output m/z range : 302.6144 to 303.3414       Cut Level : 75.74 %
 3829252
```

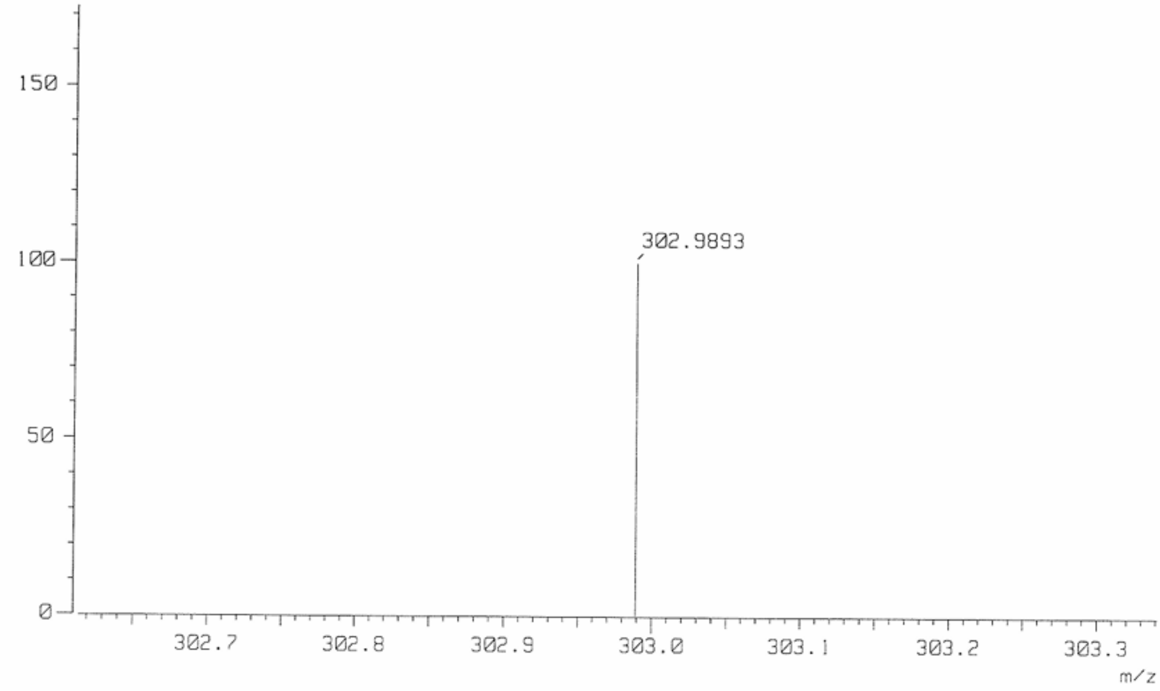

Compound 3i

[Elemental Composition]　　Page: 1

Data : FAB-8-SW165-2-007　　Date : 27-Apr-2026 11:36
Sample: High resolution GC Mass(JMS-700)
Note : SMlab-Mass Analysis & Research Center
Inlet : Direct　　Ion Mode : FAB+
RT : 3.14 min　　Scan#: 95
Elements : C 13/0, H 12/0, F 1/0, O 4/0, S 1/0
Mass Tolerance : 1000ppm, 3mmu if m/z < 3, 5mmu if m/z > 5
Unsaturation (U.S.) : -0.5 - 80.0

Observed m/z	Int%	Err[ppm / mmu]	U.S.	Composition
269.0282	100.0	-0.6 / -0.2	8.5	C 12 H 10 F O 4 S

[Mass Spectrum]
Data : FAB-8-SW165-2-007　　Date : 27-Apr-2026 11:36
Sample: High resolution GC Mass(JMS-700)
Note : SMlab-Mass Analysis & Research Center
Inlet : Direct　　Ion Mode : FAB+
Spectrum Type : Normal Ion [MF-Linear]
RT : 3.14 min　　Scan# : 95
BP : m/z 269.0282　　Int. : 29.44
Output m/z range : 268.6699 to 269.4220　　Cut Level : 79.41 %

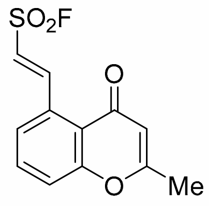

3i

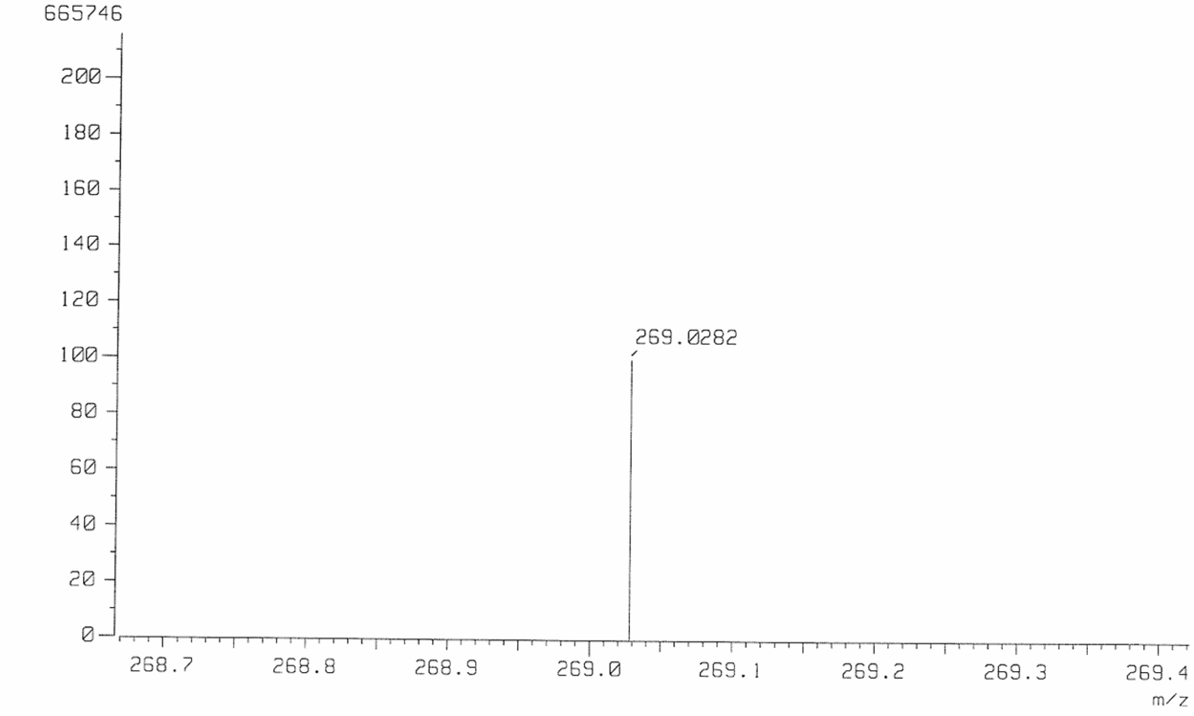

Compound 3j

[Elemental Composition] Page: 1
Data : FAB-10-KSW222-029 Date : 11-Jul-2026 16:43
Sample: High resolution GC Mass(JMS-700)
Note : SMlab-Mass Analysis & Research Center
Inlet : Direct Ion Mode : FAB+
RT : 3.30 min Scan#: 100+100+98+96
Elements : C 14/0, H 10/0, F 1/0, O 6/0, S 1/0
Mass Tolerance : 1000ppm, 3mmu if m/z < 3, 5mmu if m/z > 5
Unsaturation (U.S.) : -0.5 - 80.0

Observed m/z	Int%	Err[ppm / mmu]	U.S.	Composition
313.0181	100.0	-0.4 / -0.1	9.5	C 13 H 10 F O 6 S

[Mass Spectrum]
Data : FAB-10-KSW222-029 Date : 11-Jul-2026 16:43
Sample: High resolution GC Mass(JMS-700)
Note : SMlab-Mass Analysis & Research Center
Inlet : Direct Ion Mode : FAB+
Spectrum Type : Normal Ion [MF-Linear]
RT : 3.30 min Scan# : 100+100+98+96
BP : m/z 313.0181 Int. : 37.16
Output m/z range : 312.6828 to 313.3183 Cut Level : 77.94 %

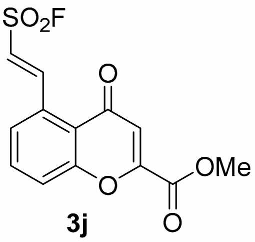

3j

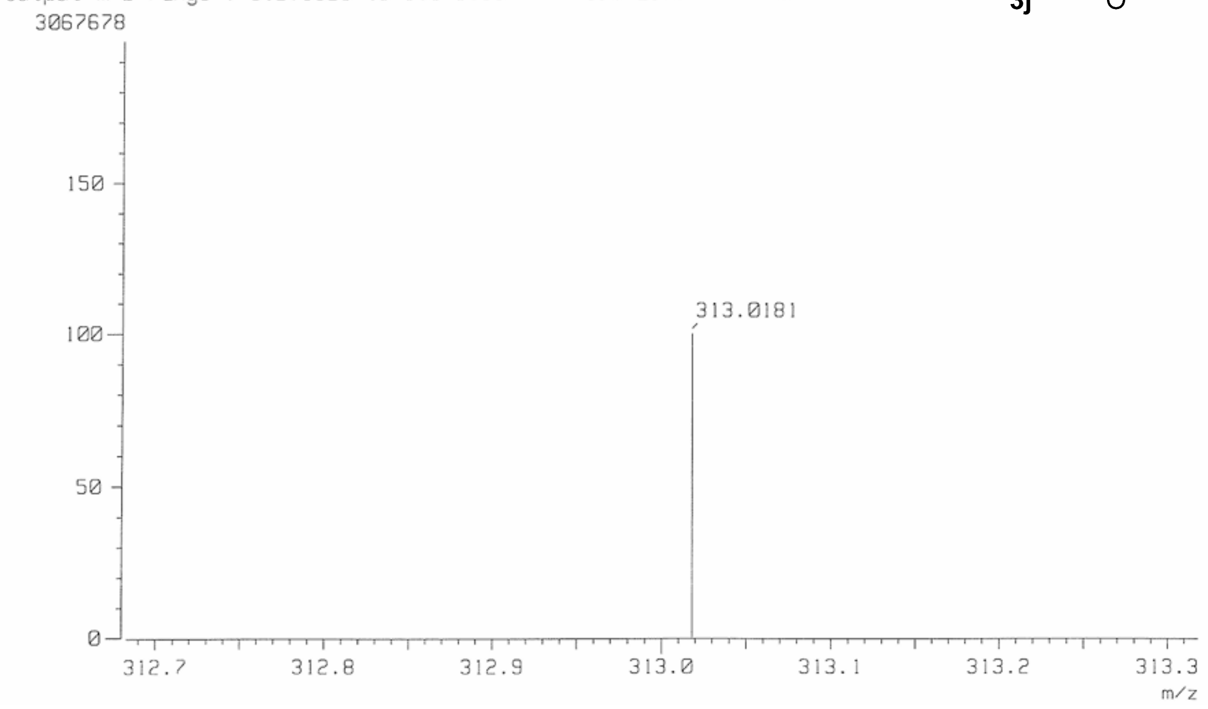

Compound 3k

[Elemental Composition]
Data : FAB-9-SW166-1-024 Date : 27-Apr-2026 12:29 Page: 1
Sample: High resolution GC Mass(JMS-700)
Note : SMlab-Mass Analysis & Research Center
Inlet : Direct Ion Mode : FAB+
RT : 2.40 min Scan#: 73
Elements : C 22/0, H 20/0, F 1/0, O 4/0, S 1/0
Mass Tolerance : 1000ppm, 3mmu if m/z < 3, 5mmu if m/z > 5
Unsaturation (U.S.) : -0.5 - 80.0

Observed m/z	Int%	Err[ppm / mmu]	U.S.	Composition
381.0593	45.7	-0.9 / -0.4	15.5	C 21 H 14 F O 4 S

[Mass Spectrum]
Data : FAB-9-SW166-1-024 Date : 27-Apr-2026 12:29
Sample: High resolution GC Mass(JMS-700)
Note : SMlab-Mass Analysis & Research Center
Inlet : Direct Ion Mode : FAB+
Spectrum Type : Normal Ion [MF-Linear]
RT : 2.40 min Scan# : 73
BP : m/z 307.1789 Int. : 63.39
Output m/z range : 380.7350 to 381.3939 Cut Level : 27.21 %

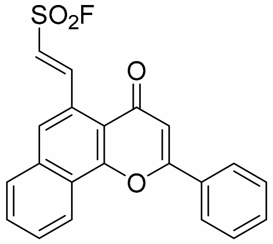

3k

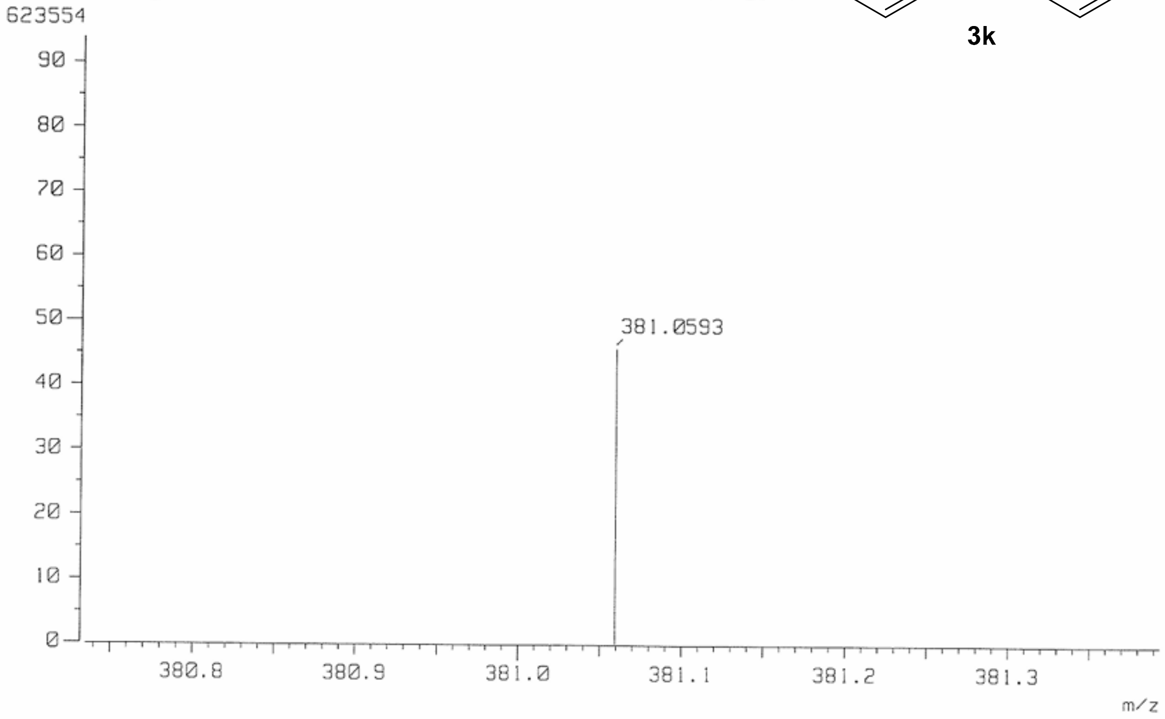

Compound 3l

```
[ Elemental Composition ]                                                    Page: 1
Data   : FAB-7-SW164-2-019              Date : 27-Apr-2026 12:14
Sample: High resolution GC Mass(JMS-700)
Note : SMlab-Mass Analysis & Research Center
Inlet : Direct                          Ion Mode : FAB+
RT : 0.55 min                           Scan#: (17,18)
Elements : C 18/0, H 13/0, F 1/0, O 4/0, S 1/0
Mass Tolerance       : 1000ppm, 3mmu if m/z < 3, 5mmu if m/z > 5
Unsaturation (U.S.) : -0.5 - 80.0

Observed m/z Int%   Err[ppm / mmu]    U.S.  Composition
  331.0443   100.0     +0.7 /  +0.2   12.5  C 17 H 12 F O 4 S
```

```
[ Mass Spectrum ]
Data   : FAB-7-SW164-2-019          Date : 27-Apr-2026 12:14
Sample: High resolution GC Mass(JMS-700)
Note : SMlab-Mass Analysis & Research Center
Inlet : Direct                      Ion Mode : FAB+
Spectrum Type : Normal Ion [MF-Linear]
RT : 0.55 min          Scan# : (17,18)
BP : m/z 331.0443      Int.  : 61.81
Output m/z range : 330.7085 to 331.3926      Cut Level : 60.29 %
```

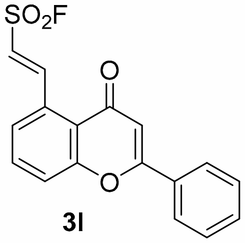

3l

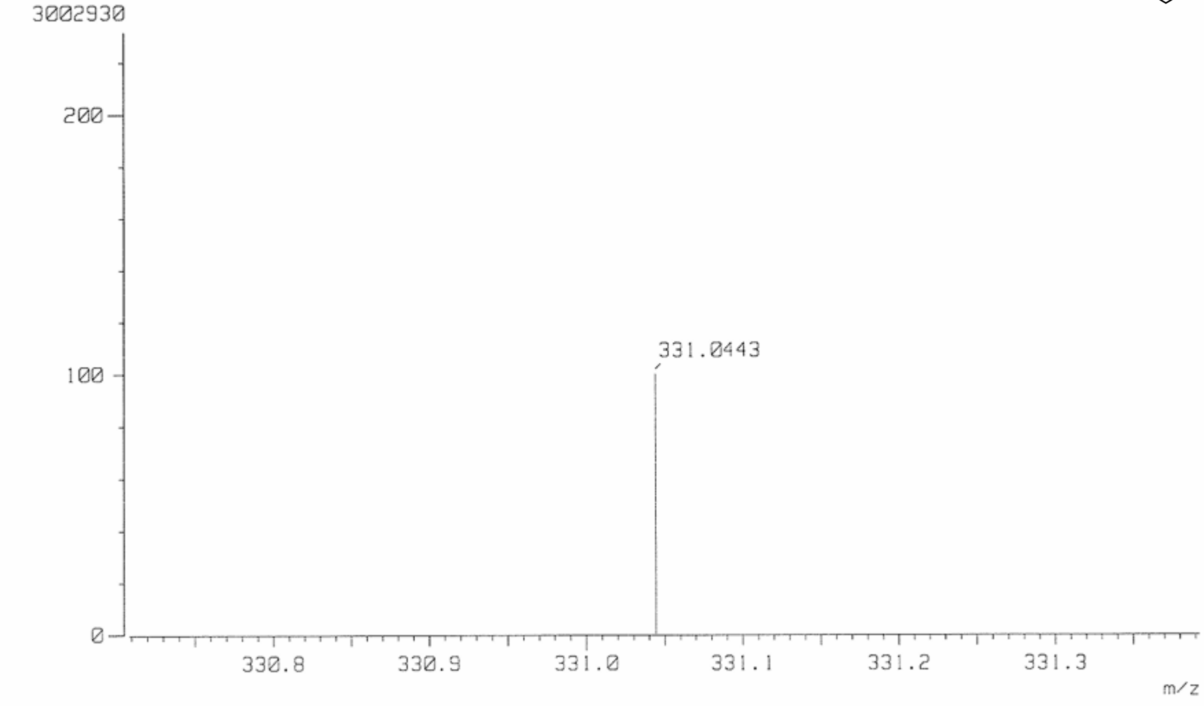

Compound 3m

```
[ Elemental Composition ]                                      Page: 1
Data   : FAB-16-SW175-006              Date : 27-Apr-2026 16:19
Sample: High resolution GC Mass(JMS-700)
Note : SMlab-Mass Analysis & Research Center
Inlet : Direct                         Ion Mode : FAB+
RT : 3.75 min                          Scan#: (112,115)
Elements : C 18/0, H 15/0, F 1/0, O 5/0, S 1/0
Mass Tolerance      : 1000ppm, 3mmu if m/z < 3, 5mmu if m/z > 5
Unsaturation (U.S.) : -0.5 - 80.0

Observed m/z Int%   Err[ppm / mmu]    U.S.  Composition
  361.0546   100.0     -0.1 /  +0.0   12.5  C 18 H 14 F O 5 S
```

```
[ Mass Spectrum ]
Data   : FAB-16-SW175-006          Date : 27-Apr-2026 16:19
Sample: High resolution GC Mass(JMS-700)
Note : SMlab-Mass Analysis & Research Center
Inlet : Direct                     Ion Mode : FAB+
Spectrum Type : Normal Ion [MF-Linear]
RT : 3.75 min          Scan# : (112,115)
BP : m/z 361.0546      Int.  : 99.95
Output m/z range : 360.7419 to 361.4188      Cut Level : 61.76 %
```

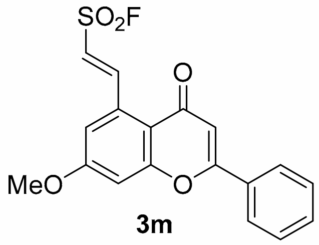

3m

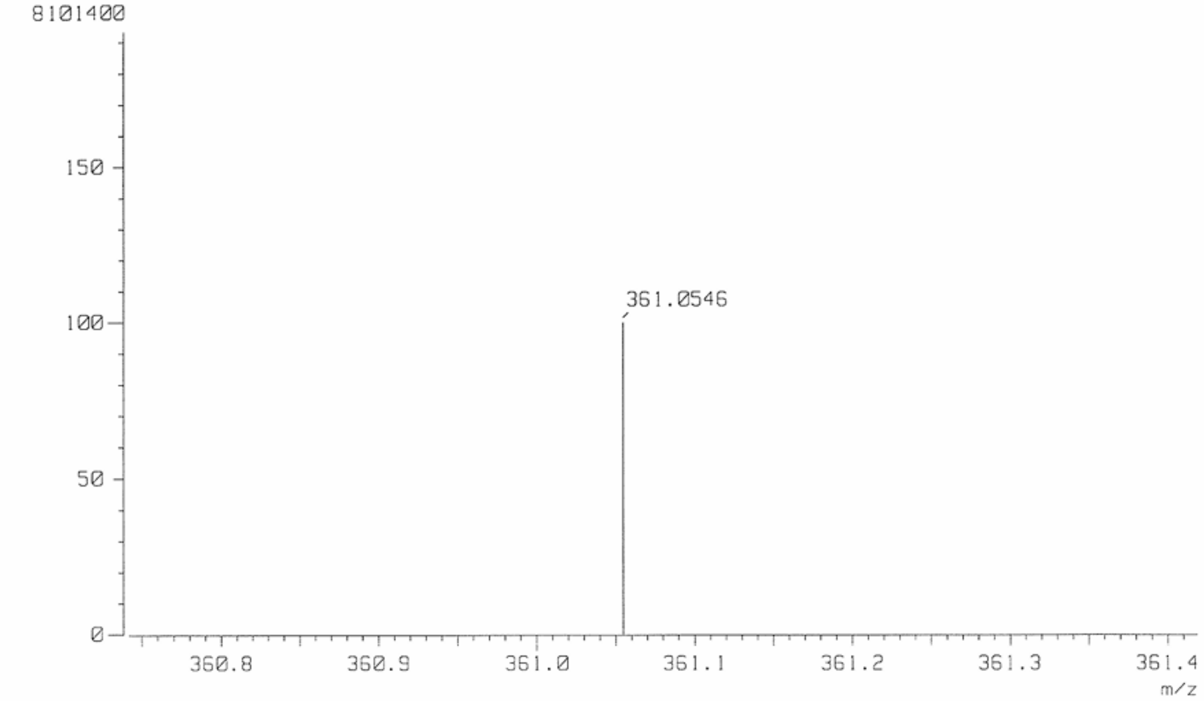

Compound 3n

```
[ Elemental Composition ]                                     Page: 1
Data   : FAB-15-SW174-4-005          Date : 27-Apr-2026 16:14
Sample: High resolution GC Mass(JMS-700)
Note : SMlab-Mass Analysis & Research Center
Inlet : Direct                       Ion Mode : FAB+
RT : 0.10 min                        Scan#: 4
Elements : C 18/0, H 15/0, F 1/0, O 5/0, S 1/0
Mass Tolerance      : 1000ppm, 3mmu if m/z < 3, 5mmu if m/z > 5
Unsaturation (U.S.) : -0.5 - 80.0

Observed m/z Int%   Err[ppm / mmu]    U.S.  Composition
  361.0542   100.0    -1.2 /  -0.4    12.5  C 18 H 14 F O 5 S
```

```
[ Mass Spectrum ]
Data   : FAB-15-SW174-4-005          Date : 27-Apr-2026 16:14
Sample: High resolution GC Mass(JMS-700)
Note : SMlab-Mass Analysis & Research Center
Inlet : Direct                       Ion Mode : FAB+
Spectrum Type : Normal Ion [MF-Linear]
RT : 0.10 min             Scan# : 4
BP : m/z 277.0856         Int.  : 100.00
Output m/z range : 360.6744 to 361.4392      Cut Level : 80.15 %
```

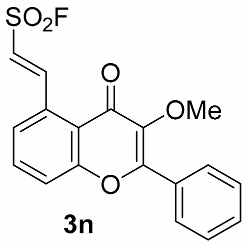

3n

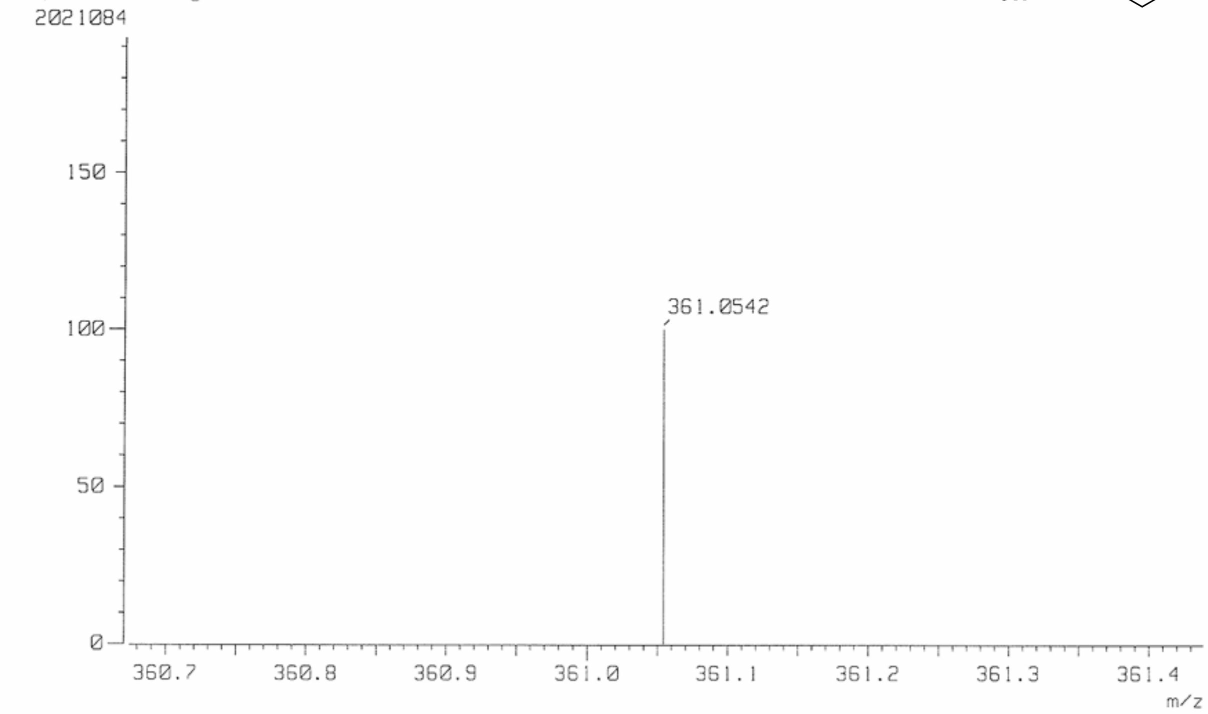

Compound 3o

```
[ Elemental Composition ]                                      Page: 1
Data   : FAB-14-SW173-3-002           Date : 27-Apr-2026 16:08
Sample: High resolution GC Mass(JMS-700)
Note : SMlab-Mass Analysis & Research Center
Inlet : Direct                        Ion Mode : FAB+
RT : 2.97 min                         Scan#: 90+88
Elements : C 18/0, H 14/0, F 1/0, O 5/0, S 1/0
Mass Tolerance       : 1000ppm, 3mmu if m/z < 3, 5mmu if m/z > 5
Unsaturation (U.S.) : -0.5 - 80.0

Observed m/z Int%   Err[ppm / mmu]    U.S.  Composition
  361.0542    69.4     -1.2 /  -0.4   12.5  C 18 H 14 F O 5 S
```

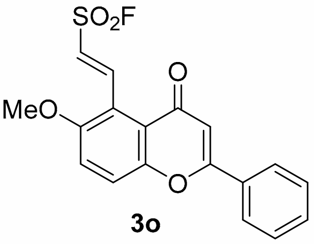

3o

```
[ Mass Spectrum ]
Data   : FAB-14-SW173-3-002           Date : 27-Apr-2026 16:08
Sample: High resolution GC Mass(JMS-700)
Note : SMlab-Mass Analysis & Research Center
Inlet : Direct                        Ion Mode : FAB+
Spectrum Type : Normal Ion [MF-Linear]
RT : 2.97 min          Scan# : 90+88
BP : m/z 307.1743      Int.  : 75.73
Output m/z range : 360.7346 to 361.4049      Cut Level : 55.88 %
```

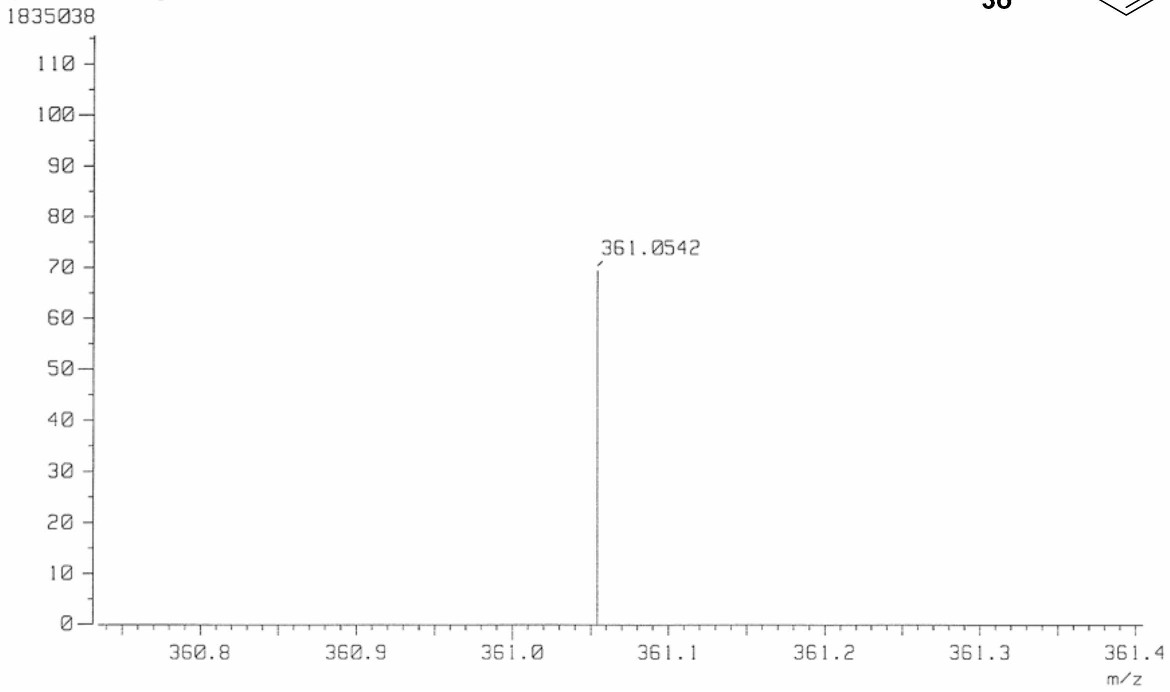

Compound 5a

[Elemental Composition] Page: 1
Data : FAB-1-KSW-199-3-002 Date : 11-Jul-2026 14:37
Sample: High resolution GC Mass(JMS-700)
Note : SMlab-Mass Analysis & Research Center
Inlet : Direct Ion Mode : FAB+
RT : 0.77 min Scan#: 24+39+24
Elements : C 16/0, H 11/0, F 1/0, O 5/0, S 1/0
Mass Tolerance : 1000ppm, 3mmu if m/z < 3, 5mmu if m/z > 5
Unsaturation (U.S.) : -0.5 - 80.0

Observed m/z	Int%	Err[ppm / mmu]	U.S.	Composition
305.0281	100.0	-0.9 / -0.3	11.5	C 15 H 10 F O 4 S

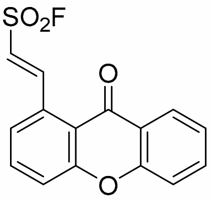

5a

[Mass Spectrum]
Data : FAB-1-KSW-199-3-002 Date : 11-Jul-2026 14:37
Sample: High resolution GC Mass(JMS-700)
Note : SMlab-Mass Analysis & Research Center
Inlet : Direct Ion Mode : FAB+
Spectrum Type : Normal Ion [MF-Linear]
RT : 0.77 min Scan# : 24+39+24
BP : m/z 305.0281 Int. : 23.87
Output m/z range : 304.7245 to 305.3199 Cut Level : 26.07 %
1537692

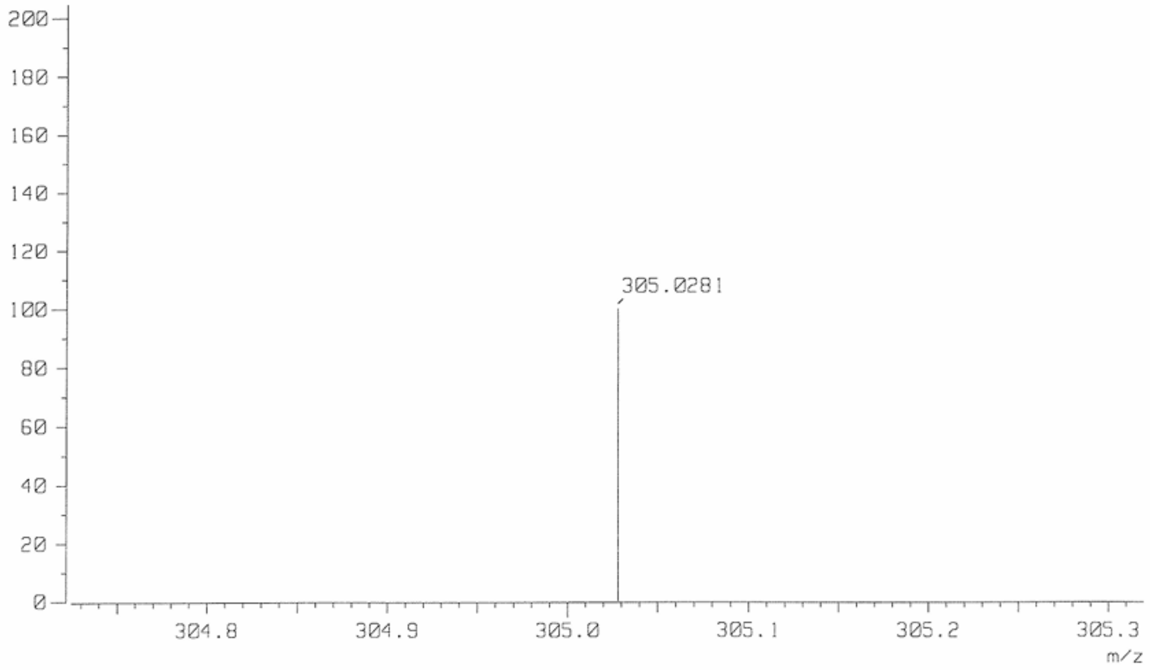

Compound 5aa

[Mass Spectrum]
Data : FAB-2-KSW-199-4-004 Date : 11-Jul-2026 14:43
Sample: High resolution GC Mass(JMS-700)
Note : SMlab-Mass Analysis & Research Center
Inlet : Direct Ion Mode : FAB+
Spectrum Type : Normal Ion [MF-Linear]
RT : 2.15 min Scan# : (65,66)
BP : m/z 307.0945 Int. : 13.01
Output m/z range : 412.6513 to 413.2512 Cut Level : 36.76 %

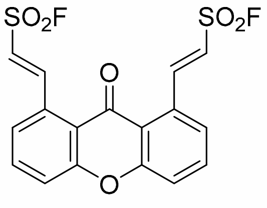

5aa

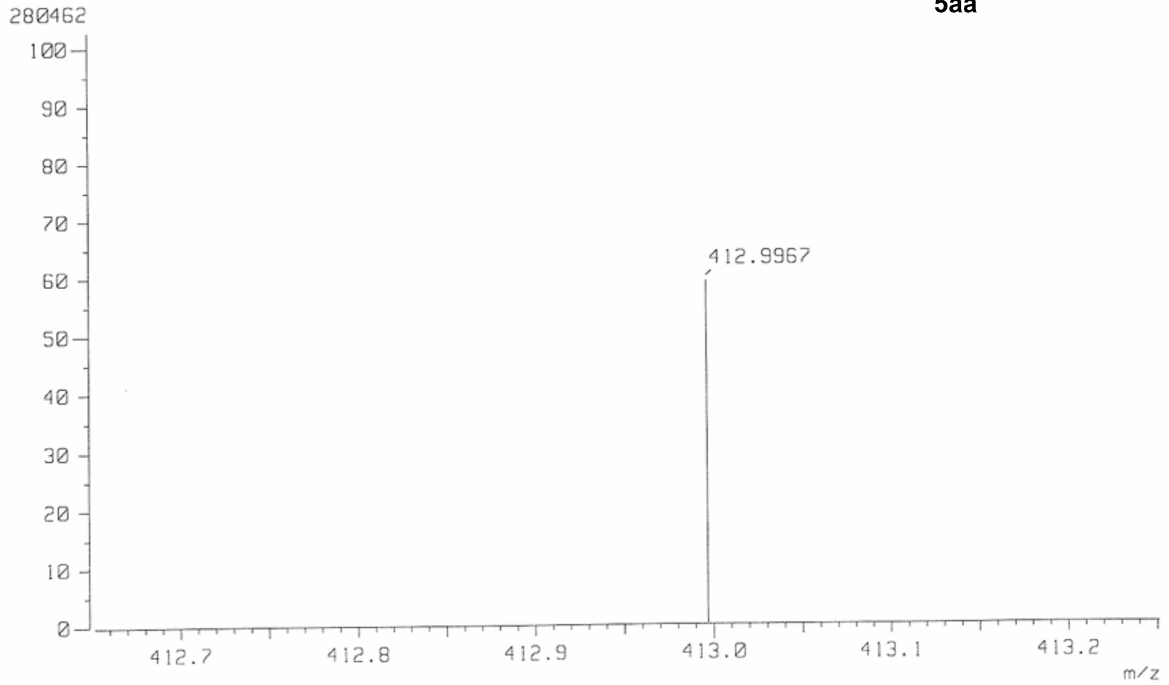

Compound 5b

```
[ Elemental Composition ]                                   Page: 1
Data   : FAB-Neg-3-KSW-239P1-023      Date : 23-Jul-2026 10:52
Sample: -
Note : -
Inlet : Direct                        Ion Mode : FAB-
RT : 2.20 min                         Scan#: 45
Elements : C 15/0, H 10/0, F 1/0, O 3/0, S 2/0
Mass Tolerance      : 1000ppm, 3mmu if m/z < 3, 5mmu if m/z > 5
Unsaturation (U.S.) : -0.5 - 80.0

Observed m/z Int%   Err[ppm / mmu]    U.S.  Composition
  319.9980   100.0     +1.0 /  +0.3   13.0  C 15 H 9 F O 3 S 2
```

```
[ Mass Spectrum ]
Data   : FAB-Neg-3-KSW-239P1-023      Date : 23-Jul-2026 10:52
Sample: -
Note : -
Inlet : Direct                        Ion Mode : FAB-
Spectrum Type : Normal Ion [MF-Linear]
RT : 2.20 min              Scan# : 45
BP : m/z 319.9980          Int.  : 1.87
Output m/z range : 319.6801 to 320.3906      Cut Level : 73.53 %
```

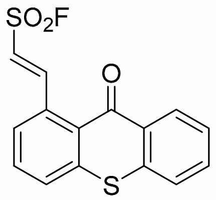

5b

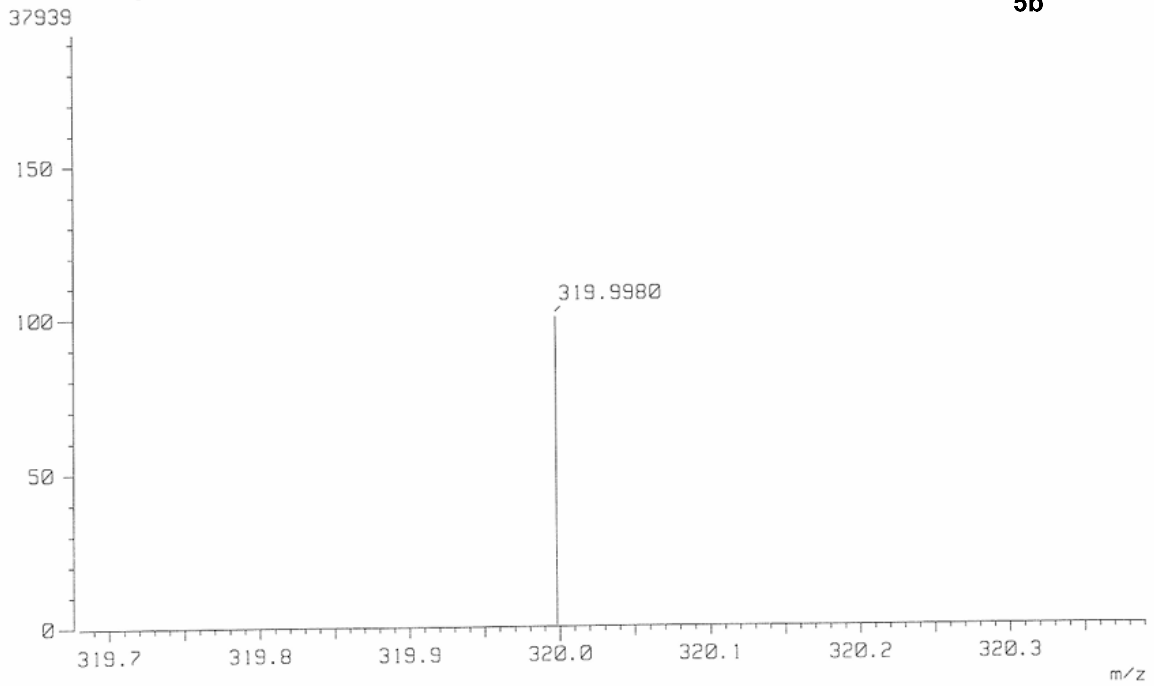

Compound 5ba

```
[ Elemental Composition ]                                              Page: 1
Data   : FAB-8-KSW216-P2-031          Date : 11-Jul-2026 17:00
Sample: High resolution GC Mass(JMS-700)
Note : SMlab-Mass Analysis & Research Center
Inlet : Direct                        Ion Mode : FAB+
RT : 3.85 min                         Scan#: (114,119)+68+62+81
Elements : C 18/0, H 12/0, F 2/0, N 2/0, O 5/0, S 3/0
Mass Tolerance       : 1000ppm, 3mmu if m/z < 3, 5mmu if m/z > 5
Unsaturation (U.S.) : -0.5 - 80.0

Observed m/z Int%   Err[ppm / mmu]    U.S.  Composition
  428.9736    13.6     -0.2 /  -0.1   14.5  C 17 H 11 F 2 O 5 S 3
```

```
[ Mass Spectrum ]
Data   : FAB-8-KSW216-P2-031          Date : 11-Jul-2026 17:00
Sample: High resolution GC Mass(JMS-700)
Note : SMlab-Mass Analysis & Research Center
Inlet : Direct                        Ion Mode : FAB+
Spectrum Type : Normal Ion [MF-Linear]
RT : 3.85 min             Scan# : (114,119)+68+62+81
BP : m/z 363.0194         Int.   : 36.51
Output m/z range : 428.6497 to 429.2751       Cut Level : 8.09 %
```

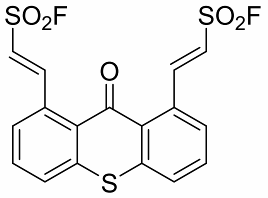

5ba

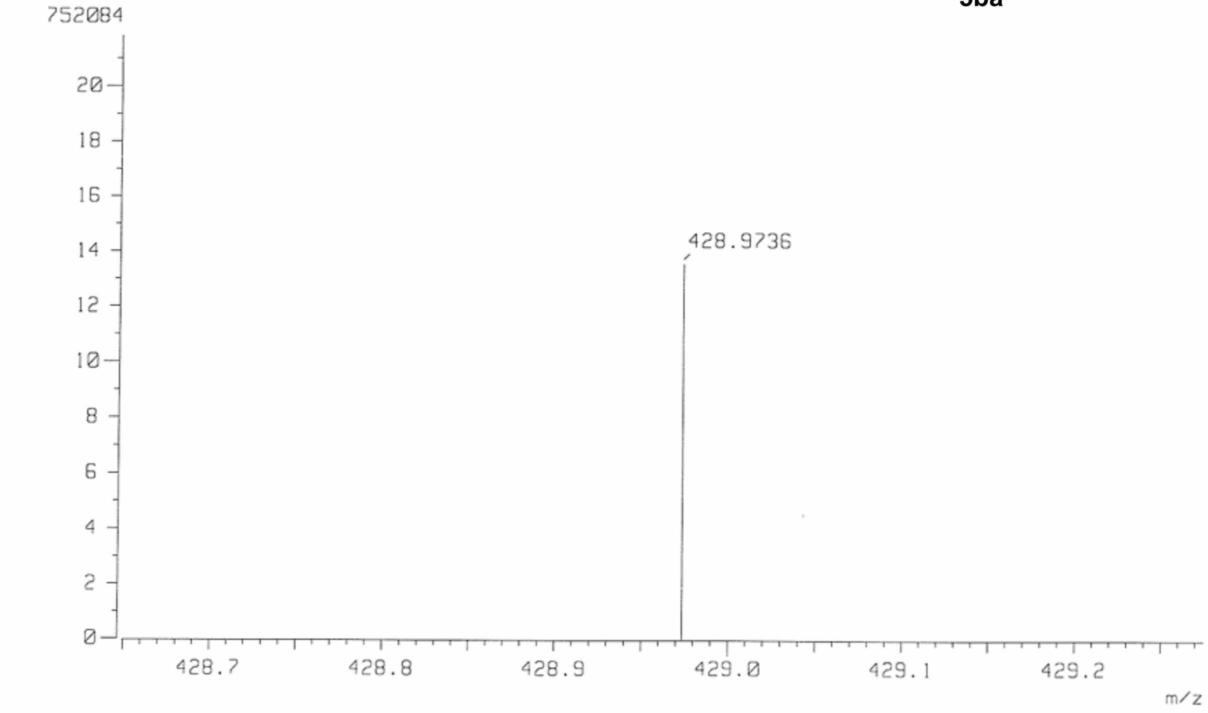

Compound 5c

[Elemental Composition]   Page: 1
Data   : FAB-13-KSW228-2-025   Date : 11-Jul-2026 16:24
Sample: High resolution GC Mass(JMS-700)
Note : SMlab-Mass Analysis & Research Center
Inlet : Direct   Ion Mode : FAB+
RT : 4.83 min   Scan#: 146+147+146
Elements : C 16/0, H 10/0, Cl 1/0, F 1/0, O 5/0, S 2/0
Mass Tolerance   : 1000ppm, 3mmu if m/z < 3, 5mmu if m/z > 5
Unsaturation (U.S.) : -0.5 - 80.0

Observed m/z	Int%	Err[ppm / mmu]	U.S.	Composition
354.9661	32.7	-1.3 / -0.5	12.5	C 15 H 9 Cl F O 3 S 2

[Mass Spectrum]
Data   : FAB-13-KSW228-2-025   Date : 11-Jul-2026 16:24
Sample: High resolution GC Mass(JMS-700)
Note : SMlab-Mass Analysis & Research Center
Inlet : Direct   Ion Mode : FAB+
Spectrum Type : Normal Ion [MF-Linear]
RT : 4.83 min   Scan# : 146+147+146
BP : m/z 281.0687   Int.   : 21.11
Output m/z range : 354.6222 to 355.2382   Cut Level : 11.35 %

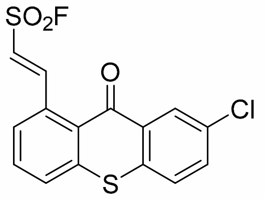

5c

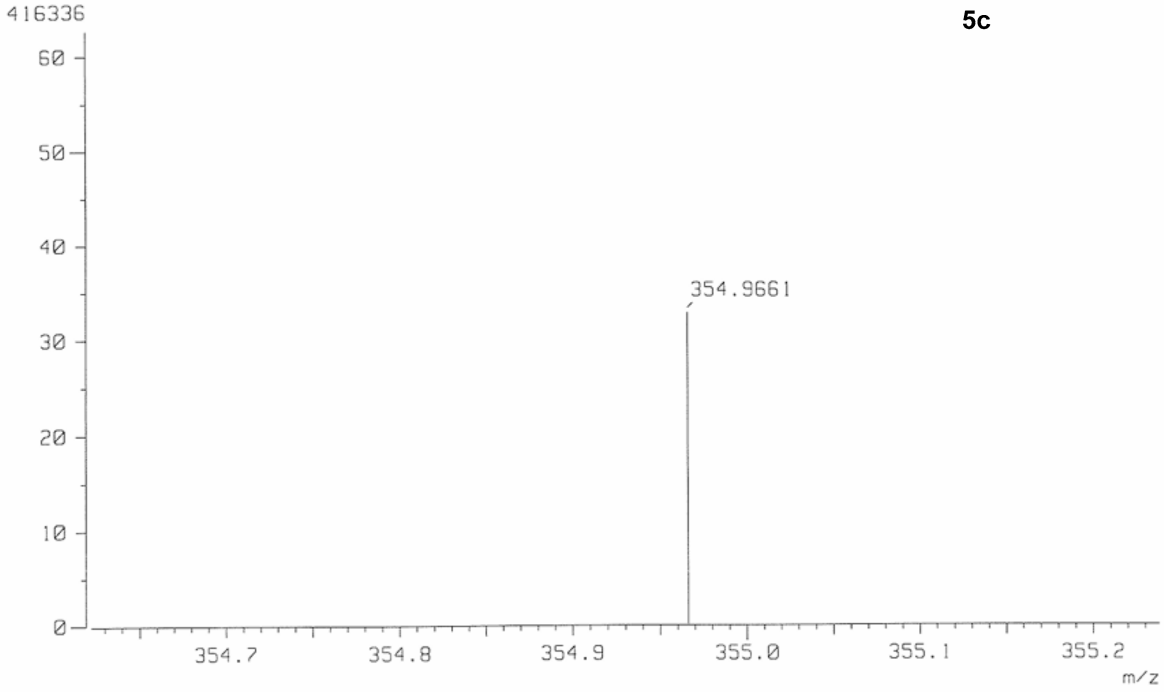

Compound 5c'

```
[ Elemental Composition ]                                   Page: 1
Data   : FAB-14-KSW228-3-024          Date : 11-Jul-2026 16:19
Sample: High resolution GC Mass(JMS-700)
Note : SMlab-Mass Analysis & Research Center
Inlet : Direct                         Ion Mode : FAB+
RT : 0.07 min                          Scan#: 3+4
Elements : C 16/0, H 10/0, Cl 1/0, F 1/0, O 5/0, S 2/0
Mass Tolerance       : 1000ppm, 3mmu if m/z < 3, 5mmu if m/z > 5
Unsaturation (U.S.) : -0.5 - 80.0

Observed m/z Int%   Err[ppm / mmu]    U.S.  Composition
  354.9669    50.2     +1.1 /  +0.4    12.5  C 15 H 9 Cl F O 3 S 2
```

```
[ Mass Spectrum ]
Data   : FAB-14-KSW228-3-024          Date : 11-Jul-2026 16:19
Sample: High resolution GC Mass(JMS-700)
Note : SMlab-Mass Analysis & Research Center
Inlet : Direct                         Ion Mode : FAB+
Spectrum Type : Normal Ion [MF-Linear]
RT : 0.07 min           Scan# : 3+4
BP : m/z 270.9993       Int.  : 21.07
Output m/z range : 354.6441 to 355.3093      Cut Level : 41.18 %
```

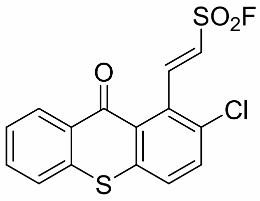

5c'

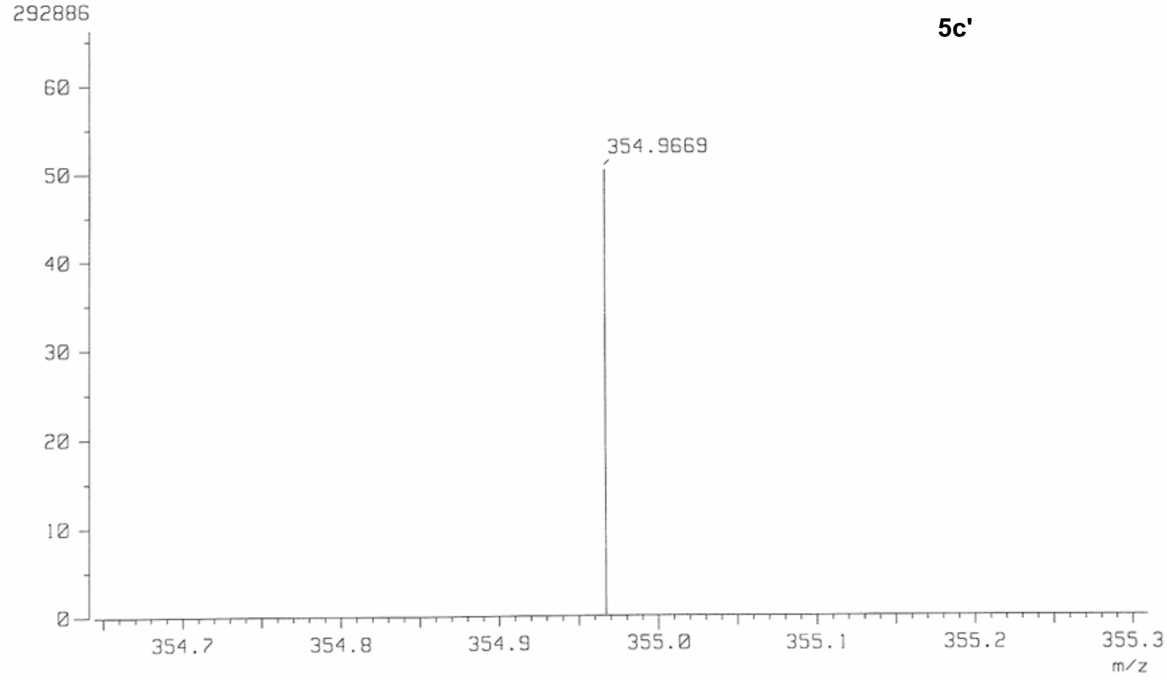

Compound 5d

[Elemental Composition] Page: 1
Data : FAB-12-KSW227-3-026 Date : 11-Jul-2026 16:32
Sample: High resolution GC Mass(JMS-700)
Note : SMlab-Mass Analysis & Research Center
Inlet : Direct Ion Mode : FAB+
RT : 2.74 min Scan#: 83
Elements : C 17/0, H 10/0, F 4/0, O 5/0, S 2/0
Mass Tolerance : 1000ppm, 3mmu if m/z < 3, 5mmu if m/z > 5
Unsaturation (U.S.) : -0.5 - 80.0

Observed m/z	Int%	Err[ppm / mmu]	U.S.	Composition
388.9924	63.0	-1.3 / -0.5	12.5	C 16 H 9 F 4 O 3 S 2

[Mass Spectrum]
Data : FAB-12-KSW227-3-026 Date : 11-Jul-2026 16:32
Sample: High resolution GC Mass(JMS-700)
Note : SMlab-Mass Analysis & Research Center
Inlet : Direct Ion Mode : FAB+
Spectrum Type : Normal Ion [MF-Linear]
RT : 2.74 min Scan# : 83
BP : m/z 305.0283 Int. : 25.27
Output m/z range : 388.7572 to 389.2343 Cut Level : 33.82 %

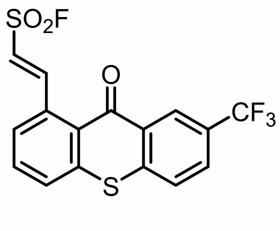

5d

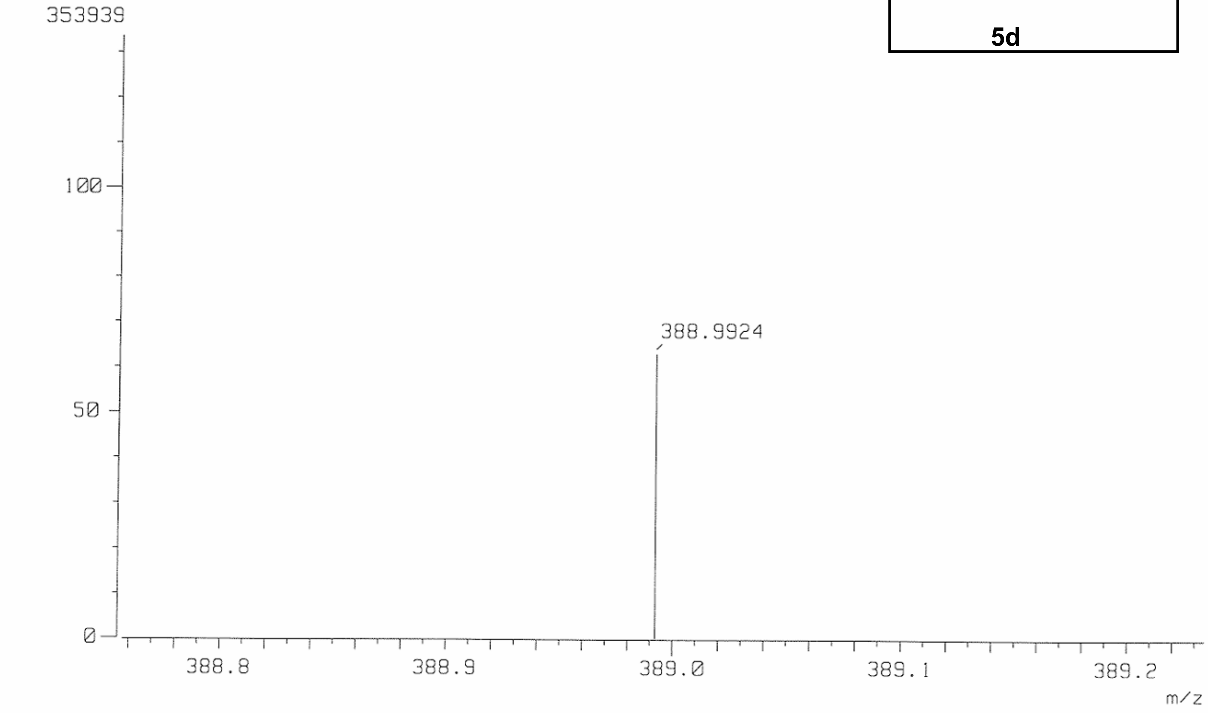

Compound 5e

```
[ Elemental Composition ]                                        Page: 1
Data   : FAB-11-KSW226-3-028          Date : 11-Jul-2026 16:38
Sample: High resolution GC Mass(JMS-700)
Note : SMlab-Mass Analysis & Research Center
Inlet : Direct                        Ion Mode : FAB+
RT : 1.60 min                         Scan#: 49+66+49
Elements : C 17/0, H 15/0, F 1/0, N 1/0, O 4/0, S 1/0
Mass Tolerance       : 1000ppm, 3mmu if m/z < 3, 5mmu if m/z > 5
Unsaturation (U.S.) : -0.5 - 80.0
```

Observed m/z	Int%	Err[ppm / mmu]	U.S.	Composition
318.0600	72.1	+0.0 / +0.0	11.5	C 16 H 13 F N O 3 S

```
[ Mass Spectrum ]
Data   : FAB-11-KSW226-3-028          Date : 11-Jul-2026 16:38
Sample: High resolution GC Mass(JMS-700)
Note : SMlab-Mass Analysis & Research Center
Inlet : Direct                        Ion Mode : FAB+
Spectrum Type : Normal Ion [MF-Linear]
RT : 1.60 min           Scan# : 49+66+49
BP : m/z 234.1028       Int.  : 26.31
Output m/z range : 317.7985 to 318.4098      Cut Level : 39.71 %
```

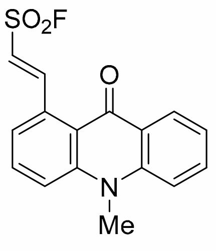

5e

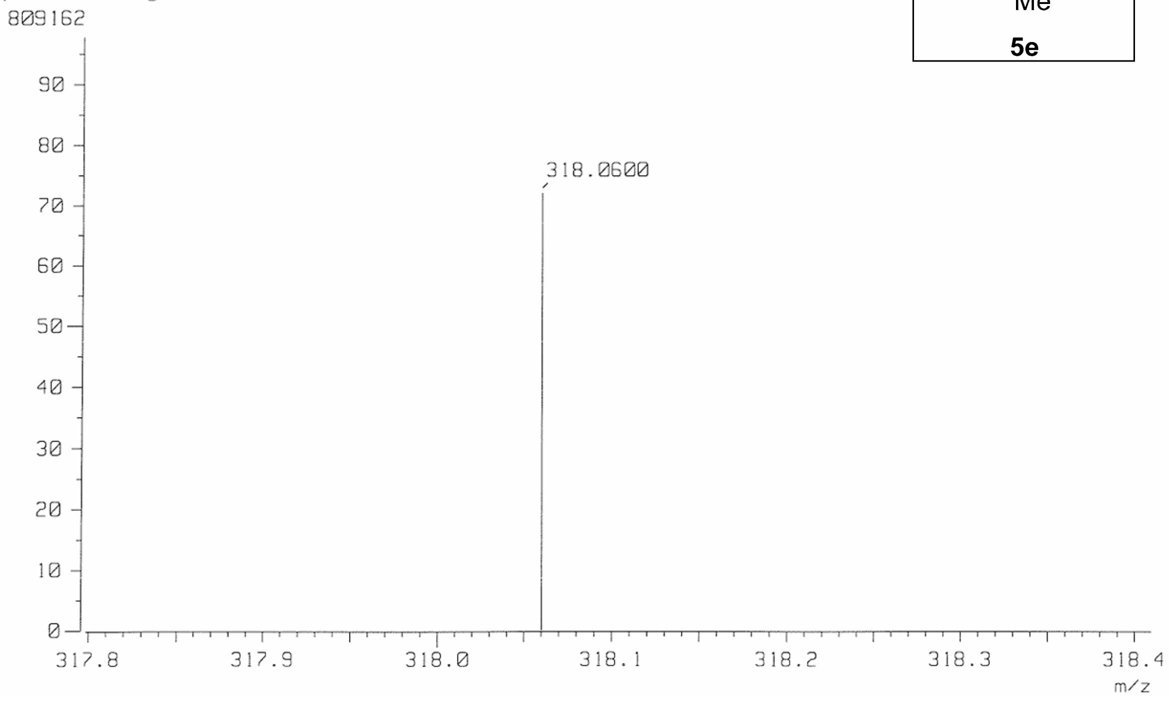

Compound 5f

[Elemental Composition]   Page: 1
Data   : FAB-Neg-1-KSW-238-p1-R-032   Date : 23-Jul-2026 12:45
Sample: -
Note : -
Inlet : Direct   Ion Mode : FAB-
RT : 0.07 min   Scan#: 2
Elements : C 13/0, H 10/0, F 1/0, O 4/0, S 1/0
Mass Tolerance   : 1000ppm, 3mmu if m/z < 3, 5mmu if m/z > 5
Unsaturation (U.S.) : -0.5 - 80.0

Observed m/z	Int%	Err[ppm / mmu]	U.S.	Composition
266.0042	100.0	-2.9 / -0.8	10.0	C 12 H 7 F O 4 S

[Mass Spectrum]
Data : FAB-Neg-1-KSW-238-p1-R-032   Date : 23-Jul-2026 12:45
Sample: -
Note : -
Inlet : Direct   Ion Mode : FAB-
Spectrum Type : Normal Ion [MF-Linear]
RT : 0.07 min   Scan# : 2
BP : m/z 266.0042   Int. : 160.44
Output m/z range : 265.6537 to 266.2756   Cut Level : 72.06 %

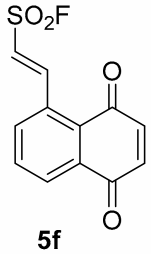

5f

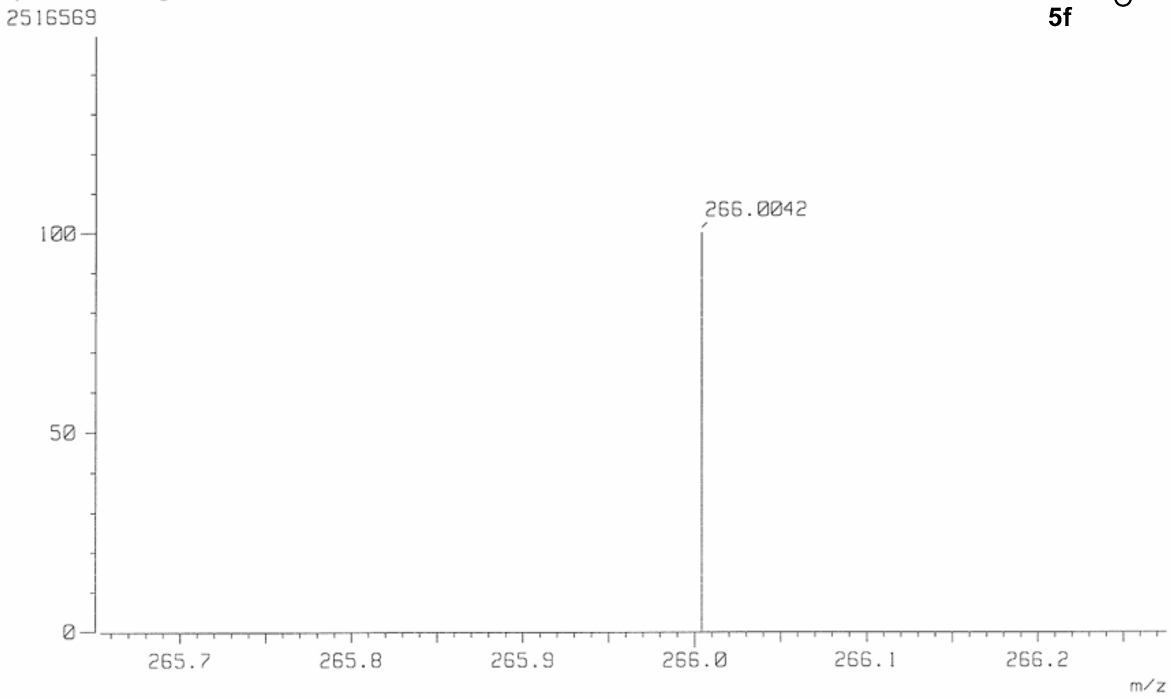

Compound 5fa

```
[ Elemental Composition ]                                          Page: 1
Data   : FAB-Neg-2-KSW-238P2-019      Date : 23-Jul-2026 10:35
Sample: -
Note : -
Inlet : Direct                         Ion Mode : FAB-
RT : 0.60 min                          Scan#: 13
Elements : C 15/0, H 10/0, F 2/0, O 6/0, S 2/0
Mass Tolerance      : 1000ppm, 3mmu if m/z < 3, 5mmu if m/z > 5
Unsaturation (U.S.) : -0.5 - 80.0

Observed m/z Int%   Err[ppm / mmu]    U.S.  Composition
  373.9729   100.0     -0.3 /  -0.1   12.0  C 14 H 8 F 2 O 6 S 2
```

```
[ Mass Spectrum ]
Data   : FAB-Neg-2-KSW-238P2-019      Date : 23-Jul-2026 10:35
Sample: -
Note : -
Inlet : Direct                        Ion Mode : FAB-
Spectrum Type : Normal Ion [MF-Linear]
RT : 0.60 min          Scan# : 13
BP : m/z 373.9729      Int.  : 144.52
Output m/z range : 373.6727 to 374.2415      Cut Level : 75.00 %
2681888
```

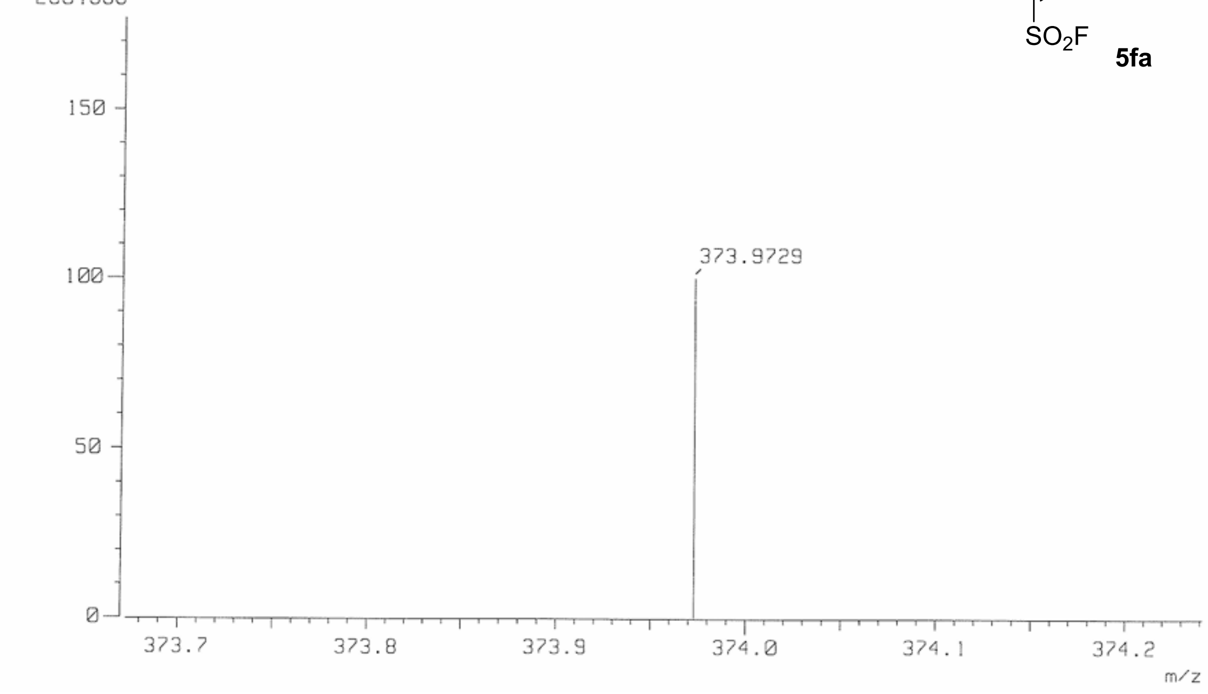

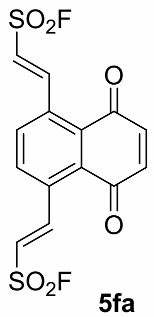

5fa

Compound 5g

```
[ Elemental Composition ]                                          Page: 1
Data   : FAB-5-KSW-206-P1-011         Date : 11-Jul-2026 15:06
Sample: High resolution GC Mass(JMS-700)
Note : SMlab-Mass Analysis & Research Center
Inlet : Direct                        Ion Mode : FAB+
RT : 2.67 min                         Scan#: 81+82+108
Elements : C 14/0, H 11/0, F 1/0, O 5/0, S 1/0
Mass Tolerance      : 1000ppm, 3mmu if m/z < 3, 5mmu if m/z > 5
Unsaturation (U.S.) : -0.5 - 80.0

Observed m/z Int%   Err[ppm / mmu]    U.S.  Composition
  281.0280    73.1     -1.3 /  -0.4    9.5  C 13 H 10 F O 4 S
```

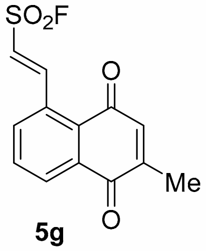

5g

```
[ Mass Spectrum ]
Data   : FAB-5-KSW-206-P1-011         Date : 11-Jul-2026 15:06
Sample: High resolution GC Mass(JMS-700)
Note : SMlab-Mass Analysis & Research Center
Inlet : Direct                        Ion Mode : FAB+
Spectrum Type : Normal Ion [MF-Linear]
RT : 2.67 min           Scan# : 81+82+108
BP : m/z 221.1380       Int.  : 26.44
Output m/z range : 280.7342 to 281.3323      Cut Level : 52.21 %
```

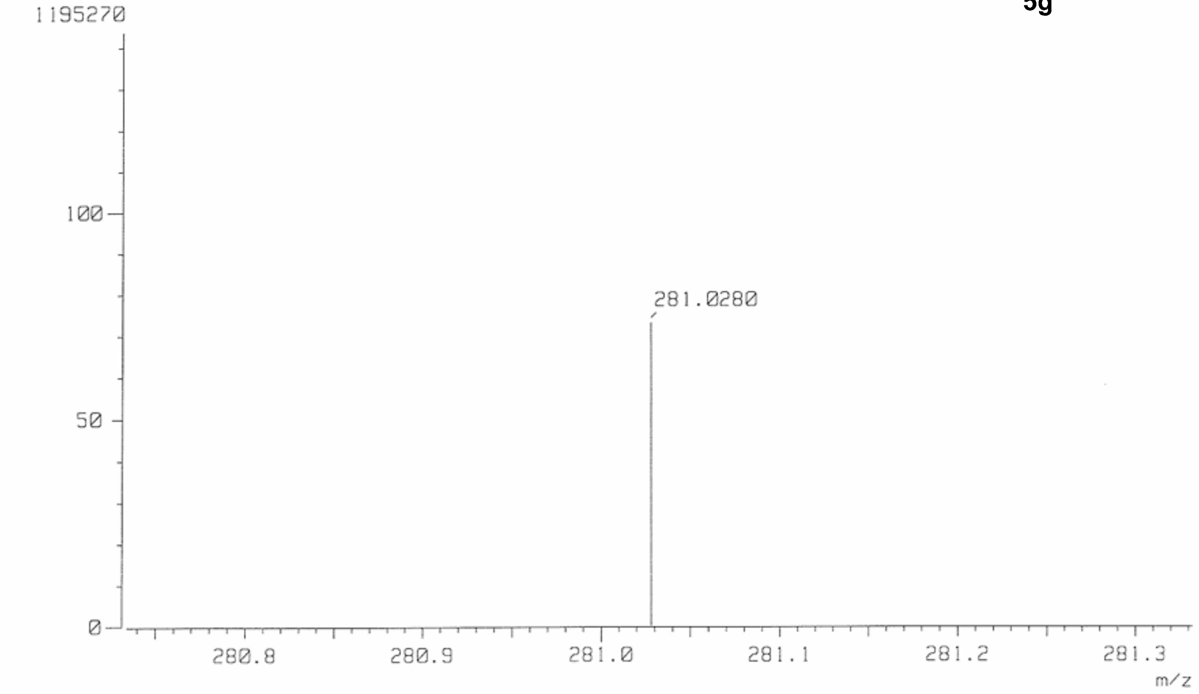

Compound 5ga

[Elemental Composition]   Page: 1
Data   : FAB-Neg-4-KSW-242-P2-029   Date : 23-Jul-2026 11:32
Sample: -
Note : -
Inlet : Direct   Ion Mode : FAB-
RT : 0.05 min   Scan#: 2+2+2+3+2+2
Elements : C 15/0, H 12/0, F 2/0, O 6/0, S 2/0
Mass Tolerance   : 1000ppm, 3mmu if m/z < 3, 5mmu if m/z > 5
Unsaturation (U.S.) : -0.5 - 80.0

Observed m/z	Int%	Err[ppm / mmu]	U.S.	Composition
387.9889	100.0	+0.6 / +0.2	12.0	C 15 H 10 F 2 O 6 S 2

[Mass Spectrum]
Data   : FAB-Neg-4-KSW-242-P2-029   Date : 23-Jul-2026 11:32
Sample: -
Note : -
Inlet : Direct   Ion Mode : FAB-
Spectrum Type : Normal Ion [MF-Linear]
RT : 0.05 min   Scan# : 2+2+2+3+2+2
BP : m/z 387.9889   Int. : 66.52
Output m/z range : 387.5896 to 388.3398   Cut Level : 70.59 %

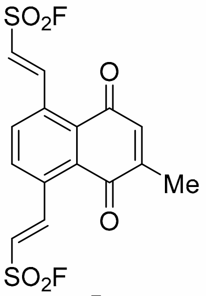

5ga

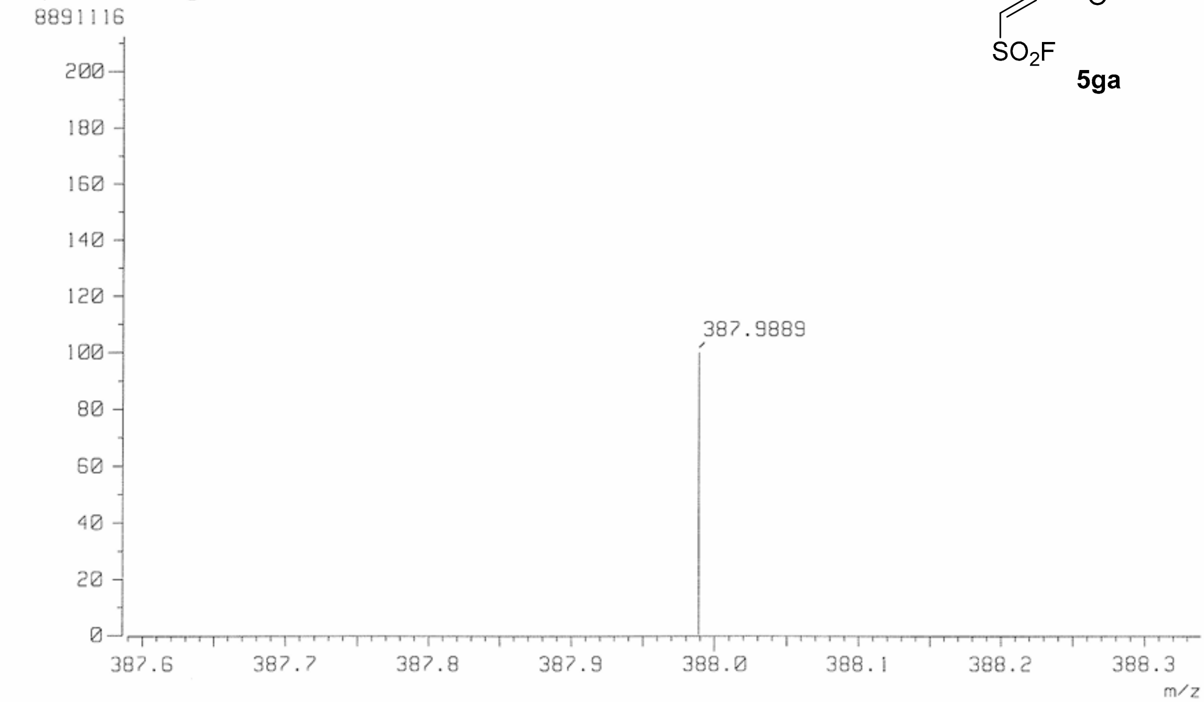

Compound 5h

```
[ Elemental Composition ]                                                Page: 1
Data   : FAB-9-KSW221-1-030              Date : 11-Jul-2026 16:47
Sample: High resolution GC Mass(JMS-700)
Note : SMlab-Mass Analysis & Research Center
Inlet : Direct                           Ion Mode : FAB+
RT : 10.47 min                           Scan#: 315+312+315
Elements : C 15/0, H 10/0, F 1/0, N 2/0, O 5/0, S 1/0
Mass Tolerance       : 1000ppm, 3mmu if m/z < 3, 5mmu if m/z > 5
Unsaturation (U.S.)  : -0.5 - 80.0

Observed m/z  Int%    Err[ppm / mmu]    U.S.  Composition
  297.0229    36.2      -1.5 /  -0.4     9.5  C 13 H 10 F O 5 S
```

```
[ Mass Spectrum ]
Data   : FAB-9-KSW221-1-030          Date : 11-Jul-2026 16:47
Sample: High resolution GC Mass(JMS-700)
Note : SMlab-Mass Analysis & Research Center
Inlet : Direct                       Ion Mode : FAB+
Spectrum Type : Normal Ion [MF-Linear]
RT : 10.47 min          Scan# : 315+312+315
BP : m/z 199.0488       Int.   : 99.99
Output m/z range : 296.7543 to 297.2397      Cut Level : 16.18 %
```

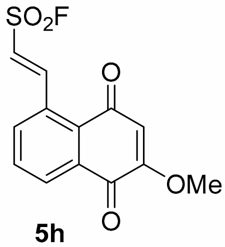

5h

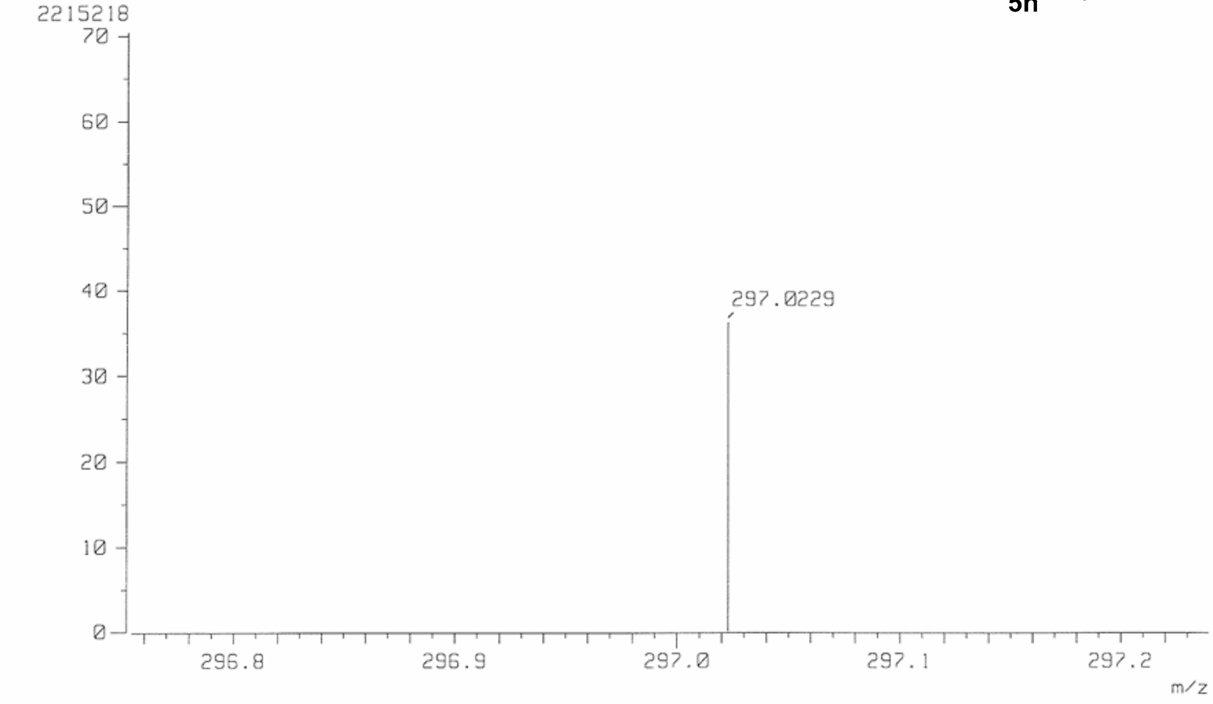

Compound 6a

```
[ Elemental Composition ]                                          Page: 1
Data   : FAB-1-KSW-267-P-007          Date : 30-Jul-2026 16:00
Sample: High resolution GC Mass(JMS-700)
Note : SMlab-Mass Analysis & Research Center
Inlet : Direct                        Ion Mode : FAB+
RT : 1.37 min                         Scan#: 42+36+39+36
Elements : C 27/0, H 30/0, N 1/0, O 10/0, S 1/0
Mass Tolerance       : 1000ppm, 3mmu if m/z < 3, 5mmu if m/z > 5
Unsaturation (U.S.) : -0.5 - 80.0

Observed m/z Int%   Err[ppm / mmu]    U.S.  Composition
  530.1486     3.4     +0.3 /  +0.1   14.5  C 26 H 28 N O 9 S
```

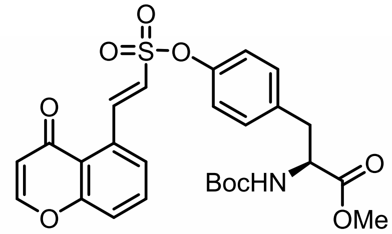

6a

```
[ Mass Spectrum ]
Data   : FAB-1-KSW-267-P-007          Date : 30-Jul-2026 16:00
Sample: High resolution GC Mass(JMS-700)
Note : SMlab-Mass Analysis & Research Center
Inlet : Direct                        Ion Mode : FAB+
Spectrum Type : Normal Ion [MF-Linear]
RT : 1.37 min             Scan# : 42+36+39+36
BP : m/z 430.1372         Int.   : 99.96
Output m/z range : 529.9083 to 530.4831      Cut Level : 2.57 %
```

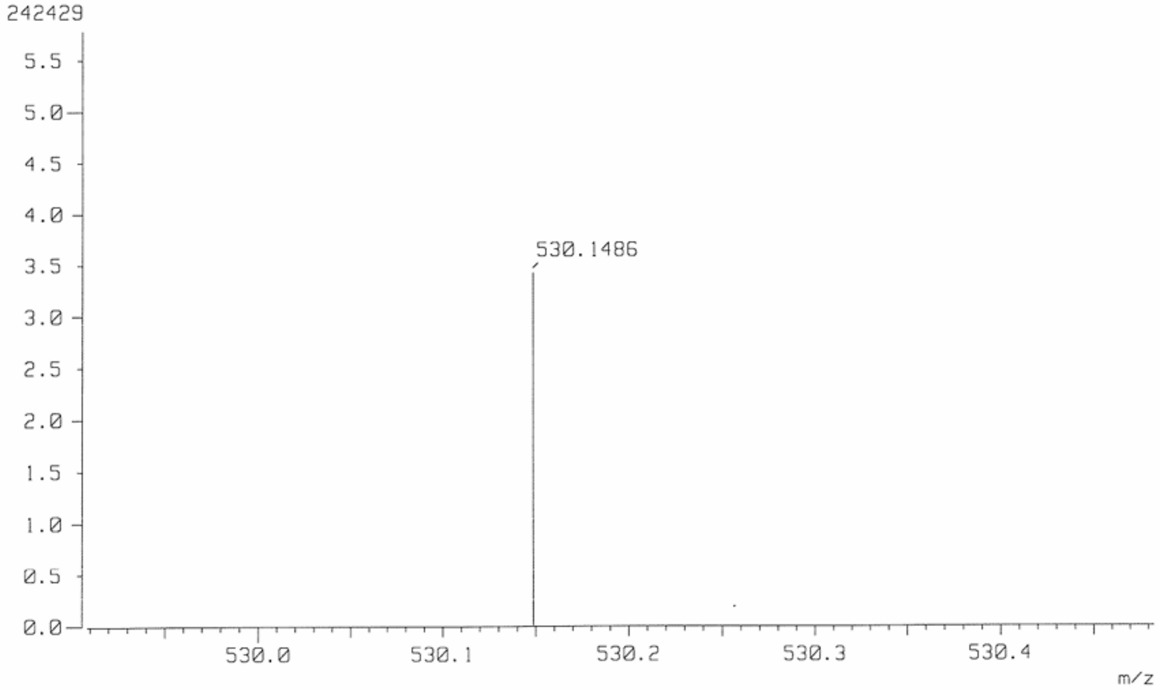

Compound 6b

```
[ Elemental Composition ]                                              Page: 1
Data   : FAB-3-KSW-287-P-010          Date : 30-Jul-2026 16:08
Sample: High resolution GC Mass(JMS-700)
Note : SMlab-Mass Analysis & Research Center
Inlet : Direct                        Ion Mode : FAB+
RT : 0.10 min                         Scan#: 4+2+2+4+7
Elements : C 20/0, H 20/0, N 1/0, O 7/0, S 1/0
Mass Tolerance       : 1000ppm, 3mmu if m/z < 3, 5mmu if m/z > 5
Unsaturation (U.S.) : -0.5 - 80.0

Observed m/z Int%   Err[ppm / mmu]    U.S.  Composition
  386.0692   100.0     -1.6 /  -0.6   13.5  C 19 H 16 N O 6 S
```

```
[ Mass Spectrum ]
Data   : FAB-3-KSW-287-P-010          Date : 30-Jul-2026 16:08
Sample: High resolution GC Mass(JMS-700)
Note : SMlab-Mass Analysis & Research Center
Inlet : Direct                        Ion Mode : FAB+
Spectrum Type : Normal Ion [MF-Linear]
RT : 0.10 min           Scan# : 4+2+2+4+7
BP : m/z 386.0692       Int.   : 28.44
Output m/z range : 385.7345 to 386.3854        Cut Level : 72.79 %
```

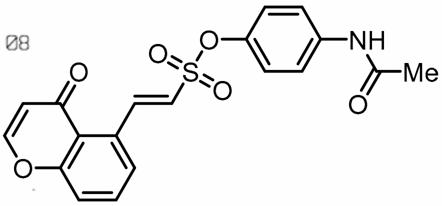

6b

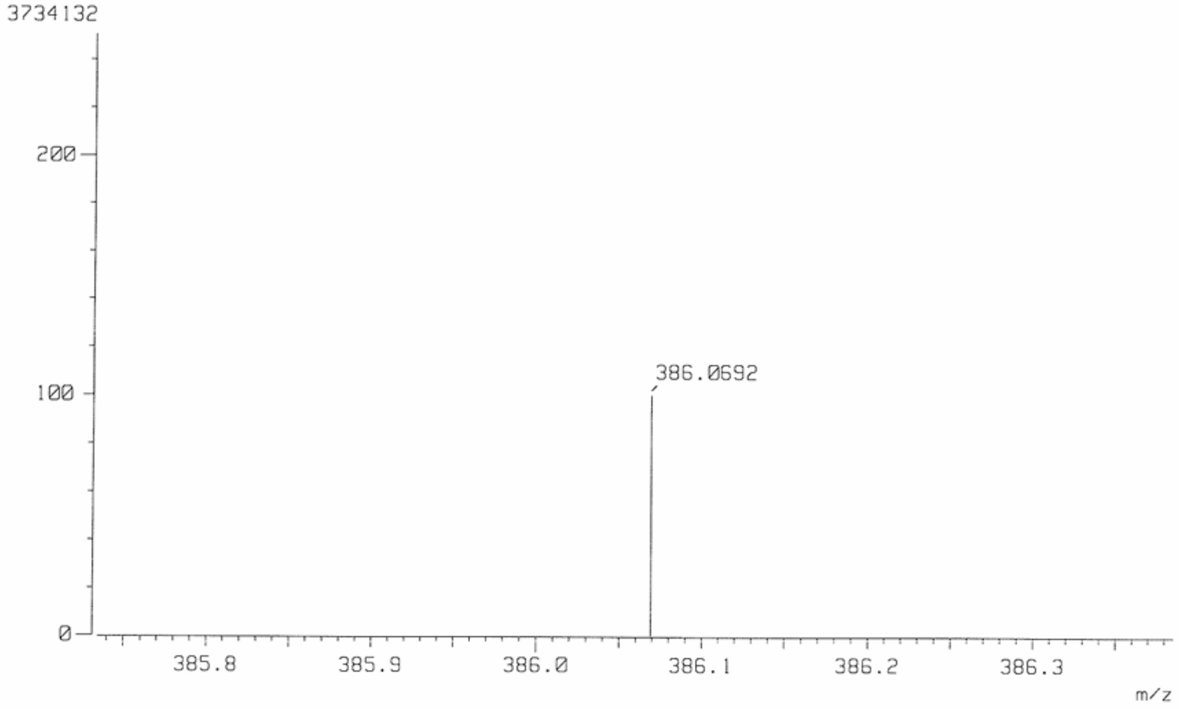

Compound 6c

```
[ Elemental Composition ]                                          Page: 1
Data   : EI-2-KSW-271-P-004            Date : 30-Jul-2026 13:45
Sample: High resolution GC Mass (JMS-700)
Note : SMLab-Mass Analysis & Research Center
Inlet : Direct                          Ion Mode : EI+
RT : 0.50 min                           Scan#: 16
Elements : C 17/0, H 17/0, F 1/0, N 1/0, O 6/0, S 1/0
Mass Tolerance       : 1000ppm, 3mmu if m/z < 3, 5mmu if m/z > 5
Unsaturation (U.S.) : -0.5 - 80.0

Observed m/z Int%   Err[ppm / mmu]    U.S.  Composition
  341.0730   100.0     -1.0 /  -0.4    9.0  C 15 H 16 F N O 5 S
```

```
[ Mass Spectrum ]
Data   : EI-2-KSW-271-P-004           Date : 30-Jul-2026 13:45
Sample: High resolution GC Mass (JMS-700)
Note : SMLab-Mass Analysis & Research Center
Inlet : Direct                        Ion Mode : EI+
Spectrum Type : Normal Ion [MF-Linear]
RT : 0.50 min          Scan# : 16
BP : m/z 341.0730      Int.  : 71.45
Output m/z range : 340.6863 to 341.4416      Cut Level : 79.41 %
```

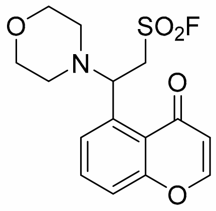

6c

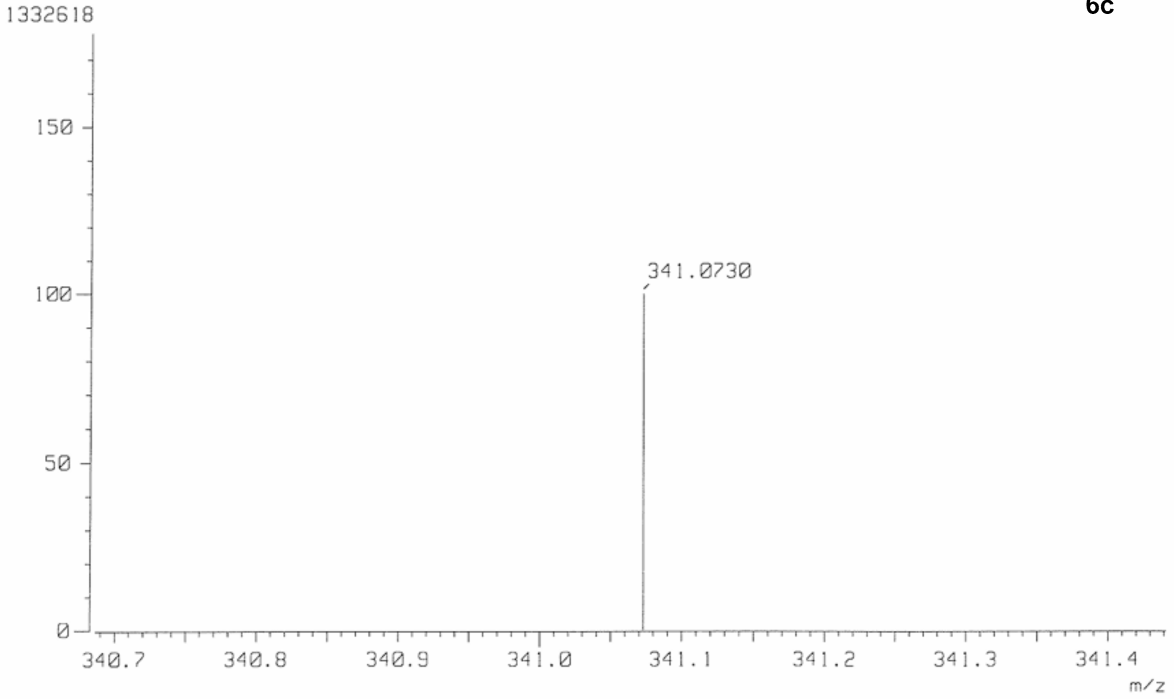

Supplement: Supplementary file 1 [file molecules-31-03094-s001.zip › molecules-4510115-supplementary.pdf]
